# Supplementary material for: Mapping the Distributions of Mosquitoes and Mosquito-Borne Arboviruses in China
Source: Viruses. 2022 Mar 27;14(4):691. doi: 10.3390/v14040691 (PMC9031751; doi:10.3390/v14040691)
Supplement: Supplementary file 1 [file viruses-14-00691-s001.zip › Supplementary materials.pdf]

Supplementary Material for:

Mapping the distributions of mosquitoes and mosquito-borne arboviruses in China

Page	Item
4	Supplementary Figure S1 The flow diagram of literature review
5	Supplementary Table S1. The specific references for all 339 mosquito species in China from 1954 to 2020
14	Supplementary Table S2. Information of data sources in this study.
15	Supplementary Table S3. Potential risk factors at the county level used in the BRT model for mosquito species and 2-stage GBRT model for Dengue and JE.
17	Supplementary Table S4. Clustering analysis of eco-climatic predictors at the county level based on pairwise Pearson correlation coefficients.
18	Supplementary Figure S2 The spatial distribution of the 1228 counties with at least one record of mosquitoes (yellow) from 1954 to 2020, China
19	Supplementary Figure S3 The spatial distribution of the mosquito genus *Culex* recorded at the county level from 1954 to 2020 in China
20	Supplementary Figure S4 The spatial distribution of the mosquito genus *Anopheles* recorded at the county level from 1954 to 2020 in China
21	Supplementary Figure S5 The spatial distribution of the mosquito genus *Aedes* recorded at the county level from 1954 to 2020 in China
22	Supplementary Figure S6 The spatial distribution of the mosquito genus *Armigeres* recorded at the county level from 1954 to 2020 in China
23	Supplementary Figure S7 The spatial distribution of the mosquito genus *Topomyia* recorded at the county level from 1954 to 2020 in China
24	Supplementary Figure S8 The spatial distribution of the mosquito genus *Uranotaenia* recorded at the county level from 1954 to 2020 in China
25	Supplementary Figure S9 The spatial distribution of the mosquito genus *Heizmannia* recorded at the county level from 1954 to 2020 in China
26	Supplementary Figure S10 The spatial distribution of the other Relatively rare mosquito genus (<10 species) recorded at the county level from 1954 to 2020 in China
27	Supplementary Figure S11 Mosquito species richness (circles) at the prefecture level in seven biogeographic zones in mainland China from 1954 to 2020.
28	Supplementary Table S5. BRT-model-estimated mean (standard deviation) relative contributions of top factors (RC≥5%) to the spatial distribution of three most prevalent mosquito species in the *Anopheles* genus.
30	Supplementary Table S6. BRT-model-estimated mean (standard deviation) relative contributions of top factors (RC≥5%) to the spatial distribution of three most prevalent mosquito species in the *Culex* genus.
32	Supplementary Table S7. BRT-model-estimated mean (standard deviation) relative contributions of top factors (RC≥5%) to the spatial distribution of three most prevalent mosquito species in the *Aedes* and *Armigeres* genus.
33	Supplementary Figure S12 The mean curves (red) and 95% percentiles (gray) for the effects of major predictors (RC≥5%) on the probability of occurrence of *Ar. subalbatus* based on the ensemble of BRT models
34	Supplementary Figure S13 The mean curves (red) and 95% percentiles (gray) for the effects of major predictors (RC≥5%) on the probability of occurrence of *An. sinensis* based on the ensemble of BRT models
35	Supplementary Figure S14 The mean curves (red) and 95% percentiles (gray) for the effects of major predictors (RC≥5%) on the probability of occurrence of *An. anthropophagus* based on the ensemble of BRT models
36	Supplementary Figure S15 The mean curves (red) and 95% percentiles (gray) for the effects of major predictors (RC≥5%) on the probability of occurrence of *An. minimus* based on the ensemble of BRT models
37	Supplementary Figure S16 The mean curves (red) and 95% percentiles (gray) for the effects of major predictors (RC≥5%) on the probability of occurrence of *An. maculatus* based on the

	ensemble of BRT models
**38**	Supplementary Figure S17 The mean curves (red) and 95% percentiles (gray) for the effects of major predictors (RC≥5%) on the probability of occurrence of *An. pattoni* based on the ensemble of BRT models
**39**	Supplementary Figure S18 The mean curves (red) and 95% percentiles (gray) for the effects of major predictors (RC≥5%) on the probability of occurrence of *An. lindesayi* based on the ensemble of BRT models
**40**	Supplementary Figure S19 The mean curves (red) and 95% percentiles (gray) for the effects of major predictors (RC≥5%) on the probability of occurrence of *An. jeyporiensis* based on the ensemble of BRT models
**41**	Supplementary Figure S20 The mean curves (red) and 95% percentiles (gray) for the effects of major predictors (RC≥5%) on the probability of occurrence of *An. tessellatus* based on the ensemble of BRT models
**42**	Supplementary Figure S21 The mean curves (red) and 95% percentiles (gray) for the effects of major predictors (RC≥5%) on the probability of occurrence of *Cx. tritaeniorhynchus* based on the ensemble of BRT models
**43**	Supplementary Figure S22 The mean curves (red) and 95% percentiles (gray) for the effects of major predictors (RC≥5%) on the probability of occurrence of *Cx. pipiens quinquefasciatus* based on the ensemble of BRT models
**44**	Supplementary Figure S23 The mean curves (red) and 95% percentiles (gray) for the effects of major predictors (RC≥5%) on the probability of occurrence of *Cx. pipiens pallens* based on the ensemble of BRT models
**45**	Supplementary Figure S24 The mean curves (red) and 95% percentiles (gray) for the effects of major predictors (RC≥5%) on the probability of occurrence of *Cx. bitaeniorhynchus* based on the ensemble of BRT models
**46**	Supplementary Figure S25 The mean curves (red) and 95% percentiles (gray) for the effects of major predictors (RC≥5%) on the probability of occurrence of *Cx. vagans* based on the ensemble of BRT models
**47**	Supplementary Figure S26 The mean curves (red) and 95% percentiles (gray) for the effects of major predictors (RC≥5%) on the probability of occurrence of *Cx. halifaxia* based on the ensemble of BRT models
**48**	Supplementary Figure S27 The mean curves (red) and 95% percentiles (gray) for the effects of major predictors (RC≥5%) on the probability of occurrence of *Cx. modestus* based on the ensemble of BRT models
**49**	Supplementary Figure S28 The mean curves (red) and 95% percentiles (gray) for the effects of major predictors (RC≥5%) on the probability of occurrence of *Cx. fuscanus* based on the ensemble of BRT models.
**50**	Supplementary Figure S29 The mean curves (red) and 95% percentiles (gray) for the effects of major predictors (RC≥5%) on the probability of occurrence of *Cx. mimeticus* based on the ensemble of BRT models
**51**	Supplementary Figure S30 The mean curves (red) and 95% percentiles (gray) for the effects of major predictors (RC≥5%) on the probability of occurrence of *Cx. pseudovishnui* based on the ensemble of BRT models
**52**	Supplementary Figure S31 The mean curves (red) and 95% percentiles (gray) for the effects of major predictors (RC≥5%) on the probability of occurrence of *Cx. fuscocephala* based on the ensemble of BRT models
**53**	Supplementary Figure S32 The mean curves (red) and 95% percentiles (gray) for the effects of major predictors (RC≥5%) on the probability of occurrence of *Cx. whitmorei* based on the ensemble of BRT models
**54**	Supplementary Figure S33 The mean curves (red) and 95% percentiles (gray) for the effects of major predictors (RC≥5%) on the probability of occurrence of *Cx. mimulus* based on the ensemble of BRT models
**55**	Supplementary Figure S34 The mean curves (red) and 95% percentiles (gray) for the effects of major predictors (RC≥5%) on the probability of occurrence of *Ae. albopictus* based on the ensemble of BRT models
**56**	Supplementary Figure S35 The mean curves (red) and 95% percentiles (gray) for the effects of major predictors (RC≥5%) on the probability of occurrence of *Ae. vexans* based on the ensemble of BRT models
**57**	Supplementary Figure S36 The mean curves (red) and 95% percentiles (gray) for the effects

	of major predictors (RC≥5%) on the probability of occurrence of *Ae. dorsalis* based on the ensemble of BRT models
**58**	Supplementary Figure S37 The mean curves (red) and 95% percentiles (gray) for the effects of major predictors (RC≥5%) on the probability of occurrence of *Ae. aegypti* based on the ensemble of BRT models
**59**	Supplementary Figure S38 The predicted county-level distributions of the one most prevalent mosquito species in the *Ar. subalbatus*, averaged over the ensemble of BRT models
**60**	Supplementary Figure S39 The predicted county-level distributions of the six most prevalent mosquito species in the *Anopheles* genus, averaged over the ensemble of BRT models (A) *An. sinensis,* (B) *An. anthropophagus*, (C) *An. minimus*, (D)*An. maculatus*, (E) *An. pattoni*, (F) *An. lindesayi*, (G) *An. jeyporiensis* and (H) *An. tessellatus*
**61**	Supplementary Figure S40 The predicted county-level distributions of the ten most prevalent mosquito species in the *Culex* genus, averaged over the ensemble of BRT models (A) *Cx. tritaeniorhynchus,* (B) *Cx. pipiens pallens*, (C) *Cx. pipiens quinquefasciatu*, (D) *Cx. Bitaeniorhynchus*, (E) *Cx. Vagans*, (F) *Cx. Halifaxia*, (G) *Cx. Modestus*, (H) *Cx. Fuscanus*, (I) *Cx. mimeticus*, (J) *Cx. pseudovishnui,* (K) *Cx. fuscocephala,* (L) *Cx. whitmorei* and (M) *Cx. mimulus*
**63**	Supplementary Figure S41 The predicted county-level distributions of the ten most prevalent mosquito species in the *Aedes* genus, averaged over the ensemble of BRT models (A) *Ae. albopictus,* (B) *Ae. vexans,* (C) *Ae. dorsalis* and (D) *Ae. aegypti*
**64**	Supplementary Table S8. The mosquito-borne arboviruses included in current study.
**65**	Supplementary Figure S42 The distribution of genotype of Japanese encephalitis virus and serotype of Dengue virus from people, host animals and mosquitoes at the province level in China.
**66**	Supplementary Figure S43 The locations of IgG against mosquito-borne arboviruses detected from humans in during 1954–2020 in China.
**67**	Supplementary Figure S44 The locations of mosquito-borne arboviruses detected in host animals during 1954–2020 in China.
**68**	Supplementary Figure S45 The mean curves (red) and 95% percentiles (gray) for the effects of major predictors (RC≥5%) on the predicted incidence rate of dengue fever based on the ensemble of two stages of GBRT models
**69**	Supplementary Figure S46 The mean curves (red) and 95% percentiles (gray) for the effects of major predictors (RC≥5%) on the predicted incidence rate of Japanese encephalitis based on the ensemble of two stages of GBRT models
**70**	Supplementary References

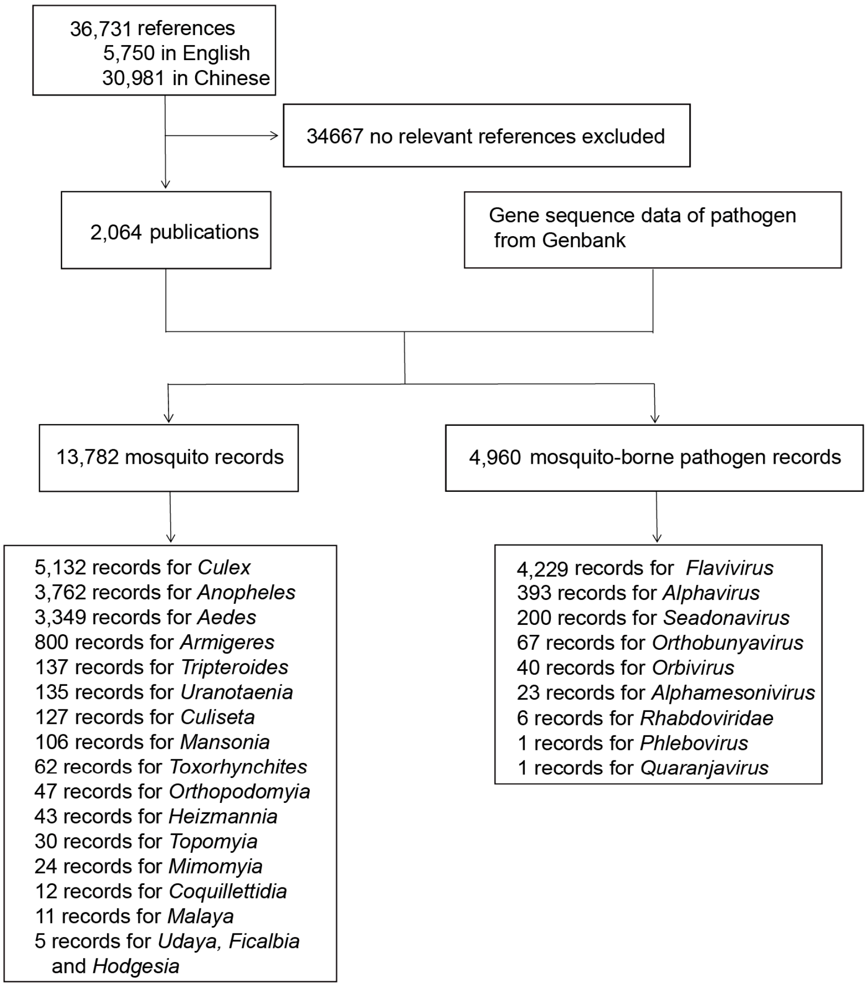

Supplementary Figure S1 The flow diagram of literature review.

Supplementary Table S1. The specific references for all 339 mosquito species in China from 1954 to 2020

Mosquito species	Reference
Anopheles	2, 5-7, 9, 11, 14, 15, 17-21, 23, 25-27, 37, 38, 42, 43, 45, 47, 49, 51-53, 57, 58, 66, 68, 71-75, 77-79, 82, 86, 91-93, 96-98, 100, 101, 103, 105-108, 112, 113, 115-117, 119-122, 124-127, 136-139, 141-145, 147, 149, 151, 153-155, 159, 161, 163, 165, 166, 168, 171, 180, 182-184, 187-189, 193-195, 200-202, 209, 211, 213-215, 218, 219, 221-225, 227, 236, 237, 240, 242, 244-249, 256, 258-263, 265, 268-270, 272, 275-277, 279, 283, 285, 287, 289-292, 301, 310-313, 315, 316, 321, 322, 324, 325, 329, 334, 335, 337, 339-342, 344-348, 350-353, 358, 359, 364, 365, 368, 371-373, 377, 378, 380-382, 385-390, 393, 394, 396, 397, 401-403, 408, 410, 412, 413, 415-418, 420-422, 426-428, 432, 434-439, 441-444, 446-451, 453-456, 459, 461, 462, 464, 466-476, 480, 484, 486, 490-499, 501-503, 505-508, 510, 512, 514, 518, 520-522, 525-528, 530, 531, 533, 534, 536-539, 541, 543-547, 549-552, 554-558, 560-565, 570-572, 574, 576, 578-581, 583-588, 592-595, 598-603, 605, 607, 608, 614-616, 621, 623, 626, 628-631, 634-638, 641-646, 652, 654-656, 658, 659, 663, 665, 666, 668-671, 676, 677, 680, 682-684, 689, 692, 693, 695, 697, 701, 702, 704-708, 710, 712, 715, 717, 718, 720, 723, 726-728, 730-737, 739, 740, 743, 745, 746, 748, 754-757, 761-767, 769, 771-774, 776, 778, 780-782, 786, 787, 789, 792, 793, 795-797, 799-805, 807, 809-813, 815-821, 824, 826, 827, 829, 831-833, 835-837, 839, 840, 843-846, 849, 851, 853, 856, 857, 859, 860, 863-865, 872, 875, 877-880, 882-885, 888, 890, 892, 894, 895, 899, 901, 902, 904, 905, 907, 911-915, 919, 921
An. sinensis	2, 5-7, 9, 11, 14, 17-21, 23, 27, 37, 38, 42, 43, 45, 49, 51-53, 57, 58, 66, 68, 71-75, 77-79, 91, 92, 96-98, 100, 101, 103, 105, 106, 108, 112, 113, 115-117, 119-121, 125-127, 136-139, 142-145, 151, 153-155, 159, 163, 165, 166, 168, 180, 182-184, 188, 193-195, 200, 201, 213-215, 218, 222, 224, 225, 227, 242, 244-249, 256, 258, 260-263, 265, 268-270, 272, 275-277, 279, 285, 287, 289, 291, 292, 311, 312, 315, 316, 321, 322, 325, 329, 334, 335, 337, 339, 341, 342, 344, 346-348, 350, 352, 353, 358, 359, 364, 365, 368, 372, 373, 377, 378, 380-382, 385-390, 393, 394, 396, 401-403, 408, 410, 412, 413, 415-418, 420, 422, 426, 428, 432, 434-439, 441-444, 446, 447, 449, 450, 453-456, 461, 462, 464, 466-473, 475, 476, 480, 484, 486, 490-496, 498, 499, 501-503, 505-508, 510, 512, 514, 518, 520, 522, 525-528, 530, 533, 534, 537, 541, 543-547, 549-552, 554-558, 560-562, 564, 565, 570-572, 574, 576, 578-581, 583, 585-587, 592-595, 598-600, 602, 603, 605, 607, 614-616, 621, 623, 628-631, 634-638, 641-646, 652, 654, 659, 663, 665, 666, 668-671, 676, 677, 680, 682-684, 689, 692, 693, 695, 697, 701, 702, 704-708, 710, 712, 715, 717, 718, 720, 723, 726-728, 730-737, 739, 740, 743, 745, 748, 754-757, 761-767, 769, 771-774, 776, 778, 780-782, 786, 789, 792, 793, 796, 799-805, 807, 809-813, 815-819, 821, 824, 826, 827, 829, 831-833, 835-837, 840, 843, 845, 849, 851, 853, 857, 859, 860, 863-865, 877-880, 882-885, 888, 892, 894, 895, 899, 901, 904, 905, 907, 911-915, 919, 921
An. anthropophagus	19, 20, 26, 38, 66, 79, 103, 107, 159, 161, 166, 183, 187-189, 202, 211, 213, 218, 221, 245, 256, 259, 265, 269, 270, 290-292, 301, 310, 311, 313, 322, 325, 337, 359, 365, 371, 380, 387, 394, 397, 415-417, 421, 441-443, 448, 451, 453, 459, 464, 474, 521, 531, 534, 536, 551, 599, 637, 638, 643, 704, 739, 766, 782, 815, 832, 915
An. minimus	7, 19, 25, 38, 45, 52, 79, 97, 98, 106, 137, 154, 166, 193, 219, 225, 236, 237, 240, 245, 256, 258, 265, 285, 291, 292, 325, 334, 337, 347, 358, 386, 394, 410, 412, 417, 426, 432, 441, 444, 466, 480, 490, 502, 506, 512, 520, 526, 527, 539, 551, 556, 563, 565, 578, 583, 584, 599, 616, 623, 656, 666, 670, 680, 723, 743, 769, 772, 797, 799, 819, 827, 837, 839, 857, 872, 880, 894, 895, 907, 911, 915
An. maculatus	19, 52, 57, 77, 97, 101, 106, 137, 149, 154, 195, 225, 265, 325, 334, 335, 386, 402, 410, 417, 418, 426, 432, 441, 444, 455, 466, 480, 490, 502, 506, 512, 520, 527, 534, 578, 579, 598, 599, 616, 623, 666, 723, 746, 769, 772, 787, 800, 819, 820, 827, 833, 872, 875, 880, 894, 904, 907, 911, 914
An. pattoni	11, 14, 53, 73, 141, 265, 312, 325, 329, 335, 339, 358, 377, 393, 410, 413, 441, 484, 496, 497, 505, 599, 601, 645, 697, 795
An. lindesayi	17, 52, 149, 265, 324, 325, 329, 335, 358, 371, 377, 413, 416-418, 426, 441, 480, 505, 506, 520, 527, 534, 579, 599, 697, 723, 755, 880
An. jeyporiensis	52, 97, 137, 154, 166, 265, 291, 292, 325, 334, 347, 386, 388, 402, 412, 417, 418, 426, 432, 444, 480, 502, 506, 512, 520, 526, 527, 551, 578, 586, 598, 599, 616, 666, 680, 769, 880
An. tessellatus	19, 20, 42, 52, 57, 77, 97, 101, 103, 106, 124, 137, 145, 154, 155, 195, 225, 244, 325, 334, 337, 344, 347, 386, 388, 410, 412, 417, 418, 426, 432, 441, 446, 480, 506, 512, 520, 527, 530, 578, 586, 598, 608, 616, 623, 630, 704, 723, 769, 772, 800, 813, 872, 880, 894, 904, 907
An. splendidus	19, 20, 45, 52, 57, 77, 101, 106, 137, 154, 225, 325, 347, 386, 410, 412, 417, 426, 441, 480, 490, 502, 506, 512, 520, 527, 578, 586, 623, 666, 723, 769, 772, 872, 880, 907
An. fluviatilis	52, 124, 154, 225, 265, 325, 347, 412, 417, 432, 441, 480, 502, 506, 512, 527, 599, 819
An. kweiyangensis	52, 265, 325, 368, 371, 417, 418, 441, 442, 466, 471, 599, 755
An. annularis	106, 325, 347, 417, 426, 441, 480, 502, 506, 520, 527, 578, 586, 598, 599, 616, 666, 680, 769, 800, 813, 872, 880, 904, 907
An. vagus	7, 19, 42, 45, 97, 100, 106, 121, 137, 142, 144, 154, 195, 334, 347, 386, 410, 426, 446, 455, 480, 502, 506, 520, 527, 530, 541, 557, 565, 578, 586, 598, 616, 623, 626, 666, 723, 769, 772, 800-803, 805, 813, 819, 880, 894, 904, 907
An. gigas baileyi	225, 265, 324, 325, 386, 441, 480, 506, 527, 534, 538, 579, 599, 723, 833, 872, 880
An. yatsushiroensis	2, 19, 141, 149, 159, 218, 325, 329, 371, 377, 387, 428, 441, 442, 464, 505, 506, 588, 636, 638
An. nigerrimus	325, 527, 534, 890
An. hyrcanus	15, 106, 122, 137, 171, 209, 334, 340, 351, 386, 480, 697, 846, 856, 864, 880, 894, 902
An. peditaeniatus	19, 45, 106, 137, 195, 334, 386, 410, 417, 441, 442, 446, 453, 490, 506, 534, 578, 586, 623, 723, 772, 787, 813, 872, 875, 880, 894, 901, 904
An. culicifacies	45, 97, 225, 325, 359, 410, 441, 490, 502, 506, 578, 586, 598, 666, 723, 769, 772, 837, 872, 880, 907
An. messeae	17, 122, 209, 329, 340, 351, 393, 514, 534, 636, 856
An. bengalensis	325, 416-418, 441, 480, 527, 534, 755
An. barbirostris	73, 106, 124, 154, 325, 334, 337, 386, 410, 506, 527, 586, 599, 623, 626, 655, 656, 704, 772, 800, 801, 813, 872, 894, 904, 907
An. kochi	19, 20, 347, 410, 432, 506, 520, 527, 623, 626, 666, 772, 800, 819, 872, 907
An. philippinensis	137, 154, 325, 334, 347, 386, 432, 506, 520, 526, 527, 598, 626, 801, 802, 805, 819
An. stephensi	225, 334, 345, 410, 417, 426, 441, 520, 527, 534, 769
An. sacharovi	15, 171, 736

An. sineroides	14, 17, 19, 329, 505, 669
An. aconitus	42, 47, 137, 154, 334, 347, 386, 506, 527, 541, 586, 598, 623, 772, 800, 872
An. subpictus	154, 225, 263, 402, 417, 446, 502, 506, 527, 586, 769
An. varuna	334, 417, 502, 506, 769
An. pseudowillmori	410, 534, 723, 833, 875, 907, 911
An. barbumbrosus	334, 337, 441, 480, 506, 586, 772, 911
An. kunmingensis	82, 225, 359, 441, 467, 506, 723, 800
An. aitkenii	325, 417, 441, 599
An. nitidus	417, 441, 442
An. dirus	25, 93, 147, 223, 256, 334, 386, 844
An. crawfordi	137, 386, 599, 872, 880
An. gigas simlensis	324, 325, 534, 880
An. claviger	15, 171, 902
An. willmori	534, 833, 911
An. indefinitus	73, 141, 149, 347, 402
An. karwari	154, 347, 527
An. argyropus	490, 534, 772
An. freyi	325, 441
An. xui	410
An. koreicus	17
An. interruptus	534
An. liangshanensis	387
An. ramsayi	86
An. hailarensis	427
An. sintonoides	658
An. menglangensis	283
Culex	1, 2, 4-7, 9, 11-18, 20, 21, 27-34, 36, 38, 42-45, 47-54, 56, 57, 64, 65, 67, 68, 71-73, 75, 77-79, 84, 85, 91, 96, 100-106, 109, 111, 112, 115-117, 119-122, 124, 126, 127, 135-146, 150, 153-155, 160, 163, 165, 167-172, 174-176, 180-182, 184, 190, 193, 195, 198-201, 203, 206, 209, 213, 214, 216, 220, 222, 224, 225, 227, 228, 230, 232, 233, 236, 237, 241-246, 249, 251, 253, 255, 256, 262-265, 268, 270, 272, 274, 277, 279, 280, 285, 286, 289, 292, 295, 300, 305, 306, 308, 309, 312, 315, 319, 323-327, 329, 334-336, 338-342, 344-348, 351-355, 358, 359, 364, 365, 368, 370, 372, 376-378, 382, 383, 385, 387-389, 393-395, 398, 399, 401-404, 406-408, 410, 411, 413-420, 422-424, 428, 430-436, 438, 440, 441, 447, 449, 450, 452-454, 456, 457, 462, 463, 465-467, 469-473, 475, 476, 480-482, 485, 487-489, 491-501, 503-510, 513-517, 519, 522, 525-527, 529, 533-535, 541-547, 550, 552-555, 557-562, 564-566, 568, 569, 571-581, 585, 587, 588, 591-594, 596, 597, 599-605, 607, 608, 610, 613-616, 618, 620, 622-624, 626-629, 631, 633, 634, 636, 638, 641-650, 652, 655, 656, 658-662, 664, 665, 668-671, 673-679, 681-687, 689, 690, 692, 693, 695-697, 699-702, 704-706, 708, 710, 712, 713, 715, 717-720, 725-729, 733-741, 743-745, 747, 748, 750-752, 754-757, 760-767, 769-771, 773-776, 778-781, 783, 784, 786-793, 795, 796, 798, 800-814, 816-819, 821, 823, 824, 826, 828, 831, 833, 835, 840, 842, 843, 845, 847-861, 863-868, 870-878, 880-890, 892, 894-896, 898-907, 910-917, 919, 920, 922
Cx. tritaeniorhynchus	2, 4-7, 9, 11, 14, 16-18, 20, 21, 27, 38, 42, 43, 47, 49, 51-54, 57, 64, 65, 68, 71-73, 75, 77-79, 91, 96, 100, 101, 103, 105, 106, 111, 112, 115-117, 119-121, 124, 126, 127, 136-139, 142, 143, 145, 154, 155, 163, 165, 168, 169, 175, 180, 184, 193, 195, 199, 201, 206, 213, 214, 216, 220, 225, 227, 230, 244-246, 249, 255, 256, 263, 265, 268, 270, 272, 277, 280, 285, 286, 289, 292, 300, 305, 306, 312, 315, 325, 326, 329, 334, 335, 338, 339, 341, 342, 344, 346-348, 352-354, 358, 359, 364, 365, 368, 372, 378, 382, 383, 387-389, 393-395, 401-403, 408, 410, 411, 413, 416-418, 420, 422, 428, 430, 432, 434-436, 438, 441, 447, 449, 450, 453, 454, 456, 457, 462, 465-467, 470-473, 475, 476, 480, 481, 489, 491-499, 501, 503, 505-508, 510, 513, 514, 516, 517, 522, 526, 527, 533-535, 541-547, 552-555, 557, 558, 560, 564-566, 568, 569, 571-574, 576, 578-581, 585, 587, 588, 591-594, 596, 597, 599-605, 607, 608, 613-615, 618, 620, 623, 626, 628, 629, 631, 633, 634, 638, 641-643, 645, 646, 650, 655, 659, 662, 665, 668-671, 673, 676-678, 681-687, 689, 692, 693, 695, 697, 699-702, 704, 705, 708, 710, 712, 715, 717-720, 726, 727, 729, 733, 735-740, 743, 745, 748, 751, 752, 754-757, 762-766, 769, 771, 773, 774, 776, 778-781, 783, 784, 786, 792, 793, 795, 796, 798, 800-805, 807, 809, 811-813, 816-819, 821, 823, 824, 826, 828, 831, 833, 835, 840, 843, 845, 849, 851, 852, 857-861, 863-868, 870-872, 874, 875, 877, 878, 880-888, 892, 894-896, 898, 899, 901, 903-905, 907, 911-917, 919, 920, 922
Cx. pipiens quinquefasciatus	1, 5, 7, 18, 20, 21, 38, 42, 43, 48, 51, 52, 54, 56, 57, 71, 75, 77-79, 91, 100, 103, 106, 109, 115, 119, 120, 127, 137-139, 142, 144, 145, 153-155, 165, 167, 169, 170, 190, 193, 195, 199, 203, 206, 213, 214, 216, 225, 230, 232, 233, 236, 237, 242, 244-246, 249, 263-265, 268, 270, 285, 289, 292, 300, 305, 309, 315, 323-325, 334, 335, 341, 342, 344, 347, 348, 355, 358, 359, 365, 368, 370, 372, 387, 388, 394, 402-404, 410, 414, 415, 417, 418, 430-432, 435, 436, 441, 449, 450, 452, 453, 456, 466, 467, 469-471, 480, 489, 494, 495, 506-508, 515, 519, 526, 527, 533-535, 541, 546, 547, 557, 559, 561, 562, 565, 578, 579, 587, 591, 592, 596, 599, 603, 607, 613, 620, 623, 626, 627, 631, 633, 634, 638, 642-644, 646, 649, 650, 656, 661, 664, 668, 676, 687, 690, 697, 702, 704-706, 715, 718, 736, 739-741, 747, 751, 754-756, 761, 764, 765, 769, 770, 776, 778, 780, 786, 791, 793, 800-803, 807, 808, 811, 813, 814, 817, 819, 821, 824, 828, 833, 840, 843, 845, 847, 853, 855, 872, 875, 880, 885, 886, 894, 896, 900, 901, 903, 906, 907, 910, 911, 915, 916, 919
Cx. pipiens pallens	2, 4, 6, 9, 11-14, 16-18, 27, 43, 44, 49-51, 53, 64, 65, 67, 68, 71-73, 78, 96, 112, 116, 117, 126, 136, 140, 141, 143, 146, 150, 163, 168, 171, 172, 174-176, 180-182, 184, 200, 201, 222, 224, 227, 228, 243, 246, 253, 255, 256, 262, 265, 270, 272, 274, 277, 279, 295, 308, 312, 319, 327, 329, 335, 339, 341, 346, 352, 353, 358, 364, 370, 377, 378, 382, 383, 385, 389, 393, 395, 398, 399, 401, 403, 406, 411, 413, 420, 422-424, 428, 433, 434, 447, 454, 465, 472, 473, 475, 476, 482, 485, 491-493, 496-501, 503-505, 507, 509, 510, 513, 514, 517, 522, 525, 529, 533, 534, 543-545, 550, 552-555, 558, 560, 564, 568, 569, 574-577, 581, 585, 588, 594, 597, 602, 604, 605, 608, 610, 614, 616, 622, 624, 628, 629, 636, 641, 645, 648, 649, 652, 659, 660, 662, 665, 669-671, 673-675, 677, 678, 682, 684, 686, 689, 692, 693, 695-697, 700, 701, 706, 708, 710, 712, 713, 717, 719, 725-729, 733-738, 740, 743, 745, 748, 750, 752, 757, 762-767, 771, 773, 774, 780, 781, 790, 792, 795, 796, 798,

	806, 808, 810-812, 816-818, 826, 831, 835, 849-852, 854, 859-861, 863, 865, 870, 874-878, 882, 888-890, 899-901, 905, 912-914
Cx. bitaeniorhynchus	7, 9, 14, 17, 38, 51, 52, 54, 57, 75, 77, 78, 96, 103, 106, 112, 124, 137, 142, 144, 145, 154, 155, 163, 168, 175, 180, 193, 206, 213, 225, 227, 246, 249, 263, 265, 280, 289, 315, 325, 329, 334, 335, 339, 344, 347, 358, 364, 377, 387, 388, 393, 402, 410, 413, 416-418, 420, 422, 441, 480, 495, 496, 499, 505-507, 522, 527, 534, 541, 557, 565, 578, 579, 599, 605, 623, 626, 631, 634, 638, 646, 655, 661, 662, 669, 689, 692, 695, 697, 704, 705, 720, 733, 755, 756, 769, 795, 798, 800, 801, 803, 811, 813, 819, 826, 833, 845, 863, 864, 872, 875, 880, 894, 895, 904, 912
Cx. vagans	2, 14, 15, 17, 38, 51, 52, 54, 73, 77, 78, 96, 115, 124, 141, 168, 175, 206, 227, 265, 319, 324, 325, 329, 334, 335, 347, 353, 358, 364, 376, 393, 403, 413, 416-418, 441, 453, 467, 480, 496, 505, 514, 522, 527, 534, 535, 553, 599, 628, 634, 636, 669, 678, 682, 692, 697, 720, 736, 770, 775, 811, 902
Cx. halifaxia	14, 20, 38, 51, 52, 54, 57, 77, 78, 96, 101, 106, 109, 137, 141, 160, 168, 203, 225, 227, 251, 265, 285, 325, 329, 334, 335, 358, 364, 368, 388, 402, 410, 413, 416-419, 432, 441, 471, 480, 495-497, 505, 506, 513, 522, 526, 527, 534, 579, 592, 599, 613, 658, 689, 697, 704, 736, 740, 755, 833, 872, 880, 907, 911
Cx. modestus	2, 14, 15, 17, 20, 122, 124, 171, 175, 176, 209, 319, 329, 335, 340, 351, 353, 364, 393, 398, 413, 428, 463, 505, 514, 534, 628, 636, 647, 669, 686, 692, 697, 733, 736, 744, 789, 796, 849, 852, 854, 856, 859, 890, 900, 902, 905
Cx. fuscanus	43, 52, 57, 75, 77, 78, 100-103, 106, 124, 137, 145, 153-155, 180, 199, 206, 213, 225, 244, 265, 272, 285, 325, 334, 335, 344, 347, 364, 410, 413, 417, 418, 422, 428, 441, 480, 506, 527, 533, 534, 579, 599, 613, 642, 678, 697, 704, 710, 736, 761, 769, 776, 780, 813, 833, 848, 853, 872, 880, 886, 894, 904, 911, 919
Cx. mimeticus	14, 17, 38, 52, 57, 101, 137, 206, 265, 324, 325, 329, 334, 358, 359, 364, 377, 389, 413, 416-418, 441, 466, 480, 506, 527, 534, 592, 599, 697, 755, 813, 821, 833, 872
Cx. pseudovishnui	38, 52, 75, 77, 78, 96, 106, 127, 137, 225, 263, 265, 280, 285, 325, 334, 347, 368, 388, 407, 410, 417, 418, 441, 466, 471, 480, 495, 506, 527, 534, 557, 572, 580, 599, 613, 626, 683, 697, 787, 800-804, 819, 833, 872, 880, 904
Cx. fuscocephala	20, 38, 47, 57, 77, 101, 103, 106, 137, 145, 154, 155, 180, 195, 225, 265, 280, 325, 334, 347, 388, 402, 410, 417, 418, 432, 441, 480, 506, 526, 527, 534, 541, 557, 571, 578, 580, 599, 623, 626, 683, 697, 702, 736, 769, 787, 800-804, 813, 819, 864, 872, 880, 894, 904, 907
Cx. whitmorei	14, 52, 77, 96, 106, 137, 154, 168, 213, 225, 251, 263, 265, 325, 329, 334, 347, 368, 389, 402, 410, 413, 417, 418, 432, 441, 466, 471, 480, 495, 496, 505, 506, 526, 527, 534, 557, 580, 599, 626, 662, 683, 800-804, 819, 833, 894, 904
Cx. mimulus	43, 77, 78, 206, 244, 265, 272, 325, 334, 335, 358, 364, 368, 416-418, 432, 441, 480, 506, 527, 534, 613, 697, 740, 848
Cx. pallidothorax	52, 57, 77, 106, 168, 175, 203, 227, 325, 334, 364, 368, 388, 389, 398, 402, 410, 416-419, 432, 441, 466, 471, 480, 506, 527, 571, 579, 599, 613, 623, 658, 697, 704, 740, 755, 872, 880, 886, 894, 904
Cx. theileri	127, 135, 225, 263, 265, 280, 325, 345, 359, 417, 441, 480, 506, 527, 534, 557, 599, 702, 769, 800, 802, 872, 911
Cx. jacksoni	17, 52, 225, 265, 325, 329, 335, 345, 358, 413, 417, 418, 441, 480, 496, 505, 506, 514, 599, 669, 697, 872
Cx. sinensis	77, 137, 225, 263, 265, 300, 325, 364, 413, 417, 418, 432, 466, 480, 526, 527, 535, 541, 599, 626, 697, 800, 802, 803, 854, 872
Cx. shebbearei	52, 160, 203, 206, 265, 324, 325, 358, 368, 416-419, 441, 466, 471, 480, 495, 527, 534, 599, 678, 755, 775, 886
Cx. malayi	38, 75, 77, 78, 96, 251, 265, 325, 358, 364, 389, 417, 441, 466, 527, 599, 697, 788
Cx. hayashii	14, 52, 57, 175, 265, 325, 329, 358, 368, 398, 413, 416-418, 441, 480, 496, 505, 514, 599, 697, 755
Cx. annulus	52, 57, 106, 137, 206, 225, 263, 280, 334, 359, 402, 410, 417, 441, 453, 557, 571, 579, 580, 599, 623, 626, 704, 755, 769, 788, 800-804, 813, 819, 833, 872, 880
Cx. pipiens	15, 122, 209, 340, 351, 416, 534, 649, 870, 901, 902
Cx. orientalis	2, 14, 17, 265, 329, 364, 466, 505, 514, 527, 534, 553, 669
Cx. murrelli	52, 57, 198, 334, 345, 358, 368, 416-418, 441, 471, 506, 599, 755, 833
Cx. huangae	325, 534, 599, 679
Cx. nigropunctatus	137, 225, 334, 359, 410, 441, 466, 480, 527, 534, 623, 626, 800, 813, 819, 872, 880, 894, 904, 907
Cx. gelidus	103, 106, 137, 265, 334, 359, 417, 418, 506, 526, 527, 557, 571, 599, 623, 626, 769, 800-803, 813, 872, 880, 904
Cx. brevipalpis	325, 402, 417-419, 527, 599, 658, 697, 736
Cx. foliatus	57, 225, 265, 325, 334, 417, 418, 480, 755
Cx. infantulus	38, 52, 141, 265, 325, 417, 418, 441, 506, 599, 697, 755
Cx. territens	2, 15, 329, 636, 697
Cx. sitiens	54, 57, 101, 154, 155, 334, 413, 417, 420
Cx. minor	417, 418, 506, 527, 755
Cx. spiculosus	30, 417, 506, 613, 755
Cx. richei	368, 417
Cx. barraudi	206, 334, 345, 453, 788
Cx. Eumelano-myia	52, 416, 418, 755, 842
Cx. bailyi	30, 31, 599, 872
Cx. kyotoensis	417, 418, 599
Cx. guizhouensis	198, 417, 599
Cx. rubensis	241, 505
Cx. fuscifurcatus	413, 527
Cx. rubithoracis	20, 78, 265, 417, 480

Cx. harrisoni	137, 206, 417, 488
Cx. tenuipalpis	52, 368, 417, 418, 471, 506, 527
Cx. bicornutus	54, 658, 920
Cx. pipiens molestus	206, 364, 808
Cx. megafolius	36, 873
Cx. tianpienensis	29, 52, 198, 417
Cx. bengalensis	45, 206
Cx. clarkii	623, 769
Cx. torrentium	31, 329, 886
Cx. wilfedi	34, 886
Cx. szemaoensis	109, 336
Cx. uniformis	863, 872
Cx. dispectus	84
Cx. macdonaldi	30, 334
Cx. mammilifer	30, 334
Cx. peytoni	30
Cx. scanloni	30, 334
Cx. mimutissimus	480, 527
An. inatomii	104
Cx. fuscocephalus	813
Cx. hutchinsoni	599
Cx. thurmanorum	31
Cx. fragilis	34
Cx. perplexus	34
Cx. soiculostylus	33
Cx. oresbius	85
Cx. megaonychus	440
Cx. hortensis	760
Cx. hainanensis	28, 658
Cx. chungkiangensis	487
Cx. miaolinggensis	32
Cx. sumatranus	154
Cx. okinawae	534
Aedes	2-8, 10, 14-17, 20-22, 24, 27, 38-44, 46, 49, 51, 52, 54-57, 59-63, 65, 68-71, 73, 75-80, 91, 94-96, 99-101, 103, 106, 109, 110, 112, 116-118, 121-124, 126, 127, 130, 135, 137-146, 148, 153-157, 160, 162, 164, 165, 167, 169-171, 173, 175-180, 182, 184-186, 190-193, 195-201, 203-210, 212-214, 216, 217, 222, 224, 225, 227, 229-231, 233-239, 242-246, 249-252, 254-257, 262, 263, 265-267, 272, 273, 279, 281, 282, 284, 285, 288, 293, 294, 296-300, 302-305, 312, 314, 315, 317-320, 324, 325, 328, 329, 331-335, 339-345, 347-349, 351-353, 356-358, 360-370, 372, 374, 375, 377-379, 382, 383, 385, 387-389, 391-395, 398, 400-405, 408-410, 413, 416-420, 422, 425, 428-433, 435, 441, 445, 450, 452-454, 456, 458, 460, 466, 467, 469-473, 475, 477-480, 483, 485, 487, 491-493, 495-499, 501, 503, 505-511, 513, 514, 522-527, 529, 532, 534, 535, 540, 541, 543, 544, 546-548, 552-555, 557, 558, 560-562, 564, 565, 567-569, 574, 576, 578-582, 585, 588-592, 594, 596, 599, 603, 605-613, 617, 620, 623, 625-629, 631-634, 636, 638-643, 645, 650-653, 655, 656, 658, 659, 661, 662, 664, 665, 667-669, 671-675, 677, 682-684, 686, 688-693, 695, 697, 698, 700, 701, 703-705, 708-712, 714, 716, 721, 724, 725, 729, 733-737, 739, 741-743, 747, 749-751, 753, 755, 757-759, 761, 766-770, 773-778, 780, 781, 785-788, 790, 792-796, 798, 800-804, 806-808, 810, 812, 813, 818, 819, 821, 822, 824, 825, 828, 831, 833, 834, 838, 840, 841, 843, 845, 847, 848, 852, 854, 856, 857, 859, 862-865, 869, 870, 872-878, 880, 886, 888, 890, 891, 893, 894, 896, 897, 899, 901-903, 905-912, 914-916, 918, 920
Ae. albopictus	4-7, 10, 14, 16, 20-22, 24, 27, 38-44, 46, 49, 52, 54-57, 60-63, 65, 68, 70, 71, 73, 75-80, 91, 94, 96, 99-101, 103, 106, 110, 112, 116-118, 121, 123, 124, 126, 138-146, 148, 153-157, 160, 164, 165, 167, 169, 170, 173, 177-180, 184-186, 191, 193, 195, 199-201, 203, 204, 206, 207, 210, 212-214, 216, 217, 222, 225, 227, 229-231, 233-237, 239, 242-246, 249-251, 254-257, 265-267, 272, 273, 279, 281, 282, 284, 285, 288, 293, 294, 296-300, 302-305, 312, 314, 315, 317, 318, 325, 329, 334, 335, 339, 341-344, 347-349, 356, 360-366, 368-370, 372, 374, 375, 378, 379, 382, 383, 385, 387, 388, 391, 392, 394, 395, 400-405, 408-410, 413, 416-419, 422, 429-433, 435, 441, 445, 450, 452-454, 456, 460, 466, 469-473, 475, 477-479, 483, 485, 491-493, 495-499, 501, 503, 506-511, 513, 523-527, 529, 532, 534, 540, 541, 543, 544, 546-548, 552, 554, 555, 557, 558, 560-562, 564, 565, 567-569, 574, 576, 580, 581, 585, 589-592, 594, 596, 599, 603, 605-607, 609-613, 617, 620, 623, 625-627, 629, 631-634, 638-643, 645, 650-653, 655, 656, 658, 659, 661, 664, 665, 667, 668, 671, 672, 674, 675, 677, 682-684, 688-692, 695, 698, 700, 701, 703-705, 708-710, 712, 714, 716, 724, 725, 729, 733-737, 739, 741-743, 747, 749-751, 753, 757-759, 761, 766-770, 773, 774, 776-778, 780, 781, 786, 788, 792-796, 800, 801, 803, 804, 806-808, 810, 812, 813, 818, 819, 821, 824, 825, 828, 831, 833, 834, 838, 840, 841, 843, 845, 847, 848, 852, 857, 859, 862, 863, 865, 869, 870, 872, 874, 875, 877, 878, 880, 886, 891, 894, 896, 903, 906, 908-910, 912, 914-916, 918, 920
Ae. vexans	2, 14, 15, 17, 20, 52, 54, 73, 96, 103, 106, 122, 127, 145, 171, 175, 176, 180, 182, 195, 209, 224, 225, 227, 236, 237, 251, 262, 263, 265, 319, 320, 324, 325, 329, 335, 340, 351-353, 358, 364, 377, 383, 393, 398, 403, 410, 413, 417, 422, 425, 441,

	467, 478, 480, 496-498, 505-507, 513, 514, 525-527, 534, 535, 553, 557, 578, 599, 623, 626, 628, 636, 641, 655, 662, 669, 688, 695, 697, 721, 733, 736, 769, 787, 790, 796, 798, 800-804, 810, 812, 813, 819, 833, 845, 854, 856, 859, 863, 864, 876, 880, 888, 890, 894, 899, 901, 902, 905, 907, 914
Ae. dorsalis	14, 15, 49, 124, 175, 176, 209, 319, 320, 329, 340, 351-353, 364, 377, 389, 393, 398, 413, 428, 472, 498, 505, 507, 525, 552, 628, 636, 669, 671, 673, 686, 693, 697, 735, 785, 790, 796, 852, 854, 856, 859, 876, 888, 890, 897, 899, 901, 905, 914
Ae. elsiae	52, 57, 101, 109, 225, 265, 325, 358, 368, 416-418, 441, 471, 506, 534, 579, 599, 613, 634, 755, 833, 872, 880, 911
Ae. japonicus	52, 160, 265, 325, 329, 334, 335, 358, 368, 416-419, 441, 480, 495, 514, 527, 599, 669, 755, 775
Ae. albolateralis	77, 80, 106, 137, 195, 225, 251, 263, 265, 325, 410, 417, 432, 441, 466, 480, 506, 526, 527, 534, 578, 580, 599, 655, 658, 683, 704, 803, 833, 848, 880, 911, 920
Ae. pseudalbopictus	52, 57, 95, 101, 109, 160, 203, 251, 325, 334, 368, 416-419, 429, 441, 471, 527, 534, 557, 580, 599, 626, 655, 683, 755, 800, 801, 803, 819, 833, 848
Ae. caspius	3, 15, 69, 122, 171, 175, 176, 209, 224, 319, 320, 340, 351, 398, 588, 697, 711, 785, 790, 854, 856, 890, 901, 902
Ae. hatorii	14, 265, 325, 329, 334, 335, 345, 358, 398, 416-418, 441, 505, 599, 697
Ae. koreicus	14, 17, 51, 141, 175, 265, 272, 325, 329, 353, 413, 420, 441, 496, 497, 505, 522, 553, 697
Ae. annandalei	20, 57, 80, 137, 195, 203, 210, 265, 410, 441, 445, 506, 527, 534, 557, 580, 599, 608, 613, 626, 634, 655, 683, 704, 769, 800, 801, 803, 819, 833, 848, 857, 872, 920
Ae. chemulpoensis	14, 17, 51, 265, 325, 329, 335, 339, 353, 364, 413, 497, 505, 513, 697
Ae. craggi	52, 57, 80, 101, 160, 325, 368, 410, 417-419, 441, 471, 599, 655, 658, 755, 788, 833, 848, 920
Ae. aegypti	8, 59, 60, 80, 154, 155, 162, 185, 190-192, 205, 210, 236-239, 245, 256, 317, 347, 349, 357, 367, 392, 417, 435, 445, 523, 541, 611-613, 625, 640, 650, 656, 688, 742, 759, 822, 825, 838, 893
Ae. excrucians	2, 17, 73, 141, 175, 176, 319, 328, 329, 393, 398, 514, 636
Ae. flavescens	2, 15, 17, 73, 141, 145, 209, 329, 393, 636, 686
Ae. punctor	2, 17, 325, 329, 393, 505, 636, 669
Ae. fengi	52, 160, 203, 265, 325, 368, 416-419, 441, 471, 599, 755, 775
Ae. togoi	14, 57, 101, 138, 329, 342, 343, 413, 417, 420, 496, 505, 697, 780, 828, 840, 915
Ae. harveyi	20, 57, 135, 160, 325, 345, 416-419, 506, 527, 534, 658, 755, 833, 848
Ae. cinereus	2, 17, 328, 329, 393, 636
Ae. lineatopennis	17, 20, 106, 225, 265, 325, 329, 417, 478, 506, 527, 534, 557, 580, 623, 626, 697, 800, 802-804, 813, 848, 872
Ae. macfarianei	52, 57, 95, 101, 198, 334, 368, 398, 416-418, 471, 527, 534, 697, 886
Ae. cataphylla	2, 17, 73, 175, 176, 329, 393, 636, 669
Ae. assamensis	95, 109, 325, 345, 458, 478, 506, 527, 534, 557, 580, 658, 683, 704, 800, 803
Ae. diantaeus	2, 17, 328, 329, 636, 669
Ae. communis	2, 17, 325, 329, 393, 636
Ae. flavidorsalis	15, 69, 175, 176, 319, 320, 329, 393, 398, 522, 697, 711, 785, 854, 890, 901
Ae. pullatus	2, 15, 175, 176, 329, 393, 636, 669, 697, 890
Ae. cyprius	2, 15, 73, 328, 329, 393, 636
Ae. formosensis	57, 101, 109, 225, 265, 417, 480, 506, 527, 534, 580, 626, 800, 801, 803, 819
Ae. mercurator	2, 329, 393, 636
Ae. pulchriventer	73, 141, 324, 325, 358, 458, 506, 534, 599
Ae. yunnanensis	325, 417, 441, 480, 599, 833
Ae. esoensis	2, 198, 329, 505
Ae. mediopunctatus	52, 57, 195, 345, 417, 527, 655, 848, 920
Ae. leucomelas	15, 17, 209, 329, 393, 636, 902
Ae. shortti	325, 334, 417, 418, 534
Ae. detritus	15, 209, 329, 393, 785, 890
Ae. sinkiangensis	15, 209
Ae. sasai	2, 329, 417, 505
Ae. caecus	206, 325, 432, 506, 527
Ae. galloisi	2, 14, 17, 329, 505, 553
Ae. seoulensis	265, 325, 329, 505, 513, 599, 775
Ae. flavopictus	14, 252, 325, 329, 505, 514, 553
Ae. implicatus	329, 636
Ae. sticticus	329, 636
Ae. malikuli	52, 57, 95, 101, 135, 160, 417, 534, 588

Ae. novoniveus	57, 101, 203, 325, 417, 441, 534
Ae. alboniveus	160, 358, 417-419, 599, 755
Ae. oreophilus	225, 325, 358, 458, 506, 534, 599, 775
Ae. niveoides	265, 410, 416, 417, 432, 441, 453, 506, 526
Ae. nipponicus	325, 329, 345, 417, 599
Ae. eldridgei	324, 335, 527, 580
Ae. saxicola	334, 441, 527, 613, 704
Stegomyia gardneriiimitator Leicester	580, 626, 658, 800, 803, 920
Ae. prominens	265, 345, 417, 775, 800
Ae. longifilamentus	15, 332
Ae. desmotes	195, 580, 626, 788, 920
Ae. mediolineatus	527, 736
Ae. perplexus	109, 325, 418, 599
Ae. watteni	14, 198, 265
Ae. alboscutellatus	265, 325, 334, 634, 788, 800
Ae. vittatus	334, 441, 527, 736
Ae. albolineatus	73, 141, 334, 658
Ae. chrysolineatus	109, 506, 613, 655
Ae. pionips	329, 636
Ae. mubiensis	198, 208, 527, 534
Ae. dissimilis	95, 417, 534, 658
Ae. intrudens	329, 636
Ae. riparius	329, 393
Ae. tonkinensis	265, 432, 534
Ae. peipingensis	325
Ae. omorii	410, 658, 848
Ae. gilli	141, 458, 775
Ae. sibiricus	2, 14
Ae. albotaeniatus mikiranus	506, 534
Ae. sedaensis	196, 873
Ae. galloisiodes	198
Ae. christophersi	197, 527
Ae. sergievi	329, 398, 697
Ae. hurlbuti	198
Ae. subalbopictus	198, 402
Ae. aureostriatus	334, 658
Ae. whartoni	534
Ae. kasachstanicus	209
Ae. ningheensis	599, 873
Ae. riparioides	331, 393
Ae. lasaensis gyirongensis	534
Ae. chungi	506
Ae. lasaensis	325, 534
Ae. occidentyunnanensis	582

Ae. unicinctus	458, 775
Ae. hexodontus	333
Ae. antuensis	329
Ae. alekurrovi	329
Ae. koreicoides	329
Ae. gonguoensis	198
Ae. novalbopictus	658
Ae. malayensis	658
Ae. vigilax	334
Ae. rhungkiangensis	487
Ae. ibis	527
Ae. andamanensis	130
Armigeres	6, 7, 9, 20, 27, 38, 42, 43, 52, 56, 57, 68, 71, 73, 75, 77, 78, 80, 89-91, 100, 101, 103, 106, 109, 112, 114, 115, 119-121, 136, 138, 141-145, 154, 155, 163, 165, 167, 169, 180, 184, 193, 195, 203, 213, 214, 216, 222, 225, 227, 236, 237, 244, 246, 249, 251, 256, 265, 270, 272, 279, 285, 289, 292, 312, 315, 325, 326, 334, 335, 339, 342, 344, 345, 347, 348, 358, 368, 372, 377, 378, 387, 388, 394, 401-403, 408, 410, 413, 416-419, 422, 429, 432-434, 438, 441, 449, 450, 454, 465-467, 469-473, 495, 497-499, 501, 506, 508, 517, 526, 527, 534, 541, 544-546, 552, 555, 557, 560, 565, 572, 574, 578-580, 587, 592-594, 597, 599, 600, 603, 605, 613, 619, 623, 626, 629, 631, 634, 638, 641, 642, 645, 646, 655, 656, 658, 660, 662, 663, 668, 677, 682, 683, 685, 689, 697, 700, 702, 704, 705, 715, 719, 722, 724, 725, 733, 737-740, 743, 745, 747, 754-756, 761, 763, 769-771, 773, 776, 780, 781, 787, 788, 793, 795, 800-803, 807, 809, 810, 812, 813, 816-819, 821, 830, 831, 833, 845, 848, 853, 861, 863-865, 872, 875, 877, 880, 882-886, 888, 892, 894, 896, 899, 903, 904, 906, 907, 910, 911, 913, 914, 916, 919, 920
Ar. subalbatus	6, 7, 9, 20, 27, 38, 42, 43, 52, 56, 57, 68, 71, 73, 75, 77, 78, 80, 91, 100, 101, 103, 106, 109, 112, 114, 115, 119-121, 136, 138, 141-145, 154, 155, 163, 165, 167, 169, 180, 184, 193, 195, 203, 213, 214, 216, 222, 225, 227, 236, 237, 244, 246, 249, 251, 256, 265, 270, 272, 279, 285, 289, 292, 312, 315, 325, 326, 334, 335, 339, 342, 344, 347, 348, 358, 368, 372, 377, 378, 387, 388, 394, 401-403, 408, 410, 413, 416-419, 422, 429, 432-434, 438, 441, 449, 450, 454, 465-467, 469-473, 495, 497-499, 501, 506, 508, 517, 526, 527, 534, 541, 544-546, 552, 555, 557, 560, 565, 572, 574, 578-580, 587, 592-594, 597, 599, 600, 603, 605, 613, 619, 623, 626, 629, 631, 634, 638, 641, 642, 645, 646, 655, 656, 658, 660, 662, 663, 668, 677, 682, 683, 685, 689, 697, 700, 702, 704, 705, 715, 719, 722, 725, 733, 737, 739, 740, 743, 745, 747, 754-756, 761, 763, 769-771, 773, 776, 780, 781, 787, 793, 795, 800-803, 807, 809, 810, 812, 813, 816-819, 821, 830, 831, 833, 845, 848, 853, 861, 863-865, 872, 875, 877, 880, 882-886, 888, 892, 894, 896, 899, 903, 904, 906, 907, 910, 911, 913, 914, 916, 919, 920
Ar. durhami	90, 106, 225, 325, 408, 410, 416-418, 506, 534, 599, 724, 800, 833, 848, 911
Ar. magnus	90, 154, 417, 506, 527, 534, 655, 800, 819, 920
Ar. annulitarsis	90, 580, 626, 655, 788, 819, 894, 920
Ar. flavus	90, 109, 527, 534, 626, 655, 769, 801, 819, 920
Ar. longipalpis	90, 655, 848, 920
Ar. digitatus	90, 109, 534, 655, 819, 894, 920
Ar. annulipalpis	90, 527, 819, 848
Ar. omissus	90, 655, 800, 819, 872, 920
Ar. inchoatus	109, 506, 527, 534, 580, 623, 655, 738, 788, 800, 819, 848, 872, 920
Ar. seticoxitus	90, 534, 599
Ar. theobaldi	90, 527, 599, 894, 920
Ar. baisasi	109, 534, 599
Ar. aureolineatus	141, 334, 599
Ar. malayi	345, 819
Ar. menglaensissp	848
Ar. lepidocoxitus	89
Culiseta	2, 15, 17, 104, 122, 175, 198, 209, 224, 265, 278, 325, 329, 340, 351, 358, 393, 441, 505, 522, 534, 599, 636, 669, 697, 833, 875, 902
Cs. niveitaeniata	175, 224, 265, 325, 358, 441, 522, 534, 599, 697, 833, 875
Cs. alaskaensis	15, 175, 209, 329, 340, 393, 505, 636, 902
Cs. bergrothi	2, 198, 278, 329, 393, 505, 636, 833
Cs. annulata	15, 122, 209, 340, 351
Cs. nipponica	2, 17, 329, 636, 669, 902
Cs. sinensis	325
Cs. mengaloba	2, 104
Tripteroides	52, 57, 101, 109, 135, 160, 203, 244, 265, 285, 325, 329, 334, 345, 368, 402, 410, 416-419, 429, 441, 466, 471, 505, 506, 527, 534, 553, 599, 655, 657, 658, 704, 755, 775, 800, 801, 848, 894, 920
Tr. bambusa	52, 57, 101, 160, 203, 265, 325, 329, 334, 368, 416-419, 429, 441, 466, 471, 505, 553, 599, 658, 755, 775

Tr. aranoides	52, 57, 101, 109, 244, 285, 325, 334, 368, 402, 416-418, 441, 506, 527, 534, 599, 655, 704, 800, 801, 894, 920
Tr. similis	52, 109, 135, 160, 345, 368, 417-419, 534, 755
Tr. palldothorax	410, 848
Tr. indicus	441
Tr. tarsalis	657
Tr. szechwanensis	325
Mansonia	20, 73, 75, 77, 106, 136, 137, 141, 144, 145, 154, 163, 168, 209, 226, 227, 244, 251, 263, 265, 312, 325, 339, 364, 389, 398, 410, 413, 417, 428, 432, 441, 447, 491, 506, 527, 534, 557, 599, 605, 623, 626, 662, 689, 693, 697, 720, 728, 776, 800-804, 813, 819, 848, 856, 865, 872, 877, 894
Ma. uniformis	20, 77, 106, 136, 137, 141, 144, 145, 154, 163, 168, 209, 227, 244, 251, 263, 265, 312, 325, 339, 364, 389, 398, 410, 413, 417, 428, 441, 447, 491, 506, 527, 534, 557, 599, 605, 623, 626, 662, 689, 693, 697, 720, 728, 776, 800-804, 813, 819, 848, 865, 877, 894
Ma. ochracea	73, 141, 389
Ma. annulifera	800, 872, 894
Ma. crassipes	145, 417, 432
Ma. bonneae	75, 226
Ma. richiardii	209, 856
Ma. dives	872
Uranotaenia	52, 57, 101, 109, 145, 160, 198, 225, 244, 265, 307, 325, 334, 345, 368, 384, 416-419, 432, 466, 471, 480, 506, 527, 534, 599, 623, 658, 694, 704, 755, 800, 872, 873, 880, 894
Ur. novobscura	52, 57, 101, 109, 160, 225, 244, 325, 334, 368, 416-419, 471, 506, 534, 599, 658, 704, 755, 872, 880, 894
Ur. macfarlanei	57, 109, 265, 325, 417, 418, 480, 527
Ur. maxima	345, 416-418, 623, 872
Ur. nivipleura	325, 417, 534, 623, 658, 800, 880, 894
Ur. annandalei	57, 198, 334, 417, 418, 480
Ur. bicolor	198, 417, 418, 534
Ur. bimaculata	527
Ur. koli	57, 694
Ur. enigmatica	694, 894
Ur. abdita	334, 506
Ur. hebes	384, 417, 418
Ur. recondita	145, 658
Ur. campestris	466
Ur. spiculosa	109
Ur. lutescens	109
Ur. testacea	894
Ur. leiboensls	307
Ur. unguiculata	432
Ur. jacksoni	325
Ur. yaeyamana	198
Ur. mengi	873
Toxorhynchites	57, 102, 103, 109, 160, 203, 244, 265, 325, 329, 330, 334, 345, 416-419, 441, 458, 506, 527, 534, 613, 642, 658, 755, 775, 910
Tx. gravelyi	57, 109, 160, 203, 325, 334, 345, 416-419, 441, 506, 534, 755
Tx. Splendens	102, 103, 109, 244, 506, 527, 642, 910
Tx. edwardsi	109, 160, 265, 325, 417-419
Tx. aurifluus	458, 658, 775
Tx. changbaiensis	329, 330
Tx. splendenroides	613
Tx. nigerrmus	613
Tx. kempi	109
Orthopodomyia	52, 57, 109, 134, 265, 325, 334, 368, 416-419, 441, 471, 506, 527, 534, 658, 755, 775, 848
Or. anopheloides	52, 57, 109, 265, 325, 368, 416-419, 441, 471, 506, 527, 534, 658, 755, 775, 848

Or. andamanensis	134
Or. albipes	334
Mimomyia	52, 106, 160, 265, 345, 410, 417, 527, 534, 578, 623, 755, 872, 880
Mi. luzonensis	52, 106, 265, 410, 417, 527, 534, 578, 623, 872, 880
Mi. fusca	160, 345, 417, 755
Mi. chamberlaini	527
Heizmannia	52, 57, 87, 101, 132, 334, 368, 417-419, 506, 527, 534, 580, 613, 626, 655, 658, 800, 801, 803, 819, 920
He. reidi	334, 534, 580, 613, 626, 658, 800, 801, 803, 819, 920
He. lii	52, 57, 101, 368, 417-419, 658, 819
He. macdonaldi	534, 580, 655, 801
He. chengi	506, 534, 658
He. menglianensis	580, 800
He. taiwanensis	334, 658
He. Lui	132
He. ruiliensis	87
He. achaetae	658
He. proxima	334
He. covelli	527
Topomyia	81, 83, 86, 88, 109, 128, 129, 131, 133, 152, 271, 325, 334, 527, 534, 599, 873
To. houghtoni	86, 109, 152, 325, 334, 527, 534, 599
To. zhangi	131, 152, 534
To. hirtysa	86, 129, 152, 534
To. lindsayi	86, 109
To. inclinata	109, 133
To. apsarae	83
To. hirtusa	88
To. longisetosa	86
To. baolini	86
To. cristata	86, 128
To. sylvatica	271
To. yanbaresoides	81
To. bannaensis	873
To. dulonggensis	873
To. mengi	873
To. margina	873
Coquillettidia	168, 364, 447, 491, 580, 728, 833, 894
Co. ochracea	168, 364, 447, 491, 580, 728
Co. crassipes	833, 894
Malaya	334, 417, 506, 527, 534, 626, 704
Ml. genurostris	334, 417, 506, 527, 534, 626, 704
Ml. jacobsoni	334
Udaya argyrurus	35, 109, 534
Hodgesia bailyi	158
Ficalbia minima	894

Supplementary Table S2. Information of data sources in this study.

Variable	Source	Note	Data period
Climate data	http://data.cma.cn/data/cdcdetail/dataCode/SURF_CLI_CHN_MUL_MMON_19812010.html	The climate data are accessible in this website from 1981 to 2010. Data from 2010 to 2018 was accessible in past yet not open now, which can be accessible by contact the data source organization.	1981–2018
Land cover	https://www.resdc.cn/Datalist1.aspx?FieldTyepID=1,3	A raster digital map with a resolution of 1km.	1985–2015
Livestock density	https://dataverse.harvard.edu/dataverse/glw_3	Including the density of sheep, goat, cattle, pig, buffalo, duck and chicken with a resolution of 5 min of arc.	2010
Mammalian richness	https://sedac.ciesin.columbia.edu/data/set/species-global-mammal-richness-2015	Provide information on the number of mammal species present at a 1km spatial resolution.	2013
Population density and proportion of older and female	http://www.stats.gov.cn/tjzs/tjsj/tjcb/tjzl/201303/t20130318_44794.html	The number of population, older and female at the county level.	2010

Supplementary Table S3. Potential risk factors at the county level used in the BRT model for mosquito species and 2-stage GBRT model for Dengue and JE.

Category	Variables	Description (Unit)	Mosquitoes	Dengue	JE
Environmental	Plain	Situated on a plain	√	√	√
	Basin	Situated in a basin	√	√	√
	Mountain	Situated in a mountainous region	√	√	√
	Hilly	Situated in a hilly region	√	√	√
	Plateau	Situated on a plateau	√	√	√
	Coastland	Situated in the seaside	√	√	√
	Paddy field	Coverage of paddy field	√	√	√
	Rainfed cropland	Coverage of rainfed cropland	√	√	√
	Forest	Coverage of forest	√	√	√
	Grassland	Coverage of grassland	√	√	√
	River	Coverage of river	√	√	√
	Permanent glacial snow	Coverage of permanent glacial snow	√	√	√
	Lake and reservoir	Coverage of lake and reservoir	√	√	√
	Mud flat and shoaly land	Coverage of mud flat and shoaly land	√	√	√
	Rural settlement	Coverage of rural settlement	√	√	√
	Other construction land	Coverage of other construction land (roads, mines, airports, etc.)	√	√	√
	Saline and alkaline land	Coverage of saline and alkaline land	√	√	√
	Marsh Land	Coverage of marsh land	√	√	√
Ecoclimatic	Bio1	Annual average temperature (℃)	√	√	√
	Bio2	Mean diurnal range (Mean of monthly max - min temperature) (℃)			
	Bio3	Isothermality (BIO02 ÷ BIO07 ×100)	√	√	√
	Bio4	Temperature seasonality (standard deviation ×100)	√	√	√
	Bio5	Max temperature of warmest month (℃)			
	Bio6	Min temperature of coldest month (℃)			
	Bio7	Annual range of temperature (BIO05-BIO06) (℃)			
	Bio8	Mean temperature of wettest quarter (℃)	√	√	√
	Bio9	Mean temperature of driest quarter (℃)			
	Bio10	Mean temperature of warmest quarter (℃)	√	√	√
	Bio11	Mean temperature of coldest quarter (℃)			
	Bio12	Annual cumulative precipitation (mm)	√	√	√
	Bio13	Precipitation of wettest month (mm)			
	Bio14	Precipitation of driest month (mm)			

	Bio15	Precipitation seasonality (Coefficient of variation)	√	√	√
	Bio16	Precipitation of wettest quarter (mm)			
	Bio17	Precipitation of driest quarter (mm)	√	√	√
	Bio18	Precipitation of warmest quarter (mm)			
	Bio19	Precipitation of coldest quarter (mm)			
Social	Index of case importation	Logarithmic transformed number of imported dengue cases		√	
	Over 60 years old	Proportion of ≥60 years old		√	√
	Female	Proportion of female population		√	√
	General hospital	Number of general hospitals		√	√
	Specialty hospital	Number of specialty hospitals		√	√
	Clinics	Number of clinics hospitals		√	√
	Health centers	Number of health centers		√	√
	Emergency Rooms	Number of ERs		√	√
Biological	Population density	Human population density (persons per km^2)	√	√	√
	Sheep	Density of sheep (10 heads per km^2)	√	√	√
	Goat	Density of goat (10 heads per km^2）	√	√	√
	Cattle	Density of cattle (10 heads per km^2)	√	√	√
	Pig	Density of pig (10 heads per km^2)	√	√	√
	Buffalo	Density of buffalo (10 heads per km^2)	√	√	√
	Duck	Density of duck (10 heads per km^2)	√	√	√
	Chicken	Density of chicken (10 heads per km^2)	√	√	√
	Mammalian richness	The number of mammal species per km^2	√	√	√
	Cx. tritaeniorhynchus	Presence possibility of *Cx. tritaeniorhynchus*			√
	Cx. pipiens quinquefasciatus	Presence possibility of *Cx. pipiens quinquefasciatus*			√
	Cx. pipiens pallens	Presence possibility of *Cx. pipiens pallens*			√
	An. sinensis	Presence possibility of *An. sinensis*			√
	Ae. albopictus	Presence possibility of *Ae. albopictus*		√	
	Ae. aegypti	Presence possibility of *Ae. aegypti*		√	

Supplementary Table S4. Clustering analysis of eco-climatic predictors at the county level based on pairwise Pearson correlation coefficients.

	bio1*	bio2*	bio3*	bio4*	bio5*	bio6	bio7	bio8	bio9	bio10	bio11	bio12*	bio13	bio14	bio15*	bio16	bio17*	bio18	bio19
bio1						0.967			0.962		0.965								
bio2																			
bio3																			
bio4							0.967												
bio5								0.815		0.880									
bio6									0.965		0.992								
bio7																			
bio8										0.934									
bio9											0.975								
bio10																			
bio11																			
bio12													0.961			0.973		0.893	
bio13																0.993		0.950	
bio14																	0.970		0.924
bio15																			
bio16																		0.955	
bio17																			0.981
bio18																			
bio19																			

Pairwise correlations above 0.8 are shown, and blank off-diagonal cells all have correlations < 0.8. Predictors grouped to the same cluster are colored the same. From each cluster, only one predictor (marked with *) is chosen to be used in county-level BRT models to avoid multicollinearity.

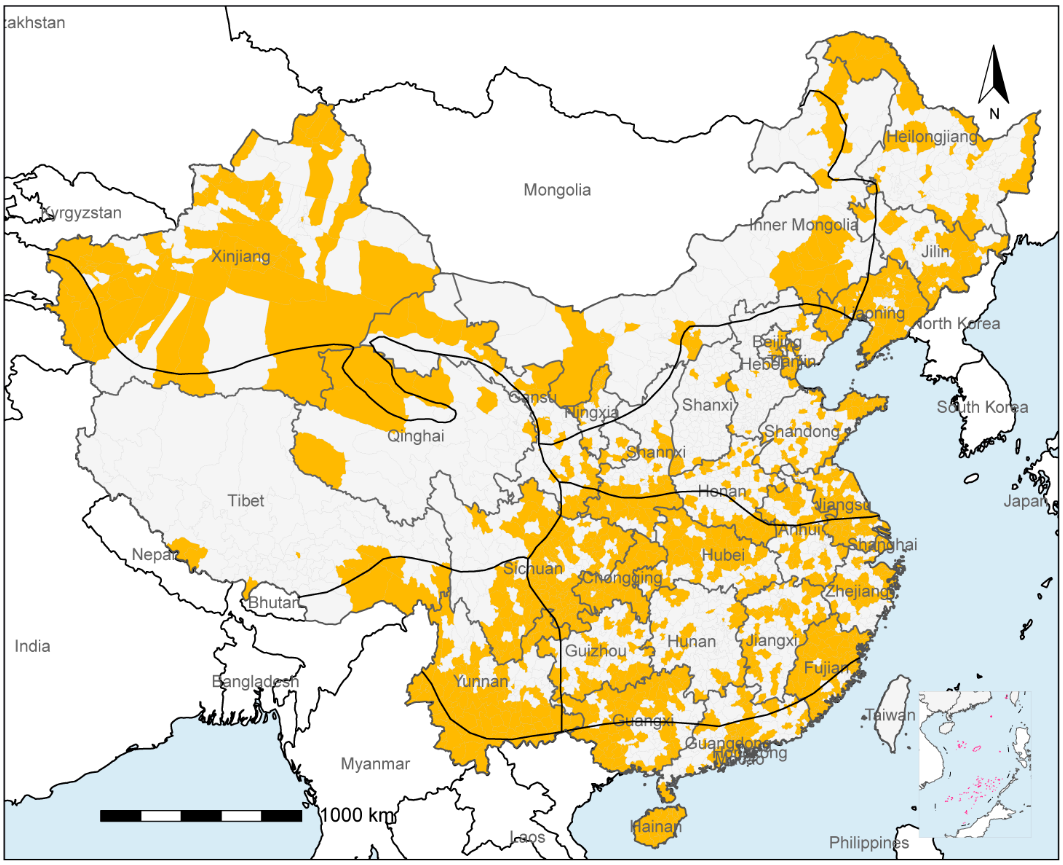

Supplementary Figure S2 The spatial distribution of the 1228 counties with at least one record of mosquitoes (yellow) from 1954 to 2020, China. Source data are provided as a Source Data file.

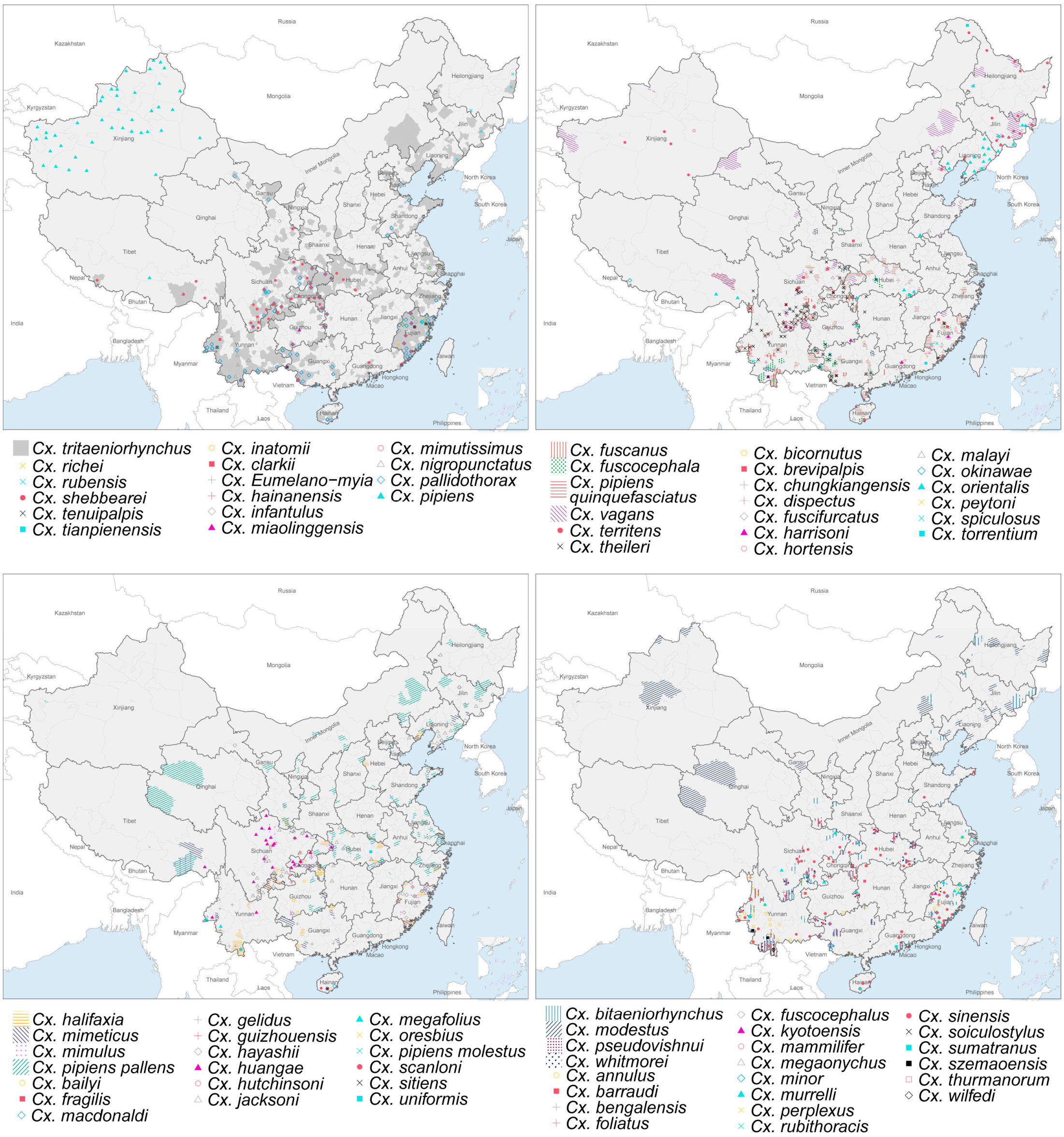

Supplementary Figure S3 The spatial distribution of the mosquito genus *Culex* recorded at the county level from 1954 to 2020 in China. Source data are provided as a Source Data file.

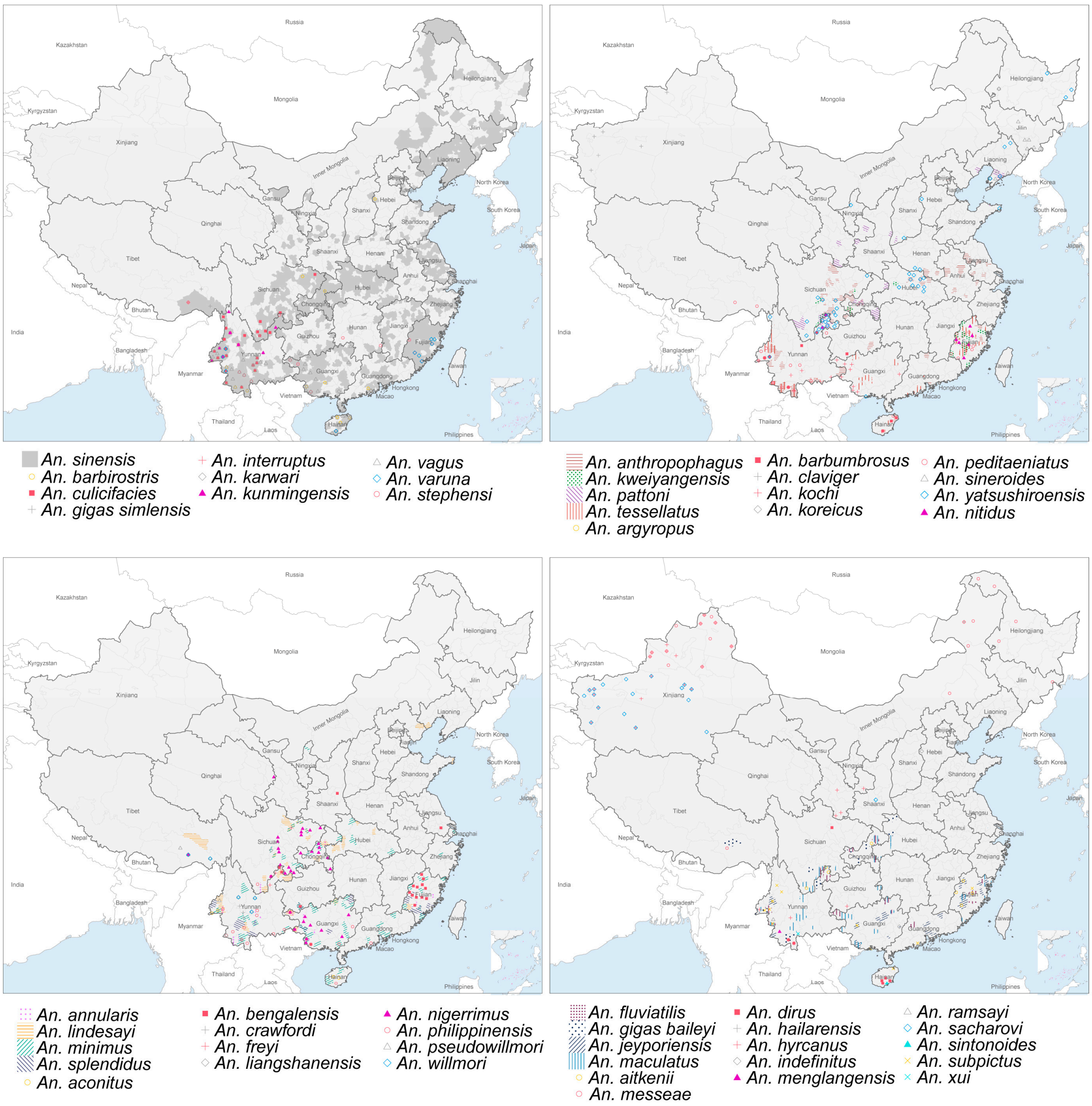

Supplementary Figure S4 The spatial distribution of the mosquito genus *Anopheles* recorded at the county level from 1954 to 2020 in China. Source data are provided as a Source Data file.

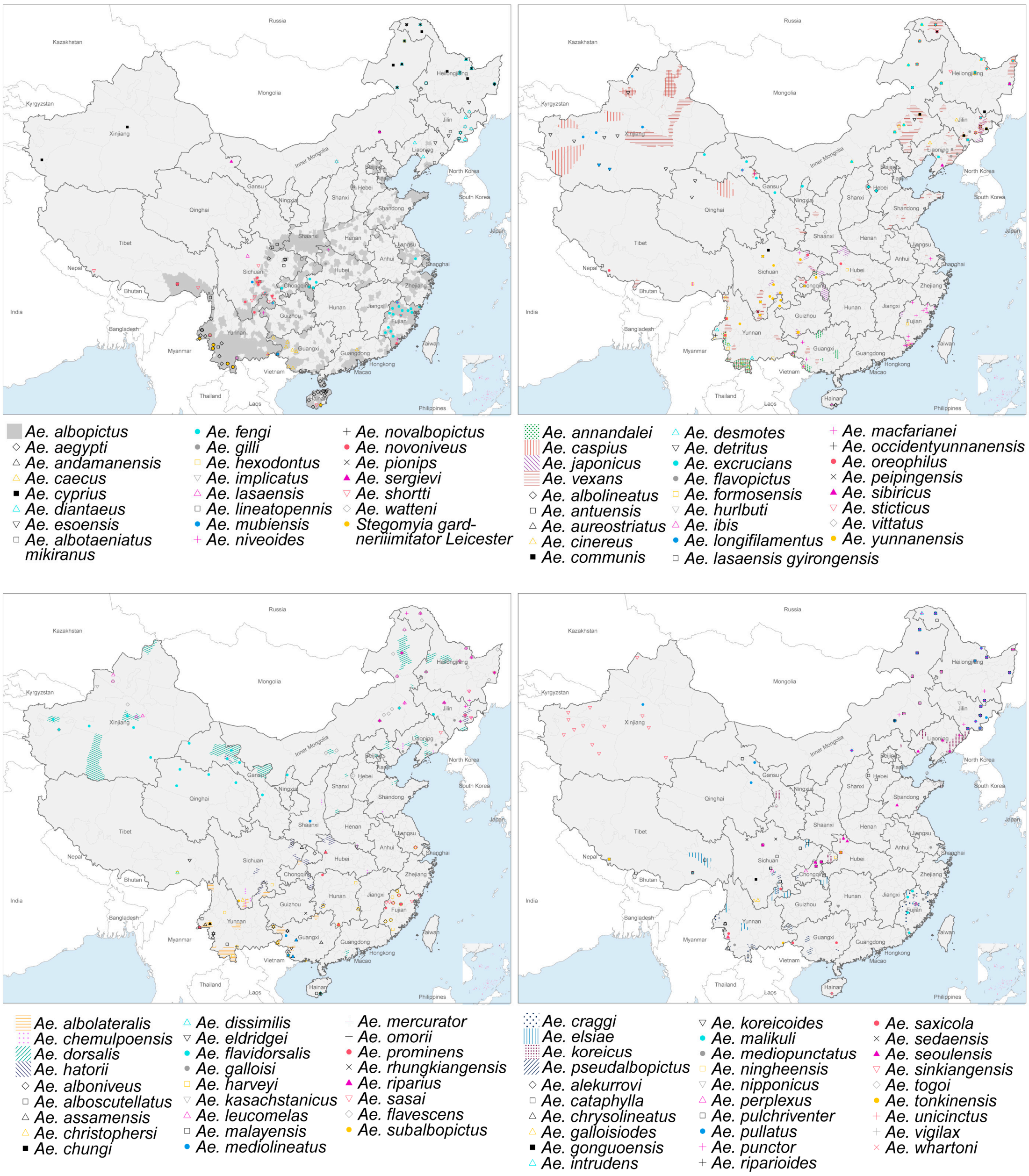

Supplementary Figure S5 The spatial distribution of the mosquito genus *Aedes* recorded at the county level from 1954 to 2020 in China. Source data are provided as a Source Data file.

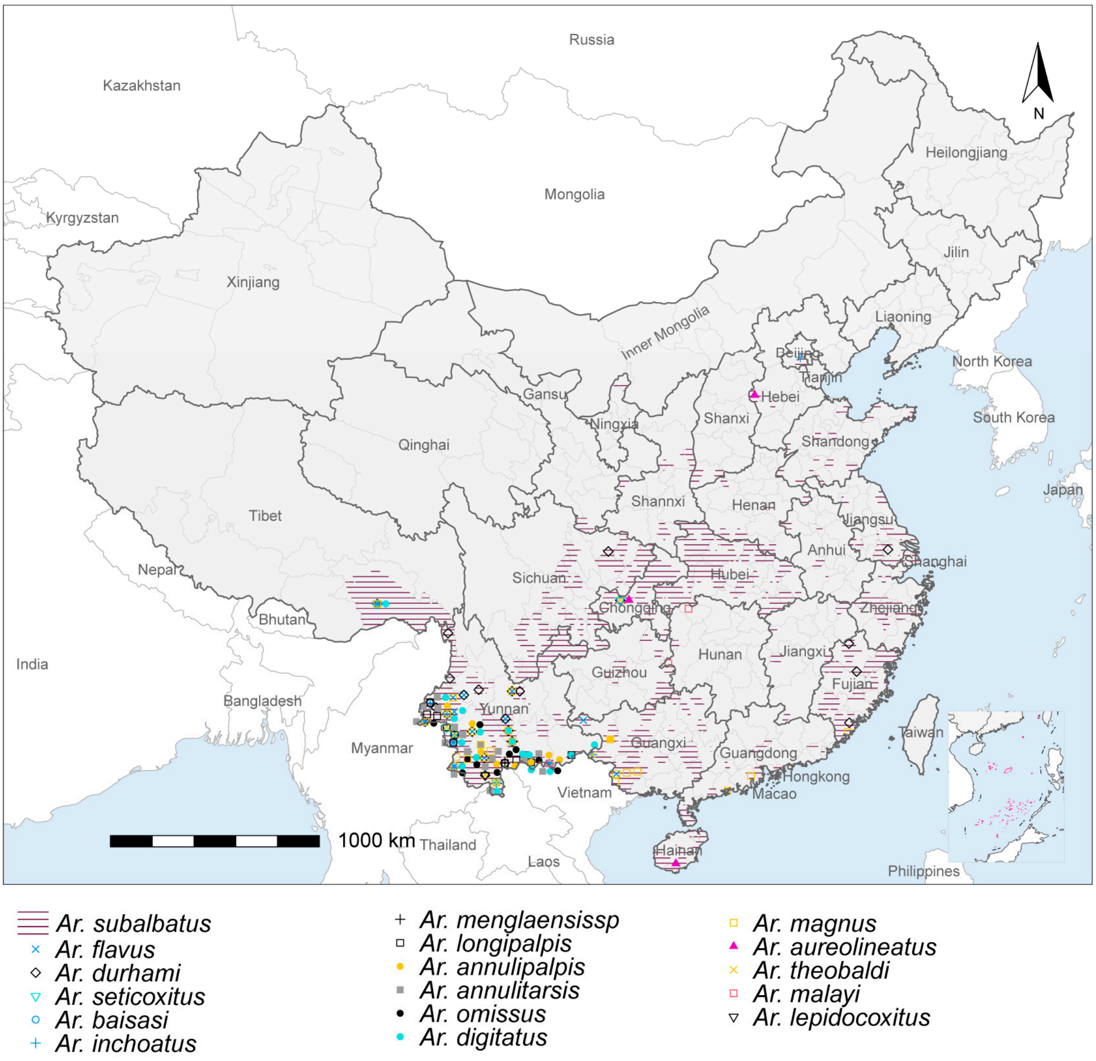

Supplementary Figure S6 The spatial distribution of the mosquito genus *Armigeres* recorded at the county level from 1954 to 2020 in China. Source data are provided as a Source Data file.

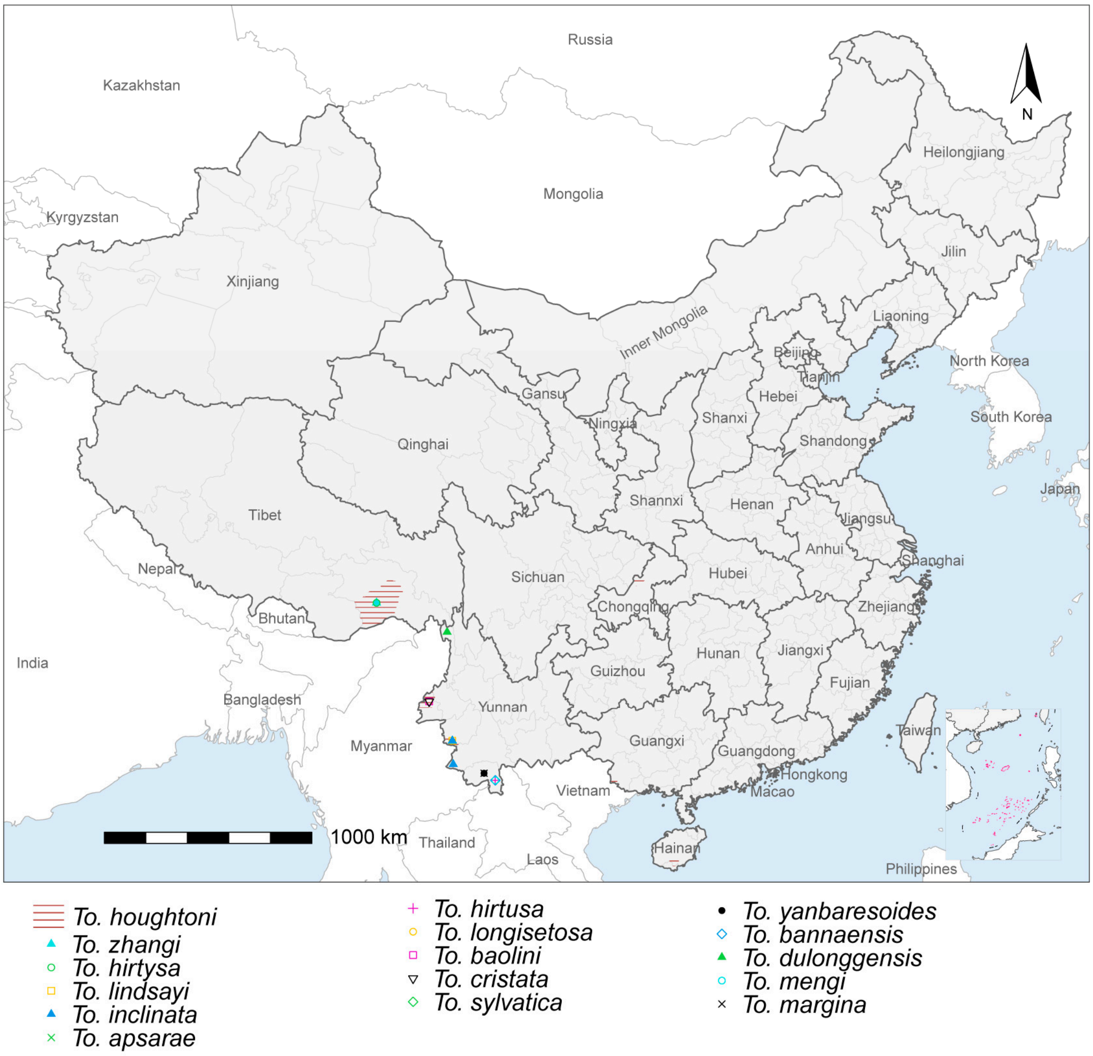

Supplementary Figure S7 The spatial distribution of the mosquito genus *Topomyia* recorded at the county level from 1954 to 2020 in China. Source data are provided as a Source Data file.

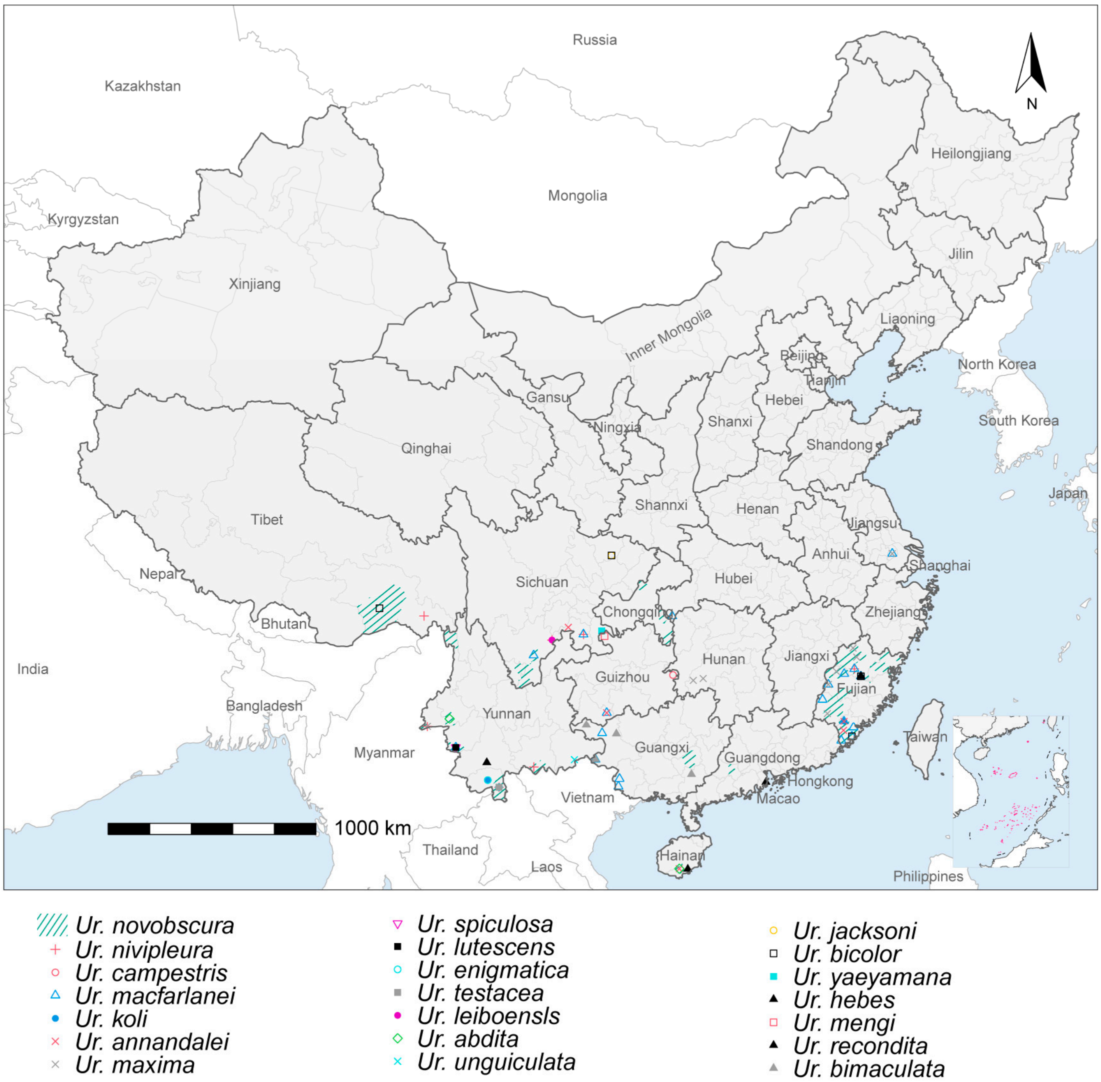

Supplementary Figure S8 The spatial distribution of the mosquito genus *Uranotaenia* recorded at the county level from 1954 to 2020 in China. Source data are provided as a Source Data file.

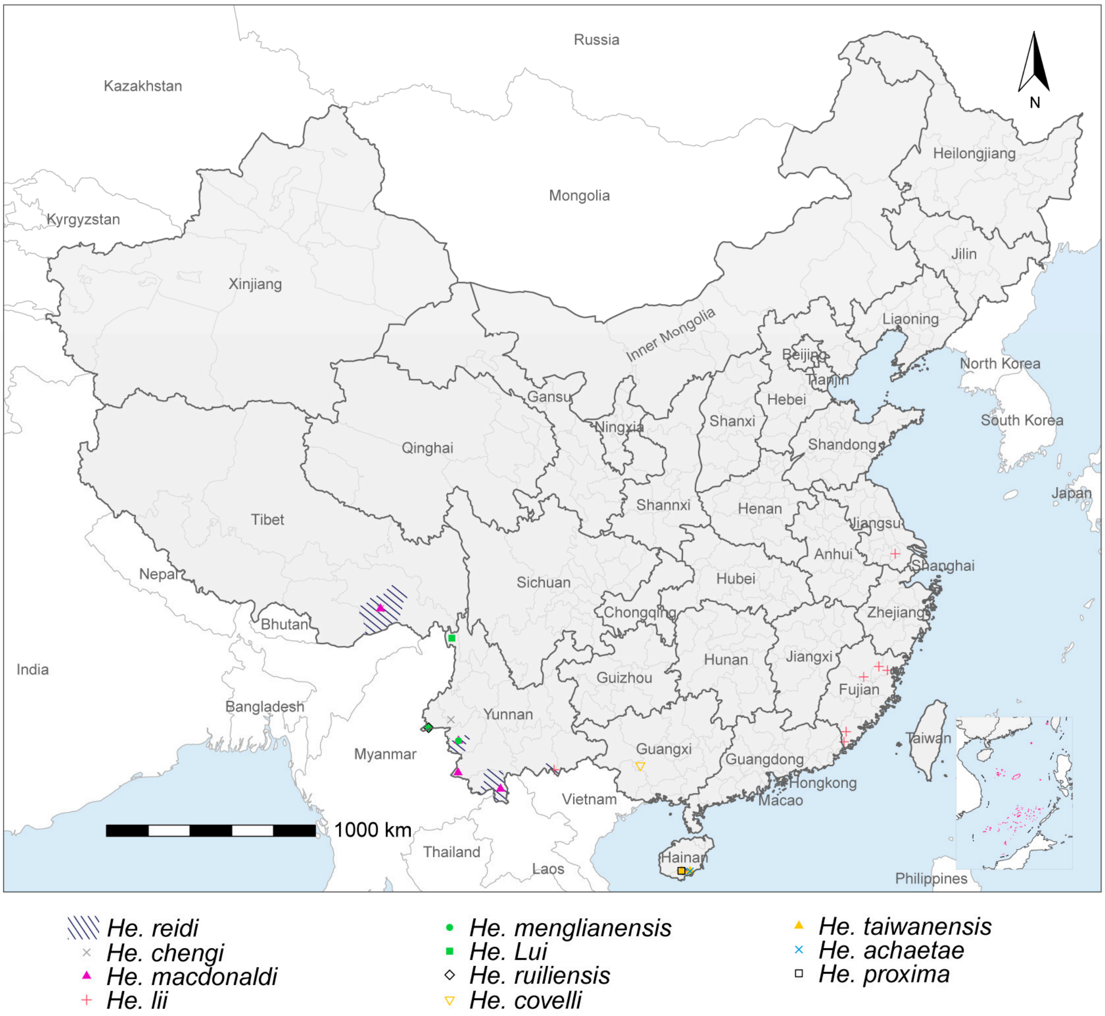

Supplementary Figure S9 The spatial distribution of the mosquito genus *Heizmannia* recorded at the county level from 1954 to 2020 in China. Source data are provided as a Source Data file.

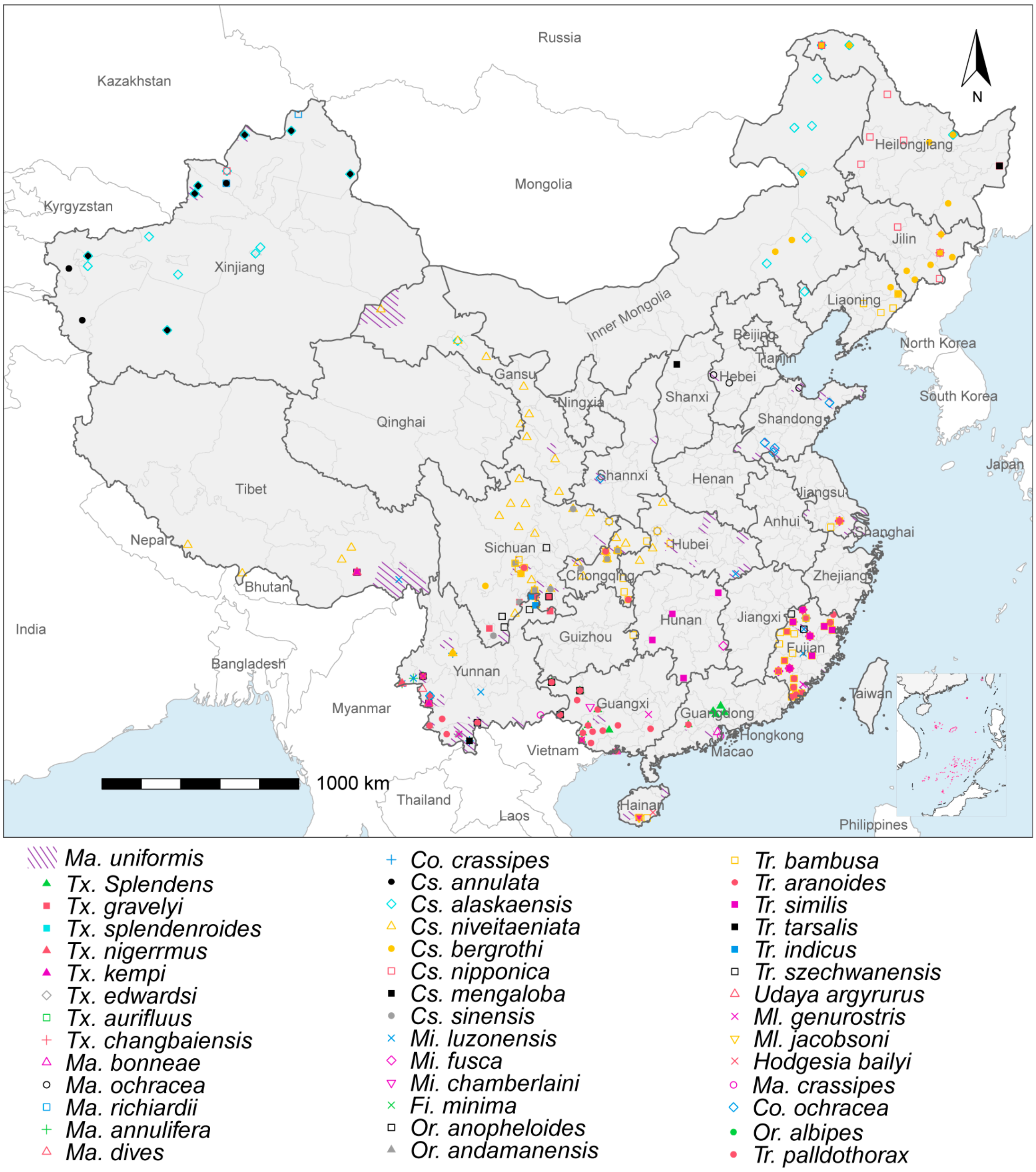

Supplementary Figure S10 The spatial distribution of the other Relatively rare mosquito genus (<10 species) recorded at the county level from 1954 to 2020 in China. The relatively rare mosquito genera include *Mansonia*, *Toxorhynchites*, *Coquillettidia*, *Culiseta*, *Mimomyia*, *Ficalbia*, *Orthopodomyia*, *Tripteroides*, *Udaya*, *Malaya*, *Hodgesia* and *Mansonia*. Source data are provided as a Source Data file.

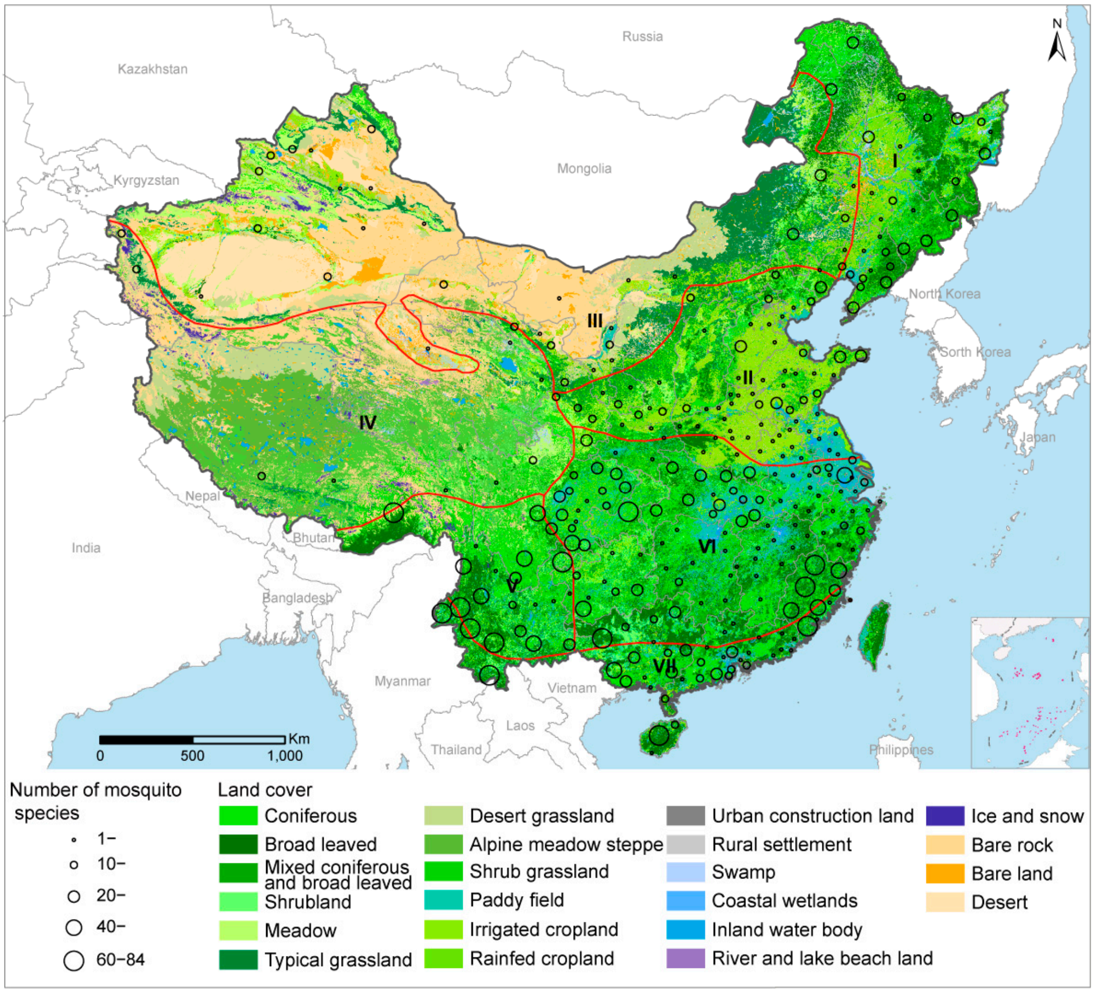

Supplementary Figure S11 Mosquito species richness (circles) at the prefecture level in seven biogeographic zones in mainland China from 1954 to 2020. I=Northeast district (NE), II=North China district (N), III=Inner Mongolia-Xinjiang district (IMX), IV= Qinghai-Tibet district (QT), V=Southwest China district (SW), VI=Central China district (C) and VII=South China district (S).

Supplementary Table S5. BRT-model-estimated mean (standard deviation) relative contributions of top factors (RC≥5%) to the spatial distribution of three most prevalent mosquito species in the *Anopheles* genus.

Category	Variable	*An. sinensis*	*An. anthropophagus*	*An. minimus*	*An. maculatus*	*An. pattoni*	*An. lindesayi*	*An. jeyporiensis*	*An. tessellatus*
**Environmental**	Basin					6.21 (1.99)			
	Paddy Field	6.25 (1.62)	6.60 (1.67)						
	Rainfed Cropland					7.97 (1.70)			
	Forest				6.41 (1.49)	7.56 (1.81)	5.97 (1.99)		
	Lake and Reservoir							5.08 (1.45)	
	Rural Residential Land						9.51 (2.35)		
	Other Construction Land		5.31 (1.08)						
**Ecoclimatic**	Annual Mean Temperature		10.14 (2.61)	21.22 (5.39)		5.47 (1.44)		25.16 (4.08)	22.28 (5.05)
	Isothermality				5.05 (2.32)				8.63 (3.30)
	Temperature Seasonality		16.62 (2.27)	17.93 (4.56)	22.57 (3.24)	8.72 (2.11)		7.57 (1.84)	11.01 (3.61)
	Mean Temperature of Warmest Quarter		7.21 (1.67)						
	Total precipitation	42.03 (5.25)		11.27 (3.28)				15.03 (3.60)	9.70 (1.98)
	Precipitation of Driest Quarter	5.76 (1.27)				5.92 (1.08)		5.17 (1.50)	
**Biological factors**	Population Density	6.65 (1.63)	5.78 (1.00)	12.16 (1.83)	5.96 (1.63)	5.03 (1.50)		5.33 (1.29)	
	Mammalian Richness		6.15 (1.24)	7.62 (1.56)	6.85 (2.56)	7.13 (1.95)	10.86 (2.68)	6.45 (1.69)	
	Pig	5.72 (1.16)				6.43 (2.09)	5.79 (1.94)		
	Buffalo		12.99 (2.77)	8.99 (2.88)					
	Cattle		5.55 (1.03)						5.29 (1.50)
	Duck	9.14 (4.14)					5.28 (1.85)		6.18 (1.64)
	Goat			5.71 (1.07)				5.45 (1.32)	
	Sheep							5.21 (1.18)	

**AUC**	Train	0.982 (0.967, 0.996)	0.991 (0.984, 0.998)	0.955 (0.940, 0.972)	0.988 (0.976, 0.997)	0.993 (0.985, 0.999)	0.966 (0.945, 0.985)	0.987 (0.977, 0.996)	0.995 (0.988, 1.000)
	Test	0.874 (0.811, 0.920)	0.904 (0.874, 0.935)	0.859 (0.820, 0.893)	0.878 (0.817, 0.929)	0.873 (0.819, 0.926)	0.793 (0.715, 0.859)	0.906 (0.852, 0.955)	0.915 (0.873, 0.953)
**Partial AUC Ratio**	Train	1.15	1.62	1.61	1.76	1.78	1.80	1.80	1.81
	Test	1.13	1.61	1.55	1.72	1.67	1.59	1.80	1.81

Mean AUCs (95% percentiles) and partial area AUC ratio (calculated at tolerance level of 0.2) are given

Supplementary Table S6. BRT-model-estimated mean (standard deviation) relative contributions of top factors (RC≥5%) to the spatial distribution of three most prevalent mosquito species in the *Culex* genus.

Category	Variable	*Cx. tritaeniorhynchus*	*Cx. pipiens quinquefasciatus*	*Cx. pipiens pallens*	*Cx. bitaeniorhynchus*	*Cx. vagans*	*Cx. halifaxia*	*Cx. modestus*
**Environmental**	Rainfed Cropland							5.65 (1.30)
	Forest					10.16 (2.39)		5.31 (1.35)
	Grasslands						5.52 (1.28)	
	Lake and Reservoir				5.38 (1.62)			
	Rural Residential Land						7.97 (1.50)	
**Ecoclimatic**	Annual Mean Temperature	7.07 (1.77)	5.70 (1.38)			28.21 (4.30)		17.03 (6.19)
	Isothermality					6.16 (1.61)		
	Temperature Seasonality	13.55 (3.33)	59.87 (3.31)	61.01 (2.24)	8.88 (2.58)		13.11 (2.14)	44.59 (6.21)
	Mean Temperature of Warmest Quarter					11.03 (3.12)	6.02 (1.09)	5.50 (1.51)
	Total precipitation	11.77 (3.64)		12.67 (2.01)	11.74 (2.73)	10.19 (2.22)	7.50 (1.45)	
	Precipitation Seasonality	5.66 (1.06)		5.93 (1.03)	6.45 (1.61)	9.48 (2.14)		
	Precipitation of Driest Quarter	7.06 (1.31)	6.38 (1.09)					
**Biological factors**	Population Density	8.23 (2.75)						
	Mammalian Richness		5.96 (0.91)				5.68 (1.98)	
	Pig	13.46 (3.98)						
	Buffalo	5.35 (1.25)	6.77 (1.32)					
	Duck	6.18 (1.52)			6.64 (1.88)	6.84 (1.70)	8.09 (1.60)	
**AUC**	Train	0.973 (0.945, 0.991)	0.976 (0.967, 0.987)	0.990 (0.984, 0.995)	0.936 (0.900, 0.971)	0.930 (0.907, 0.957)	0.978 (0.953, 0.996)	0.982 (0.974, 0.991)
	Test	0.827 (0.771, 0.874)	0.897 (0.853, 0.930)	0.940 (0.910, 0.967)	0.682 (0.629, 0.734)	0.772 (0.690, 0.843)	0.756 (0.697, 0.818)	0.926 (0.876, 0.962)
**Partial AUC Ratio**	Train	1.23	1.42	1.44	1.57	1.67	1.70	1.75
	Test	1.19	1.35	1.42	1.18	1.46	1.47	1.72

Supplementary Table S6 continued

Category	Variable	*Cx. fuscanus*	*Cx. mimeticus*	*Cx. pseudovishnui*	*Cx. fuscocephala*	*Cx. whitmorei*	*Cx. mimulus*
**Environmental**	Paddy Field						6.28 (2.03)
	Forest		10.62 (2.26)				
	Grasslands					6.03 (1.39)	
	River				5.74 (2.03)	6.37 (1.89)	
	Lake and Reservoir				5.43 (1.77)	5.49 (1.73)	
	Rural Residential Land		5.63 (1.42)				11.24 (2.64)
	Other Construction Land						6.37 (1.88)
**Ecoclimatic**	Annual Mean Temperature				6.10 (2.28)		
	Isothermality		6.46 (1.53)	5.58 (2.14)	12.95 (4.99)		
	Temperature Seasonality	17.64 (2.73)	10.10 (2.18)	12.64 (3.62)	26.63 (5.85)	7.41 (2.95)	10.02 (2.25)
	Total precipitation	5.40 (1.50)	5.88 (1.59)	11.59 (2.55)	7.28 (2.24)	7.27 (1.88)	8.31 (2.11)
	Precipitation Seasonality	5.64 (1.32)	5.75 (1.32)				
	Precipitation of Driest Quarter	5.08 (1.46)	6.86 (1.45)	5.79 (1.79)		6.71 (1.98)	7.21 (1.85)
**Biological factors**	Population Density			6.74 (1.96)	7.08 (2.45)		
	Mammalian Richness		9.04 (2.71)	17.70 (3.94)	7.35 (2.66)	8.70 (3.24)	5.85 (1.71)
	Buffalo	5.49 (1.59)			5.86 (1.87)	8.64 (2.62)	
	Cattle	5.08 (1.17)					6.17 (1.83)
	Duck	5.33 (1.25)	5.78 (1.48)				
	Goat				5.44 (1.87)		6.93 (2.25)
	Sheep	5.26 (1.36)					5.20 (1.58)
**AUC**	Train	0.979 (0.959, 0.994)	0.967 (0.929, 0.992)	0.969 (0.950, 0.986)	0.958 (0.940, 0.981)	0.978 (0.945, 0.998)	0.981 (0.960, 0.996)
	Test	0.799 (0.742, 0.857)	0.753 (0.655, 0.837)	0.822 (0.751, 0.884)	0.846 (0.746, 0.919)	0.765 (0.698, 0.837)	0.758 (0.683, 0.818)
**Partial AUC Ratio**	Train	1.76	1.76	1.79	1.81	1.81	1.84
	Test	1.51	1.36	1.60	1.67	1.41	1.56

Mean AUCs (95% percentiles) and partial area AUC ratio (calculated at tolerance level of 0.2) are given.

Supplementary Table S7. BRT-model-estimated mean (standard deviation) relative contributions of top factors (RC≥5%) to the spatial distribution of three most prevalent mosquito species in the *Aedes* and *Armigeres* genus.

Category	Variable	*Ae. albopictus*	*Ae. vexans*	*Ae. dorsalis*	*Ae. aegypti*	*Ar. subalbatus*
**Ecoclimatic**	Paddy Field					5.80 (1.12)
	Rainfed Cropland					5.15 (1.09)
	Forest		5.09 (1.13)			5.90 (0.96)
	River				5.51 (2.96)	
	Rural Residential Land				10.01 (4.08)	
	Other Construction Land		5.31 (1.18)	5.37 (1.34)		
**Environmental**	Annual Mean Temperature	40.58 (2.95)	7.84 (3.22)	14.08 (4.69)	18.55 (7.29)	41.88 (4.46)
	Isothermality		9.58 (1.39)		7.51 (3.47)	
	Temperature Seasonality	10.80 (1.86)	17.58 (3.68)	34.23 (4.54)	45.49 (7.20)	13.35 (3.32)
	Mean Temperature of Warmest Quarter		22.96 (5.18)			
	Total precipitation	8.97 (1.56)		7.74 (2.06)		
	Precipitation of Driest Quarter	5.39 (1.27)	5.14 (1.14)			
**Biological factors**	Population Density	7.31 (1.04)				
	Mammalian Richness	5.66 (0.96)				7.89 (1.47)
	Pig	5.82 (1.00)				
	Sheep		6.26 (1.32)			
**AUC**	Train	0.974 (0.959, 0.988)	0.945 (0.920, 0.967)	0.990 (0.982, 0.998)	0.997 (0.995, 0.999)	0.967 (0.954, 0.985)
	Test	0.870 (0.827, 0.902)	0.820 (0.758, 0.869)	0.932 (0.890, 0.962)	0.951 (0.838, 0.996)	0.839 (0.786, 0.885)
**Partial AUC Ratio**	Train	1.33	1.58	1.78	1.94	1.43
	Test	1.30	1.38	1.76	1.94	1.38

Mean AUCs (95% percentiles) and partial area AUC ratio (calculated at tolerance level of 0.2) are given.

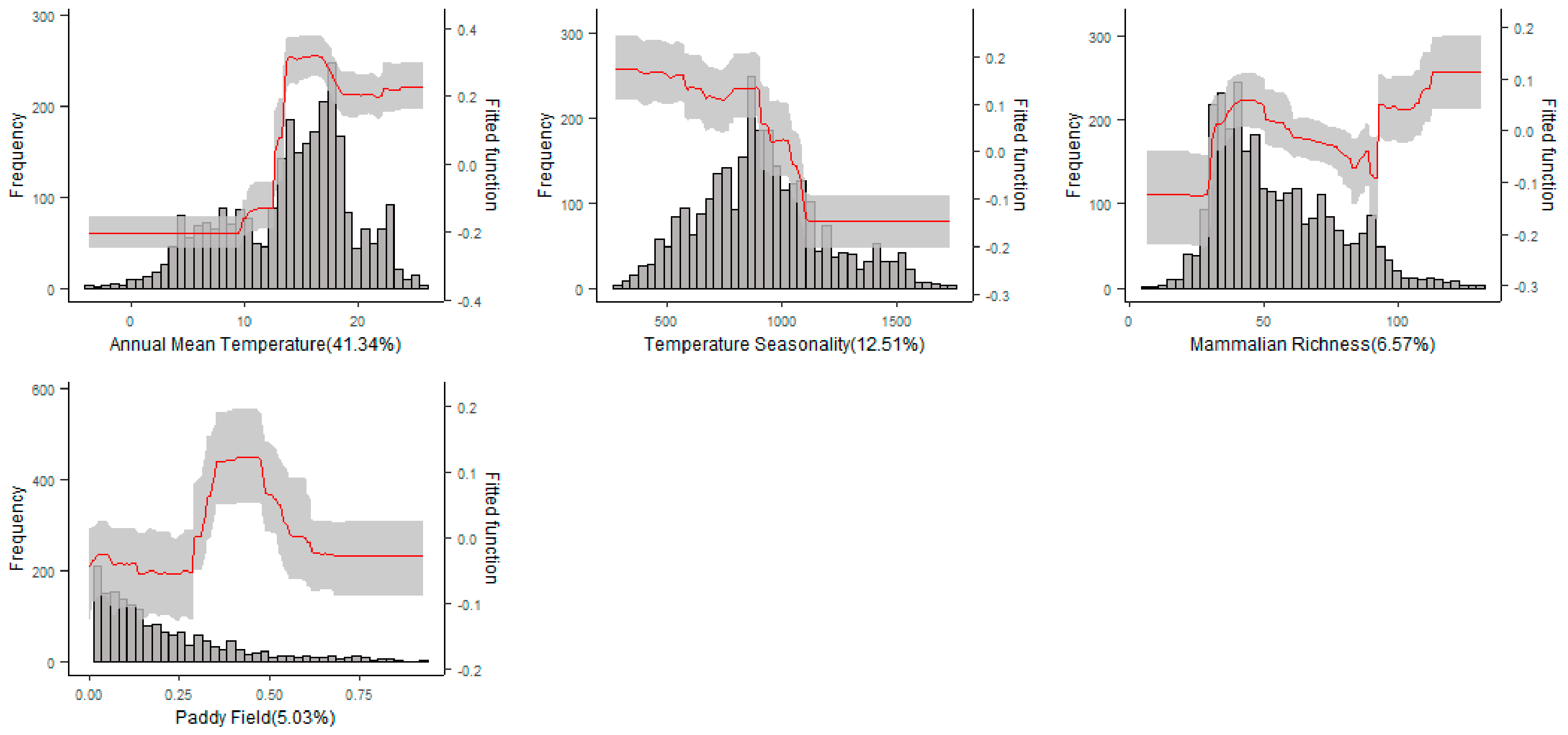

Supplementary Figure S12 The mean curves (red) and 95% percentiles (gray) for the effects of major predictors (RC≥5%) on the probability of occurrence of *Ar. subalbatus* based on the ensemble of BRT models.

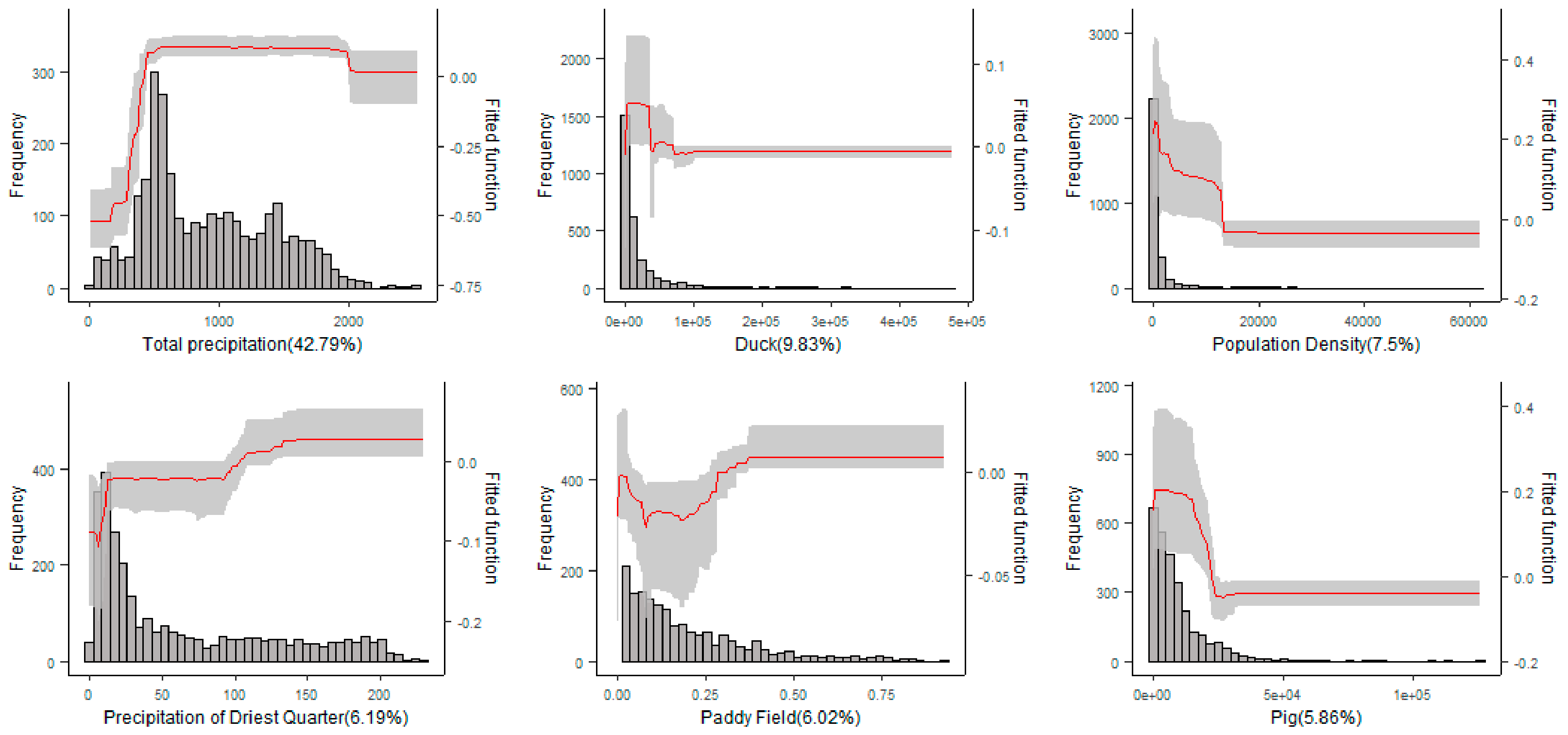

Supplementary Figure S13 The mean curves (red) and 95% percentiles (gray) for the effects of major predictors (RC≥6%) on the probability of occurrence of *An. sinensis* based on the ensemble of BRT models.

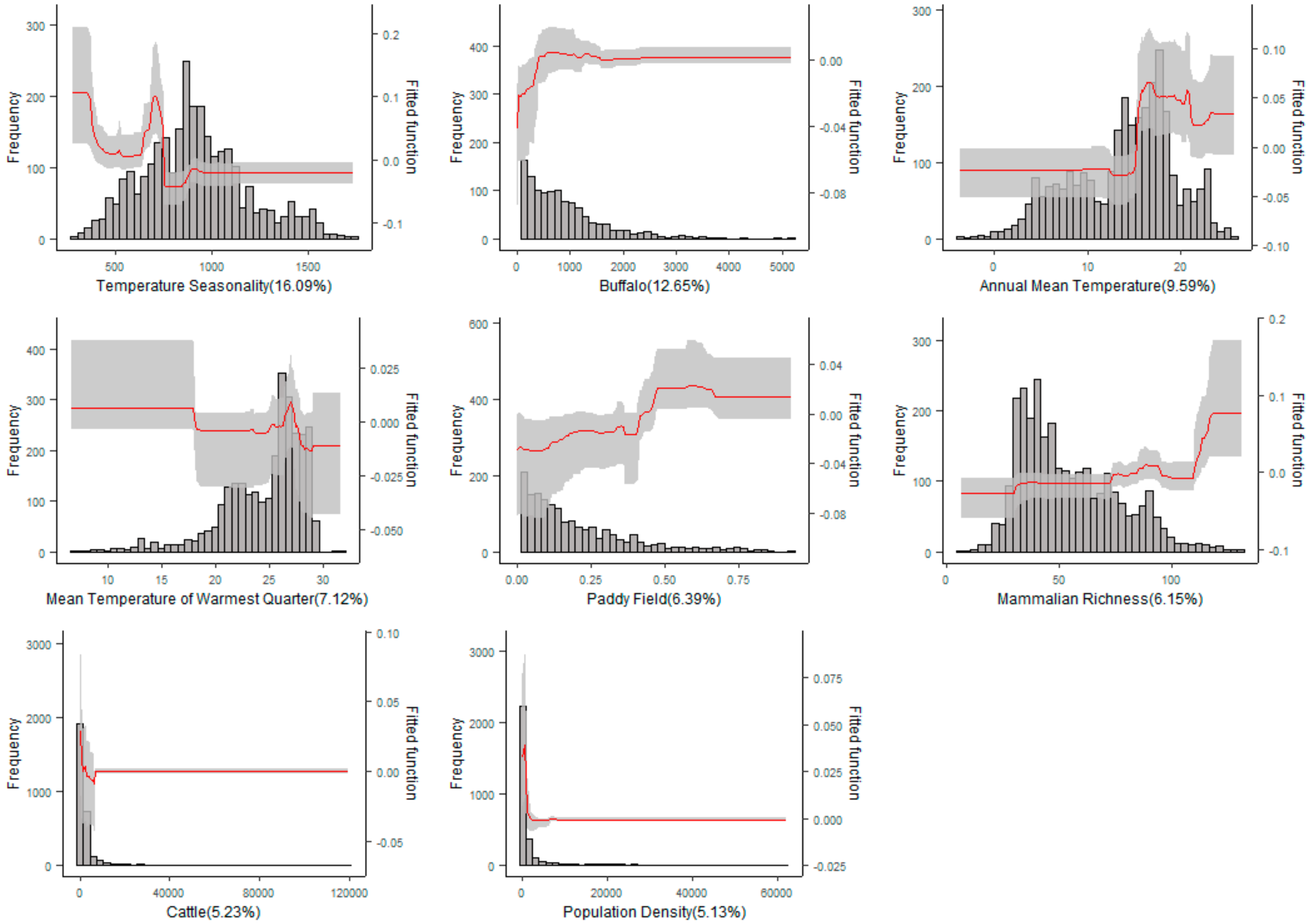

Supplementary Figure S14 The mean curves (red) and 95% percentiles (gray) for the effects of major predictors (RC≥7%) on the probability of occurrence of *An. anthropophagus* based on the ensemble of BRT models.

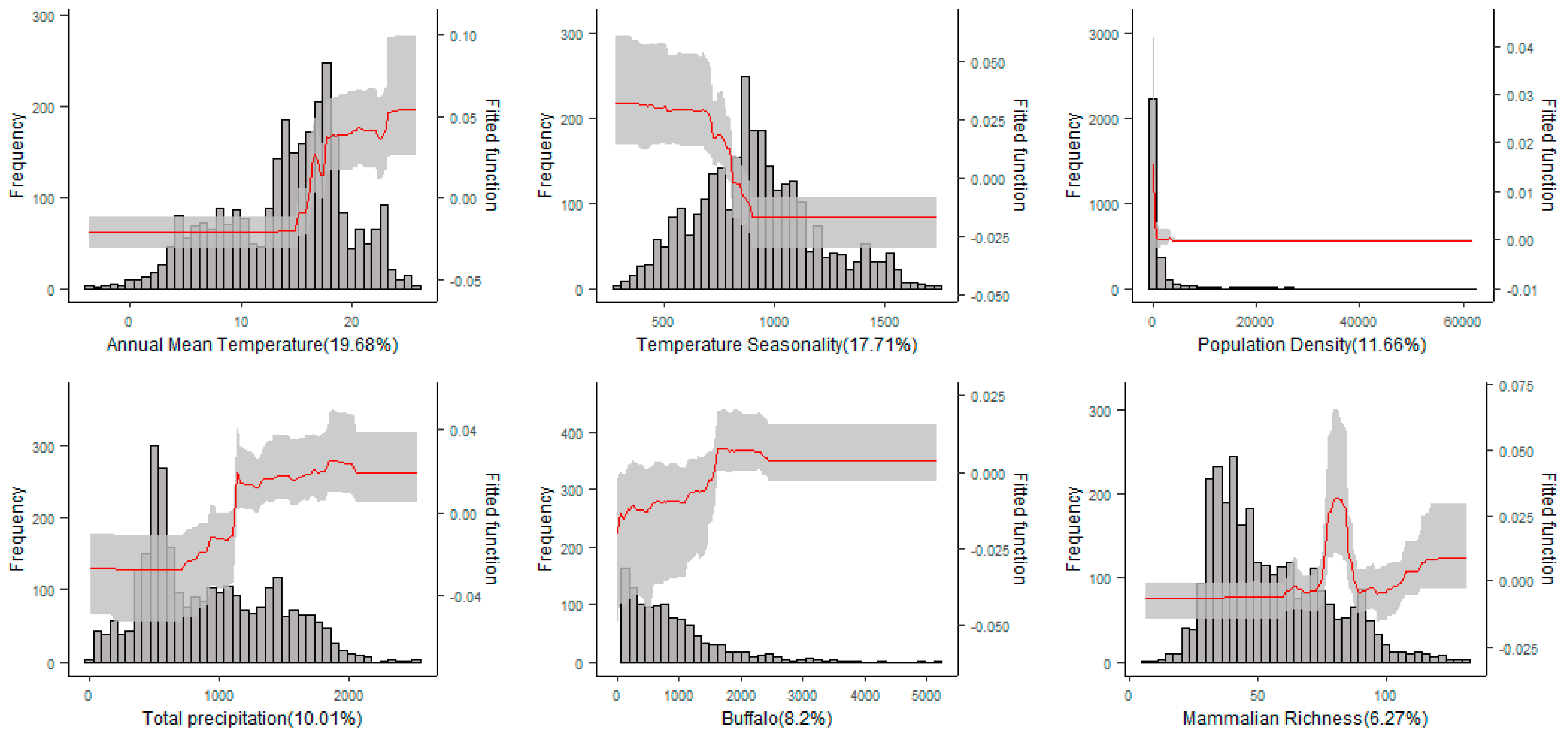

Supplementary Figure S15 The mean curves (red) and 95% percentiles (gray) for the effects of major predictors (RC≥8%) on the probability of occurrence of *An. minimus* based on the ensemble of BRT models.

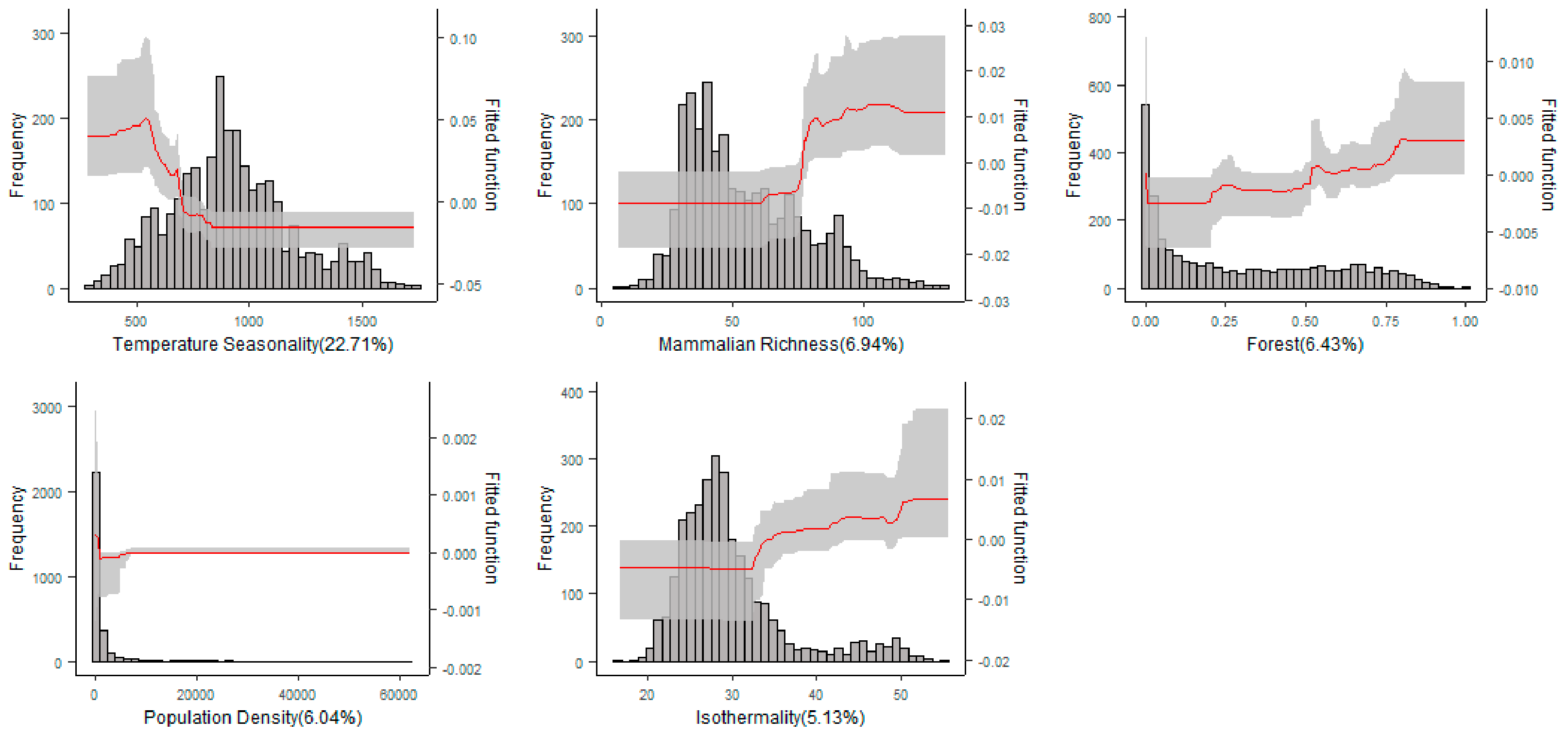

Supplementary Figure S16 The mean curves (red) and 95% percentiles (gray) for the effects of major predictors (RC≥9%) on the probability of occurrence of *An. maculatus* based on the ensemble of BRT models.

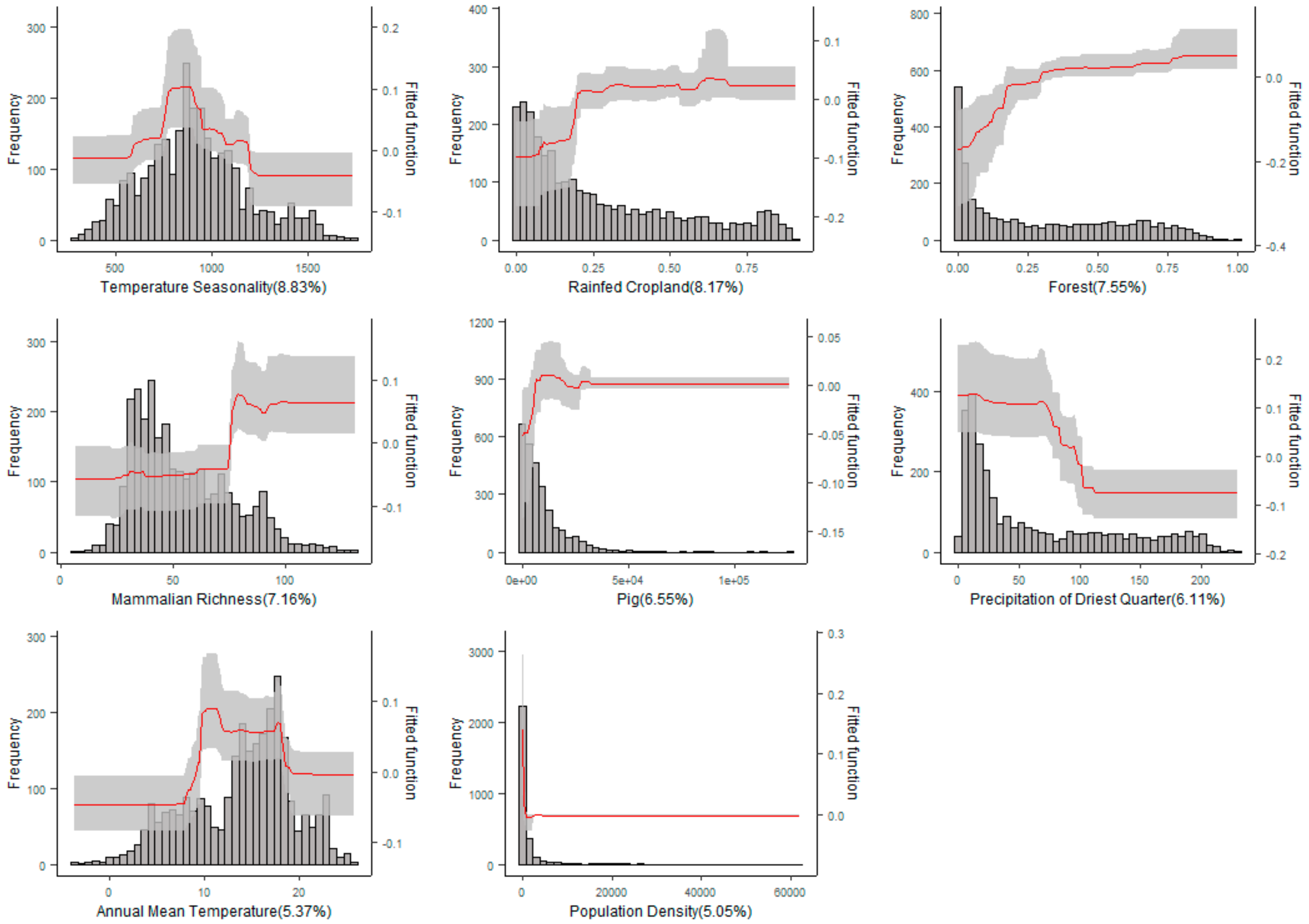

Supplementary Figure S17 The mean curves (red) and 95% percentiles (gray) for the effects of major predictors (RC≥10%) on the probability of occurrence of *An. pattoni* based on the ensemble of BRT models.

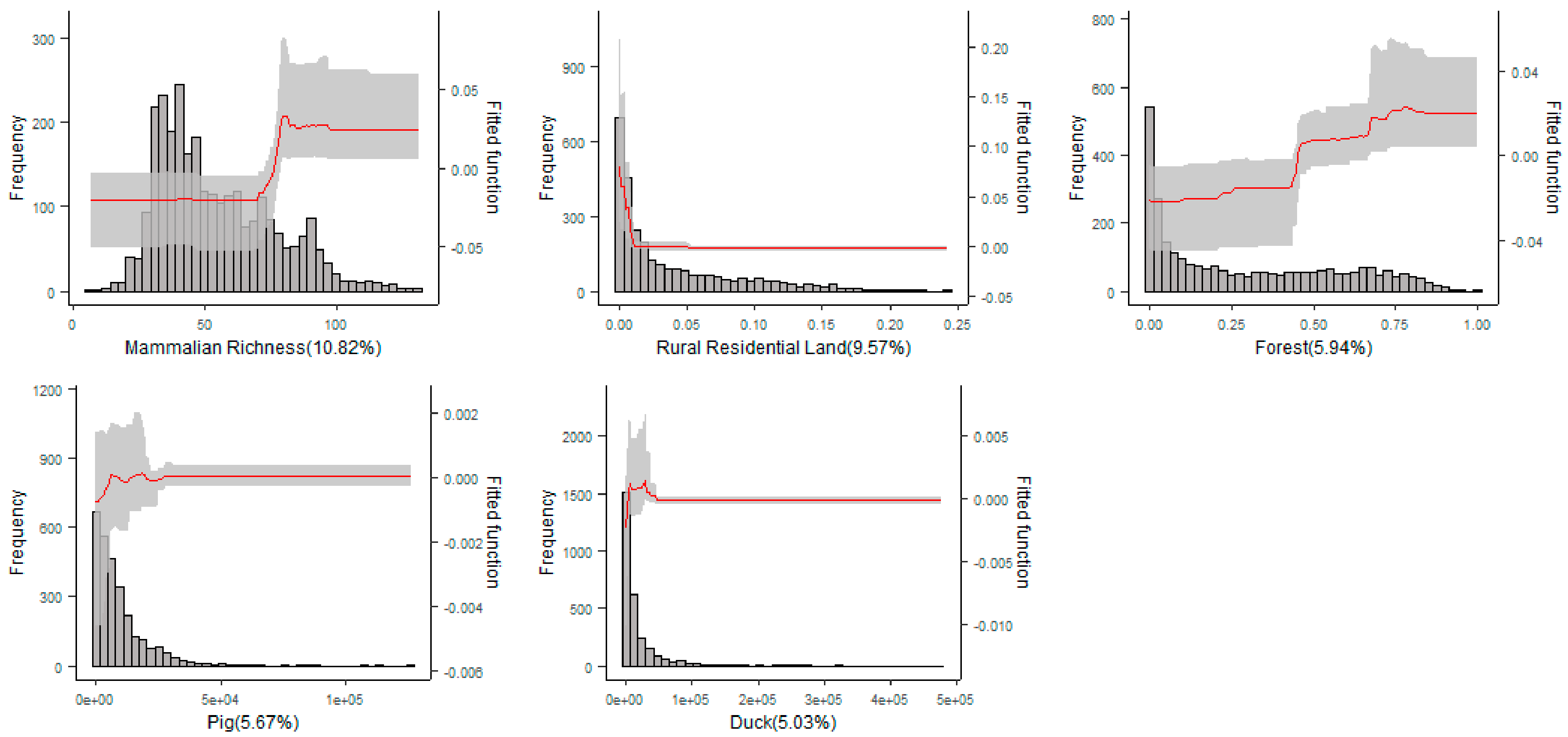

Supplementary Figure S18 The mean curves (red) and 95% percentiles (gray) for the effects of major predictors (RC≥11%) on the probability of occurrence of *An. lindesayi* based on the ensemble of BRT models.

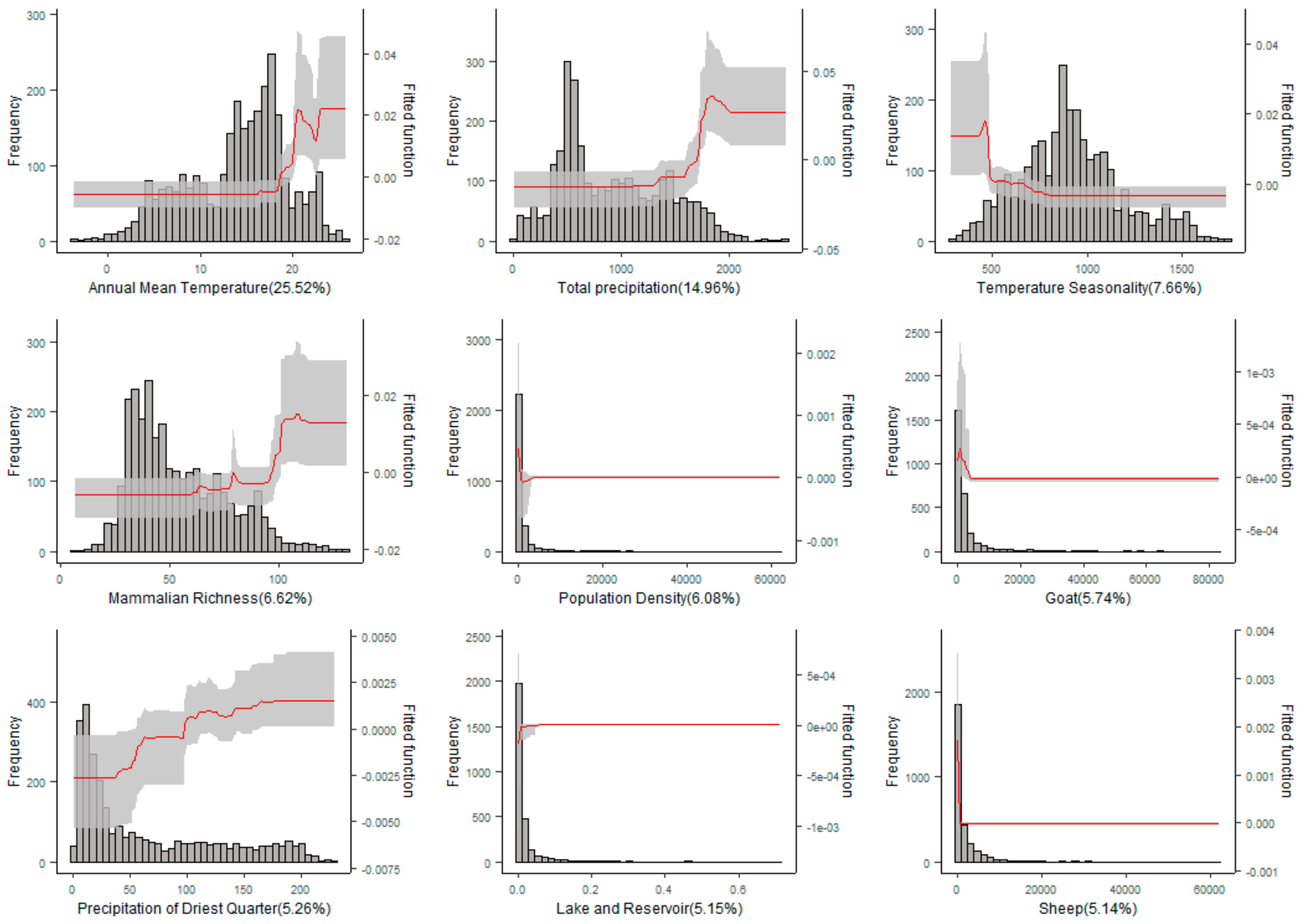

Supplementary Figure S19 The mean curves (red) and 95% percentiles (gray) for the effects of major predictors (RC≥12%) on the probability of occurrence of *An. jeyporiensis* based on the ensemble of BRT models.

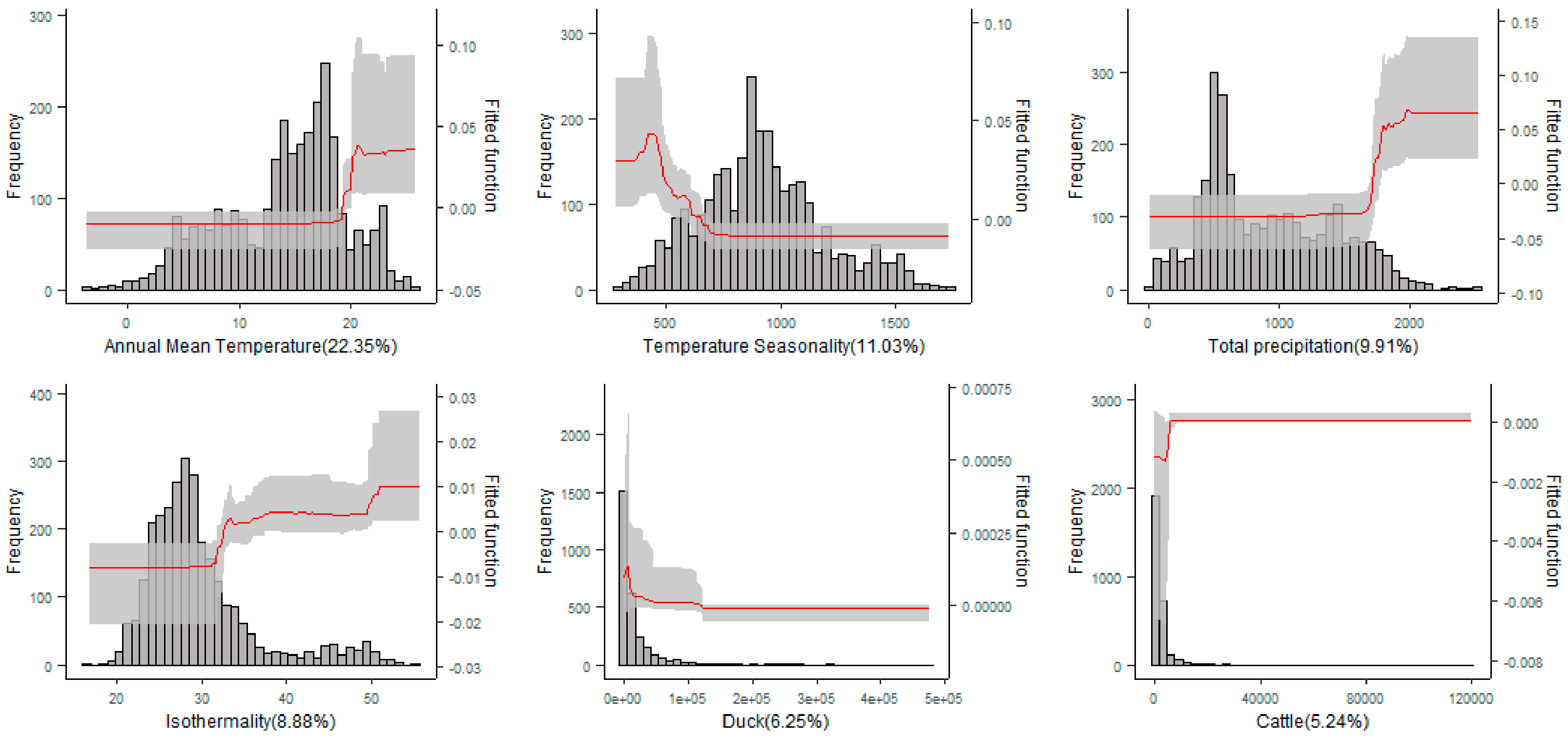

Supplementary Figure S20 The mean curves (red) and 95% percentiles (gray) for the effects of major predictors (RC≥13%) on the probability of occurrence of *An. tessellatus* based on the ensemble of BRT models.

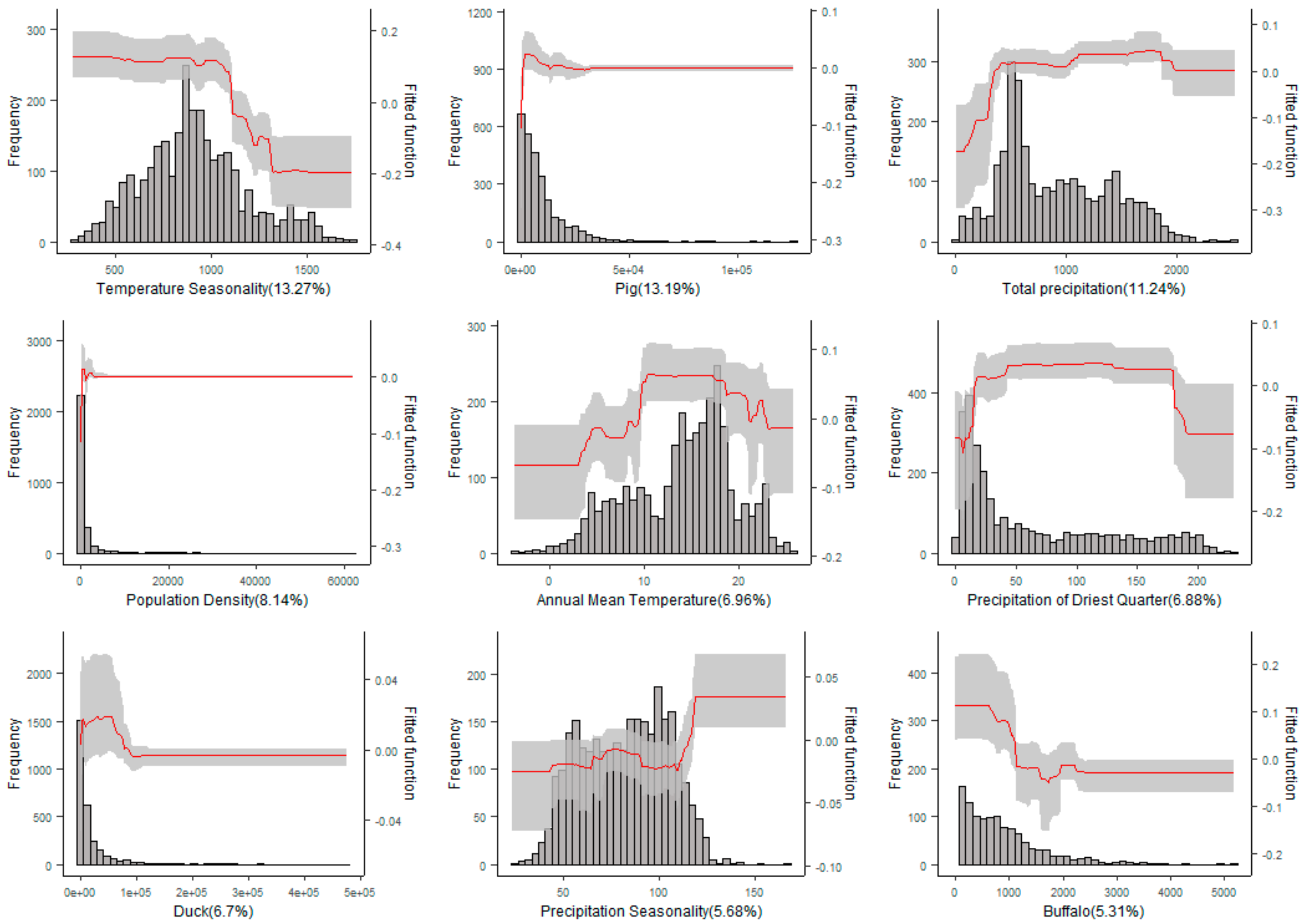

Supplementary Figure S21 The mean curves (red) and 95% percentiles (gray) for the effects of major predictors (RC≥14%) on the probability of occurrence of *Cx. tritaeniorhynchus* based on the ensemble of BRT models.

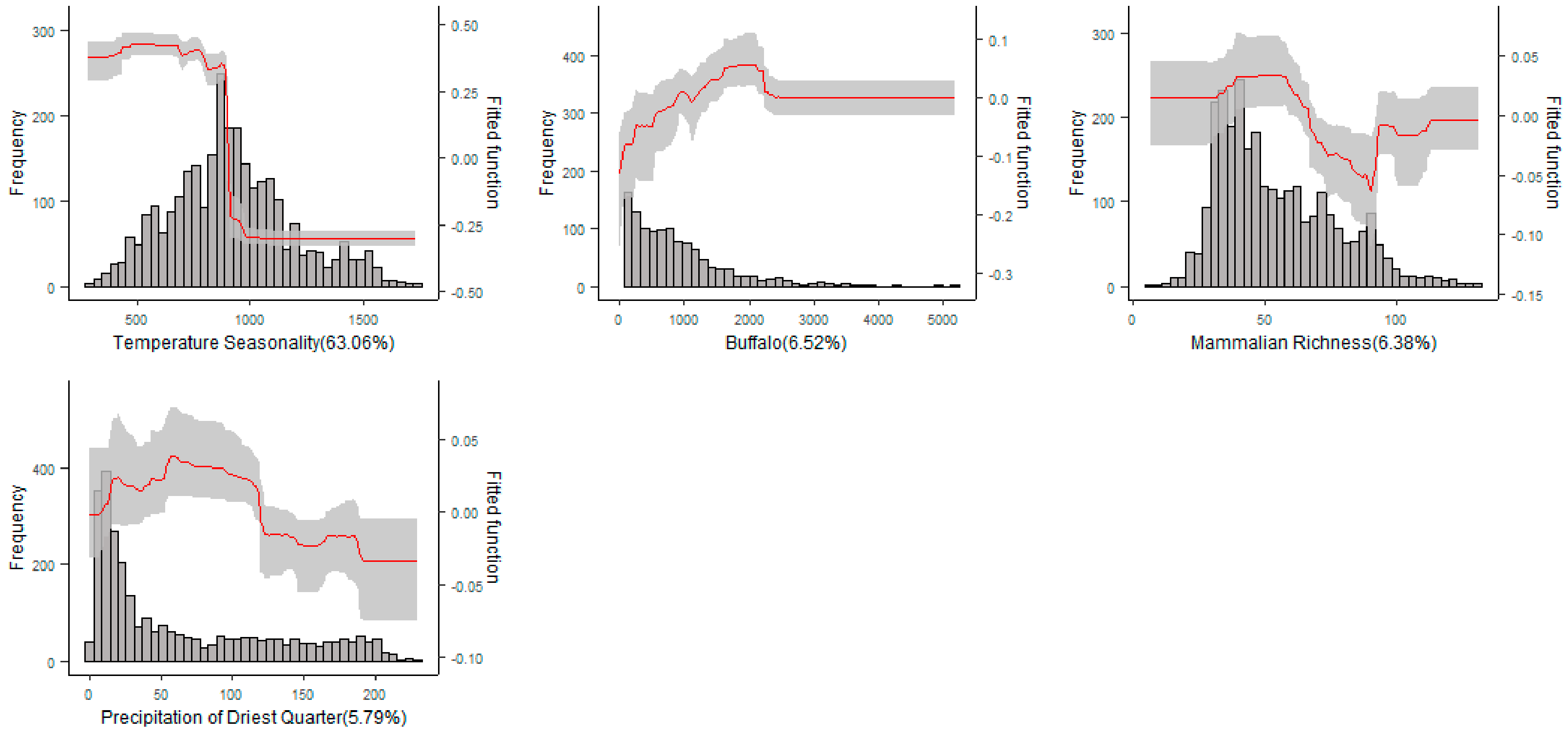

Supplementary Figure S22 The mean curves (red) and 95% percentiles (gray) for the effects of major predictors (RC≥15%) on the probability of occurrence of *Cx. pipiens quinquefasciatus* based on the ensemble of BRT models.

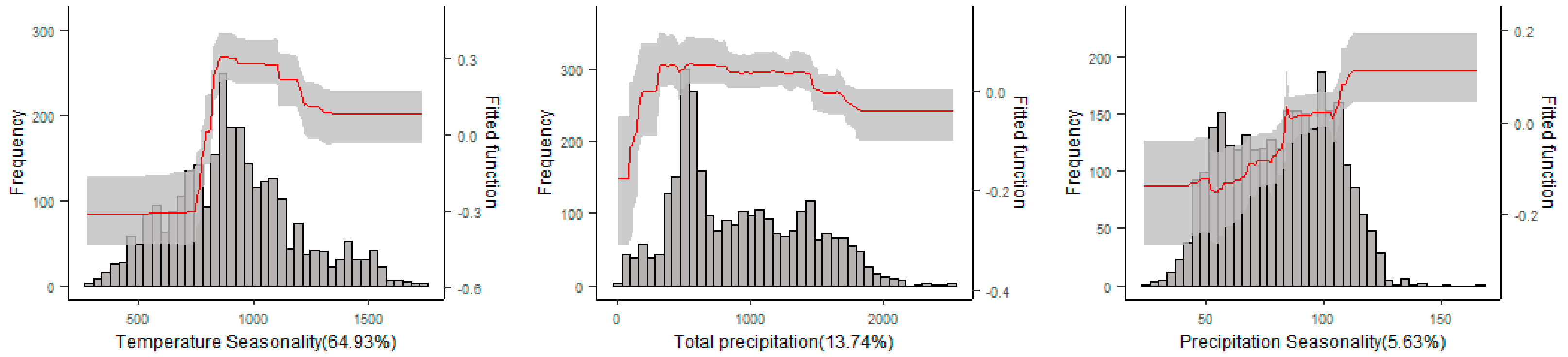

Supplementary Figure S23 The mean curves (red) and 95% percentiles (gray) for the effects of major predictors (RC≥16%) on the probability of occurrence of *Cx. pipiens pallens* based on the ensemble of BRT models.

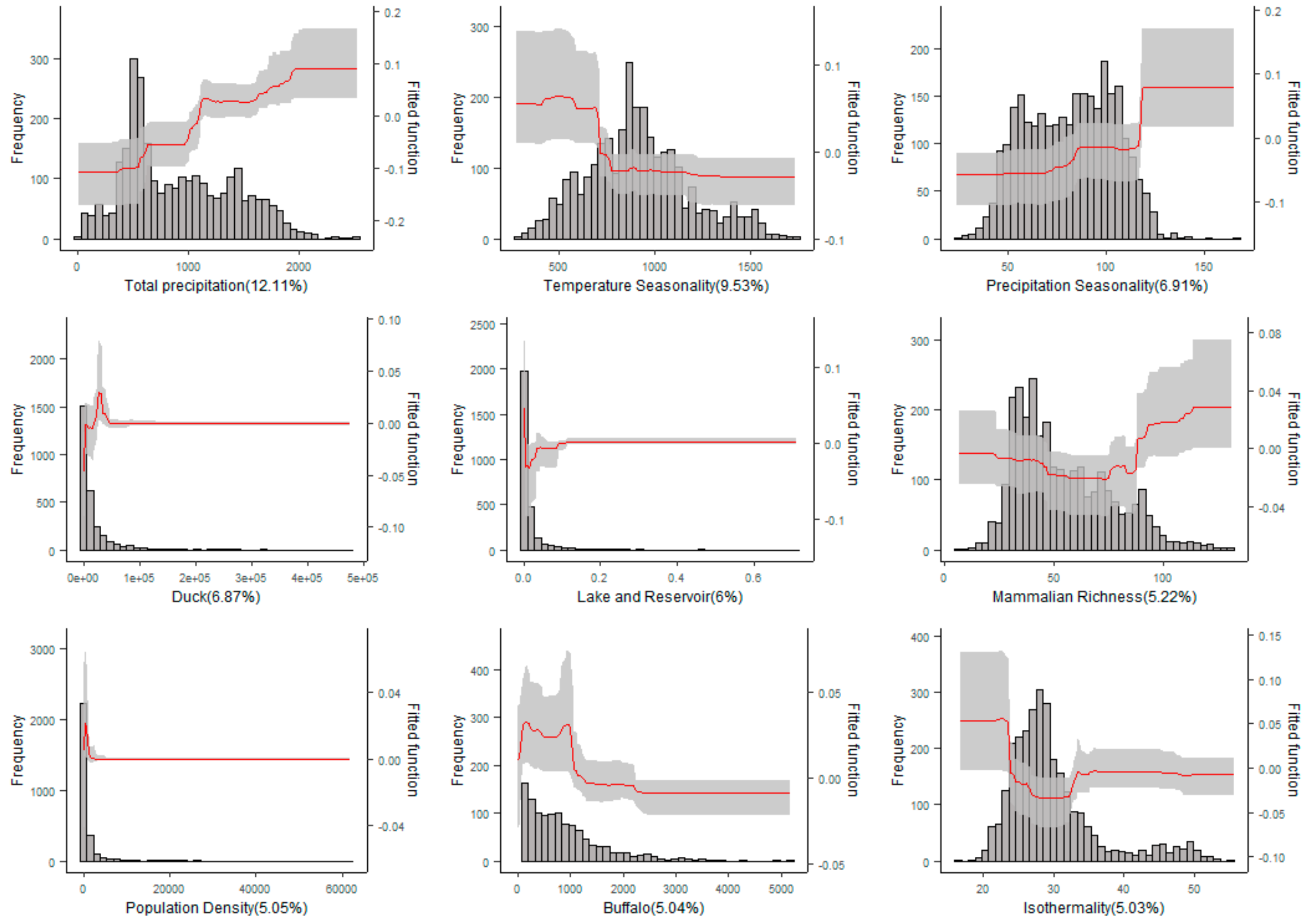

Supplementary Figure S24 The mean curves (red) and 95% percentiles (gray) for the effects of major predictors (RC≥17%) on the probability of occurrence of *Cx. bitaeniorhynchus* based on the ensemble of BRT models.

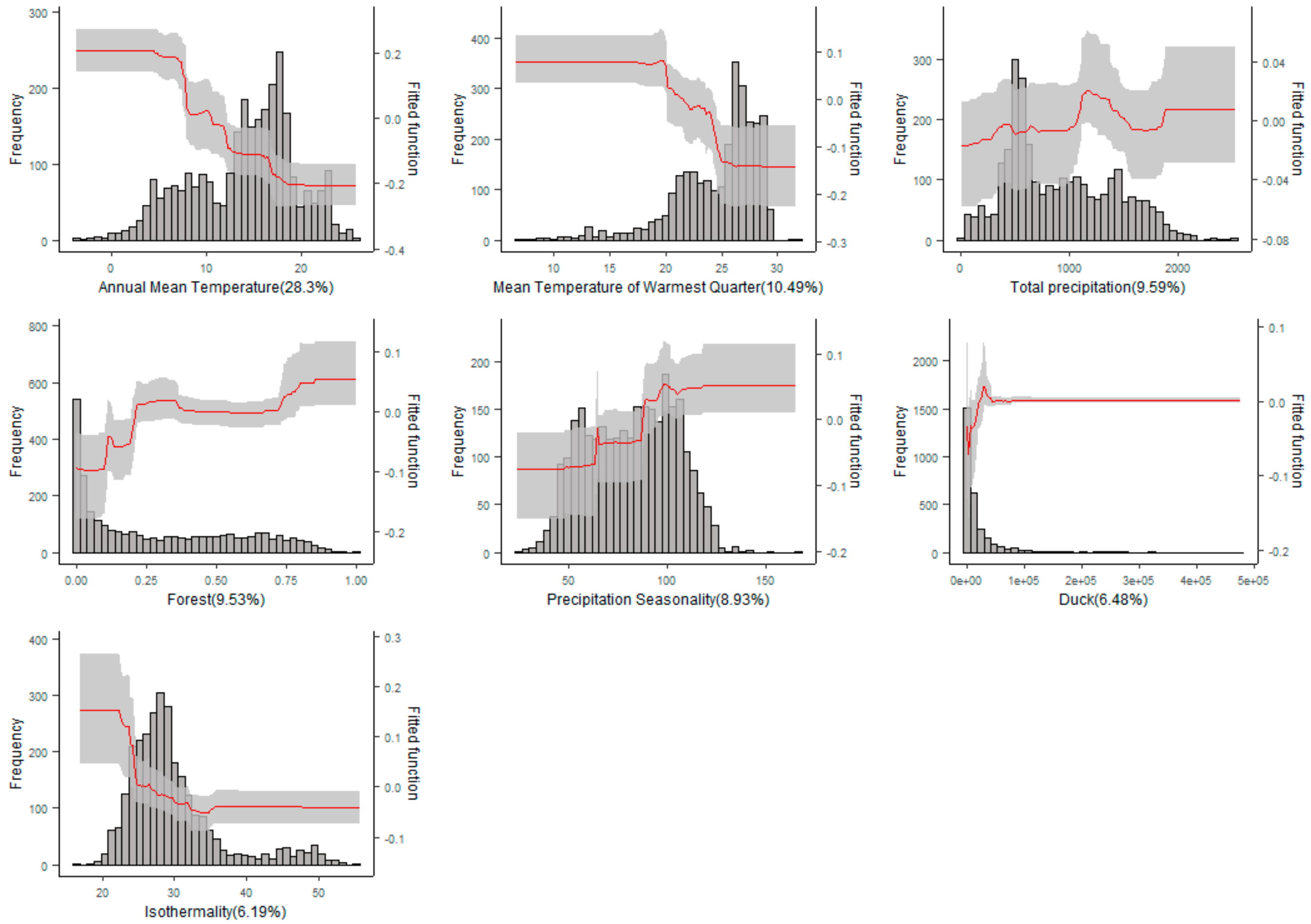

Supplementary Figure S25 The mean curves (red) and 95% percentiles (gray) for the effects of major predictors (RC≥18%) on the probability of occurrence of *Cx. vagans* based on the ensemble of BRT models.

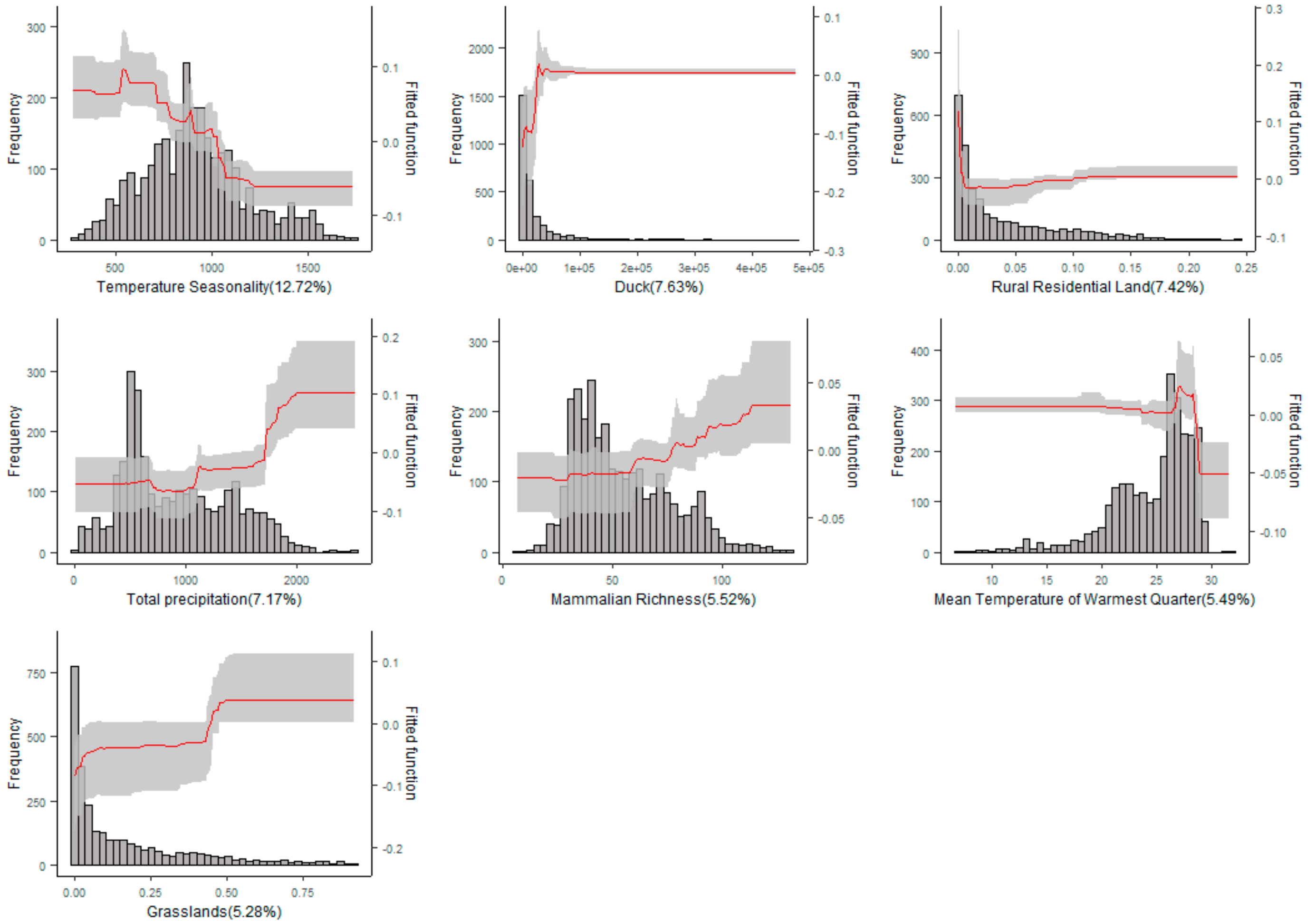

Supplementary Figure S26 The mean curves (red) and 95% percentiles (gray) for the effects of major predictors (RC≥19%) on the probability of occurrence of *Cx. halifaxia* based on the ensemble of BRT models.

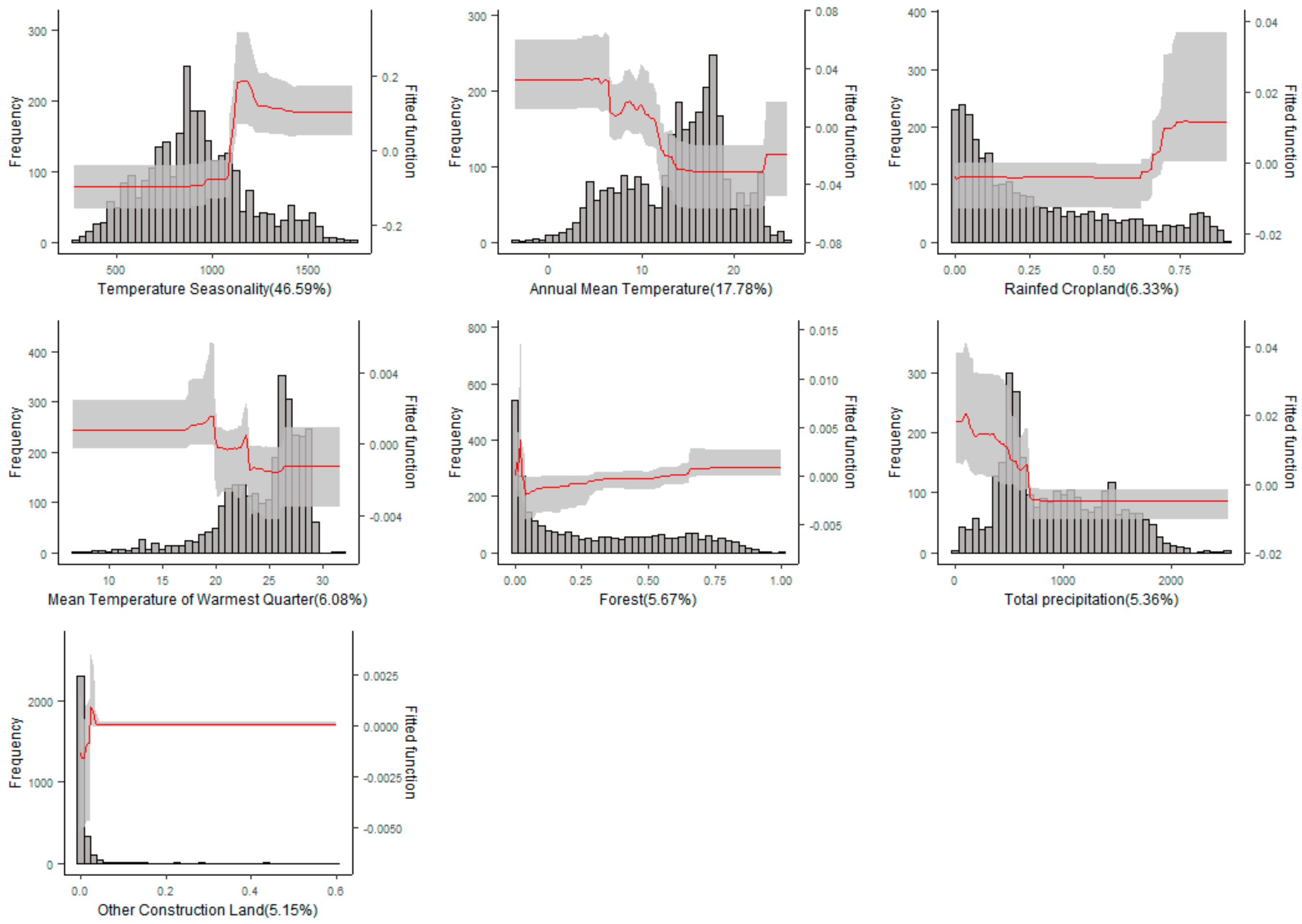

Supplementary Figure S27 The mean curves (red) and 95% percentiles (gray) for the effects of major predictors (RC≥20%) on the probability of occurrence of *Cx. modestus* based on the ensemble of BRT models.

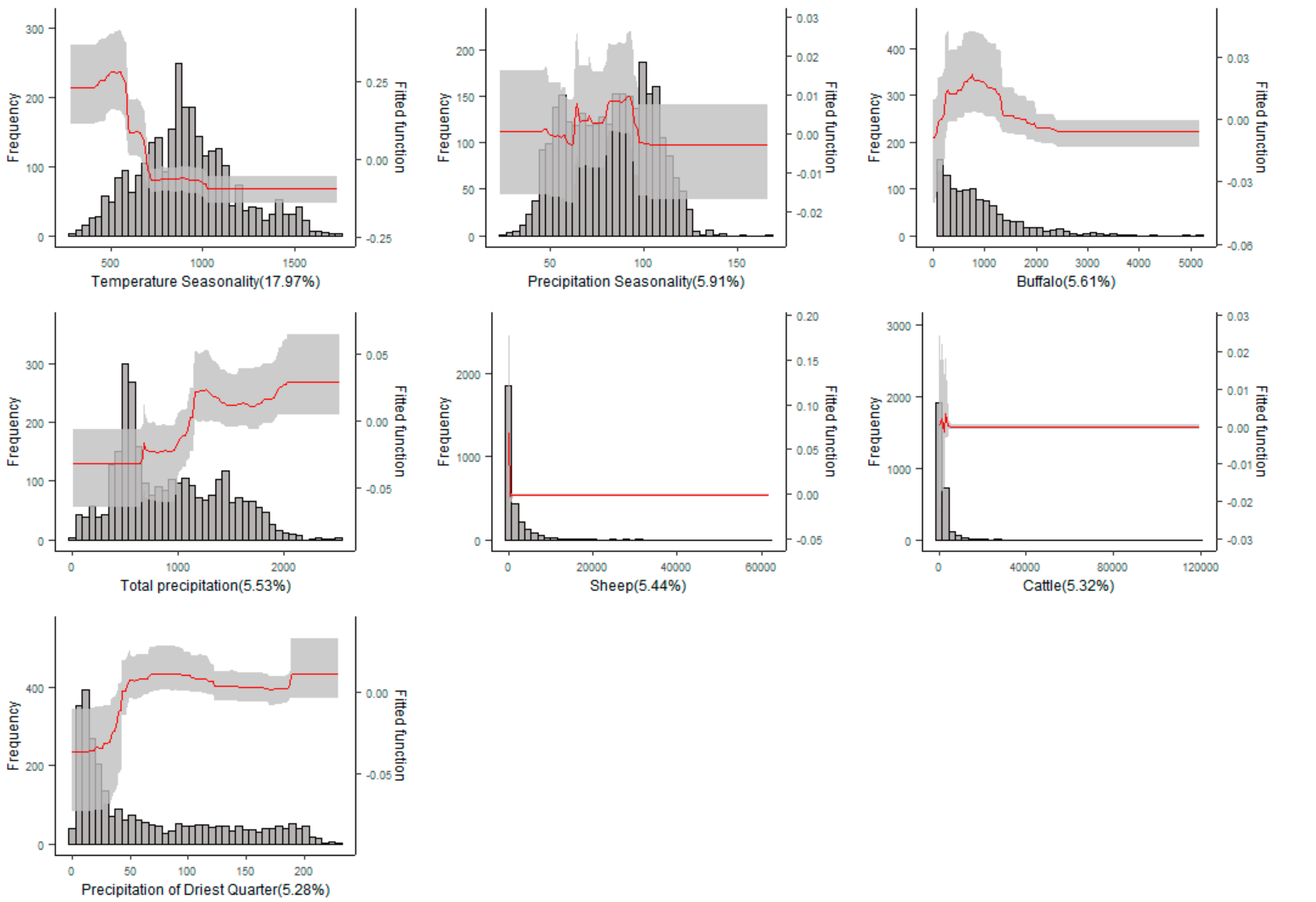

Supplementary Figure S28 The mean curves (red) and 95% percentiles (gray) for the effects of major predictors (RC≥21%) on the probability of occurrence of *Cx. fuscanus* based on the ensemble of BRT models.

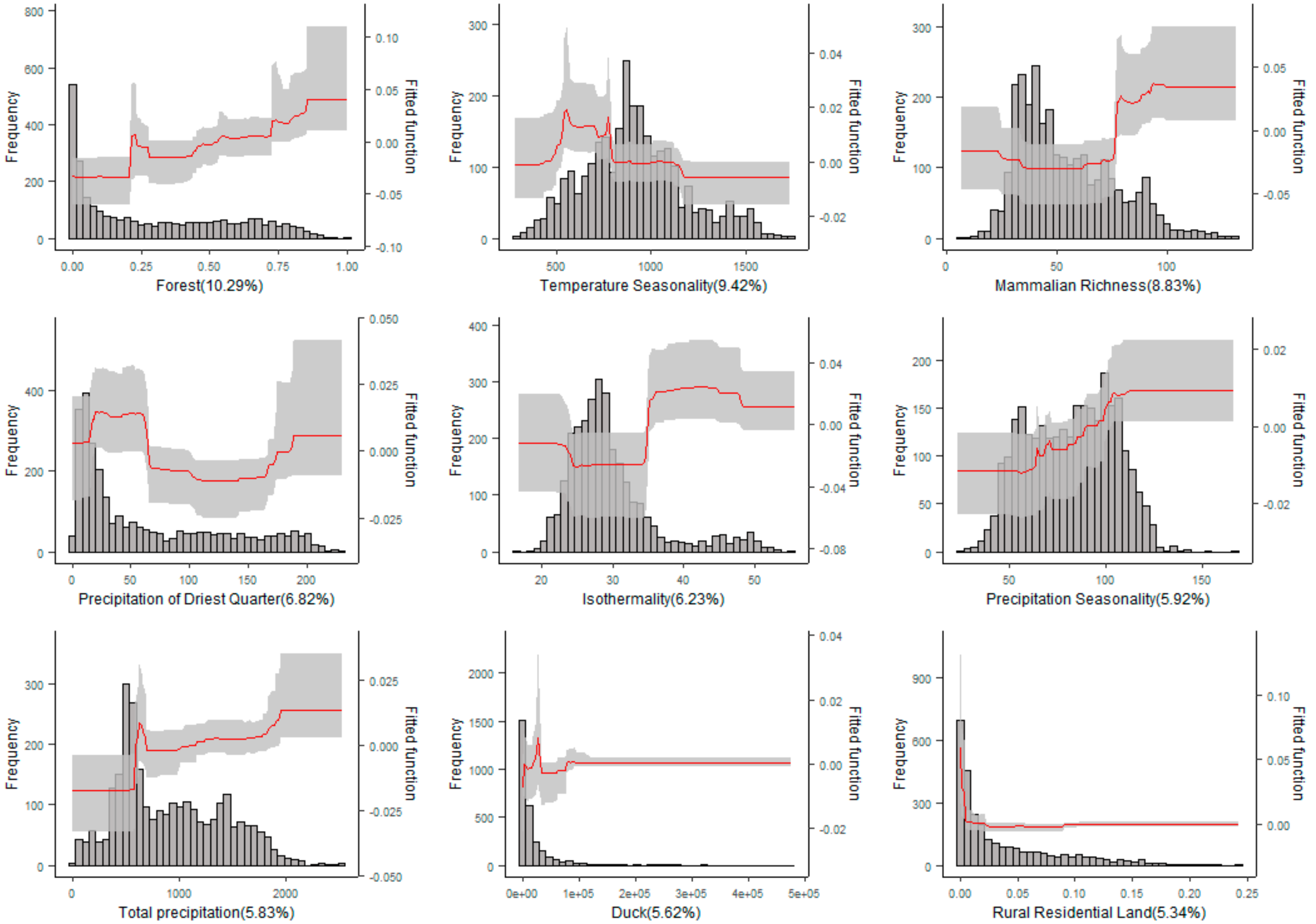

Supplementary Figure S29 The mean curves (red) and 95% percentiles (gray) for the effects of major predictors (RC≥22%) on the probability of occurrence of *Cx. mimeticus* based on the ensemble of BRT models.

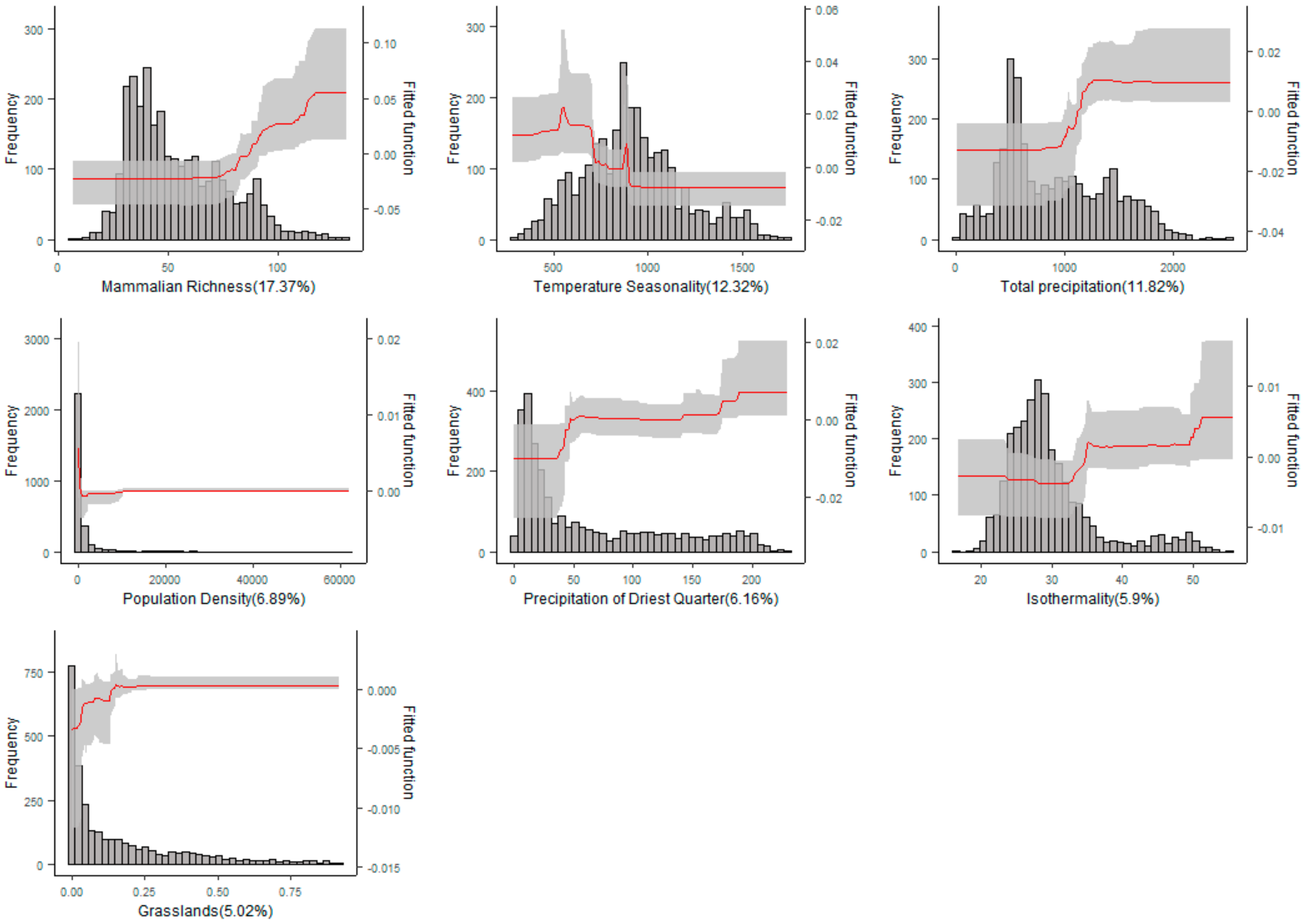

Supplementary Figure S30 The mean curves (red) and 95% percentiles (gray) for the effects of major predictors (RC≥23%) on the probability of occurrence of *Cx. pseudovishnui* based on the ensemble of BRT models.

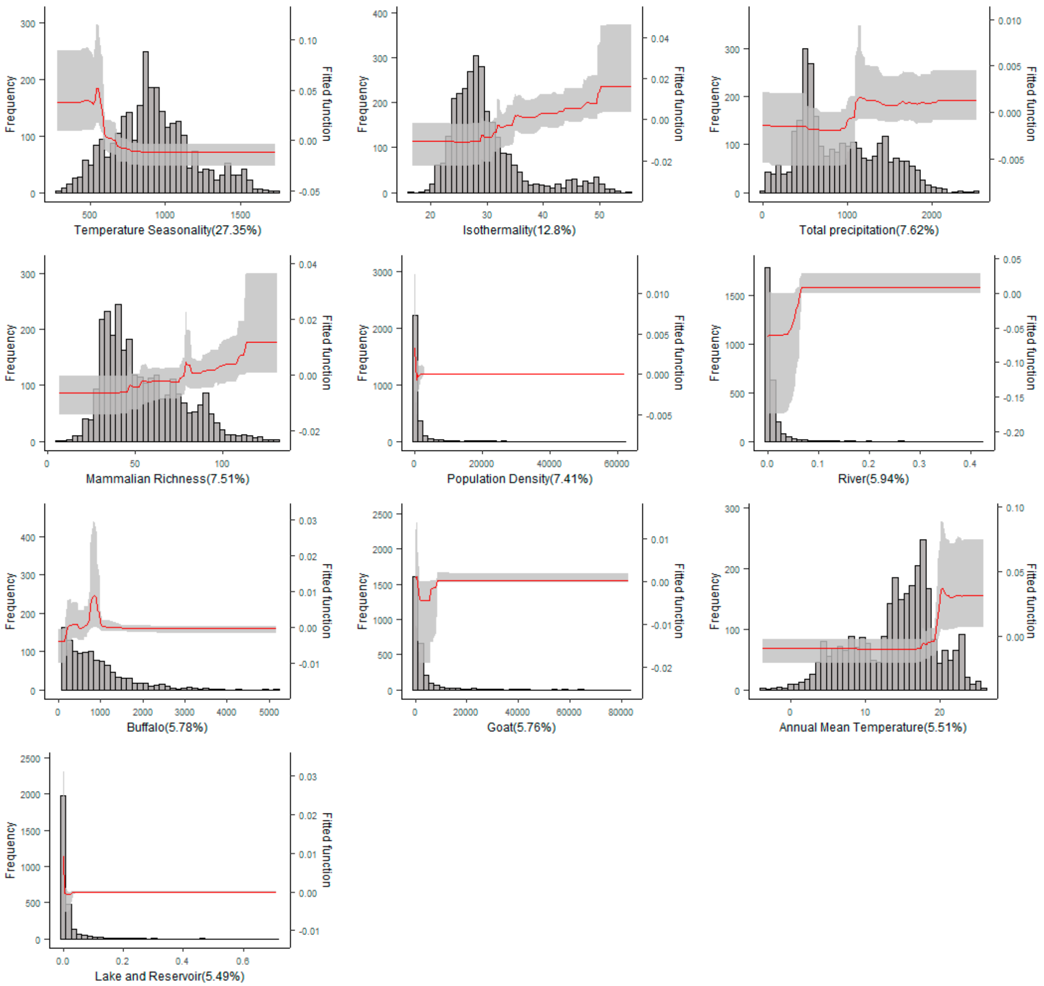

Supplementary Figure S31 The mean curves (red) and 95% percentiles (gray) for the effects of major predictors (RC≥24%) on the probability of occurrence of *Cx. fuscocephala* based on the ensemble of BRT models.

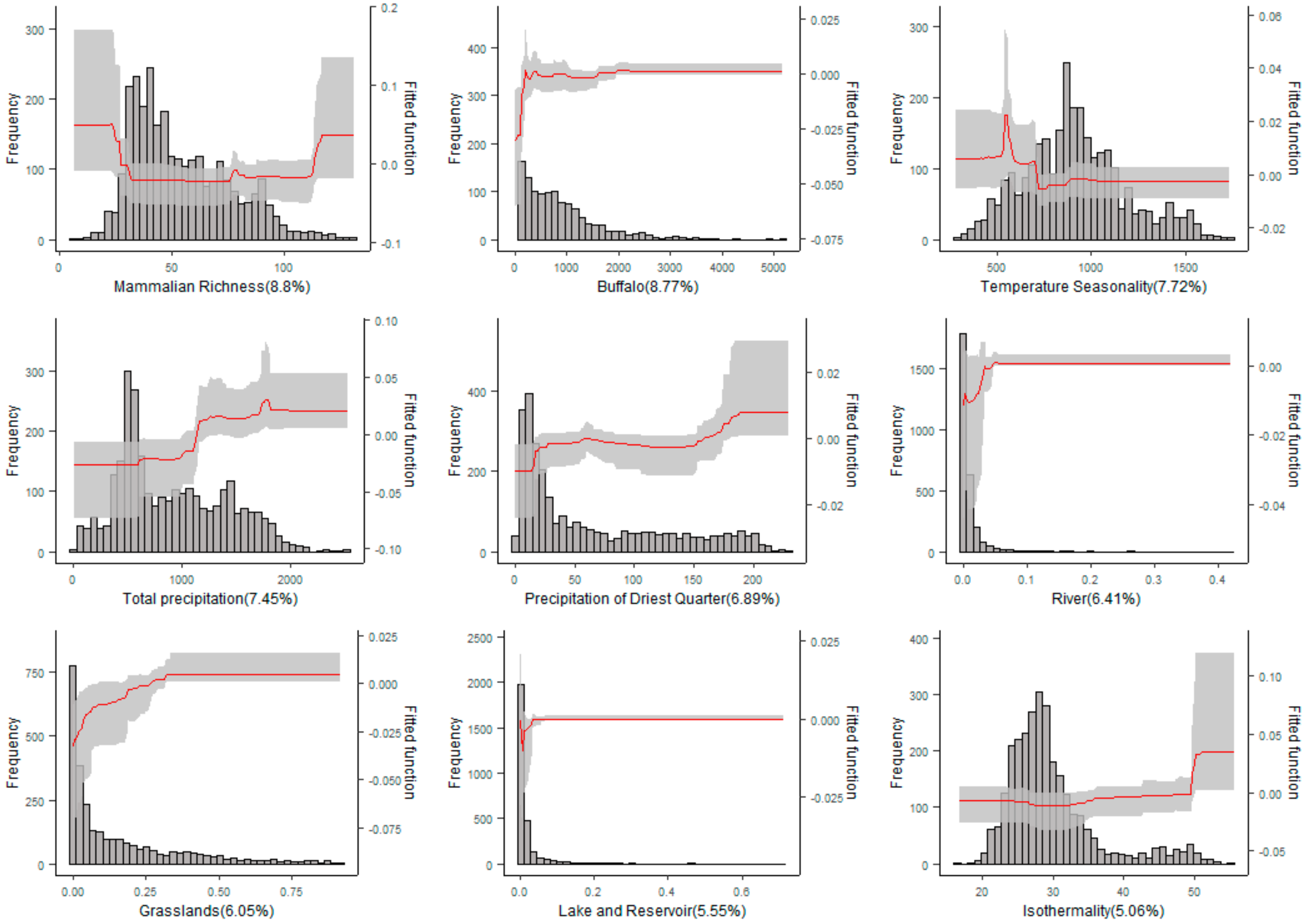

Supplementary Figure S32 The mean curves (red) and 95% percentiles (gray) for the effects of major predictors (RC≥25%) on the probability of occurrence of *Cx. whitmorei* based on the ensemble of BRT models.

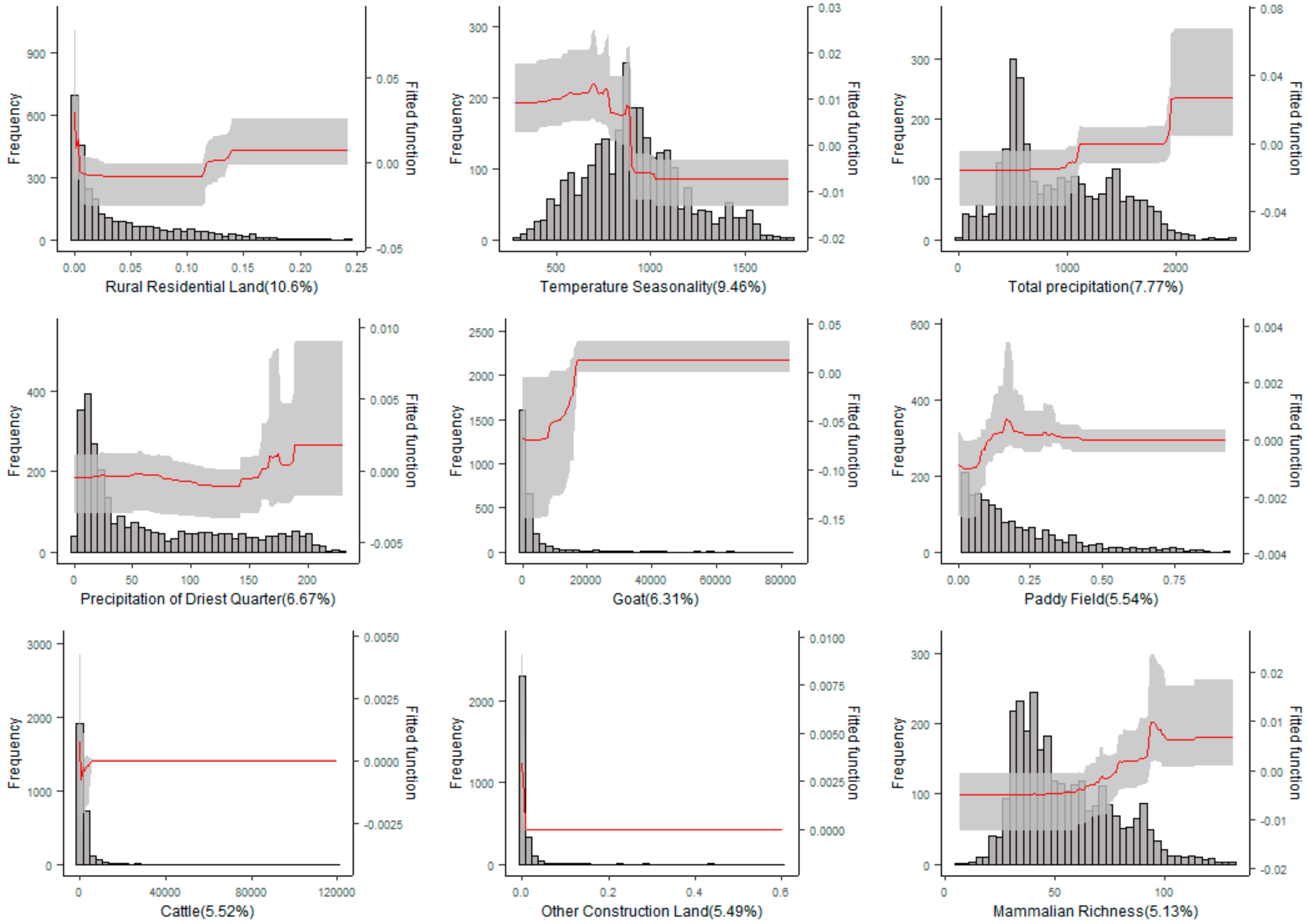

Supplementary Figure S33 The mean curves (red) and 95% percentiles (gray) for the effects of major predictors (RC≥26%) on the probability of occurrence of *Cx. mimulus* based on the ensemble of BRT models.

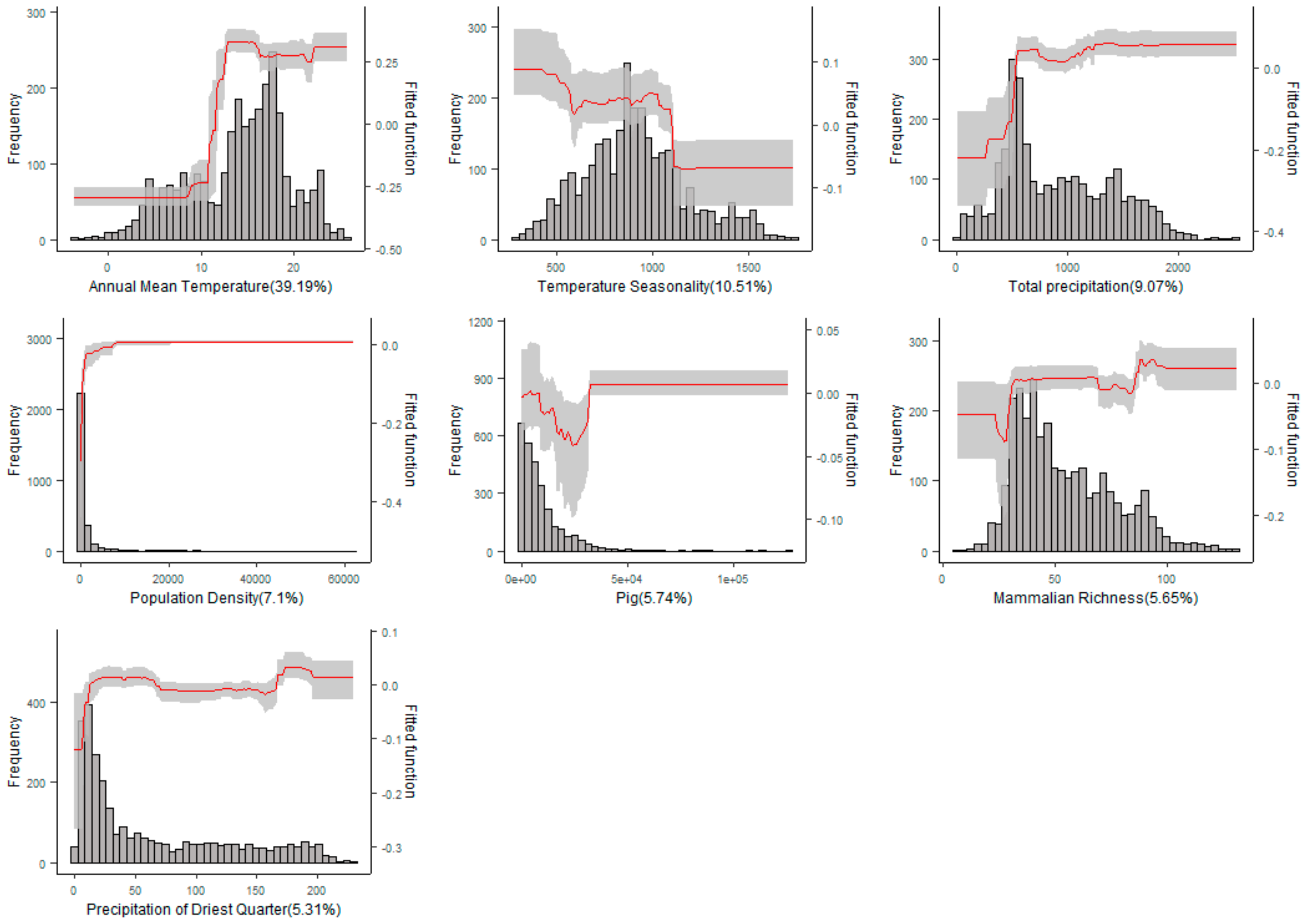

Supplementary Figure S34 The mean curves (red) and 95% percentiles (gray) for the effects of major predictors (RC≥27%) on the probability of occurrence of *Ae. albopictus* based on the ensemble of BRT models.

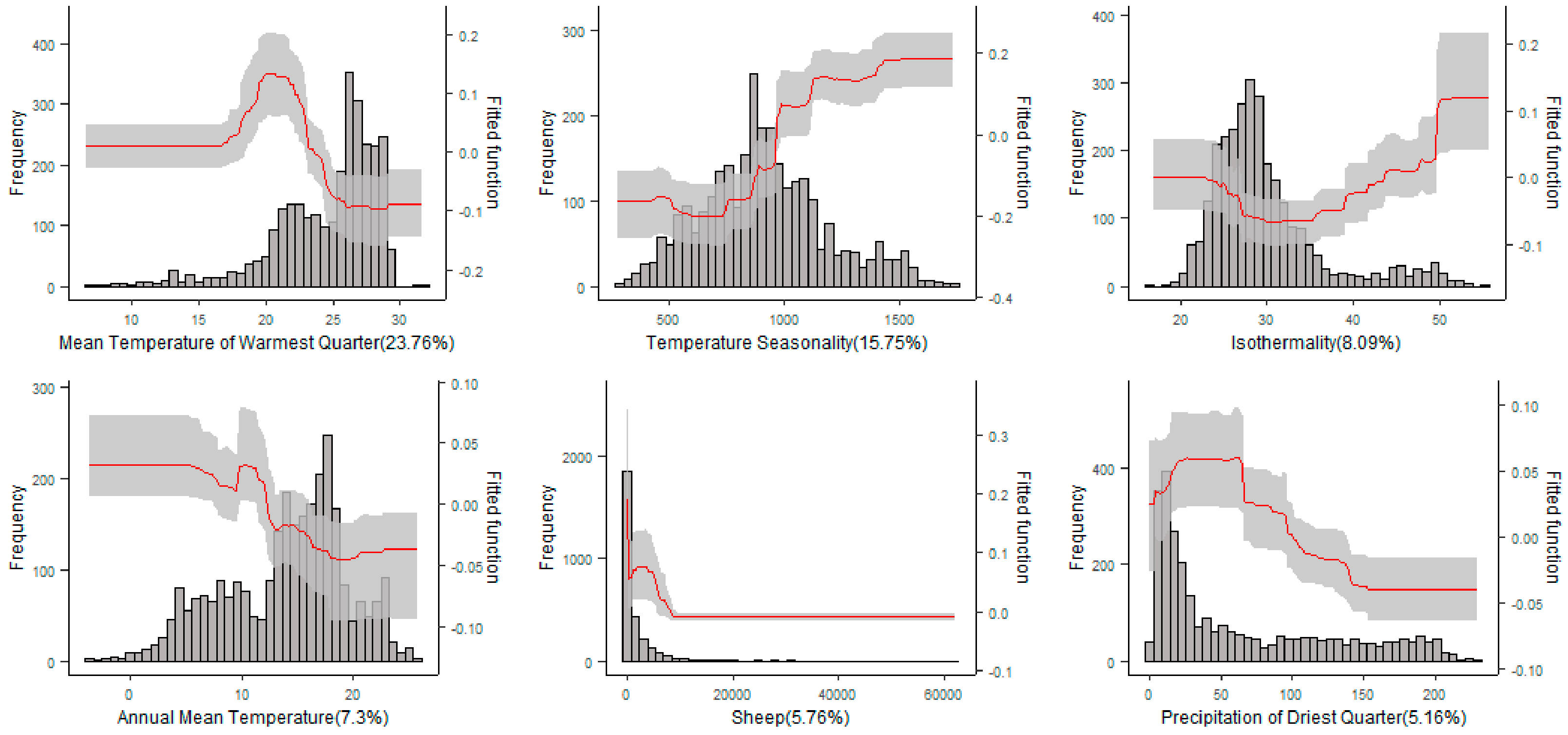

Supplementary Figure S35 The mean curves (red) and 95% percentiles (gray) for the effects of major predictors (RC≥28%) on the probability of occurrence of *Ae. vexans* based on the ensemble of BRT models.

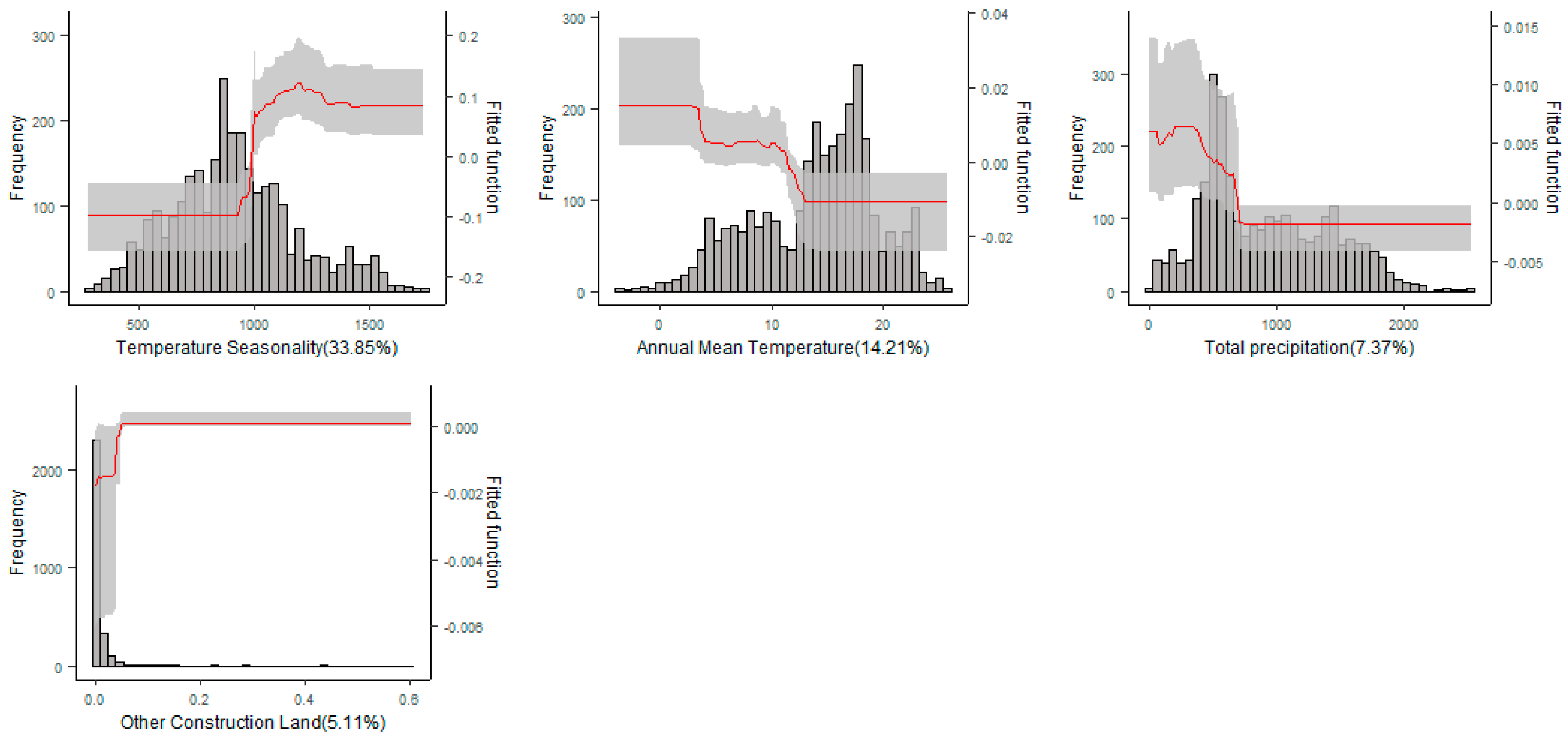

Supplementary Figure S36 The mean curves (red) and 95% percentiles (gray) for the effects of major predictors (RC≥29%) on the probability of occurrence of *Ae. dorsalis* based on the ensemble of BRT models.

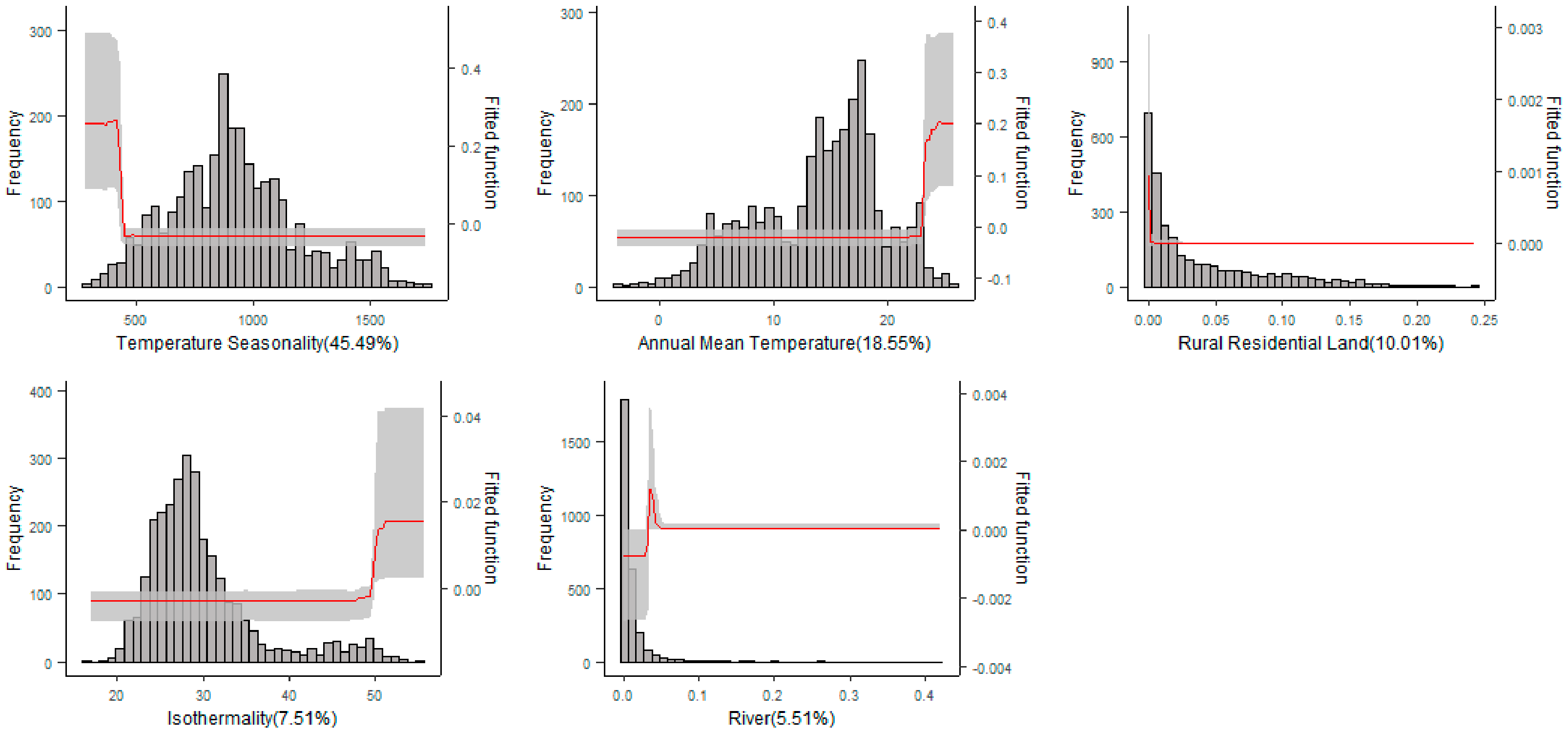

Supplementary Figure S37 The mean curves (red) and 95% percentiles (gray) for the effects of major predictors (RC≥30%) on the probability of occurrence of *Ae. aegypti* based on the ensemble of BRT models.

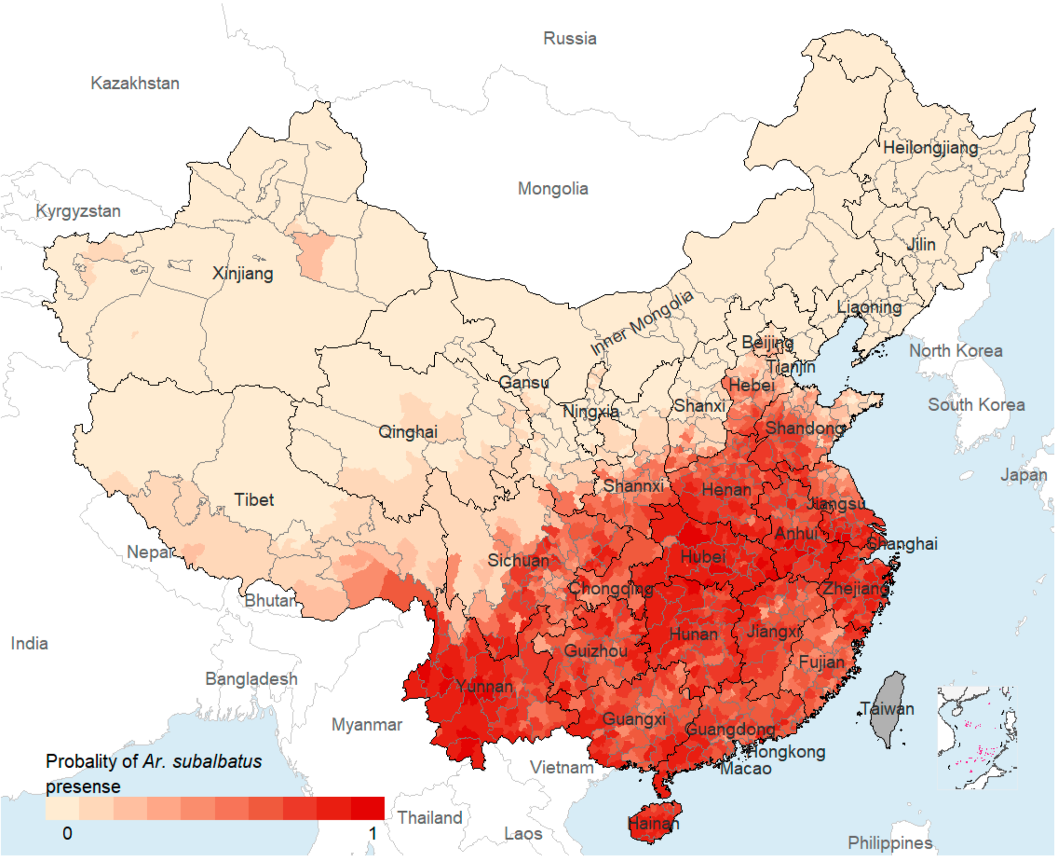

Supplementary Figure S38 The predicted county-level distributions of the one most prevalent mosquito species in the *Ar. subalbatus*, averaged over the ensemble of BRT models. Source data are provided as a Source Data file.

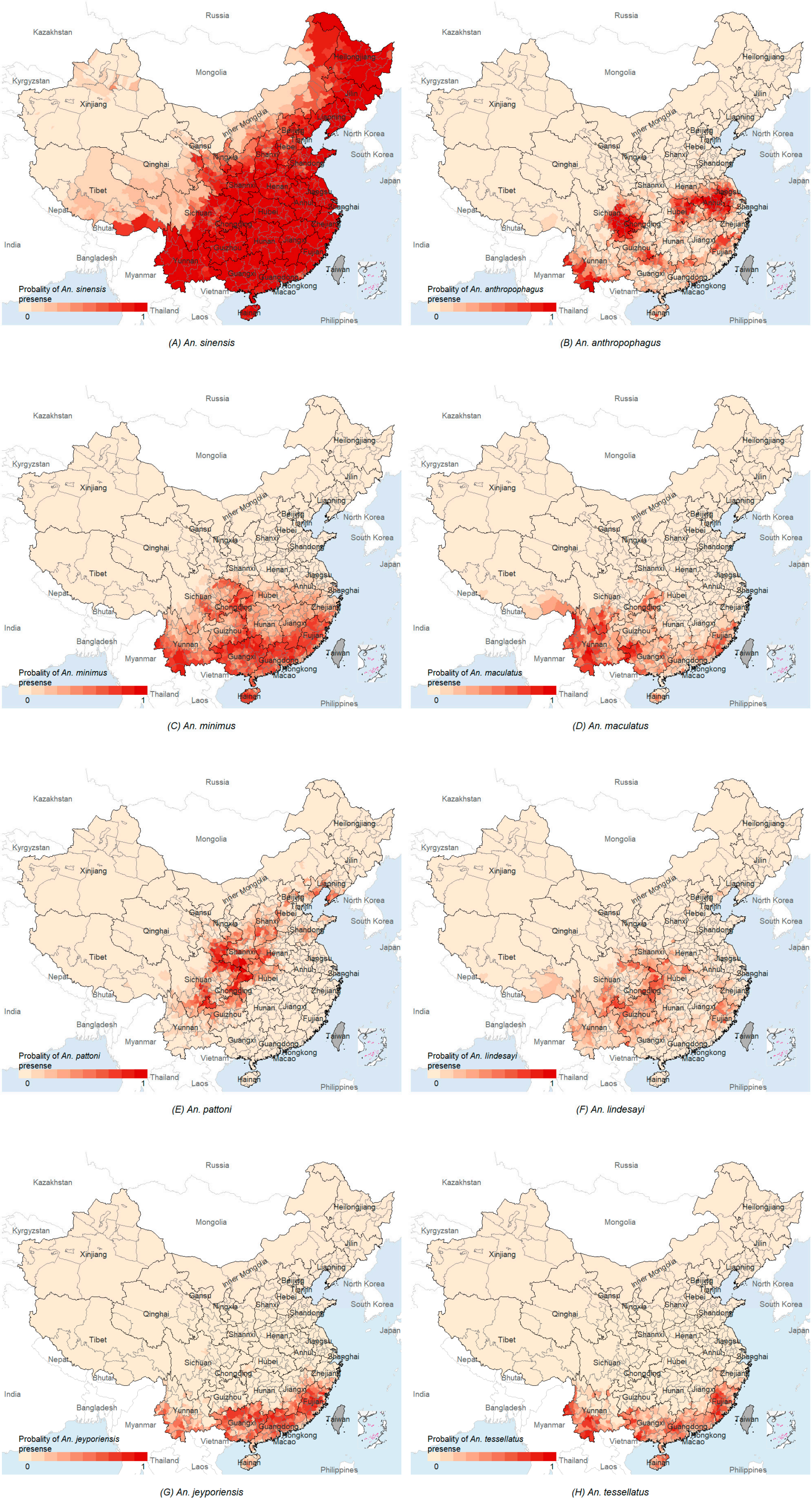

Supplementary Figure S39 The predicted county-level distributions of the eight most prevalent mosquito species in the *Anopheles* genus, averaged over the ensemble of BRT models (A) *An. sinensis,* (B) *An. anthropophagus*, (C) *An. minimus*, (D)*An. maculatus*, (E) *An. pattoni*, (F) *An. lindesayi*, (G) *An. jeyporiensis* and (H) *An. tessellatus*. Source data are provided as a Source Data

file.

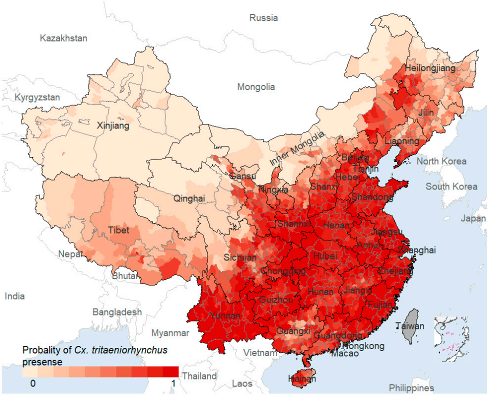

(A) *Cx. tritaeniorhynchus*

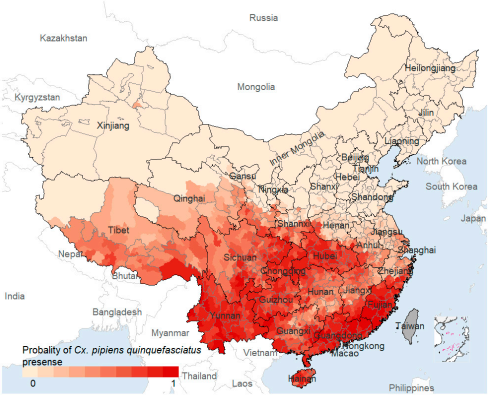

(B) *Cx. pipiens quinquefasciatus*

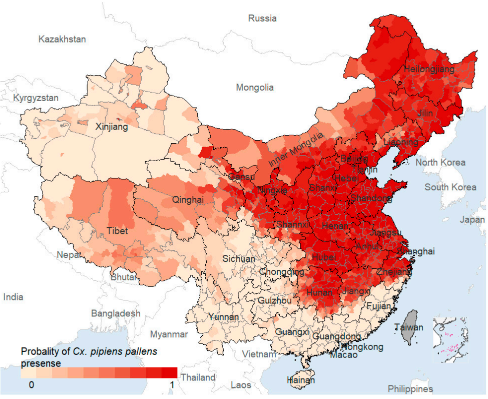

(C) *Cx. pipiens pallens*

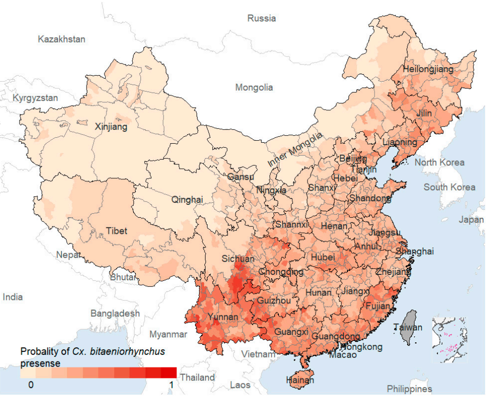

(D) *Cx. bitaeniorhynchus*

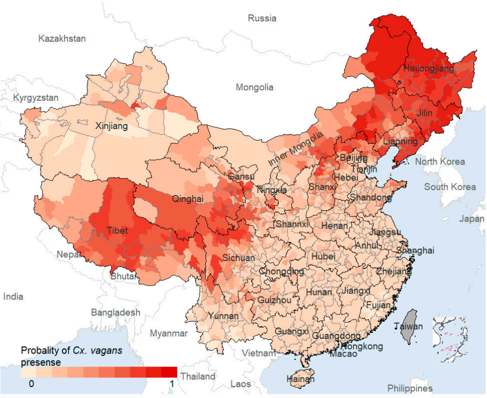

(E) *Cx. vagans*

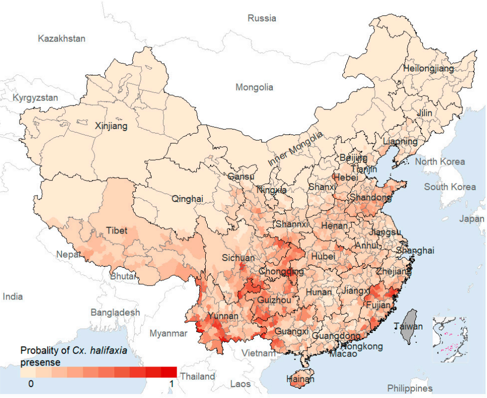

(F) *Cx. halifaxia*

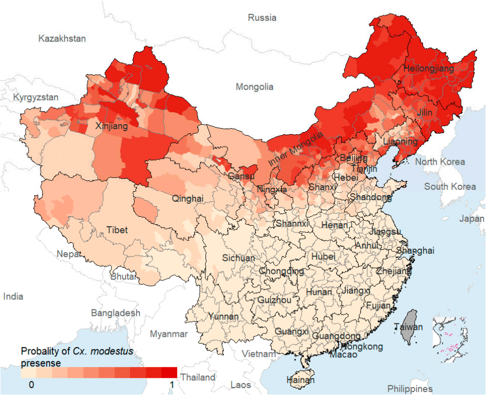

(G) *Cx. modestus*

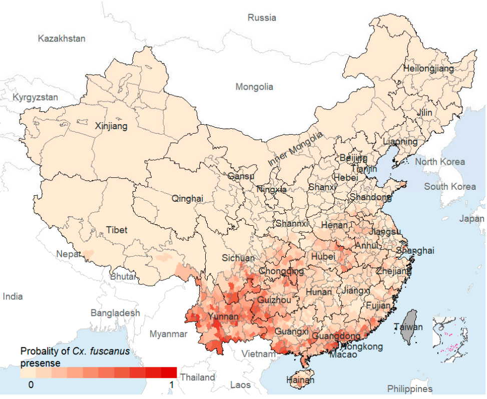

(H) *Cx. fuscanus*

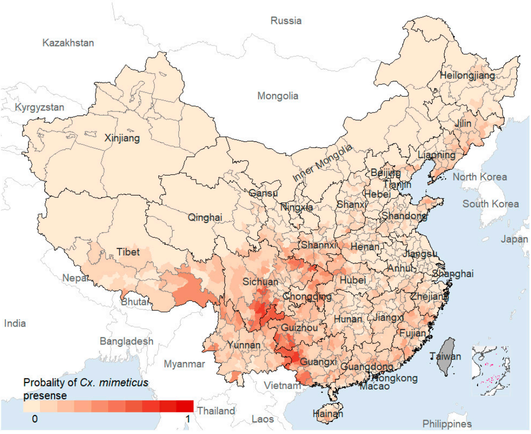

(I) *Cx. mimeticus*

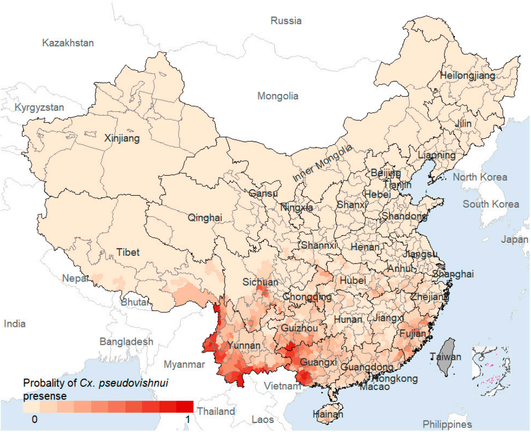

(J) *Cx. pseudovishnui*

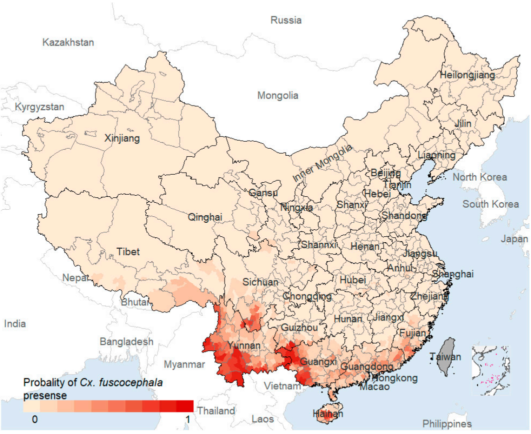

(K) *Cx. fuscocephala*

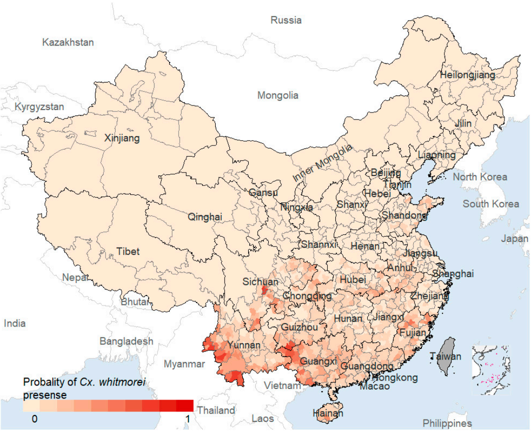

(L) *Cx. whitmorei*

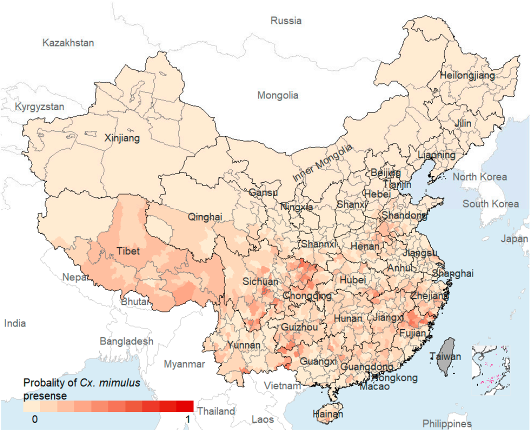

(M) *Cx. mimulus*

Supplementary Figure S40 The predicted county-level distributions of the 13 most prevalent mosquito species in the *Culex* genus, averaged over the ensemble of BRT models (A) *Cx. tritaeniorhynchus*, (B) *Cx. pipiens quinquefasciatu*, (C) *Cx. pipiens pallens*, (D) *Cx. Bitaeniorhynchus*, (E) *Cx. Vagans*, (F) *Cx. Halifaxia*, (G) *Cx. Modestus*, (H) *Cx. Fuscanus*, (I) *Cx. mimeticus*, (J) *Cx. pseudovishnui*, (K)*Cx. fuscocephala*, (L) *Cx. whitmorei and* (M) *Cx. mimulus*. Source data are provided as a Source Data file.

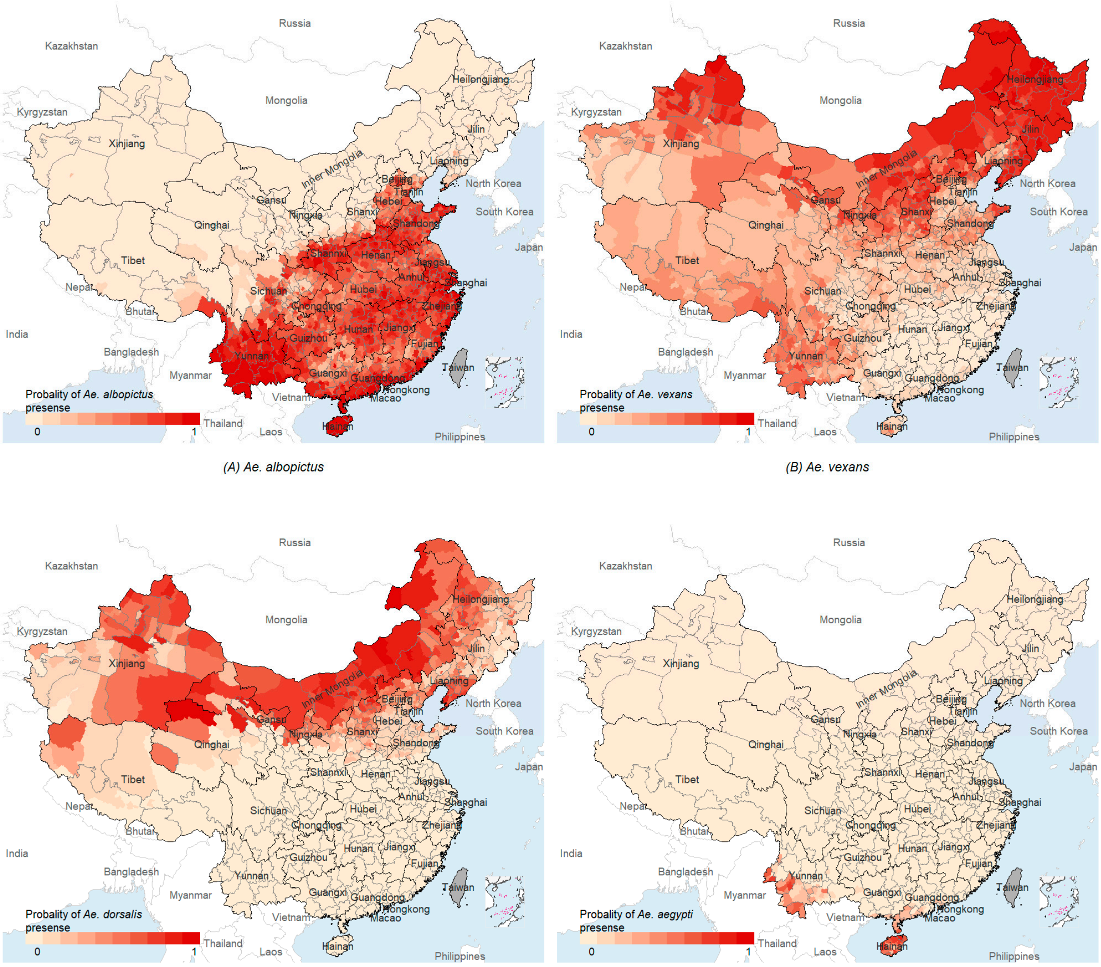

(A) *Ae. albopictus* (B) *Ae. vexans*

(C) *Ae. dorsalis* (D) *Ae. aegypti*

Supplementary Figure S41 The predicted county-level distributions of the four most prevalent mosquito species in the *Aedes* genus, averaged over the ensemble of BRT models (A) *Ae. albopictus,* (B) *Ae. vexans,* (C) *Ae. dorsalis and* (D) *Ae. aegypti.* Source data are provided as a Source Data file

Supplementary Table S8. The mosquito-borne arboviruses included in current study.

Family	Genus	Name of viruses	Abbreviation	Infectivity
Flaviviridae	*Flavivirus*	Japanese encephalitis virus	JEV	*
		Dengue virus	DENV	*
		Yellow fever virus	YFV	*
		Zika virus	ZIKV	*
		West Nile virus	WNV	*
		Tembusu virus	TMUV	*
		Kunjin virus	KUNV	*
		Murray Valley encephalitis virus	MVEV	*
		St. Louis encephalitis virus	SLEV	*
Togaviridae	*Alphavirus*	Getah virus	GETV	*
		Chikungunya virus	CHIKV	*
		Sindbis virus	SINV	*
		Venezuelan equine encephalitis virus	VEEV	*
		Western equine encephalitis virus	WEEV	*
		Eastern equine encephalitis virus	EEEV	*
		Ross River virus	RRV	*
		Mayaro virus	MAYV	*
		Barmah Forest Virus	BFV	*
		Semliki Forest virus	SFV	*
		Sagiyama virus	SAGV	*
Peribunyaviridae	*Orthobunyavirus*	Ťahyňa virus	TAHV	*
		Oya virus	OYAV	*
		Batai virus	BATV	*
		Ebinur Lake virus	EBIV	*
		Snowshoe hare virus	SSHV	*
Reoviridae	*Orbivirus*	Tibet orbivirus	TIBOV	
		Yunnan orbivirus	YUOV	*
		Novel orbivirus	NOBV	*
Reoviridae	*Seadonavirus*	Banna virus	BAV	*
		Mangshi virus	Mangshi virus	
		Kadipiro virus	KDV	*
Mesoniviridae	*unknown*	Nam Dinh virus	NDV	*
Peribunyaviridae	*Phlebovirus*	Rift Valley fever virus	RVFV	*
Orthomyxoviridae	*Quaranjavirus*	Longchuan virus	Longchuan virus	
Rhabdoviridae	*Almendravirus*	Menghai rhabdovirus	MRV	

* Viruses infectious to humans.

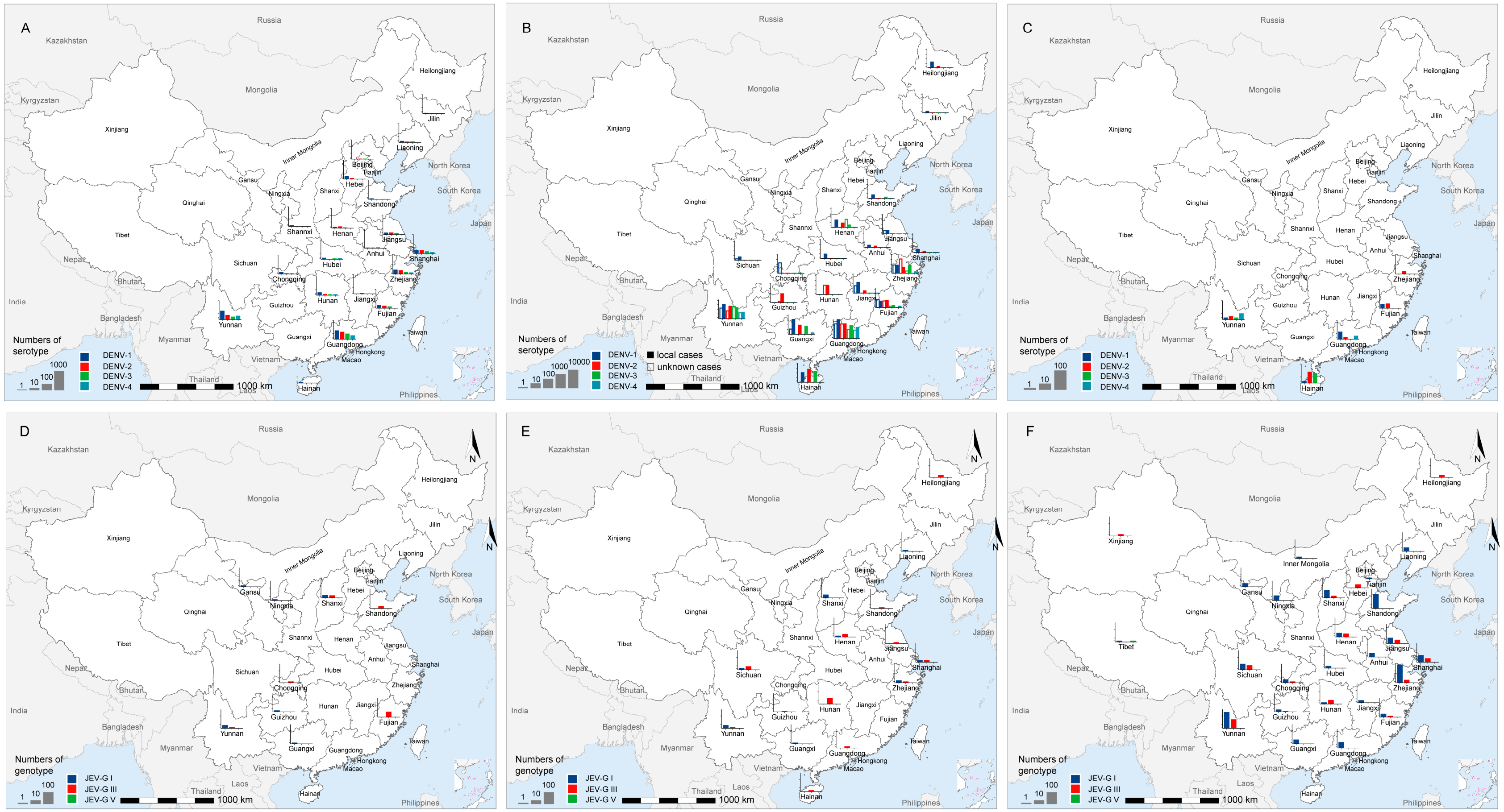

Supplementary Figure S42 The distribution of genotypes of JEV and serotypes of DENV from people, host animals and mosquitoes at the province level in China. (A) serotype of DENV from imported cases; (B) serotype of DENV from local and unknown cases; (C) serotype of DENV from mosquitoes; (D) genotype of JEV from cases; (E) genotype of JEV from host animals; (F) genotype of JEV from mosquitoes.

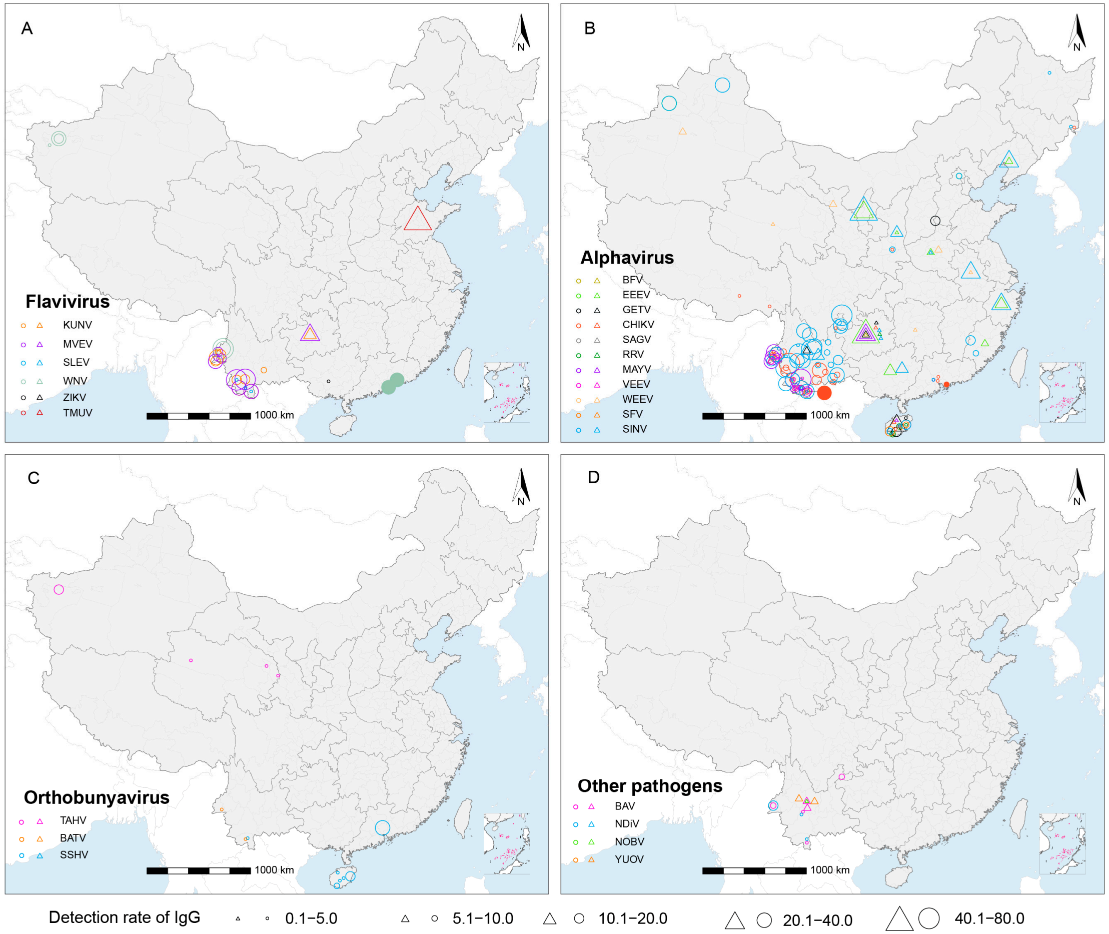

Supplementary Figure S43 The locations of IgG against mosquito-borne arboviruses detected from humans in during 1954–2020 in China. Data were plotted at the county or prefecture level, depending on available resolution in the literature. The viruses detected only at province level was represented as triangle. (A) Flavivirus; (B) Alphavirus; (C) Orthobunyavirus; (D)other viruses.

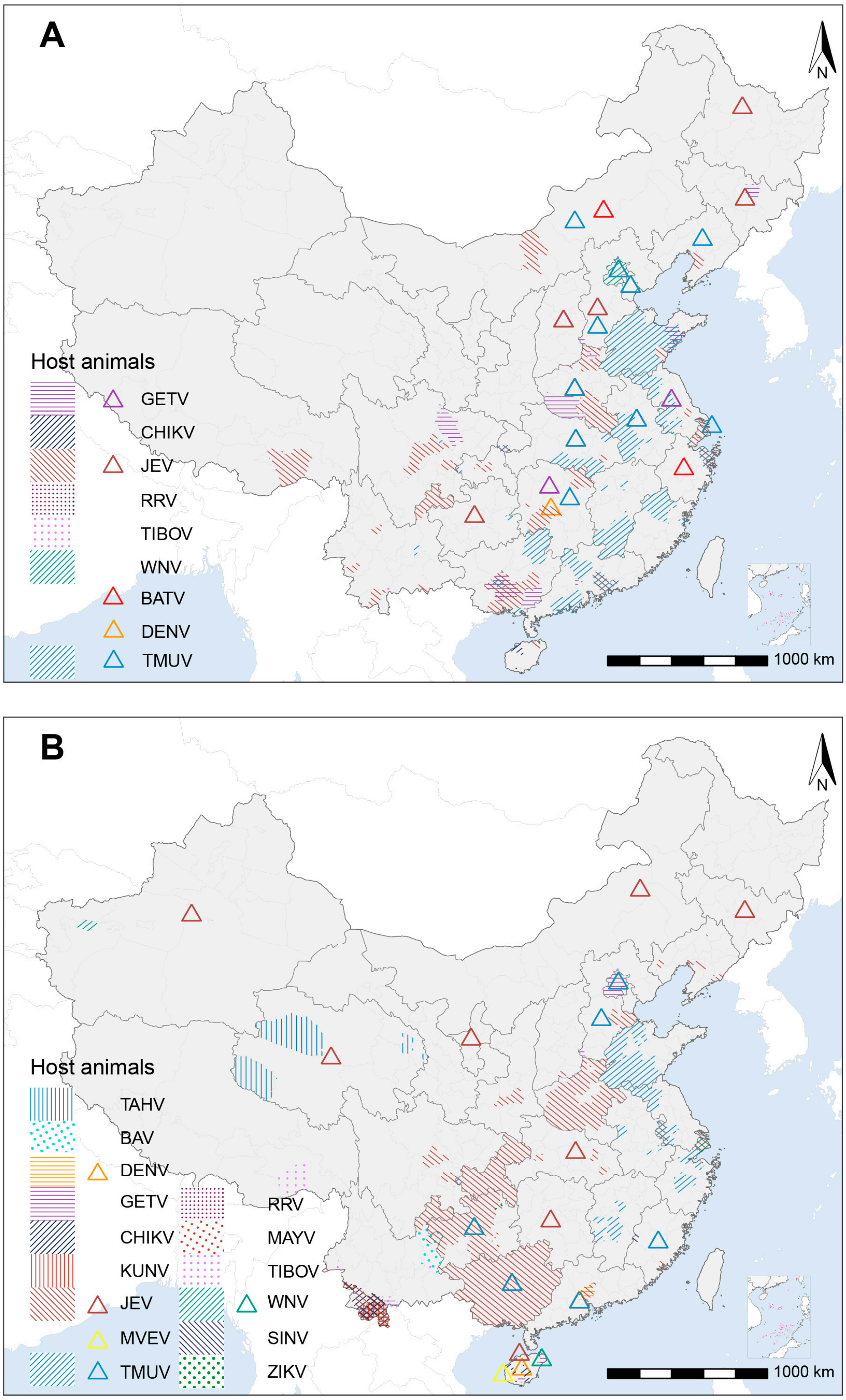

Supplementary Figure S44 The locations of mosquito-borne arboviruses detected in host animals during 1954–2020 in China. Data were plotted at the county or prefecture level, depending on available resolution in the literature. The viruses detected only at province level was represented as triangle. (A) detected with viruses or their IgM; (B) detected with IgG antibody. Source data are provided as a Source Data file.

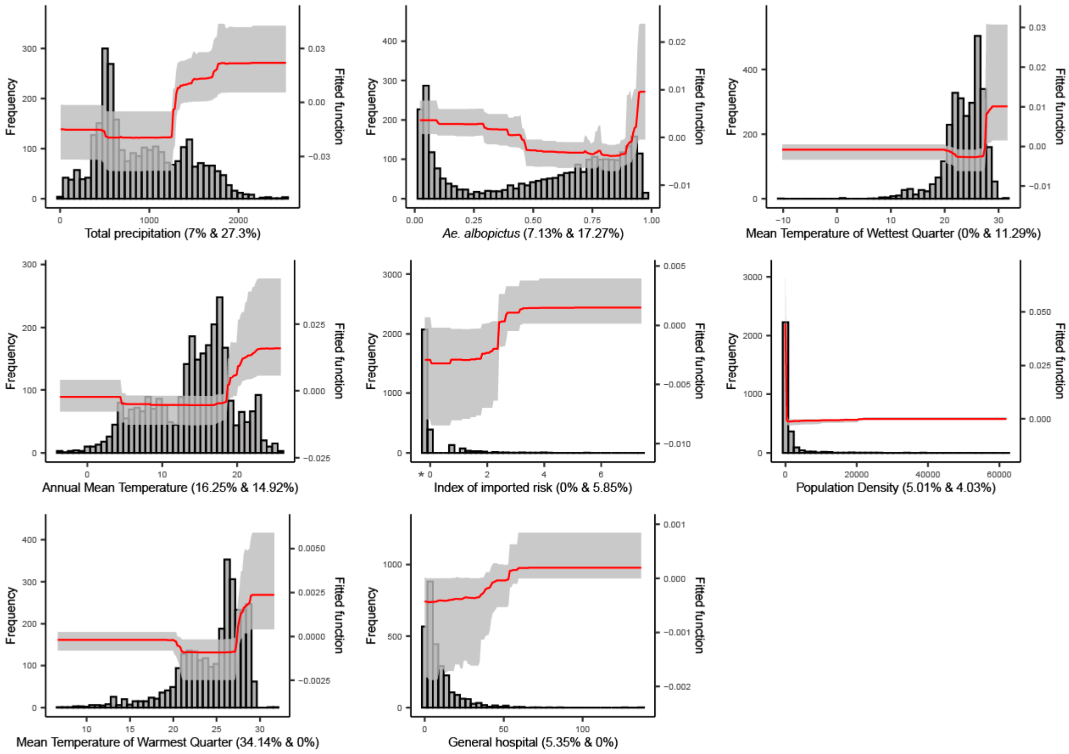

Supplementary Figure S45 The mean curves (red) and 95% percentiles (gray) for the effects of major predictors (RC≥5%) on the predicted incidence rate of dengue based on the ensemble of two stages of GBRT models. The relative influences of predictors in the two models are shown in the parentheses. The former numbers represent the influences of first stage model (based on Logistic distribution) and the latter numbers represent the influences of second stage model (based on Gamma distribution). In the curve of index of imported risk, asterisk represent the index value of zero case.

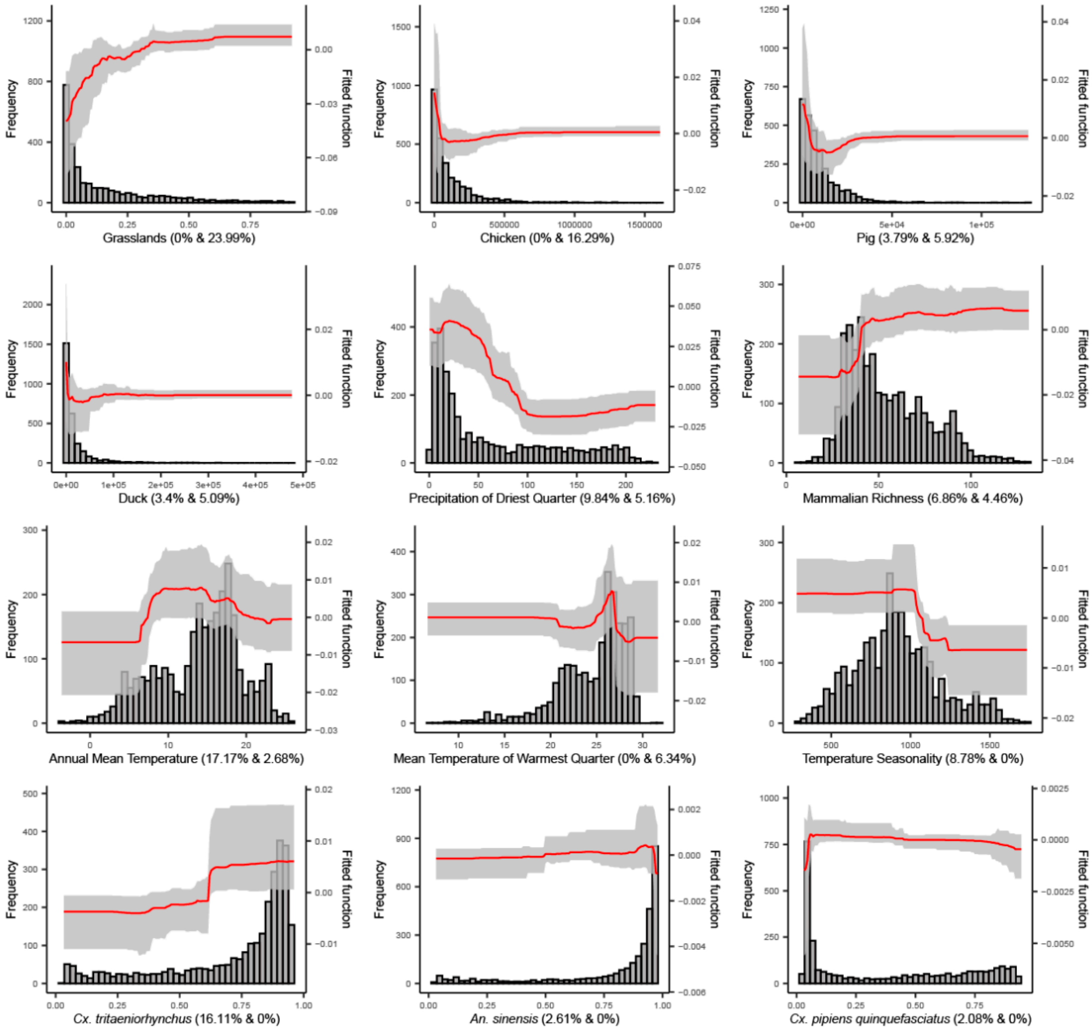

Supplementary Figure S46 The mean curves (red) and 95% percentiles (gray) for the effects of major predictors (RC≥5%) on the predicted incidence rate of Japanese encephalitis based on the ensemble of two stages of GBRT models. The relative influences of predictors in the two models are shown in the parentheses. The former numbers represent the influences of model based on Logistic distribution and the latter numbers represent the influences of model based on Gamma distribution.

Supplementary References

1 Dai, S.; Kong, F.J.; Chen, L.F.; Huang, Z.X.; Mai, Y.X. Carrying HBsAg in different physiological states of *Culex pipiens pallens* in Shiwan area of Foshan City. *South China J Prev Med* **1987,**1, 13–15. Chinese.

2 An, J.Y.; Yu, Y.X.; Gao, Y.G.; Zou, M.J.; Liu, G.P.; Xu, Z.F. A study on the blood-sucking insects of "three shifts" in Zhenbao Island area: III Investigation on the composition of mosquitoes. *Mil Med Sci* **1986,**5, 363–368. Chinese.

3 An, J.Y.; Yu, Y.X.; Ma, D.X. Observation on the diurnal stinging activity of *Aedes caspius* and *Titanopteryx maculata Meigen* in the Caspian Sea in Beiwan area. *Mil Med Sci* **1989,**3, 199–201. Chinese.

4 An, P.T.; Shi, K.W.; Shi, Y.P. Analysis on Background Investigation of Mosquito Vectors In Huanghua Port In 2017. *Port Health Control* **2018,**23, 56–58. Chinese.

5 Bao, J.S.; Zhao, L.S.; Wei, Y. Investigation report on mosquitoes at Meizhou Port. *Port Health Control* **2009,**14, 45–47. Chinese.

6 Bao, Q.H.; Jiang, C.M.; Wu, L.M.; Yu, H.N. Monitoring results of mosquito density and drug resistance in Qiandao Lake Town. *Zhejiang Prev Med* **2016,**28, 60–62. Chinese.

7 Cai, C.L.; Wang, A.M.; Song, X.F.; Hui, B.; Yin, Z.H. A preliminary report on the investigation of flies and mosquitoes in Haian mouth. *J Med Pest Control* **1998,**1, 32–34. Chinese.

8 Cai, C.L.; Wang, A.M.; Xu, B.F.; Lin, J.X.; Chen, J.C. Investigation of Distribution, Ecological Status, Density and Seasonal Growth-decline of Aegypti in Leizhou Peninsula. *Chinese Frontier Health Quarantine* **2007,**6, 361–364+370. Chinese.

9 Cai, H.Q. A preliminary report on the investigation of vector species of Brugia malayi in Shucheng Chengbei Township and Chengguan Town, Anhui Province (Abstract). *Acta Universitatis Medicinalis Anhui* **1959,**1, 81. Chinese.

10 Cai, J.S.; Yao, Z.J. Detection of dengue virus in *Aedes albopictus* in Haizhu District of Guangzhou City. *Journal of Qiqihar Medical University* **2011,**32, 1970–1971. Chinese.

11 Cai, J.Y.; Yan, J.; Li, G.T. Analysis of mosquito surveillance results in Kongtong District of Pingliang City from 2006 to 2011. *Strait J Prev Med* **2013,**19, 68–69. Chinese.

12 Cai, W.; Hou, W.Y.; Zhang, Y.; Ying, H.Q.; Zhao, Z.; Wang, L.; Xu, Y. Analysis on population density of *Culex pipiens pallens* in different habitats in Haidian District from 2013 to 2015. *Chin J Hyg Insect & Equip* **2017,**23, 541–543. Chinese.

13 Cai, W.; Liu, L.X.; Zhang, Y.; Liu, M.D.; Hou, W.Y.; Zhao, Y.; Wang, L.; Xu, Y. Investigation of population seasonality and insecticide resistance of *Culex pipiens pallens* larvae in Haidian District Beijing in 2015. *J Med Pest Control* **2017,**33, 854–856. Chinese.

14 Cai, Z.X.; Yu, Z.S.; Lv, Q.F.; Jiang, H. A preliminary study on mosquitoes and vector mosquitoes of JEV in Dalian. *Journal of Dalian Medical University* **1986,**1, 49–52. Chinese.

15 Cao, H.L.; Zhang, Y.J.; Qu, F.Y. Mosquitoes and their geographical distribution in southern Xinjiang. *Bull Dis Control Prev* **1993,**2, 48–53. Chinese.

16 Cao, H.; Leng, P.E.; Zhou, Y.B.; Ding, Z.W.; Lou, J.P.; Shu, H.H. The comparison study of red box and CO2 trap in mosquito surveillence. *Chin J Hyg Insect & Equip* **2009,**15, 445–448. Chinese.

17 Cao, T.Q.; Yang, S.H.; Wang, J.N.; Zuo, Y.H.; Luo, Z.W.; Ji, Y. Mosquito Survey and Diversity Analysis in Zhalong National Nature Reserve. *Biological Disaster Science* **2020,**43, 58–61. Chinese.

18 Cao, X.B.; Ji, G.; Zhang, Y.C.; Tong, H.Y. Analysis of mosquito vector monitoring results in rural areas of Hai'an city from 2016 to 2019. *Anhui J Prev Med* **2020,**26, 470–473. Chinese.

19 Cao, X.M.; Fang, Z.Q.; Li, Y.; Liu, Y.Y.; Ye, H.B.; Yun, X.Y.; Xu, J.; Zhang, X.L. Monitoring and analysis of mosquitoes at key ports of Guangxi and Yunnan along the Belt and Road. *Chin J Hyg Insect & Equip* **2021,**27, 21–25. Chinese.

20 Cao, X.M.; Fang, Z.Q.; Wang, Y.; Wang, J.M.; Zhang, X.L. Investigation on the population structure of mosquitoes and mosquito-borne pathogens at Mohan port of Yunnan Province in 2018. *Chin J Hyg Insect & Equip* **2020,**26, 131–134. Chinese.

21 Cao, X.J.; Li, X.L.; Guo, Z.N.; Wang, M.Z.; Zhang, Z.W.; Chen, W.; Chen, G.W. Investigation of adult mosquito density in Huli District of Xiamen City. *Chin J Hyg Insect & Equip* **2015,**21, 499–500. Chinese.

22 Cao, Y.O.; Liu, X.C.; Zhu, X.P.; Zhang, J.K.; Qian, W.P.; Luan, R.S. Analysis of surveillance results of dengue fever in Sichuan province. *Chin J Vector Biol & Control* **2011,**22, 117–120. Chinese.

23 Cao, Y.C.; Liu, C.M.; Chen, J.Y. A preliminary observation on the breeding status of *Anopheles sinensis* in Shenyang Paddy Field. *Chinese Journal of Ecology* **1984,**3, 46–47. Chinese.

24 Ceng, D.J.; Wu, S.L.; Wu, N.J.; He, Z.M.; Chen, W.W.; Wang, D.Q. Insecticides resistance of aedes albopictus larvae in different habitats in Pingshan District of Shenzhen. *Modern Preventive Medicine* **2019,**46, 2653–2657. Chinese.

25 Ceng, L.H.; Wang, S.Q.; Liu, Y.; Zhao, W.; Li, S.G.; He, C.H.; Ou, T.T. Analysis of the surveillance data about malaria vector in Hainan from 2005 to 2014. *China Tropical Medicine* **2015,**15, 1436–1440. Chinese.

26 Che, W.H.; Huang, Y.Q.; Cai, M.J.; Chen, F.L. Investigation on the Distribution of *Anopheles anthropophagus* in Hanchuan City, Hubei Province. *Journal of Pathogen Biology* **2002,**1, 58. Chinese.

27 Chen, C.W.; Guo, Y.H.; Sun, C.X.; Ren, D.S.; Zhu, L.; Liu, X.; Luo, Y.D.; Shen, Y.; Liu, J.L.; Liu, X.B, et al. Analysis on mosquito species composition and seasonal fluctuation in Yongcheng city of Henan province. *Chin J Vector Biol & Control* **2017,**28, 144–147. Chinese.

28 Chen, H.B. Notes on a new species of the genus *Culex*, *Culex hainanensis*. *Acta Entomologica Sinica* **1977,**1, 95–98. Chinese.

29 Chen, H.B. A new species of *Culex*, *Culex tianpienensis*. *Journal of Guizhou Medical University* **1980,**2, 69–74. Chinese.

30 Chen, H.B. New records of *Culex* from China. *Journal of Guizhou Medical University* **1980,**2, 75–77. Chinese.

31 Chen, H.B. New records and some revisions of culex from China. *Acta Zootaxonomica Sinica* **1980,**4, 448. Chinese.

32 Chen, H.B. A new species of *Culex*, *Culex miaolinggensis*, found in Guizhou. *Acta Zootaxonomica Sinica* **1982,**3, 312–316. Chinese.

33 Chen, H.B. A new species of the genus *Culex* from China (Diptera: Culicidae). *Acta Zootaxonomica Sinica* **1989,**1, 86–90. Chinese.

34 Chen, H.B. Four new records of the genus *Culex* from China. *Sichuan Journal of Zoology* **1989,**2, 28–29. Chinese.

35 Chen, H.B.; Dong, X.S. The first discovery of the genus *Udaya* in China (Diptera: Culicidae). *Sichuan Journal of Zoology* **1989,**3, 23–24. Chinese.

36 Chen, H.B.; Dong, X.S.; Wang, X.Z. A new species of the genus *Culex* (Diptera: Culicidae). *Acta Zootaxonomica Sinica* **1992,**1, 78–83. Chinese.

37 Chen, H.L.; Shi, J.X.; Deng, M.Z.; Bai, Y. Investigation on Ecological habits of *Anopheles sinensis* in Fengqiu area. *Henan J Prev Med* **1979,**4, 54–58. Chinese.

38 Chen, H.S.; Huang, S.B.; Li, T.K.; Liu, L.S.; Xie, Z.L.; Jiang, N.Y. Investigation on the growth and decline of mosquitoes in Guilin Airport in 1983. *Chinese Frontier Health Quarantine* **1985,**1, 24–33. Chinese.

39 Chen, H.; Lv, M.; Xing, L.D.; Deng, H.P.; Teng, Z.X.; Leng, P.E. A study of the breeding characteristics of *Aedes albopictus* in different housing types in Jing′an district of Shanghai, China. *Chin J Vector Biol & Control* **2019,**30, 536–539. Chinese.

40 Chen, J.; Fan, S.Y.; Shi, X.H.; Chen, J.Z.; Niu, J.F.; Lai, Z.F. Study on insecticide resistance in *Aedes albopictus*, Futian district, Shenzhen city, 2016. *Prev Med Trib* **2018,**24, 248–250. Chinese.

41 Chen, J.; Liang, X.Y.; Zhang, X.; Li, N.J.; Nong, L.H.; Jiang, Y.M. Investigation on resistance of *Aedes albopictus* larvae to common insecticides in Baiyun District, Guangzhou, 2017. *China Tropical Medicine* **2018,**18, 131–134. Chinese.

42 Chen, J.L.; Sun, X.H.; Zhang, Q.H.; Bai, H.Y.; Yang, X.D.; Wu, Z.K. A survey on mosquitoes in Hekou (Yunnan, China)-Laocai (Laocai, Vietnam) ports region. *Chinese Frontier Health Quarantine* **2015,**38, 124–127. Chinese.

43 Chen, M.; Wu, Y.P.; Zhu, G.R.; Feng, X.Y.; Yang, T.C. Preliminary investigation on vectors of mosquito-borne diseases in Yiwu, Zhejiang. *Chin J Vector Biol & Control* **2011,**22, 581–583. Chinese.

44 Chen, Q.; Ma, M.H.; Huang, M.S.; Leng, P.E. Investigation and analysis on the breeding of *Culex pipiens pallens* larvae in an outdoor water treatment testing system in Shanghai, China. *Chin J Vector Biol & Control* **2017,**28, 308–313. Chinese.

45 Chen, T.M.; Zhou, H.N.; Luo, C.H.; Ceng, X.C.; Guo, X.R.; Lin, Z.R.; Tu, H.; Wang, X.Z.; Zhang, S.S.; Zhou, S.S., et al. Composition of *Anopheles* larvae in the China-Myanmar border region in Yingjiang County of Yunnan Province. *Chin J Parasitol Parasit Dis* **2018,**36, 112–118. Chinese.

46 Chen, W.J.; Liu, Z.H.; Meng, D.P.; Zhao, D.Y. Risk Assessment of the Imported Mosquito-borne Infectious Diseases at Tianjin Port by *Aedes* Monitoring. *Port Health Control* **2018,**23, 59–62. Chinese.

47 Chen, W.Z.; Chen, Y.B.; Liu, Y.; Kuang, J.S.; Chen, M.J.; Chen, C.Q.; Zhao, Z.G.; Li, X.D.; Liu, J.H.; Cui, W.Q, et al. Isolation of two strains of Japanese Encephalitis virus from *Culex tritaeniorhynchus* for the first time in Hainan Island. *South China J Prev Med* **1986,**2, 30–34. Chinese.

48 Chen, W.Z.; Zhou, L.W.; Lin, C.Y.; Wang, J.H.; Zhao, Z.G.; Li, X.D.; Ji, Y.J.; Cui, W.Q.; Yu, Y.X. Isolation of dengue virus from *Culex pipiens pallens*. *Hainan Med J* **1982,**1, 33–35. Chinese.

49 Chen, X.L.; Liu, Y.; Wang, D.W.; Cao, J.Z.; Chen, Y.N. Mosquito monitoring report at Beijing International Airport in 2013. *Chinese Frontier Health Quarantine* **2014,**37, 299–301. Chinese.

50 Chen, X.L.; Shi, Y.; Zhou, Y.N.; Chen, Y.N.; Cao, J.Z.; Qiao, D.S.; Wang, Z. Investigation report on mosquitoes at Capital Airport Port in 2014. *Chinese Frontier Health Quarantine* **2015,**38, 71. Chinese.

51 Chen, X.N.; Lv, X.H.; Wang, E.N.; Li, Q. A preliminary investigation on the species and living habits of mosquitoes in summer resorts. *Journal of Chengde Medical University* **1986,**2, 54–56. Chinese.

52 Chen, X.G.; Yuan, B.; Yuan, G.L. Distribution and seasonal dynamics of mosquito species in Ningde, Fujian province. *Chin J Vector Biol & Control* **2010,**21, 368–370. Chinese.

53 Chen, X.C.; Sai, S.Y.; Hu, G.Y. A preliminary report on the ecological study of mosquitoes. *Journal of Taishan Medical College* **1982,**1, 26–29+37. Chinese.
54 Chen, X.N.; Lin, S.H.; Wu, Z.J.; Fang, Y.; Zhou, L.; Lu, X.F. Monitoring results analysis of mosquitoes at Honghai Bay New Port of Shanwei from 2015 to 2016. *Chin J Health Lab Tec* **2018,**28, 867–869. Chinese.
55 Chen, Y.X.; Zhang, X.R.; Ye, S.L.; Peng, M.J.; Ceng, Y.Q.; Chen, X.T.; Li, Y.Z.; Chen, Q.; Yu, S.Y. Applicability of three surveillance methods for *Aedes albopictus* in dengue risk indication. *Chin J Dis Control Prev* **2019,**23, 723–727. Chinese.
56 Chen, Y.; Chen, P.; Zhou, R.L.; Ou, Y. An investigation of *Aedes* in Ningde port. *Chin J Hyg Insect & Equip* **2015,**21, 58–59+62. Chinese.
57 Chen, Z.Y.; Fang, Y.L.; Xie, H.G.; Yang, F.Z.; Xu, B.H. Mosquito species and their habitats in Zhangzhou, Fujian. *Chin J Vector Biol & Control* **2015,**26, 555–557. Chinese.
58 Chen, Z.Y.; Xie, H.G.; Xiao, L.Z.; Ou, Y.R.; Zhang, S.Y. An investigation of malaria vector species in historical distribution areas of *Anopheles lesteri* in Fujian province, China. *Chin J Vector Biol & Control* **2019,**30, 348–349+353. Chinese.
59 Chen, Z.J.; Qin, B.; Bai, A.Y.; Wu, J.; Deng, H.; Duan, J.H.; Liu, L.P.; Lu, R.P.; Yin, W.X.; Lin, L.F., et al. An experimental study of interspecific competition between *Aedes aegypti* from Wushi town of Leizhou and *Ae. albopictus* from different places in Guangdong province, China. *Chin J Vector Biol & Control* **2020,**31, 486–489. Chinese.
60 Chen, Z.J.; Xing, F.; Zhang, L.J.; Deng, H.; Zhou, J.Y.; Huang, J.Y.; Cai, S.W.; Liu, L.P.; Yin, W.X.; Wu, J, et al. Investigation of distribution of *Aedes aegypti* and *Ae. albopictus* in Leizhou, Guangdong province. *Chin J Vector Biol & Control* **2018,**29, 46–49. Chinese.
61 Chen, Z.H.; Huang, J.; Tang, G.; Li, Q.; Yan, S.L.; Liu, Y. Investigation on the distribution of dengue vector *Aedes albopictus* in Panzhihua City. *Chin J Hyg Insect & Equip* **2020,**26, 243–246. Chinese.
62 Chen, Z.H.; Huang, J.; Tang, G.; Yan, S.L.; Li, Q.; Li, W.C.; Chen, S.P.; Liu, Y. Analysis on *Aedes albopictus* density surveillance in Panzhihua City in 2015. *Chin J Hyg Insect & Equip* **2016,**22, 487–489. Chinese.
63 Chen, Z.H.; Pan, H.Y.; Huang, J.; Tang, G.; Yan, S.L.; Liu, Y.; Li, Q.; Huang, X.H.; Shen, L.H. Resistance of *Aedes albopictus* to commonly used insecticides in Panzhihua City. *Chin J Hyg Insect & Equip* **2016,**22, 38–39+45. Chinese.
64 Zhen, X.S.; Lu, X.X. Study on isolation of viruses from common mosquito in Shanghai. *Acta Microbiologica Sinica* **1957,**5, 324–330. Chinese.
65 Chen, S.Y. Analysis on the correlation between mosquito surveillance and diseases in Nanchang County in 2015. *World Latest Medicine Information* **2017,**17, 121. Chinese.
66 Cheng, Q.L. Correlation analysis between the density of *Anopheles sinensis* and the incidence of malaria in Nanxi County from May to September, 1994-1997. *Parasitoses and Infectious Diseases* **1999,**S1, 30–31. Chinese.
67 Chu, H.L.; Chen, D.Y.; Yang, W.F.; Liu, D.P.; Zhang, A.J.; Chen, Z.L.; Liu, H.; Xu, Y.; Sun, J. Larvae resistances of *Culex pipiens pallens* in Jiangsu in 2009. *Chin J Hyg Insect & Equip* **2010,**16, 188–190. Chinese.
68 Cui, M.H.; He, R.W.; Wang, H.; Yao, C.Y. Surveillance on adult mosquito population density from 2013 to 2015 in Suqian City. *Chin J Hyg Insect & Equip* **2016,**22, 577–579+581. Chinese.

69 Cui, S.H.; Fu, S.H.; Zhao, S.C.; Cao, L.; Fu, J.N.; Tang, Z.J.; Zhang, J.; Jiang, S.Y.; Lu, X.Q.; Liang, G.D., et al. Investigation of mosquitoes and arboviruses in Delingha area in Qinghai, 2013. *Chin J Parasitol Parasit Dis* **2016,**31, 346–350. Chinese.
70 Cui, X.B.; Hu, X.D.; Yang, J.S. Analysis on mosquito vectors surveillance results of dengue fever in Yun county of Hubei province in 2010. *J Med Pest Control* **2011,**27, 981–983. Chinese.
71 Dai, Y.M.; Deng, C.L.; Luo, Y.C. Investigation and Analysis on the Distribution and growth and decline of mosquitoes in Xuanhan County. *Modern Preventive Medicine* **2010,**37, 156+161. Chinese.
72 Deng, H.P.; Xu, Y.X.; Wang, S.H. Density and insecticide resistance of *Anopheles sinensis* in north suburbs of Shanghai city. *Chin J Hyg Insect & Equip* **2013,**19, 539–542. Chinese.
73 Zhai, S.Y.; Zhao, Z.Q. Investigation and analysis of *Anopheles sinensis* in Shijiazhuang, China in 2012. *Chin J Vector Biol & Control* **2014,**25, 476. Chinese.
74 Zhai, Z.T.; Chen, H.L.; Zhang, F.Y. Investigation on resistance of *Anopheles sinensis* and *Culex pipiens pallens* to several pesticides in seven cities (counties) of Henan Province. *Henan J Prev Med* **1983,**2, 58–61. Chinese.
75 Diao, Y.X.; Li, T.T.; Dan, Z.J.; Qiu, D.Y.; Yue, Q.Y.; Liu, H.Y. Investigation of mosquito and arbovirus in Zhongshan area. *Chinese Frontier Health Quarantine* **2018,**41, 178–180. Chinese.
76 Ding, C.X.; Tian, M.; Huang, J. Surveillance results for mosquitoes in Jiashan county of Zhejiang province, 2016. *Inter J Epidemiol Infect Dis* **2018,**45, 120–123. Chinese.
77 Ding, E.C. Preliminary investigation report on mosquitoes in Quanzhou City. *Journal of Fujian Medical University* **1978,**1, 22–28. Chinese.
78 Ding, L.M.; Lu, Z.R.; Zhou, X.X.; Yang, Q. Investigation on mosquitoes in Hongqiao Airport area of Shanghai. *Chinese Frontier Health Quarantine* **1990,**4, 230–233. Chinese.
79 Dong, J.; Lin, L.; Liu, Q.; Zhang, Q.W.; Liu, F.R.; Wang, W.L.; Shen, P.L.; Wu, S.L. Mosquito surveillance and control strategy in Longgang district, Shenzhen in 2009. *J Trop Med* **2012,**12, 80–82. Chinese.
80 Dong, S.H.; Fan, S.H.; Ma, L.; Lei, J.; Zhou, H.N. Survey of *Aedes aegypti* distribution in Mangshi, Yunnan. *Chin J Vector Biol & Control* **2011,**22, 592–594. Chinese.
81 Dong, X.S. A new species of the genus *Topomyia*, subgenus Suaymyia (Diptera: Culicidae). *Zoological Research* **1995,**4, 343–347. Chinese.
82 Dong, X.S.; Wang, X.Z. Notes on a new species of the subgenus *Anopheles*. *Zoological Research* **1985,**2, 117–122. Chinese.
83 Dong, X.S.; Wang, X.Z. A new record of *Topomyia* from China. *Acta Zootaxonomica Sinica* **1987,**2, 180. Chinese.
84 Dong, X.S.; Wang, X.Z.; Lu, Y.R. A new record of *Culex* from China. *Acta Zootaxonomica Sinica* **1983,**2, 161. Chinese.
85 Dong, X.S.; Wang, X.Z.; Zhou, H.N. A new record of the genus *Culex* from China. *Acta Zootaxonomica Sinica* **1993,**1, 53. Chinese.
86 Dong, X.S.; Zhang, Z.Q. Notes on a new record of *Anopheles* Ramsayi from Yunnan province. *Acta Zootaxonomica Sinica* **1979,**3, 306. Chinese.
87 Dong, X.S.; Zhou, H.N.; Wang, P.Y.; Wang, X.Z. A new species of the subgenus *Heizmannia* (Diptera: Culicidae). *Zoological Research* **1997,**1, 28–32. Chinese.

88 Dong, X.S.; Wang, X.Z.; Lu, B.L. A new species of *Topomyia* from Yunnan (Diptera: Culicidae). *Acta Zootaxonomica Sinica* **1995,**4, 484–486. Chinese.

89 Dong, X.S.; Zhou, H.N.; Dong, L.M. A New species of the Genus *Armigeres* (Diptera: Culicidae). *Entomotaxonomia* **1995,**4, 281–286. Chinese.

90 Dong, X.S.; Zhou, H.N.; Dong, L.M. Description of the genus *Armigeres* and a new species from Yunnan (Diptera: mosquito family). *Zoological Research* **1995,**2, 95–104. Chinese.

91 Du, B.; Zhang, C.F.; Li, B.; Liu, S.N. Monitoring report on density and seasonal growth and decline of rodent, fly, mosquito, cockroach in Jiangyang District of Luzhou City. *Chinese Community Doctors* **2008,**10, 249–250. Chinese.

92 Du, L.Y.; Peng, Q.R. Investigation on Ecological habits of *Anopheles sinensis* in Sangzhi County, Hunan Province. *Chin J Parasitol Parasit Dis* **1996,**1, 59. Chinese.

93 Du, Z.W.; Gu, Y.A.; Lu, Y.R.; Li, C.F. Observation on Ecological habits of *Anopheles dirus* in Yunnan Province. *Journal of Pathogen Biology* **1994,**2, 155–156. Chinese.

94 Duan, J.H.; Cai, S.W.; Wu, J.; Yin, W.X.; Deng, H.; Zou, Q.; Lin, L.F. Resistance of *Aedes albopictus* to insecticides in the late stage of emergency control of dengue fever of Guangdong province in 2014. *Chin J Vector Biol & Control* **2017,**28, 141–143. Chinese.

95 Duo, J.Z.M.; Zhuo, M.Y.J.; Yong, J.; Zha, X.Z.M.; Yang, X.D.; Yang, G.R.; Wang, J.; Zhou, H.N.; Gong, Z.D. A study of the taxonomy and fauna of mosquitoes in Tibet autonomous region, China IX: New records of 3 subgenera and 6 species of *Aedes*. *Chin J Vector Biol & Control* **2020,**31, 203–208. Chinese.

96 Fan, T.B.; Wang, J.Q.; Li, H.H. Preliminary investigation on habitat habits of mosquitoes in Tancheng county, Liang Village. *Chinese Journal of Applied Entomology* **1965,**4, 224–227. Chinese.

97 Fan, B.; Ren, D.S.; Yang, Z.X.; Zhang, W.Q.; Ma, X.W. Investigation on *Anopheles* in the early stage of malaria epidemic peak in Yuanjiang County. *J Med Pest Control* **1996,**2, 17–18. Chinese.

98 Fan, B.; Zhang, Z.X.; Huang, Z.M.; Long, S.W.; Su, Y.; Wen, R.S.; Zhao, W.; Du, C.H. Investigation on population number and Distribution of *Anopheles* in Yuanjiang County. *Journal of Pathogen Biology* **1997,**1, 79–80. Chinese.

99 Fan, S.Y.; Gu, J.B.; Shi, X.H.; Chen, J.; Chen, J.Z.; Lin, Z.P. Resistance of *Aedes albopictus* to deltamethrin in Futian District of Shenzhen City. *Chin J Hyg Insect & Equip* **2020,**26, 294–295. Chinese.

100 Fan, Z.H.; Wei, R.Q.; Li, L.J.; Jin, Z.H.; Li, J.; Guo, X.F.; Wang, Y.B.; Zhang, D.; Jiang, X.Y.; Yang, M.D., et al. Investigation of *Aedes* mosquito distribution in the central Yuxi city. *Chin J Vector Biol & Control* **2016,**27, 614–616. Chinese.

101 Fang, Y.L.; Gao, B.; Wang, Y.P.; Xiao, W.; Xu, B.H.; Zhang, J.Q.; Wang, G.H.; Wu, R.Q. Investigation on mosquito-borne species and breeding environment at ports to Taiwan in Fujian. *Chinese Frontier Health Quarantine* **2013,**36, 92–95. Chinese.

102 Fang, Y.; Chen, S.R.; Xie, H.; He, Y.M.; Wang, J.; Li, P.Y. Observation of the ecological habits of *Toxorhynchites* splendens and *Culex fuscanus* larvae in laboratory. *Chinese Frontier Health Quarantine* **2017,**40, 34–38. Chinese.

103 Fang, Y.; Chen, S.R.; Xie, H.; He, Y.M.; Wang, J.; Li, P.Y.; Wang, J.; Han, X.Q. Investigation of population composition and seasonal growth-decline of mosquitoes at Nanning airport. *J Med Pest Control* **2017,**33, 714–717+721. Chinese.

104 Fang, Y.; Shi, W.Q.; Zhang, Y.; Hu, Q.A.; Zhou, Z.B.; Wu, J.T.; Zhang, L.L. Discovery of *Culex inatomii*（Diptera ： Culicidae） in Chongming, Shanghai. *Chin J Parasitol Parasit Dis* **2017,**35, 31–35. Chinese.

105 Feng, Y.; Chen, W.W.; Yang, W.H.; Zhang, Y.Z.; Yang, D.J.; Liu, F.; Zhang, J.; Wang, P.Y.; Bai, P.F.; Dou, Y.J, et al. Isolation and identification of mosquito-borne viruses in Mile county, Yunnan province, China in 2009. *Chin J Vector Biol & Control* **2012,**23, 402–405. Chinese.

106 Feng, Y.; Zhang, H.L.; Fu, S.H.; Yang, W.H.; Zhang, Y.Z.; Wang, P.Y.; Yang, J.; Liu, Y.H.; Dong, C.L.; Li, S, et al. Investigation on mosquitoes and mosquoto-brone viruses in Dehong prefecture, Yunnan province, 2007 and 2010. *Chin J Epidemiol* **2014,**35, 528–532. Chinese.

107 Fu, F.H.; Fu, Z.F.; Feng, C.Y.; Tang, J.L.; Pang, X.J.; Si, Y.Z.; Chen, W.J.; Cai, H.L. A preliminary observation on the ecological habits of *Anopheles anthropophagus* in the Nanbeigou Village of Wenchang, Hainan. *Hainan Med J* **1996,**3, 146–147. Chinese.

108 Fu, R.L.; Fan, L.X.; Liu, Y.Q.; Peng, G.H.; Yi, H.S.; Hu, Z.H.; Liu, X.Q.; Feng, X.W. Quantitative study on malaria transmission by *Anopheles sinensis* in Nanchang city. *J Trop Med* **2016,**16, 1438–1440+1444. Chinese.

109 Fu, X.F.; Guo, Y.H.; Gong, Z.D.; Yang, G.R. Distribution of mosquitoes in residential area of Banlao Township, Cangyuan County, Yunnan Province. *Chin J School Doctor* **2014,**28, 689–690. Chinese.

110 Fu, Q.L.; Zhou, J.Y.; Shao, Z.M. Investigation on resistant to insecticides of aedes albopictus in Chancheng District of Foshan City. *Occup and Health* **2017,**33, 2721–2722+2726. Chinese.

111 Gao, J.F. Investigation report on biological characteristics of *Culex tritaeniorhynchus* in Southern Jiangsu Province. *J Med Pest Control* **1998,**5, 50–51. Chinese.

112 Gao, J.F.; Wu, Z.H.; Xu, Q.L. Ecological investigation of adult mosquitoes in Wujin district. *J Med Pest Control* **1994,**2, 128–131. Chinese.

113 Gao, J.F.; Zhou, L.G.; He, C.M. Investigation on biological characteristics of *Anopheles sinensis* in Wujin District of Changzhou City from 1982 to 2002. *Chin J Vector Biol & Control* **2004,**4, 322–323. Chinese.

114 Gao, J.F.; Chen, J.Z.; Zhou, L.K. Observation on the density of harassing *Armigeres subalbatus* in Wujin district. *Chin J Vector Biol & Control* **1996,**1, 71. Chinese.

115 Gao, L.; Lu, C.G.; Li, X.; Li, X.Y.; Yang, J.Z.; Long, F.X.; Bai, M.; Deng, G.H. Surveillance of adult mosquito vectors of Japanese encephalitis in rural areas of Guiyang city. *Chin J Vector Biol & Control* **2010,**21, 551–553. Chinese.

116 Gao, Q.; Zhou, Y.B.; Cao, H.; Leng, P.E. Status and public health impact of mosquito infestation in residential and green areas in Shanghai downtown. *Chin J Hyg Insect & Equip* **2016,**22, 40–45. Chinese.

117 Gao, Q.; Zhou, Y.B.; Leng, P.E.; Xiong, C.L.; Jiang, Q.W.; Cao, H. Study on adult mosquito population dynamics by human landing catch in downtown Shanghai, China. *Chin J Vector Biol & Control* **2014,**25, 215–218. Chinese.

118 Gao, W.; Huang, G.; Han, X.L.; Song, J.W. An investigation and analysis of the emergence of *Aedes albopictus* in small water containers in surveillance sites for dengue vector *Aedes* in Hebei province, China. *Chin J Vector Biol & Control* **2019,**30, 648–652. Chinese.

119 Gao, X.Y.; Fu, S.H.; Zou, W.J.; Hu, Y.Q.; Liu, H.; Zhan, F.X.; Huo, X.X.; Liang, G.D. Investigation of mosquito-borne arboviruses in some regions of Hubei province, China in 2009. *Chin J Vector Biol & Control* **2012,**23, 421–423+427. Chinese.

120 Gao, X.Y.; Fu, S.H.; Zou, W.J.; Peng, Y.; Liu, H.; Cao, Y.X.; Jiang, Y.Z.; Liang, G.D. Investigation of arbovirus in some areas of Hubei province, 2010. *Chin J Vector Biol & Control* **2015,**26, 133–136. Chinese.
121 Gao, Y.F.; Cheng, X.L.; Ding, Y.; Liu, Y.; Yang, J.; Lu, Y.L.; Li, X.J.; Wan, D.Z.; Song, F.L. Investigation on mosquito-borne pathogens in the southwest border areas, China. *Chinese Frontier Health Quarantine* **2020,**43, 91–94. Chinese.
122 Gao, Z.G.; Ma, H.M.T.; Deng, H.; Zhang, J.; Liu, T.Z.; Li, X.L.; Zhang, B.Z. Investigation of mosquito species distribution and arbovirus in Xinjiang. *Chin J Vector Biol & Control* **2016,**27, 186–189. Chinese.
123 Ge, B.; Liu, Q.; Zhang, Y.; Li, X.C.; Zhang, X.Y.; Liu, H.L. Investigation on the resistance of *Aedes albopictus* larvae to commonly used insecticides in Fengxian District of Shanghai. *Chin J Hyg Insect & Equip* **2019,**25, 91–92. Chinese.
124 Gong, J.C.; Huang, W.H.; Zhang, W.L.; Shang, W.L. Investigation of mosquitoes in Kaiping Port. *Chin J Vector Biol & Control* **1999,**6, 470. Chinese.
125 Gong, Y.F.; Lei, L.; Li, Z.H.; Shang, G.J.; Li, Z.F.; Zheng, J.G. An analysis of surveillance results of malaria vectors *Anopheles* in Jiangxi province, China, 2018. *Chin J Vector Biol & Control* **2020,**31, 27–31. Chinese.
126 Gong, Z.Y.; Liu, Q.Y.; Hou, J.; Fu, G.M.; Bai, Y.; Xu, X.P.; Chen, Z.P.; Yang, T.C.; Guo, Y.H.; Ren, Z.Y, et al. Integrated monitoring of mosquitoes and mosquito-borne diseases in Zhejiang province. *Chin J Vector Biol & Control* **2010,**21, 184–187. Chinese.
127 Gong, Z.D. Mosquitoes in Dali Prefecture, Yunnan Province. *Sichuan Journal of Zoology* **1984,**4, 17–19. Chinese.
128 Gong, Z.D. Notes on the mosquitoes new to China. *Entomotaxonomia* **1986,**Z1, 54. Chinese.
129 Gong, Z.D. A new species of *Topomyia* (Diptera: Culicidae). *Acta Entomologica Sinica* **1989,**1, 90–91. Chinese.
130 Gong, Z.D. *Aedes andamanensis* was found in China. *Chin J Vector Biol & Control* **1990,**3, 196–194. Chinese.
131 Gong, Z.D. A new species of the genus *Topomyia* from China (Diptera: Culicidae). *Acta Zootaxonomica Sinica* **1991,**2, 228–231. Chinese.
132 Gong, Z.D.; Li, Z.H. A new species of the genus Diptera from Yunnan, China (Diptera: Culicidae). *Acta Zootaxonomica Sinica* **1999,**1, 97–100. Chinese.
133 Gong, Z.D.; Lu, B.L. New records of *Topomyia* in China. *Sichuan Journal of Zoology* **1984,**2, 10. Chinese.
134 Gong, Z.D.; Lu, B.L. Notes of the mosquitoes new to China, 9. *Acta Zootaxonomica Sinica* **1984,**4, 386. Chinese.
135 Gu, M.D. Discovery of four new mosquito species in Lianyun Mountain, Pingjiang county. *J Clin Res* **1986,**2, 98. Chinese.
136 Gu, H.J.; Zhou, J.P.; Shen, H.Y. Surveillance results of mosquito density in rural areas of Lili Town, Wujiang District, Suzhou City in 2014. *J Med Pest Control* **2016,**32, 312–313. Chinese.
137 Gu, Y.A.; Xu, J.W.; Lu, Y.R.; Huang, R.; Zhu, G.J.; Wang, X.; Yang, C.J.; Chen, W.C. Comparative study on the colony characteristics of mosquitoes in villages and wild in semi-mountainous area of Xishuangbanna. *J Med Pest Control* **1997,**4, 193–195. Chinese.
138 Guan, L.F.; Tan, Q.L.; Zhao, J.G.; Ye, L.; Ren, Y. Investigation on the composition and seasonal growth and decline of mosquitoes in Daishan County. *Prev Med* **2018,**30, 704–706. Chinese.

139 Guo, H.J.; Feng, D.; Xiao, S.; Yang, G.J.; Huang, J. Surveillance for *Aedes albopictus* density in Fenggang County and Chishui City of Guizhou Province in 2016. *Chin J Hyg Insect & Equip* **2018,**24, 373–376. Chinese.

140 Guo, Y.H.; Chen, C.W.; Zhu, L.; Liu, X.; Luo, Y.D.; Shen, Y.; Liu, J.L.; Liu, Q.Y. Study on daily activity rhythm of *Aedes albopictus* in Yongcheng city. *Chin J Vector Biol & Control* **2016,**27, 484–486. Chinese.

141 Guo, Z.J.; Hou, Y.F.; Jiang, X.C.; Zhao, Z.Q.; Zhai, S.Y. Investigation on mosquitoes and flies at Xibaipo scenic spot in Shijiazhuang city in 2008. *Chin J Vector Biol & Control* **2009,**20, 601–602. Chinese.

142 Han, H.R.; Mo, X.C.; Chen, Z.P. Investigation on mosquito population and seasonal growth and decline in Xihai Port of Zhanjiang City. *South China J Prev Med* **1991,**4, 52–54. Chinese.

143 Han, J.P.; Li, Z.Y.; Ding, Y.J. Investigation report on mosquito species in Nanjing Airport. *Chin J Hyg Insect & Equip* **1995,**1, 26–27. Chinese.

144 Han, X.H.; Ceng, N.; Fu, W. Investigation on mosquito population and seasonal growth and decline in Qinglan Port. *Chinese Frontier Health Quarantine* **1999,**5, 280–282. Chinese.

145 Hao, B.S.; Chen, J.L. Investigation and study on Blood-sucking mosquitoes and midges in Zhuhai, Guangdong Province. *J Med Pest Control* **1990,**3, 41–44. Chinese.

146 He, C.; Tian, B.; Cheng, W.L.; Ma, T.Z. Seasonal fluctuations in the mosquito density in Shunyi district, Beijing from 2005 to 2008. *Chin J Vector Biol & Control* **2010,**21, 255–256. Chinese.

147 He, G.M.; Li, S.T.; Pan, S.Q.; Li, M.X.; Liang, Z.T.; Xu, F.H.; Zhang, H.J. Observation on the age of *Anopheles dirus* in Hainan Island. *Journal of Sun Yat-sen University (Medical Sciences)* **1983,** Z1, 96–101. Chinese.

148 He, P.Q.; Li, J.; Guo, J.Y.; Lan, T.; Li, H. Investigation of the resistance of *Aedes albopictus* to common insecticides in urban areas in the city of Dongguan in 2018. *Journal of Pathogen Biology* **2020,**15, 202–206. Chinese.

149 He, C.T. Geographical distribution of *Anopheles* in Dulong River Valley, Yunnan Province. *Chinese Journal of Zoology* **1999,**3, 9–10. Chinese.

150 Hou, Y.X.; Wang, L.C.; Yu, Y.K.; Zhu, H.T.; Tu, J.J.; Jia, X.M.; Huang, F.Y. Investigation on the resistance of *Culex pipiens pallens* to commonly used insecticides in Mingguang City. *Chin J Hyg Insect & Equip* **2020,**26, 328–330. Chinese.

151 Hu, Y.Q.; Li, K.J.; Zhang, H.X.; Pei, S.J.; Yuan, F.Y.; Dong, X.R. Detection of *Anopheles sinensis*'resistance to common insecticides in areas of Hubei Province. *Journal of Pathogen Biology* **2010,**5, 611–614. Chinese.

152 Hu, S.L.; Yong, J.; Li, H.D.; Duo, J.Z.M.; Zhuo, M.Y.J.; Yang, X.D.; Wang, J.; Yang, G.R.; Zhou, H.N.; Gong, Z.D., et al. Taxonomy and fauna of mosquitoes in Tibet VI. Genus *Topomyia* and a new record species. *Chin J Vector Biol & Control* **2019,**30, 434–437. Chinese.

153 Hu, W.H.; Ye, T.T.; Yan, Q.L.; Fu, Y.F.; Lu, M.; Xiong, X.Y.; Fang, S.C. Report on technology of monitoring and control for mosquitoes in Nansha port area of Guangzhou city. *Chinese Frontier Health Quarantine* **2008,**31, 25–30. Chinese.

154 Huang, B.X. Collection and record of mosquito species in southwest coastal areas of Guangdong Province. *Chin J Vector Biol & Control* **1992,**5, 336. Chinese.

155 Huang, B.X.; Zhang, Z.Y. Investigation report on mosquito species in three towns of Yangjiang County. *South China J Prev Med* **1989,**1, 58–59. Chinese.

156 Huang, B.Q.; Wang, F.Y.; Zhou, A.W. Observation on the seasonal growth and decline of *Aedes albopictus* in Baise Town. *Journal of Youjiang Medical University for Nationalities* **1988,**2, 42–44. Chinese.

157 Huang, D.F.; Ling, B.Z.; Huang, Y.J.; Ban, Y.H.; Li, F.H.; Li, Z.L. Results of surveillance of mosquito vectors transmitting dengue fever in Fangchenggang City from 2005 to 2010. *China Tropical Medicine* **2011,**11, 1211–1212. Chinese.

158 Huang, F.S.; Wang, X.X.; Xiao, G.Z. New records of mosquitoes in China. *Acta Entomologica Sinica* **1983,**1, 120. Chinese.

159 Huang, G.Q.; Zhang, H.X.; Liu, J.Y.; Yuan, F.Y.; Yu, P.H.; Chen, G.Y.; Ming, G.Z.; Zhang, J.B.; Xu, B.Z. Study on the Ecology Character of Distribution and Role of Malarial Transmission in *Anopheles anthropophagus* in Hubei, China. *Chin J Vector Biol & Control* **2000,**3, 208–212. Chinese.

160 Huang, G.C.; Wang, J.S.; Jiang, Y. Mosquito species composition and vertical distribution of *Aedes albopictus* in bamboo forest of Wuyishan Nature Reserve, Fujian Province (Diptera: Culicidae). *Wuyi Science Journal* **1985,**5, 77–80. Chinese.

161 Huang, H.M.; Su, B.Y.; Li, G.F. Effect of *Anopheles anthropophagus* for tramission of malaria in Baibao brigade of Shangsi County. *Guangxi Medical Journal* **1987,**3, 119–121. Chinese.

162 Huang, J.R.; Li, J.L.; Tang, G.K.; Zhang, G.D. Experience in eliminating *Aedes aegypti* in Qisha Town, Fangcheng County, Guangxi province. *Chin J Public Health* **1992,**1, 35–36. Chinese.

163 Huang, P.X.; Song, H.L.; Zhang, Y.P.; Gu, Z.Q.; Min, J.G.; Wei, X.Y.; Yao, L.T. Investigation on natural infection of the encephalitis B virus of mosquitoes in pig houses in early spring. *Chin J Public Health* **2004,**11, 80–82. Chinese.

164 Huang, Q.F.; Wu, Q.; Lin, H.; Zheng, F.; Wang, S.; Lin, Z.; Chen, Y.; Du, X.; Gong, S.B. *Aedes* surveillance in Luoyuan bay harbor in Fuzhou, 2017. *Chinese Frontier Health Quarantine* **2018,**41, 414–416. Chinese.

165 Huang, S.C.; Cha, J.X.; Huang, Z.M.; Chen, B.; Shen, K.Z. Monitoring of adult mosquitoes in Zhangzhou City in 2016. *Chin J Hyg Insect & Equip* **2018,**24, 484–487. Chinese.

166 Huang, S.Y.; Huang, L.G.; Huang, J.R.; Meng, Z.Q.; Tan, Y.J.; Huang, Y.M. Population density of *Anopheles* in Huanjiang County, Guangxi. *China Tropical Medicine* **2019,**19, 352–355+381. Chinese.

167 Huang, S.L.; Kang, S.P.; Lin, X.W.; Chen, J.Y. Monitoring report on mosquito population and seasonal growth and decline in Xiuyu Port at Putian city. *Port Health Control* **2010,**15, 43–45. Chinese.

168 Huang, X.D.; Wang, H.F.; Tian, H.; Guo, X.X.; Liu, L.J.; Shi, Q.Q.; Song, X.; Zhang, C.X.; Wang, H.W.; Cheng, P., et al. Seasonal fluctuations of mosquitoes and application of two surveillance tools in Taibai Lake area of Jining City. *Chin J Schisto Control* **2018,**30, 329–331. Chinese.

169 Huang, Y.W.; Peng, S.Q.; Lian, C.L. Survey on Mosquito at Shenzhen Shekou Port. *Chinese Frontier Health Quarantine* **2010,**33, 112–114. Chinese.

170 Huang, Z.Y.; Wang, G.L.; Li, Y.; Zhan, Z.Q.; Zhu, Y.; Rao, D.P. Analysis on correlation between mosquito-borne disease and seasonal fluctuation of mosquito density, Baoan district, Shenzhen city, 2010-2016. *Prev Med Trib* **2017,**23, 204–205+208. Chinese.

171 Huang, C.A. Investigation and report on mosquitoes in Kashgar Prefecture. *Chinese Journal of Zoology* **1960,**3, 128–129. Chinese.

172 Huo, X.Y.; Ding, S.G.; Wang, J.; Sun, J.Y. Resistance of *Culex pipiens pallens* in Gaomi City of Shandong Province. *Chin J Hyg Insect & Equip* **2017,**23, 200–201. Chinese.

173 Ji, S.H.; Zhang, J.; Lu, C.H.; Lu, X.Y. Resistance investigation of *Aedes albopictus* to five kinds of commonly used insecticides in Yangpu District of Shanghai. *Chin J Hyg Insect & Equip* **2017,**23, 335–336. Chinese.

174 Ji, S.H.; Zhang, J.; Xu, X.; Wang, J.F.; Sun, A.G. Investigation on resistance of *Culex pipiens pallens* to four common pesticides in Yangpu District, Shanghai. *J Med Pest Control* **2013,**29, 1072–1073+1076. Chinese.

175 Jia, Y.X.; Liu, X.H.; Wang, X.X.; Liang, S.; Mao, W.S.; Zhu, H.B.; Li, H. Investigation on the distribution of mosquito in Hexi corridor in Gansu Province. *Chin J Hyg Insect & Equip* **2013,**19, 239–241. Chinese.

176 Jia, Y.X.; Wang, X.X.; Liang, S.; Liu, X.H.; Zhu, H.B.; Tian, Y.Y.; Mao, W.S.; Li, H. Survey of vectors of mosquito-borne diseases in Hexi Corridor of Gansu province, China. *Chin J Vector Biol & Control* **2014,**25, 165–167. Chinese.

177 Jiang, Y.M.; Wang, Y.L.; Yan, Z.Q.; Hu, Z.G.; Li, C.L.; Yang, Z.C. Monitoring of dengue virus from *Aedes albopictus* in Guangzhou, 2008－2010. *J Trop Med* **2012,**12, 327–328. Chinese.

178 Jiang, Y.M.; Yan, Z.Q.; Hu, Z.G.; Li, C.L. Analysis of *Aedes albopictus* density dynamics in previous dengue fever epidemic focus in Guangzhou rural area. *J Trop Med* **2012,**12, 222–223. Chinese.

179 Jiang, Y.M.; Yan, Z.Q.; Hu, Z.G.; Li, C.L.; Zhan, X.M.; Zheng, X.Y. Analysis on dengue virus from *Aedes albopictus* in Guangzhou. *Chin J Hyg Insect & Equip* **2009,**15, 121–123. Chinese.

180 Jiang, H.F.; Yao, L.X.; Hao, S.X.; Liu, Y.H.; Xu, W.; Zhong, W.C. Mosquito species and their seasonal growth and decay at Zhenjiang port. *Chin J Hyg Insect & Equip* **2007,**3, 213–215. Chinese.

181 Jiang, L.; Shi, N.; Gao, Y.F. Surveillance and Analysis of resistance to drugs of *Culex pipiens pallens* in Dalian Port from 2009 to 2010. *Port Health Control* **2016,**21, 60–62. Chinese.

182 Jiang, R.L.; Wang, C.; Cen, F. Investigation on the composition and seasonal growth and decline of mosquitoes in Benxi Iron and Steel Company. *Chin J Vector Biol & Control* **1995,**5, 373. Chinese.

183 Jiang, S.G.; Bai, B.L.; Dai, F.D.; Jiang, Z.J. Observation on the relationship between the seasonal growth and decline of *Anopheles anthropophagus* and the incidence of malaria. *Chongqing Medicine* **1987,**5, 33. Chinese.

184 Jie, B.; Hua, Y.; Wang, Q.; Hang, Z.M. Investigation report on mosquitoes in Taizhou Port. *Port Health Control* **2017,**22, 60–62. Chinese.

185 Jin, Y.M.; Wu, W.X.; Sun, L.Y.; Su, X.Y.; Jia, P.B.; Li, Z.; Lao, S.J.; Nie, S.F. Survey on the distribution of aedes mosquitoes transmitting dengue fever of Hainan Province in 2007. *China Tropical Medicine* **2008,**12, 2096–2098. Chinese.

186 Kan, N.P.; Weng, Y.W.; Lin, Q.; Chen, J.R.; You, L.B.; Wang, J.Z. Isolation and identification of dengue virus from *Aedes albopictus* in Fujian Province. *Chinese Journal of Virology* **2019,**35, 71–76. Chinese.

187 Kan, S.P. Investigation on ecological habits of *Anopheles anthropophagus* in low mountains and hilly areas of central Anhui Province. *Chin J Public Health* **1987,**6, 234–235. Chinese.

188 Kan, S.P. Investigation on breeding habits of *Anopheles anthropophagus*'s larvae in early Spring in Dabie Mountain area. *Bull Dis Control Prev* **1999,**2, 54–55. Chinese.

189 Kang, W.M.; Huang, X.Y. Distribution of *Anopheles anthropophagus* in Sichuan Province. *Sichuan Journal of Zoology* **1991,**1, 29. Chinese.

190 Kuang, J.S.; Chen, Y.B.; Liu, Y.; Chen, W.Z. Isolation of dengue serotype II virus from mosquitoes in Hainan Island. *South China J Prev Med* **1986,**4, 8–11. Chinese.

191 Lan, X.M.; Zheng, Y.T.; Dong, C.L.; Liu, Y.H.; Yin, X.X.; Yang, M.D.; Jiang, J.Y. Investigation on the resistance of *Aedes aegypti* and *Ae. albopictus* to several insecticides in Ruili city, Yunnan province. *Chin J*

Vector Biol & Control **2017,**28, 572–575. Chinese.
192 Lan, X.M.; Zhu, J.; Li, H.C.; Gao, Y.; Zou, J.H.; Tan, L.T.; Pu, J.W.; Yang, M.D. An investigation of the resistance of *Aedes aegypti* to 11 insecticides in key areas of dengue fever in Yunnan province, China. *Chin J Vector Biol & Control* **2019,**30, 582–585. Chinese.
193 Lan, C.G.; Yi, M.Y.; Liao, G.H.; Feng, X.Y.; Ou, Z.B.; Xiong, Q.M.; Tan, X.L.; Li, C.; Jiang, J.H.; Nong, L.M, et al. Surveillance report of mosquito vector in flood disaster area of Guiping City from 2006 to 2007. *Applied Prev Med* **2008,**1, 40–41. Chinese.
194 Yue, M.G. Investigation on *Anopheles anthropophagus* in Jingxian County. *J Trop Dis Parasitol* **1994,**2, 120–121. Chinese.
195 Lei, W.W.; Guo, X.F.; Fu, S.H.; Feng, Y.; Yang, Z.H.; Wang, H.Y.; He, Y.; Gao, X.Y.; Lv, Z.; Zhou, H.N, et al. Investigation of mosquitoes and arboviruses in the border areas of Yunnan province, 2012. *Chinese J Exp Clin Virol* **2017,**31, 311–314. Chinese.
196 Lei, X.T. A new species of *Aedes* (Diptera: Culicidae). *Sichuan Journal of Zoology* **1989,**1, 6–8. Chinese.
197 Lei, X.T.; Yang, C.W.; Lu, B.L. A brief account of the new record of *Aedes christophersi* in China. *Sichuan Journal of Zoology* **1984,**3, 8–9. Chinese.
198 Lei, X.T.; Yang, C.W.; Yin, Z.C.; Wu, Y.F. New records of mosquitoes from Sichuan Province. *Sichuan Journal of Zoology* **1986,**3, 36–37. Chinese.
199 Li, Q.F. Investigation on the density of *Aedes* before and after environmental transformation at Huanggang Port. *Chinese Frontier Health Quarantine* **1999,**4, 197–198. Chinese.
200 Li, X.Y.; Li, C.Q.; Zhang, S.J.; Chen, Y.W.; Tian, L.L.; He, J.; Wang, Q.Y. Epidemiological analysis on mosquito monitoring from 2009 to 2011 in Shunyi district, Beijing. *Chin J Vector Biol & Control* **2012,**23, 472–473+476. Chinese.
201 Li, X.Y.; Zhang, S.J.; Zhao, X.; Li, C.Q.; Wang, X.M.; He, Z.Y.; Tian, L.L.; He, J.; Pang, X.H.; He, X, et al. Edipemiological analysis of mosquito monitoring around the Capital International Airport in Beijing, China. *Chin Prev Med* **2013,**14, 86–88. Chinese.
202 Li, A.M.; Yan, Q.Y.; Su, Y.P.; Li, D.F. Investigation on the Distribution of *Anopheles anthropophagus* in Southern Henan Province. *Journal of Pathogen Biology* **1999,**1, 56. Chinese.
203 Li, B.S.; Xu, R.M.; Li, Z.C.; Lu, B.L. A brief report on the investigation of mosquitoes breeding in bamboo tube in Changning Bamboo Forest region of Sichuan Province. *Sichuan Journal of Zoology* **1983,**1, 30–31. Chinese.
204 Li, C.T.; Wang, L.Y.; Wu, X.; Lang, Y.; Yan, L.; Liu, C.M. Preliminary report on the distribution of *Aedes albopictus* in Shenyang. *Chin J Vector Biol & Control* **2010,**21, 291. Chinese.
205 Li, C.M.; Dong, X.S.; Yang, M.D. Geographical distribution and seasonal variations of *Aedes aegypti* in Yunnan province. *Chin J Vector Biol & Control* **2018,**29, 394–396+399. Chinese.
206 Li, F.L.; Huang, S.J.; Wang, J.X.; Liu, B.; Chen, Y.S.; Li, Z.L.; Zhao, Y.X. Investigation on mosquito vectors in 5 pig farms in Liangkou Town, Conghua district, Guangzhou province. *Animal Science Abroad (Pigs and Poultry)* **2019,**39, 68–72. Chinese.
207 Li, G.T.; Fu, H.; Guo, Y.H.; Shen, M.X.; Ren, X.M.; Li, H.L.; Ding, X.Q. Geographical distribution of *Aedes albopictus* in the Yangtze River basin in Gansu province. *Chin J Vector Biol & Control* **2010,**21, 248–249. Chinese.

208 Li, H.D.; Duo, J.Z.M.; Zhuo, M.Y.J.; Zha, X.Z.M.; Zhang, R.; Yang, X.D.; Yang, G.R.; Wang, J.; Zhou, H.N.; Gong, Z.D., et al. Taxonomy and fauna of Culicidae in Tibet Autonomous Region, China VII. First discovery of *Aedes* subgenus and species in Tibet. *Chin J Vector Biol & Control* **2019,**30, 554–556. Chinese.

209 Li, H.L.; Yuan, J.L.; Zhang, G.L.; Zheng, C.; Yin, X.P.; Sun, X.; Liu, X.M. Horizontal pattern and correlation with meteorological factors on mosquito diversity in the arid wetlands in north of Xinjiang Uygur Autonomous Region. *Chin J Vector Biol & Control* **2015,**26, 458–463. Chinese.

210 Li, H.C.; Shi, J.; Sun, X.D. Distribution of *Aedes* mosquitoes in residential areas of Lincang in Yunnan province, China. *Chin J Vector Biol & Control* **2019,**30, 472–474+480. Chinese.

211 Li, H.X.; Chen, G.W. Study on malaria control strategies in the malaria epidemic areas transmitted by *Anopheles anthropophagus* in Yunnan province. *Chin J Vector Biol & Control* **2009,**20, 569–572. Chinese.

212 Li, J.; Zhou, D.G.; Hu, S.X.; Liu, B. Dengue virus carriage and insecticide resistance of *Aedes albopictus* in Xiaolin Town of Cixi City. *Chin J Hyg Insect & Equip* **2019,**25, 527–530. Chinese.

213 Li, J.Z.; Kui, L.Y.; Li, Y.Q.; Liu, J.J.; Shi, X.Q. Preliminary investigation on mosquito species in room in Zhongshan. *Journal of Southwest Medical University* **1981,**4, 85. Chinese.

214 Li, J.S. Mosquitoes density and seasonal fluctuations monitoring analysis in provincial vector biological observation point of Luzhou city in 2014. *J Med Pest Control* **2015,**31, 875–877. Chinese.

215 Li, J.L.; Zhu, G.D.; Zhou, H.Y.; Tang, J.X.; Yang, G.J.; Cao, J. Investigation on population and density of *Anopheles* mosquitoes in Jiangsu province. *Chin J Vector Biol & Control* **2018,**29, 47–49+52. Chinese.

216 Li, L.D.; Liang, J.H.; Zhen, J.B.; Ceng, Q.S. Study of population density and seasonal fluctuation of mosquito in urban area of Xinhui district in 2014. *China Modern Medicine* **2015,**22, 161–163. Chinese.

217 Li, M.Q.; Liu, B.; Luo, L.; Wu, H.X.; Liu, Y.; Deng, X.L.; Cheng, L.Y.; Hu, G.F.; Chen, Q.; Yu, S.Y., et al. The application of *Aedes* traps for the mosquito monitoring in community. *J Trop Med* **2012,**12, 1260–1263. Chinese.

218 Li, M.B.; Yi, Y.H.; Shi, Y.F.; Li, M.G.; Liu, C.F.; Hu, J.S.; Cui, X.D.; Zhu, W.H. Preliminary investigation on ecological habits and malaria transmission of *Anopheles anthropophagus* in the foothills of Dahong Mountain in northern Hubei Province. *J Med Pest Control* **1989,**4, 36–39. Chinese.

219 Li, M.X.; Liang, Z.T.; Chen, S.C.; Zhang, J.Z. Observation on Ecological habits and Control effect of *Anopheles minimus* in Daxiqiao area, Baisha County, Hainan Island. *South China J Prev Med* **1983,**2, 72–77. Chinese.

220 Li, N.; He, Y.W.; Meng, J.X.; Wang, J.L. Isolation and identification of Mangshi virus of Seadornavirus from mosquitoes collected in Guangdong Province, China. *Chinese Veterinary Science* **2020,**50, 582–588. Chinese.

221 Li, P.; Shang, Y.Y.; Zhuang, J.A.; Li, A.M. Investigation on the distribution of *Anopheles anthropophagus* in Henan Province. *Parasitoses and Infectious Diseases* **2001,**1, 37–38. Chinese.

222 Li, P.; Wang, L.Y.; Cao, G. Mosquito monitoring from 2014 to 2017 in Wujin District of Changzhou City. *Chin J Hyg Insect & Equip* **2019,**25, 566–568. Chinese.

223 Li, S.G.; Ceng, L.H.; Liang, Q.C. Investigation on resistance of *Anopheles dirus* to DDT in Hainan province. *Hainan Med J* **1999,**3, 140. Chinese.

224 Li, S.J.; Ma, Y.C.; Guo, P.; Wang, R.; Zhang, J.; Ma, Z.W.; Rao, H.X. Investigation on the mosquito species and distribution of breeding sites in Minhe county of Qinghai province. *Chin J Vector Biol & Control* **2018,**29, 499–501. Chinese.

225 Li, S.Q.; Zhang, J.X.; Gong, Z.D.; Li, C.L.; He, Z.Y.; Yang, G.C.; Li, D.G.; Li, Z.H. The composition and distribution of mosquitoes (Culicidae) in residential area of Nujiang prefecture, Yunnan province. *Chin J Vector Biol & Control* **2008,**3, 184–188. Chinese.
226 Li, T.T.; Qiu, D.Y.; Chen, J.; Liu, D.X.; Wei, X.Y.; Yue, Q.Y. *Mansonia bonneae*, a China non-recorded species collected at Zhongshan port, Guangdong. *Chinese Frontier Health Quarantine* **2020,**43, 10–13. Chinese.
227 Li, W.; Xing, L.L.; Guo, X.X.; Cheng, P.; Wang, H.F.; Wang, Z.X.; Zhang, Y.B. Investigation of relationship between distribution of river systems, density of *Anopheles* vector and malaria incidence in Shanxian county of Shandong province. *China Tropical Medicine* **2015,**15, 932–935. Chinese.
228 Li, W.J.; Cheng, P.; Liu, L.J.; Nie, S.D.; Wang, H.F.; Zhang, C.X.; Chen, Y.P. Investigation on overwintering mosquitoes in Taibaihu District, Jining City. *Chin J Schisto Control* **2016,**28, 687–688+710. Chinese.
229 Li, W.S.; Yuan, J.F.; Min, X.T. Preliminary investigation report on *Aedes albopictus* in Campus. *J South Med Univ* **1982,**4, 354–355. Chinese.
230 Li, X.H.; Peng, S.Q.; Wu, X.B.; Ceng, J.F. Surveillance report on mosquito in 2012 at Yantian port. *Chinese Frontier Health Quarantine* **2013,**36, 382–383+390. Chinese.
231 Li, Y.L.; Gao, S.W. Preliminary study on surveillance results of *Aedes* in Fuzhou port in 2011. *Quality and Technical Supervision Research* **2012,**6, 44–47+50. Chinese.
232 Liang, Q.G.; Wang, Z.Y.; Yang, Q.; Cheng, J.Z.; Wu, J.H. Resistance of adult *Culex pipiens quinquefasciatus* to four common insecticides in Guiyang City. *Chin J Endemiol* **2019,**6, 476–480. Chinese.
233 Liang, Q.G.; Wen, S.; Yang, Q.; Wu, Y.N.; Cheng, J.Z.; Zhu, R.F.; Wu, J.H. Resistance of *Culex pipiens quinquefasciatus* and *Aedes albopictus* to four insecticides in Guiyang, China. *Chin J Vector Biol & Control* **2018,**29, 30–33. Chinese.
234 Liang, W.Q.; Lin, Y.; Li, H.; Dai, H.; Tian, Z.Z.; Zou, Z.T. Resistance of *Aedes albopictus* to commonly used insecticides in Guiyang City of China. *Chin J Hyg Insect ＆ Equip* **2018,**24, 348–351. Chinese.
235 Liang, X.Y.; Xu, J.M.; Hu, Z.G.; Yan, Z.Q.; Jiang, Y.M.; Zhang, X. Analysis of production rate of female *Aedes albopictus* in Guangzhou in 2012. *Chin J Hyg Insect ＆ Equip* **2017,**23, 531–533. Chinese.
236 Lin, H.S.; Zhang, W.; Wang, S.W.; Chen, H.S. Analysis and monitoring of dengue fever vector in Leizhou city in 2006. *Chin J Vector Biol & Control* **2008,**5, 464–466. Chinese.
237 Lin, H.S.; Zhang, W.; Wang, S.W.; Chen, H.S. Results of monitoring of dengue fever vectors in Leizhou City in 2006. *China Tropical Medicine* **2008,**5, 830–831. Chinese.
238 Lin, K.S. Distribution and control of *Aedes aegypti* in Zhanjiang area. *Chinese Frontier Health Quarantine* **1996,**6, 336–338+384. Chinese.
239 Lin, K.S.; Feng, J.Q.; Wu, S.; Cai, C.L.; Cai, H.W.; Song, X.F.; Huang, Z.X.; Zhuo, W.X. Investigation report on *Aedes albopictus* and *Aedes aegypti* on Zhanjiang Port and both sides of the Waterway. *Chinese Frontier Health Quarantine* **1983,**3, 41–49. Chinese.
240 Lin, M.H.; Wu, K.C.; Chen, W.J. A review and analysis of focus outbreak of malaria in areas with *Anopheles minimus* as vector in Hainan Island. *China Tropical Medicine* **2009,**9, 805–806+933. Chinese.
241 Lin, S.P.; Chen, H.B. A new record of the genus *Culex* from China. *Acta Zootaxonomica Sinica* **1986,**1, 213. Chinese.
242 Lin, Y.F.; Lu, W.T.; Xu, G.H. Surveillance results of mosquito density in Qingcheng District of Qingyuan city

from 2011 to 2013. *China Tropical Medicine* **2014,**14, 1539–1540. Chinese.
243 Ling, C.; Wang, S.H.; Wu, Z.R.; Chen, Q.; Li, Y. Comparison of the effect of two adult mosquito monitoring methods in mosquito trapping. *J Med Pest Control* **2020,**36, 294–295. Chinese.
244 Liu, C.G.; Luo, L.; Hu, L.F.; Deng, J.T.; Ma, Z.Z.; Hou, J.; Zhang, Y. The primary investigation of mosquitoes population and density at Yunfu port. *Chin J Vector Biol & Control* **2012,**23, 246–248. Chinese.
245 Liu, F.R.; Han, N.; Zhang, Q.W.; Dong, J.; Liang, X.S. Analysis on Mosquito Monitoring and Its Influential Factors in Longgang District of Shenzhen from 2008 to 2009. *Practical Preventive Medicine* **2010,**17, 2393–2396. Chinese.
246 Liu, G.H.; He, D.L. Preliminary investigation on species and Distribution of mosquitoes in Qianjiang City. *Chin J Vector Biol & Control* **2000,**2, 102. Chinese.
247 Liu, H.K.; Sun, Y.K.; Wang, F.X.; Dan, J.S. Investigation on transmission energy of *Anopheles sinensis* for malaria in Ganyu County. *Journal of Pathogen Biology* **1995,**2, 160. Chinese.
248 Liu, H.M.; Cheng, P.; Wang, H.F.; Liu, L.J.; Huang, X.D.; Dai, Y.H.; Zhao, Y.Q.; Wang, H.W.; Gong, M.Q. Habits and insecticide resistance of *Anopheles sinensis* in Shandong province, China, 2008-2011. *Chin J Vector Biol & Control* **2013,**24, 17–18+23. Chinese.
249 Liu, J.G.; Zhu, B.L.; Feng, J. Investigation report on mosquitoes in Taizhou Port. *Chinese Frontier Health Quarantine* **2005,**5, 147–148. Chinese.
250 Liu, J.; Huang, J.H.; Xiao, X.L.; Yu, D.D.; Xiang, Y.F.; Lai, Q.Y.; Zhan, L.H. Analysis on the monitoring results of *Aedes albopictus* density in Yuexiu District. *Chin J Hyg Insect & Equip* **2017,**23, 340–344. Chinese.
251 Liu, J.H.; Yu, X.L. A study on the mosquitoes and the biting midges in Jianfengling, Hainan province. *Chinese Journal of Applied Entomology* **1995,**5, 283–285. Chinese.
252 Liu, J.C. Investigation on breeding habits and seasonal growth and decline of *Aedes flavopictus*. *Sichuan Journal of Zoology* **1985,**1, 14. Chinese.
253 Liu, J.; Gu, Y.P.; Feng, L.; Xie, B.; Zhou, Y.B. Comparison on CO2 trap and labor hour method in *Culex pipiens pallens* surveillance. *Chin J Hyg Insect & Equip* **2018,**24, 48–50+55. Chinese.
254 Liu, L.; Wu, Y.; Guan, X.H.; Ding, J.M.; Lv, B.; Ding, H.; Zhou, H.J.; Zhao, G.B.; Yang, X.B.; Gu, Q., et al. Distribution characteristics of dengue fever vector (*Aedes albopictus*) population in Hubei. *J of Pub Health and Prev Med* **2010,**21, 18–21. Chinese.
255 Liu, M.D.; Zhang, Y.; Zhang, H.J.; Tong, Y.; Liu, T.; Li, Q.H.; Zhou, X.J.; Fu, X.F.; Tian, Y.L.; Qian, K, et al. A study of the correlation between lamp trapping mosquito density and biting rate in Beijing. *Chin J Vector Biol & Control* **2019,**30, 630–633. Chinese.
256 Liu, P.; Duan, J.C.; Zheng, X.F.; Guo, H.J.; Huang, Z.Z.; Guo, Y.; Chen, H. Mosquito investigation in Beichuan county in 2009. *Modern Preventive Medicine* **2012,**39, 2024–2025. Chinese.
257 Liu, Q.M.; Hou, J.; Wei, L.Y.; Ma, M.; Zhong, J.Y.; Wu, Y.P.; Wang, J.N.; Wu, Y.Y.; Li, T.Q.; Gong, Z.Y., et al. Surveillance of insecticide resistance and density of the dengue vector *Aedes albopictus* in four prefectures of Zhejiang province, China, 2018. *Chin J Vector Biol & Control* **2020,**31, 263–267. Chinese.
258 Liu, S.Q.; Pan, H.H.; Huang, C.X.; Chen, Y.H.; Hu, G.P. Analysis of the monitoring results of *Anopheles* mosquito in Taishan 2013-2016. *J Trop Med* **2018,**18, 693–696. Chinese.
259 Liu, T.L. Investigation report on the Distribution of *Anopheles anthropophagus* in Shaoguan City. *South China J Prev Med* **1988,**4, 57–58. Chinese.

260 Liu, W.D.; Zhang, C.Y.; Chen, W.M. Investigation on the sensitivity of adults of *Anopheles sinensis* to five pesticides. *Henan J Prev Med* **1979,**4, 112–115. Chinese.

261 Liu, W.Y.; Yang, S.B.; Tang, J. Monitoring and Analysis of *Anopheles* population and density in Funing County from 2010 to 2011. *Jiangsu J Prev Med* **2013,**24, 43–44. Chinese.

262 Liu, X.P.; Zhang, F.S.; Gao, Y.; Ren, X.L. Investigation of *Anopheles* in Benxi city. *Chin J Hyg Insect & Equip* **2013,**19, 550–551+554. Chinese.

263 Liu, H.Z.; Yang, Q.R.; Zhang, J.Y.; Gong, Z.D. Surveillance of mosquitoes for Japanese Encephalitis in Eryuan County, Yunnan Province. *Chinese Journal of Zoonoses* **1993,**4, 29–31. Chinese.

264 Liu, Y.; Zhang, S.H.; Qin, Y.M.; Liang, C.N.; Lin, L.Q.; Li, J.F. An investigation of resistance of *Culex pipiens quinquefasciatus* to common insecticides in Shenzhen, Guangdong province, China. *Chin J Vector Biol & Control* **2020,**31, 362–365. Chinese.

265 Liu, Y.R. Discussion on known mosquitoes and their Geographical regionalization in Hubei Province. *J Med Pest Control* **1986,**2, 40–46. Chinese.

266 Liu, Y.C.; Li, H. Surveillance results of dengue mosquito vectors in Qijiang District of Chongqing in 2015. *Occup and Health* **2017,**33, 1261–1263. Chinese.

267 Liu, Y.W.; Li, X.Z.; Chen, C.H. Monitoring and analysis on the Northern distribution of *Aedes albopictus* and investigate the density of border. *J Med Pest Control* **2009,**25, 324–327. Chinese.

268 Liu, Z.Y.; Liu, C.F.; Jiang, B.; Liu, M.; Lin, X.Q.; Zhao, J. Surveillance on vector mosquitoes of Japanese encephalitis in Kaijiang county, Sichuan province in 2011. *Chin J Vector Biol & Control* **2012,**23, 356–358. Chinese.

269 Liu, C.F.; Qian, H.L.; Gu, Z.C.; Zheng, X.; Wu, Z.Y.; Chen, F.Q.; Shen, Y.Z. Study on malaria transmission of *Anopheles anthropophagus* in malaria area of the lower reaches of the Yangtze River. *Chin J Parasitol Parasit Dis* **1983,**4, 69. Chinese.

270 Lu, Q.C.; Li, X.X.; Wang, W.S.; Wang, Q.; Chen, G.Y.; Zhang, W.T.; Wang, W.M. Surveillance of species and density of mosquitoes after earthquake. *Chin J Vector Biol & Control* **2009,**20, 374+384. Chinese.

271 Lu, B.L.; Dong, X.S.; Wang, X.Z. Description of a new species of the subgenus *Topomyia* (Diptera: Culicidae). *Acta Zootaxonomica Sinica* **1986,**4, 406–408. Chinese.

272 Lu, H.; Hou, P.Q.; Zhao, A.H.; Zhao, Y.; Fan, A.P.; Ma, D.Z.; Wang, X.J.; Jing, X. Analysis on the diversity of mosquito species in Mount Tai scenic spots in Shandong province. *Chin J Vector Biol & Control* **2018,**29, 364–366. Chinese.

273 Lu, M.Y.; Zhang, S.G.; Ma, T.; Sun, Y.Q.; Zhang, Y.; Sun, H.M.; Li, W.; Wei, P.M. Evaluation of the risk of imported dengue and local spreading through mosquito vectors in Nanjing in 2020. *Chinese Journal of Zoonoses* **2020,**36, 720–725. Chinese.

274 Lu, R.Z.; Liu, Q.Y.; Wu, H.X.; Guo, Y.H. Zoogeographical analysis of *Culex pipiens pallens*/*Cx. pipiens quinquefasciatus* in China, 2014. *Chin J Vector Biol & Control* **2016,**27, 107–111. Chinese.

275 Lu, Z.M.; Zhang, J.M.; Gao, E.Y.; Cao, H.N.; Song, Y.Z. Observation on Reproductive Nutrition cycle of *Anopheles sinensis* in Northern Yancheng city, Jiangsu Province. *Chin J Parasitol Parasit Dis* **1984,**4, 65. Chinese.

276 Lu, Z.M.; Zhang, J.M.; Cao, H.J.; Liu, Q.M.; Ceng, Y.L.; Gao, E.Y. Investigation on the habit of blood-sucking of *Anopheles sinensis* in the north Yancheng. *Chin J Vector Biol & Control* **1996,**5, 327–328. Chinese.

277 Lu, X.; Zhang, N.; Zhang, B.H. Investigation on mosquito population and seasonal growth and decline in Tianjin Binhai International Airport in 1996. *Port Health Control* **1997,**2, 19–20. Chinese.
278 Lu, Y.L.; Wang, C.J.; Qiao, Z.D. Discovery of *Culiseta bergrothi* and *Culiseta* megaloba in Shanxi Province. *J Shanxi Med Univ* **1988,**2, 94–95. Chinese.
279 Luo, C.; Wang, H.W.; Meng, Y.P.; Yan, C.Y.; Tang, L.X. Analysis of surveillance results of adult mosquitoes in Wanzhou section of the three Gorges Reservoir area of the Yangtze River in 1998. *Chinese Journal of Preventive Medicine* **1999,**5, 297. Chinese.
280 Luo, X.F.; Xu, J.J. Investigation on Natural infection of Romanomermi, chen-zhouensis Fu in Rice Field of Libo County, Guizhou Province. *Chin J Parasitol Parasit Dis* **1998,**2, 75–76. Chinese.
281 Lv, X.H.; Cheng, H.; Guo, X.Q.; Fei, S.J.; Pang, B.W.; Lu, W.W.; Gao, P.Y.; Leng, P.E. Investigation and analysis on the overwintering of *Aedes albopictus* in the containers in Songjiang district, Shanghai, China. *Chin J Vector Biol & Control* **2018,**29, 254–258. Chinese.
282 Lv, X.H.; Guo, X.Q.; Fei, S.J.; Pang, B.W.; Leng, P.E. Surveillance of the dengue vector *Aedes* larvae in Songjiang district, Shanghai, China, 2017-2018. *Chin J Vector Biol & Control* **2020,**31, 78–82. Chinese.
283 Ma, S.F. A new species of genus *Anopheles*, subgenus *Anopheles*. *Entomotaxonomia* **1981,**4, 277–279. Chinese.
284 Ma, X.; Fan, F.N.; Lv, H.; Xu, R. Analysis of surveillance results for *Aedes albopictus* as dengue vector in Ningbo city, China 2011. *Chin J Vector Biol & Control* **2012,**23, 567–568. Chinese.
285 Mao, X.Q. Investigation report on mosquitoes and seasonal growth and decline at Fangchenggang Port. *Science of Travel Medicine* **1998,**2, 17–19. Chinese.
286 Min, J.G.; Gu, P.Q.; Sun, J.H.; Zhang, Y.P.; Gu, Z.Q. Studies on the predictive model of the meteorology at the first appearing date of *Culex tritaeniorhynchus* in spring in Shanghai. *Chin J Hyg Insect & Equip* **2004,**4, 225–228. Chinese.
287 Miu, J.W.; Liu, W.D. Study on bioclimatology and natural infection with Brugia malayi of *Anopheles sinensis* in coastal area of Zhejiang Province. *Acta Entomologica Sinica* **1962,**4, 363–370. Chinese.
288 Nong, L.H.; Chen, J.; Li, N.J.; Liang, X.Y.; Zhang, X.; Jiang, Y.M. Study on the sensitivity of *Aedes albopictus* to common insecticides in Baiyun District of Guangzhou City. *J Med Pest Control* **2018,**34, 566–569. Chinese.
289 Nong, C.H.; Cai, K.X.; Liang, L. Analysis of surveillance results of mosquito vector of JEV in Jingxi City from 2014 to 2015. *Applied Prev Med* **2016,**22, 256–257+260. Chinese.
290 Pan, B.; Liu, Y.Y.; Zhu, T.H.; Zhou, H.H.; Ruan, F.; Peng, W.H.; Wu, C.G.; Wu, X.G.; Wu, J.; Guo, X.A, et al. Relationship between the distribution of *Anopheles anthropophagus* and the conditions of natural geography and social economy in Guangdong province. *Chinese Journal of Zoonoses* **2005,**1, 62–65+69. Chinese.
291 Pan, B.; Zhu, T.H.; Huang, Q.L.; Yang, Z.H.; Wu, X.G.; Ye, L.T.; Yang, W.S.; He, Q. Investigation of Malaria vectors and their positive sporozoite rate in Guangdong province. *Chin J Vector Biol & Control* **1997,**1, 43–46. Chinese.
292 Pan, S.X.; He, G.; Lv, X.G.; Liang, S.F.; Xie, Z.Y.; Chen, S.J.; Liu, S.Y.; Lan, J.L.; Tan, W.R. Investigation on mosquitoes after basically eliminating Malay filariasis in Huanjiang area in Guangxi province. *Chin J Parasitol Parasit Dis* **1985,**2, 72+76. Chinese.

293 Pan, Y.J.; Ren, Z.H.; Xu, L.G.; Dai, Y.M.; Leng, P.E. Study on mosquito-ovitrap index and Breteau index in Qingpu district. *Shanghai Journal of Preventive Medicine* **2016,**28, 528–531. Chinese.
294 Pang, B.W.; Liu, H.X.; Guo, X.Q.; Fei, S.J.; Lv, X.H. Resistance of *Aedes albopictus* larvae to commonly used insecticides in Songjiang District of Shanghai. *Chin J Hyg Insect & Equip* **2018,**24, 609–610. Chinese.
295 Pang, B.W.; Zhou, Y.B.; Lv, X.H.; Lu, W.W.; Guo, X.Q.; Fei, S.J. Investigation on the overwintering of *Culex pipiens pallens* in Songjiang urban area from 2016 to 2018. *Chin J Hyg Insect & Equip* **2020,**26, 364–366. Chinese.
296 Peng, L.L.; Jie, R.L.; Yan, H.; Su, X.H.; Li, J.; He, P.Q.; Wang, X.D.; Chen, X.G.; Li, H. Investigation on resistance of *Aedes albopictus* to five pesticides in Guangzhou in 2017. *Acta Parasitol Med Entomol Sin* **2018,**25, 206–211. Chinese.
297 Peng, W.J.; Zhou, X.F.; Gan, Z.Y.; Chen, H.B.; Luo, J.W.; Cao, H. Resistance of *Aedes albopictus* larvae to commonly used insecticides in Longhua District of Shenzhen City in 2018. *Chin J Hyg Insect & Equip* **2019,**25, 125–127. Chinese.
298 Pu, L.Y.; Chen, J.L.; Zhang, Q.H.; Zhang, C.M.; Li, Z.X.; Liu, W.; Wu, Z.K. *Aedes* surveillance and dengue fever serological survey at Sino-Vietnam Hekou-Laocai ports. *Chinese Frontier Health Quarantine* **2018,**41, 255–257+268. Chinese.
299 Pu, S.W.; Li, J.J. Survey of distribution and population density on dengue vector mosquito in Honghe prefecture, China. *Chin J Vector Biol & Control* **2017,**28, 594–596. Chinese.
300 Pu, S.W.; Li, J.J.; Rao, S.Z.; Zhang, R.S.; Tong, J.W.; Huang, Y.G. Primary study on mosquito community in Mengzi City, Honghe Prefecture, Yunnan. *China Tropical Medicine* **2017,**17, 1044–1046. Chinese.
301 Qian, H.L.; Pan, J.Y.; Wang, Z.G.; Ma, C.; Cai, X.Z.; Fu, F.H.; Fu, Z.F.; Deng, D. *Anopheles anthropophagus* found in Hainan Province. *Journal of Pathogen Biology* **1992,**2, 122. Chinese.
302 Qu, B.W.; Zhang, J.M.; Zhu, Y.P.; Huang, M.C.; Huang, X.J.; Huang, Y.Z. Resistance of *Aedes albopictus* to commonly used insecticides in Jiangmen city, Guangdong province, 2016. *Chin J Vector Biol & Control* **2017,**28, 386–389. Chinese.
303 Qu, B.W.; Zhang, J.M.; Zhu, Y.P.; Huang, M.C.; Huang, X.J.; Huang, Y.Z. Study on the seasonal dynamics and insecticide resistance of *Aedes albopictus* in Jiangmen city, China, 2015-2017. *Chin J Vector Biol & Control* **2018,**29, 77–80. Chinese.
304 Qu, B.W.; Zhang, J.M.; Zhu, Y.P.; Huang, M.C.; Huang, X.J.; Huang, Y.Z. Resistance of *Aedes albopictus* to commonly used insecticides in Jiangmen city, Guangdong province, 2018. *J Trop Med* **2019,**19, 796–800. Chinese.
305 Qu, J.; Ding, W.H.; Ye, G.T.; Zheng, X. Surveillance report on mosquitoes in Futian Free Trade area of Shenzhen city in 2006. *Port Health Control* **2008,**1, 39–41. Chinese.
306 Qu, M.Q.; Wang, Z.W.; Su, S.Z. Observation on nocturnal activities of *Culex tritaeniorhynchus* in Xinyang city, Henan Province. *Journal of Zhengzhou University (Medical Sciences)* **1982,**3, 11–14. Chinese.
307 Qu, F.Y. A new species of the genus *Uranotaenia* from China. *Entomotaxonomia* **1981,**4, 273–275. Chinese.
308 Ren, Z.Y.; Lv, J.; Zhang, J.; Ying, Z.P. Monitoring of resistance of *Culex pipiens pallens* to five pesticides in Linhai City. *J Med Pest Control* **1999,**6, 293–295. Chinese.
309 Ren, Z.Y.; Lv, J.; Zhu, Q.P.; Mao, S.H. Determination of Resistance of *Culex pipiens quinquefasciatus* to five pesticides in Zhuji City. *Zhejiang Prev Med* **1999,**7, 14–15. Chinese.

310 Shang, Y.Y.; Chen, J.S.; Li, D.F.; Li, P.; Su, Y.P.; Liu, H. Study on distribution, ecological feature and malaria transmission effect of *Anopheles anthropophagus* in Henan Province, China. *Journal of Pathogen Biology* **2007,**4, 304–306. Chinese.
311 Shao, S.Z.; Ou, Y.M. Study on blocking the spread of falciparum malaria by drug control in Liuhe County. *Journal of Pathogen Biology* **1997,**4, 80+47. Chinese.
312 She, J.J.; Sun, L.; Sun, Y.X.; Xue, M.X.; Su, H. Investigation on mosquitoes at Hancheng city of Shaanxi province. *Chin J Vector Biol & Control* **2007,**4, 279–282. Chinese.
313 Shen, D.Y.; You, X.C.; Shi, D.Y.; Zhuang, J.A.; Zhang, K.H.; Lian, S.C.; Chen, J.S.; Li, A.M.; Li, H.; Wang, S.Y., et al. A preliminary investigation on the ecological habits of *Anopheles anthropophagus* in Huaibin County, Henan Province. *Henan J Prev Med* **1987,**3, 21–24. Chinese.
314 Shen, L.M.; Zhang, C.X.; Kong, Q.X.; Chen, H.; Huang, M.Y.; Wei, L.Y. Investigation on the status of Dengue virus in wintering eggs of *Aedes albopictus* in Xihu District of Hangzhou in 2018. *Chin J Health Lab Tec* **2020,**30, 1121–1123. Chinese.
315 Shen, P.L.; Liao, J.L.; Zhang, Q.W. Mosquito surveillance in Longgang District of Shenzhen from 2013 to 2017. *Chin J Hyg Insect & Equip* **2020,**26, 445–448. Chinese.
316 Shen, Y.Z. Investigation on transmission factors of malaria in *Anopheles sinensis* areas in Anhui province. *Journal of Pathogen Biology* **2006,**4, 301–303. Chinese.
317 Shi, C.N.; Kai, W.L.; Liu, Q.Y.; Fan, J.H.; Zhou, H.N.; Li, H.B.; Meng, F.X. Resistance and mortality pattern of dengue vectors exposed to pyriproxyfen of dengue vectors exposed to pyriproxyfen. *Chin J Vector Biol & Control* **2017,**28, 108–112. Chinese.
318 Shi, Y.Y.; Zhang, J.M. Preliminary investigation on geographic distribution of *Aedes albopictus* in distribution of *Aedes albopictus* in. *Chin J Hyg Insect & Equip* **2012,**18, 420–421. Chinese.
319 Shi, S.Z.; Cao, J.N.; Liu, Z.J. Seasonal growth and decline of common mosquito species in Ningxia province. *J Med Pest Control* **1989,**1, 53–55. Chinese.
320 Shi, S.Z.; Liu, Z.J.; Cao, J.N. Investigation on blood-sucking *Aedes* invading stables in Pingjibao area of Yinchuan city. *Chinese Veterinary Science* **1989,**12, 21. Chinese.
321 Shi, Q.Q.; Cheng, P.; Tian, H.; Wang, H.F.; Guo, X.X.; Zhang, C.X.; Liu, L.J.; Kou, J.X.; Huang, X.D.; Wang, H.W, et al. Seasonal fluctuations and insecticide resistance of *Anopheles sinensis* in the Taibai Lake area, Jining City Shandong Province, 2013 - 2016. *Chin J Endemiol* **2018,**37, 501–504. Chinese.
322 Shu, G.H.; Peng, Z.Z.; Wen, X.M. *Anopheles* species and their Ecological habits in Yibin area. *Sichuan Journal of Zoology* **1986,**2, 14. Chinese.
323 Shu, Y.; Zhao, X.H.; Xian, D.Q.; Liu, P.; Zheng, X.F.; Shi, Y.H. Survey on the sensitivity of *Culex pipiens pallens* in the earthquake area of Beichuan. *Chin J Hyg Insect & Equip* **2014,**20, 37–38. Chinese.
324 Song, J.Z.; Fang, Q.J. A preliminary survey of the mosquitoes of Bo-Mi district,Tibet. *Acta Entomologica Sinica* **1956,**4, 541. Chinese.
325 Song, J.Z.; Li, Z.C.; Xiong, X.R.; Liu, K.N. List and Distribution of mosquitoes in Sichuan Province. *Sichuan Journal of Zoology* **1981,**1, 1–34. Chinese.
326 Song, S.; Fu, S.H.; Li, Y.Y.; Li, X.L.; Wang, D.M.; Tian, Z.Z.; Zhou, J.Z.; He, Y.; Lei, W.W.; Wang, H.Y, et al. Investigation of mosquitoes and mosquito-borne viruses in Guizhou province, 2017. *Chin J Vector Biol & Control* **2018,**29, 428–435+461. Chinese.

327 Song, X.; Cheng, P.; Wang, H.F.; Liu, H.M.; Guo, X.X.; Liu, L.J.; Zhang, C.X.; Zhao, Y.Q.; Wang, H.W.; Gong, M.Q., et al. Resistance of *Culex pipiens pallens* to Bacillus thuringiensis and chemical insecticides. *Chin J Hyg Insect ＆ Equip* **2019,**25, 13–16. Chinese.

328 Su, L. Four newly discovered species of *Aedes* in Jilin and Heilongjiang provinces. *Acta Entomologica Sinica* **1960,**1, 103–110. Chinese.

329 Su, L. Mosquitoes in Northeast China and its adjacent areas. *Journal of Jilin University (Medicine Edition)* **1983,**1, 1–9. Chinese.

330 Su, L.; Wang, C. A new species of the genus *Toxorhynchites*. *Acta Entomologica Sinica* **1981,**3, 327–331. Chinese.

331 Su, L.; Zhang, Y.S. Description of a new species of the subgenus Ochlerotatus (abstract). *Journal of Jilin University (Medicine Edition)* **1988,**2, 151. Chinese.

332 Su, L.; Zhang, Y.S. A New species of the Genus *Aedes* (Abstract). *Journal of Jilin University (Medicine Edition)* **1989,**3, 303. Chinese.

333 Su, L.; Zhang, Y.S.; Zhang, D.C. A new recorded mosquito species of the subgenus Ochlerotatus from China. *Journal of Jilin University (Medicine Edition)* **1985,**1, 55–56. Chinese.

334 Su, S.Q.; Gu, B.X.; Cai, W.H.; Chen, D. Investigation on mosquitoes in Baoting county of Hainan Island. *J Med Pest Control* **1994,**2, 123–127. Chinese.

335 Su, S.; Qu, M.Q. Investigation report on mosquitoes in Kaifeng and Jigong Mountion of Xinyang, Henan Province. *Acta Entomologica Sinica* **1956,**2, 219–225. Chinese.

336 Su, X.Q.; Chen, H.B. Supplementary description and taxonomic discussion of *Culex szemaoensis*. *Zoological Research* **1985,**S1, 21–25. Chinese.

337 Sun, D.W.; Wang, F.; Wang, S.Q.; Hu, X.M.; Wang, G.Z.; Ceng, L.H.; Li, S.G.; Cai, H.L.; Lin, S.X.; Liu, Y., et al. Distribution of *Anopheles* mosquitoes （Diptera: Culicidae） in five cities/counties of Hainan Province. *China Tropical Medicine* **2012,**12, 160–162. Chinese.

338 Sun, Y.X.; Lv, Y.J.; Sun, L.; An, C.H.; Lv, W. Study on the susceptibility of *Culex tritaeniorhynchus* to the usual insecticides in Shaanxi Province. *Journal of Pathogen Biology* **2009,**4, 601–602+607. Chinese.

339 Sun, Y.X.; She, J.J.; Sun, L.; Ruan, C.L. Study on dividing line and influence factor of *Aedes albopictus* in Shaanxi province. *Chin J Vector Biol & Control* **2009,**20, 430–432. Chinese.

340 Tan, B.; Guo, Q.; Wang, L.B.; Qiang, W.; Hua, N.; Feng, X.F.; Jia, N.E.·.M.X.; Tian, F. Investigation on mosquitoes and arboviruses at North Xinjiang ports, 2018. *Chinese Frontier Health Quarantine* **2019,**42, 108–110. Chinese.

341 Tan, J.F. Surveillance report on mosquitoes at Fangcheng Port. *Chinese Frontier Health Quarantine* **1995,**6, 348–350. Chinese.

342 Tan, Q.L.; Guan, L.F.; Shu, J.W. Investigation on mosquitoes and pathogen carrying in Daishan County in 2017. *Jiangsu J Prev Med* **2019,**30, 210–212. Chinese.

343 Tan, Q.L.; Wang, D.T.; Han, S.Z. Surveillance results of dengue fever mosquito vector in Daishan County. *Zhejiang Prev Med* **2014,**26, 1018–1019+1022. Chinese.

344 Tan, R.Z.; Huang, P.; Li, X.W.; Qu, G.Y.; Xie, S.M.; Liu, W.M. Investigation on mosquito population and seasonal growth and decline in Baiyun Airport. *Chinese Frontier Health Quarantine* **1989,**3, 172–175. Chinese.

345 Tang, D.C.; Hu, Y.F.; Li, B.S. 16 new species of mosquitoes found in Hunan Province. *J Clin Res* **1987,**1, 50. Chinese.
346 Tang, H.J.; Chen, X.L.; Wu, X.K. A preliminary report on the Ecological observation of Common mosquito species in the suburbs of Hefei City, Anhui Province. *Acta Universitatis Medicinalis Anhui* **1960,**5, 391–397. Chinese.
347 Tang, L.Q.; Zhang, J.P.; Ling, T.K.; He, Y.X.; Song, J.Q.; Liu, X.; He, Q.H. Investigation report on mosquitoes at Wuzhou Port. *Port Health Control* **2004,**3, 23–26. Chinese.
348 Tao, X.Y.; Zhao, X.; Tu, T.T. An analysis of surveillance results of adult mosquitoes in Shapingba district,Chongqing, China, 2017-2019. *Chin J Vector Biol & Control* **2020,**31, 710–713. Chinese.
349 Teng, X.B.; Xu, X.; Yang, R.; Li, H.C.; Deng, W.; Zheng, X.X.; Jiang, J.Y. The larvae charateristics of dengue vector mosquitoes in residential area of Gengma county, Yunnan province in 2016. *Chin J Vector Biol & Control* **2018,**29, 355–357+363. Chinese.
350 Tian, B.; Ma, T.Z.; Tang, C.; Zhu, S.J.; Quan, F.; Zhang, S.J.; Li, C.Q. Investigation on *Anopheles sinensis* density and malaria parasite from 2012 to 2014 at Beijing airport, China. *Chin J Vector Biol & Control* **2015,**26, 376–378. Chinese.
351 Tian, F.; Xia, H.W.M.; Guo, Q.; Tan, B.; Dang, X.X.; Wang, L.B. Investigation on biological characteristics of mosquito vectors in Northern Xinjiang ports of Western China. *Occup and Health* **2020,**36, 379–382+386. Chinese.
352 Tian, S.J.; Cao, F.R. Investigation on mosquitoes in main residential areas of Jintie Branch Bureau. *Chin J Vector Biol & Control* **1998,**5, 83. Chinese.
353 Tian, S.J.; Cao, F.R. Investigation on the growth and decline of mosquitoes in Jinzhou Railway residential area. *Chin J Vector Biol & Control* **1999,**5, 329. Chinese.
354 Tian, W.J.; Deng, L.L.; Li, P.; Zhang, W.; Liu, Z.; Ma, L. Investigation on *Culex tritaeniorhynchus* Japanese encephalitis virus poisoned rate in Chengdu area. *J Med Pest Control* **2015,**31, 498–500. Chinese.
355 Tu, T.T.; Fang, X.F.; Ji, H.Q. Resistance of *Culex* quinquefasciatus larvae to four insecticides in Yuzhong District of Chongqing. *Chin J Hyg Insect & Equip* **2017,**23, 297–298. Chinese.
356 Tu, T.T.; Ji, H.Q.; Feng, S.Q.; Yang, X.F.; He, Y.M.; Luo, C.; Ran, Z.W.; Liu, C.H.; Liu, Y.P. A survey on susceptibility of *Aedes albopictus* larvae to four insecticides in some areas of Chongqing. *Chin J Vector Biol & Control* **2016,**27, 506–507. Chinese.
357 Wang, A.J. Isolation of dengue virus from male *Aedes aegypti*. *Hainan Med J* **1986,**3, 53. Chinese.
358 Wang, C.D. Investigation on mosquito species in Nanjiang County, Sichuan Province. *Sichuan Journal of Zoology* **1991,**4, 8. Chinese.
359 Wang, C.H.; Zhao, L.Q.; Xin, Y.J.; Huang, X.T.; Qiu, W.J.; Gao, C.Y. The Composition and Distribution of Mosquitoes in Dali University Campus. *Journal of Dali University* **2013,**12, 58–61. Chinese.
360 Wang, C.C.; Zhu, C.Q.; Wu, T.; Yun, X.Y.; Ai, Y.Y.; Tan, W.L. Discovery and identification of *Aedes* flavivirus in Haikou, Hainan province. *Chinese Frontier Health Quarantine* **2020,**43, 26–28. Chinese.
361 Wang, F.; Jiang, L.; Lu, J.L.; Yao, W. Investigation of *Aedes albopictus* resistance to commonly used insecticides in Hongkou. *Chin J Hyg Insect & Equip* **2016,**22, 513–514. Chinese.
362 Wang, F.; Lu, J.L.; Xu, J.Q.; Leng, P.E. Ultra-low-volume application of pyrethrins for the control of *Aedes albopictus* in field. *Chin J Hyg Insect & Equip* **2017,**23, 38–40. Chinese.

363 Wang, G.A.; Ma, X.; Yang, S.J.; Sun, B.; Ma, M.; Chen, X.Y.; Xu, G.Z. An analysis of the density monitoring results of *Aedes* mosquitoes in Ningbo, China, in 2017. *Chin J Vector Biol & Control* **2019,**30, 341–344. Chinese.

364 Wang, H.Y.; Guo, X.X.; Yang, Q.L.; Wang, X.J.; Kong, X.L.; Wang, H.F.; Wang, H.W.; Liu, H.M.; Gong, M.Q.; Cheng, P., et al. Investigation on the distribution of mosquitoes and the insecticide resistance of *Culex pipiens pallens* in the wetlands of southwest Shandong Province. *Chin J Hyg Insect & Equip* **2020,**26, 423–426. Chinese.

365 Wang, H.; Chen, H.B.; Liu, Q.; Shen, P.L. Analysis of mosquito-borne diseases monitoring in Longgang district of Shenzhen in 2017. *J Trop Med* **2019,**19, 244–248. Chinese.

366 Wang, J.Z.; Shang, Z.Y.; Yin, S.Q.; Wang, X.J.; Li, X.S. An analysis of the surveillance results of dengue vector *Aedes* in Tengchong, Yunnan province, from 2014 to 2017. *Chin J Vector Biol & Control* **2019,**30, 206–208. Chinese.

367 Wang, J.Y.; Lan, X.M.; Yang, R.; Jiang, J.Y.; Li, H.C.; Deng, W. Study on the resistance of *Aedes aegypti* to six common insecticides in Gengma county, Yunnan province. *Chin J Vector Biol & Control* **2017,**28, 444–446. Chinese.

368 Wang, J.X.; Jin, D.L.; Wang, G.H.; Deng, Y.Q. Investigation report on mosquitoes at Ningde Port. *Chinese Frontier Health Quarantine* **1999,**2, 90–92. Chinese.

369 Wang, L.; Hou, W.Y.; Zhang, Y.; Zhou, X.J.; Ying, H.Q. Resistance of *Aedes albopictus* larvae to three insecticides in Haidian District in Beijing from 2018 to 2019. *Chin J Hyg Insect & Equip* **2020,**26, 217–219. Chinese.

370 Wang, L.; Xie, Y.H.; Yang, Y.; Zhao, N. Surveillance report on mosquitoes at Zhengzhou Railway Port in 2014. *Port Health Control* **2015,**20, 47–50. Chinese.

371 Wang, L.L.; Liu, Y.R. Known *Anopheles* species and their relationship with Disease Transmission in Hubei Province. *Chin J Vector Biol & Control* **1996,**3, 170–173. Chinese.

372 Wang, M.; Li, H.; Wen, T. Monitoring results of mosquito density in Xindu District of Chengdu from 2017 to 2019. *Occupational Health and Damage* **2020,**35, 46–49. Chinese.

373 Wang, M.; Shang, L.H.; Gu, Z.C.; Jiang, W.K.; Zhu, J.M.; Zhao, Z.H.; Zheng, X.; You, F.H. Study on Threshold Density of *An. sinensis* for Transmission of Malaria in the Northern Anhui Province. *J Trop Med* **2007,**6, 597–599. Chinese.

374 Wang, Q.Y.; Wang, S.H.; Dong, T.F.; Wu, Z.R.; Liu, H.X.; Zhong, P.S.; Leng, P.E. Resistance of *Aedes albopictus* to deltamethrin and beta-cypermethrin in Jiading District of Shanghai. *Chin J Hyg Insect & Equip* **2020,**26, 128–130. Chinese.

375 Wang, Q.; Hou, C.X.; He, G.H.; Cheng, Y.Y.; Liang, Y.P. Monitoring on the density of dengue fever vector *Aedes albopictus* and its insecticide resistance in Dianbai District of Maoming City in 2017 and 2018. *Chin J Hyg Insect & Equip* **2020,**26, 322–325. Chinese.

376 Wang, R.; Liu, Q.Y.; Liu, G.X.; Li, S.J.; Ma, Y.C.; Shi, Y.; Ma, B.Z.; Ma, Z.W.; Jiang, M.X.; Guo, Y.H., et al. First discovery of *Culex vagans* Wiedemann in Ledu county, Qinghai province, China. *Chin J Vector Biol & Control* **2020,**31, 209–211. Chinese.

377 Wang, S.Q.; Wang, C.F.; Xin, Z.M.; An, M.J. Preliminary investigation on mosquitoes in Yuanqu County, Shanxi Province. *J Shanxi Med Univ* **1992,**2, 192. Chinese.

378 Wang, S.H.; Wu, Z.R.; Zhong, P.S.; Leng, P.E.; Liu, H.X. Distribution of *Aedes albopictus* and its population density monitoring in Jiading District of Shanghai. *Shanghai Journal of Preventive Medicine* **2018,**30, 640–644. Chinese.
379 Wang, S.C.; Ding, J.; Zhang, J.B.; Wang, X.L.; Liu, C.M.; Han, L.S. The distribution of *Aedes albopictus* in the northern of Liaoning province. *Chin J Vector Biol & Control* **2009,**20, 191–192. Chinese.
380 Wang, T.Z.; Zhang, W.T.; Zhang, H.P. Survey of *Anopheles* vectors in Nanyang City from 2005 to 2017. *Chin J Schisto Control* **2020,**32, 100–102. Chinese.
381 Wang, W.M.; Zhou, H.Y.; Liu, Y.B.; Li, J.L.; Cao, Y.Y.; Cao, J. Comparison of seasonal fluctuation and nocturnal activity patterns of *Anopheles sinensis* in different regions of Jiangsu pronince. *China Tropical Medicine* **2013,**13, 292–295. Chinese.
382 Wang, X.J.; Cai, Y.C.; Ren, G.Q.; Tong, B.B.; Jia, X.Z. Surveillance of mosquitoes and their virus infection in Wudu district, Longnan,Gansu province, China, 2017-2019. *Chin J Vector Biol & Control* **2021,**32, 188–192. Chinese.
383 Wang, X.D.; Li, H.; Yu, L.; Li, W.P. Efficacy of mosquito magnet trapping mosquitoes. *Chin J Hyg Insect & Equip* **2014,**20, 32–33. Chinese.
384 Wang, X.X.; Feng, C.Y. *Uranotaenia loshanensis* and description of male and larvae of *Uranotaenia hebes*. *Acta Zootaxonomica Sinica* **1965,**3, 206–210. Chinese.
385 Wang, X.Y.; Zhang, J.T.; He, H.G.; Wang, T.; Zhang, B.; Liu, S. Mosquito density monitor in Changping District of Beijing in 2018. *Chin J Hyg Insect & Equip* **2020,**26, 45–47. Chinese.
386 Wang, X.Z.; Du, Z.W.; Lu, Y.R.; Huang, R.; Zhu, G.J.; Gu, Y.A.; M, A.S.A.H.I.R.O.T.A.K.A.G.I.; Y, O.S.H.I.O.T.S.U.D.A. Bionomics of *Anopheles minimus* and Its Role in Malaria Transmission in South Parts of Yunnan. *Chin J Vector Biol & Control* **1999,**5, 343–346. Chinese.
387 Wang, Y.X.; Zhao, D.J.; Li, G.S.; Fan, W.D. Survey of adult mosquitoes and mosquito-borne diseases in Yucheng District of Yaan City, 2007-2013. *J Prev Med Inf* **2015,**31, 623–626. Chinese.
388 Wang, Y.X.; Liu, J.Y. Investigation on the distribution of mosquitoes and nocturnal blood sucking in Changle City. *J Med Pest Control* **1996,**2, 33–34. Chinese.
389 Wang, Y.A.; Zhang, X.Z. Preliminary investigation on mosquito species in the Yellow River Delta. *Chin J Vector Biol & Control* **1999,**5, 339. Chinese.
390 Wang, Y.S.; Ding, Z.S. Investigation and study on *Anopheles* of transmission malaria in Yiyang City. *Practical Preventive Medicine* **1996,**4, 252. Chinese.
391 Wang, Z.Y.; Liang, Q.G.; Yang, Q.; Chen, L.L.; Zhang, X.; Hu, R.; Cheng, J.Z.; Wu, J.H. A premilinary investigation of symbiotic bacteria (Wolbachia) and the WO phage infecting *Aedes albopictus* in Guizhou province. *Journal of Pathogen Biology* **2020,**15, 69–73+77. Chinese.
392 Wang, Z.G.; Wang, S.Q.; Xiao, Y.Y.S.; Gao, M.Z.Y.; Jin, T.L.F.; Ceng, L.H.; Wu, Q.H.; Lan, X.H.; Guo, R.N.; Liang, Q.C, et al. Investigaton on *Ae. aegypti* and *Ae. albopictus* in the north-western part of Hainan Province. *China Tropical Medicine* **2005,**2, 230–233. Chinese.
393 Wang, Z.G.; Song, H.Y.; Li, B.R. Distribution and Epidemiological significance of mosquitoes in Chifeng City. *J Med Pest Control* **2007,**12, 945. Chinese.
394 Wei, Q.M.; Xian, H.; Zhou, D.X.; Duan, L.H.; Ou, Y.; Feng, J. Density monitoring of mosquitoes, flies, mice and cockroaches in Mianzhu, 2009-2011. *Occupational Health and Damage* **2013,**28, 22–25. Chinese.

395 Wei, X.Q.; Liu, T.; Zhou, X.J.; Li, Q.H.; Zhang, Y. Investigation of mosquito-vector density and West Nile Virus carrying status in Beijing. *Chin J Hyg Insect & Equip* **2015,**21, 582–586. Chinese.

396 Wen, X.M.; Wang, M.Q. Determination of Resistance of *Anopheles sinensis* in Luxian County. *Sichuan Journal of Zoology* **1983,**2, 42. Chinese.

397 Wen, X.M.; Xiang, B.C.; Yue, J.H.; Shu, G.H.; Yan, J.C.; Cheng, Z.L.; Zou, Y.D. Observation on Ecological habits of *Anopheles anthropophagus* in Yibin area of Sichuan Province. *Sichuan Journal of Zoology* **1992,**3, 33. Chinese.

398 Wu, X.S.; Yu, Q.H. Distribution of mosquitoes in Jiuquan city. *Chin J Vector Biol & Control* **1995,**6, 472. Chinese.

399 Wu, X.; Zhou, B.; Wang, H.M. The susceptibility of *Culex pipiens pallens* larvae against the five common insecticides in Shenyang city, China. *Chin J Vector Biol & Control* **2015,**26, 534. Chinese.

400 Wu, Y.H.; Xia, Y.P.; Zhou, G.D. Analysis of surveillance results of *Aedes albopictus* in Fenghua City in 2011. *Shanghai Journal of Preventive Medicine* **2012,**24, 457–459. Chinese.

401 Wu, Y.Y.; Ling, F.; Gong, Z.Y. Surveillance for mosquito density and species in Zhejiang, 2011-2013. *Chin J Parasitol Parasit Dis* **2015,**30, 497–500. Chinese.

402 Wu, Z.X.; Chen, J.H.; Ceng, L.; Huang, B.; Xiao, H.M. Investigation report on mosquitoes in Qisha Port. *Chinese Frontier Health Quarantine* **1998,**3, 160–162. Chinese.

403 Xia, S.D.; Wang, X.F.; Xu, X.M.; Gu, W.X.; Zheng, B.X.; Yao, X.G.; Fan, A.Q.; Li, L.D. Summary of pilot work on mosquito control in Caoyang street. *Shanghai Journal of Preventive Medicine* **1992,**7, 45–47+46. Chinese.

404 Xia, X.S.; Xu, D.W.; Li, G.D. Survey on mosquitoes at Mawan port, Shenzhen, 2016. *Chinese Frontier Health Quarantine* **2017,**40, 269–271. Chinese.

405 Xia, Y.; Liu, H.X.; Shang, H.; Li, Y.; Yin, W.S.; Cai, E.M. Resistance surveillance of *Aedes albopictus* to six commonly used insecticides in Changning District of Shanghai. *Chin J Hyg Insect & Equip* **2016,**22, 545–546+549. Chinese.

406 Xia, Y.; Yin, W.S. Resistance surveillance of field *Culex pipiens pallens* to four commonly used insecticides in Changning. *Chin J Hyg Insect & Equip* **2015,**21, 578–579. Chinese.

407 Xiang, B.C. Observation on overwintering of *Culex pseudovishnui* in Luopei Township, Gongxian County. *Sichuan Journal of Zoology* **1987,**4, 41. Chinese.

408 Xiao, P.P.; Li, C.H.; Zhang, J.Y.; Han, J.C.; Guo, X.F.; Zhou, H.N.; Lu, H.J.; Jin, N.Y. Detection and Phylogenetic Analysis of Japanese Encephalitis Virus Carried by Mosquitoes in the Southern Area of Yunnan Province. *China Animal Health Inspection* **2018,**35, 85–89. Chinese.

409 Xie, H.Q.; Huang, J.Y.; Wei, K.C.; Su, W. Seasonal distribution of *Aedes albopictus* in Nanning airport, Guangxi. *Chinese Frontier Health Quarantine* **1983,**3, 18–22. Chinese.

410 Xie, L.; Lin, A.Q.; Wang, J.; Wu, C.; Luo, Y.; Yu, A.S.; Chen, R.; Yang, B.; Xie, X.L.; Li, C.M, et al. An investigation of mosquitoes and mosquito-borne pathogens in the southwest border regions of Yunnan province, China. *Chin J Vector Biol & Control* **2019,**30, 264–267. Chinese.

411 Xie, R.H.; Zhang, X.F.; Zhu, H.P.; Xu, F.; Yao, P.P.; Cheng, Y.K.; Zhang, Y.J.; Zhu, Z.Y. The distributions of mosquito vectors carrying Japanese encephalitis virus in Zhejiang province. *Chinese Journal of Preventive Medicine* **2009,**4, 282–286. Chinese.

412 Xie, Y.M.; Zhou, S.Q.; Luo, W.F. Monitoring results of population size and density of *Anopheles* mosquito in Guangning County. *Occup and Health* **2017,**33, 1253–1256. Chinese.
413 Xing, J.L.; Yang, G.Q.; Guo, T.H.; Guan, Z.J.; Pei, G.Z. List and Distribution of mosquitoes and flies in Yantai city. *J Med Pest Control* **1994,**4, 260–263+275. Chinese.
414 Xiong, J.F.; Tian, J.H.; Yao, X.; Tan, L.F.; Yang, R.; Peng, Q.H. Population density and insecticide resistance of *Culex pipiens quinquefasciatus* in Wuhan, 2014. *Chin J Vector Biol & Control* **2016,**27, 32–34. Chinese.
415 Xiong, M.H.; Zou, Y.D.; Xu, L.N. Experimental study on haemophilic habits of mosquito vectors in Sinan and Libo counties, Guizhou Province. *Chin J Vector Biol & Control* **1997,**2, 164. Chinese.
416 Xu, B.H. A preliminary investigation on mosquitoes in Wuyi Mountain (Diptera: Culicidae). *Wuyi Science Journal* **1984,**4, 205–208. Chinese.
417 Xu, B.H. Investigation on mosquitoes in Fujian Province (Diptera: Culicidae). *Wuyi Science Journal* **1991,**8, 73–84. Chinese.
418 Xu, B.H.; Lin, Z.G. Species and breeding habits of Mosquito vector in Nanping City. *Wuyi Science Journal* **1992,**0, 315–320. Chinese.
419 Xu, B.H.; Lin, Z.G. Vertical distribution of *Aedes albopictus* and characteristics of mosquito community in Nanping bamboo forest area. *Chin J Vector Biol & Control* **1992,**1, 7–10. Chinese.
420 Xu, J.W.; Xu, C.C.; Sun, F.Z.; Wang, R.T. Investigation of mosquitoes in Lanshan Port. *Chinese Frontier Health Quarantine* **1994,**S5, 167–169. Chinese.
421 Xu, J.J.; Zhou, G.R.; Wang, X.L.; Yao, S.L.; Li, H.Y.; Li, Z.Q. Investigation on the Geographical Distribution characteristics of *Anopheles anthropophagus* in Guizhou Province. *Guizhou Medical Journal* **2002,**9, 795. Chinese.
422 Xu, J.; Zhong, W.C.; Jiang, H.F. Mosquito population and seasonal fluctuation investigation in Zhenjiang port. *Chin J Hyg Insect & Equip* **2009,**15, 156–158. Chinese.
423 Xu, N.Z.; Fan, L.X.; Mu, Z.H.; Xia, Z.H.; Gao, Y.F.; Liu, Y.H.; Guo, R.Y. Insecticide resistance of *Culex pipiens pallens* to the commonly used insecticides in Hohhot city and the surrounding areas, Inner Mongolia autonomous region, China. *Chin J Vector Biol & Control* **2017,**28, 182–184. Chinese.
424 Xu, P.; Hua, S.J. Investigation on resistance of *Culex pipiens pallens* in Hebei District of Tianjin in 2016. *Port Health Control* **2020,**25, 61–63. Chinese.
425 Xu, P.; Liu, F.X.; Ma, Y.; Gao, H.C.; Li, J.Y.; Chen, Q.M. Observation on the Growth of *Aedes* Vexans in Late Autumn in Tianshui City. *Port Health Control* **2020,**25, 61–62. Chinese.
426 Xu, R.; Li, B.; Zhou, Z.Y.; Yin, J.C.; Xie, Q. Results of monitoring of malaria vectors in Jinggu county from 2007 to 2012. *China Tropical Medicine* **2013,**13, 1116–1118. Chinese.
427 Xu, J.J.; Luo, X.F. A new species of anopheles hyrcanus group from Nei Mongol autonomous region (Diptera: Culicidae). *Chin J Parasitol Parasit Dis* **1998,**1, 48+55+49–50+52+54. Chinese.
428 Xu, J.J.; Yu, Y.; Wang, S.P.; Ni, Z.G. Discovery of *Culex modestus* inatomii Kamimura et Wada in Rongcheng City, Shandong Province. *Chin J Parasitol Parasit Dis* **1993,**1, 59–61. Chinese.
429 Xu, R.M.; Li, B.S.; Zhang, J.S.; Nie, Z.H.; Lu, B.L. Investigation on breeding characteristics of *Aedes albopictus* in villages of bamboo forest area. *J Med Pest Control* **1991,**3, 178–180. Chinese.
430 Xu, W.L.; Huang, S.H.; Zheng, Z.P.; Shi, Y.Q. Investigation on seasonal growth and decline of mosquitoes in Shantou Port. *Chinese Frontier Health Quarantine* **1994,**S2, 60–62. Chinese.

431 Xu, W.P.; Wu, X.; Wang, B.; Shao, L. Surveillance report on mosquitoes at Gongbei Port of Zhuhai in 2013. *Port Health Control* **2015,**20, 55–57. Chinese.
432 Xue, J.; Zhou, P.F. A preliminary report on the investigation of mosquitoes and midges in Laoshan area of Yunnan Province. *Journal of Pathogen Biology* **1990,**1, 84–85. Chinese.
433 Xue, W.H.; Mo, D.H.; Liu, L.J.; Zhu, M.H. Surveillance of mosquito in Q Town in Shanghai from 2009 to 2016. *J Prev Med Inf* **2018,**34, 406–409. Chinese.
434 Xue, Z.J.; Wang, J.; Song, X.P.; Li, D.M.; Zhao, N.; Yan, D.M.; Liang, W.Q.; Zhou, J.Z.; Wang, D.; Zhang, R.L, et al. An investigation of mosquitoes and mosquito-borne viruses in different regions of Guizhou province, China, in 2018. *Chin J Vector Biol & Control* **2019,**30, 259–263. Chinese.
435 Yan, X.J.; Zhao, W.; Ceng, L.H. Study on seasonal growth and decay of *Culex tritaeniorhynchus* and its influence in Sanya and Qiongzhong. *Modern Preventive Medicine* **2012,**39, 1768–1769+1772. Chinese.
436 Yan, X.J.; Zhao, W.; Ceng, X.J.; Ceng, D.H.; Chen, T.Y. Investigation on seasonal growth and decline of Japanese Encephalitis vector, *Culex tritaeniorhynchus* and infection with JEV in host animals in southwest Hainan Province. *Chin J Vector Biol & Control* **2008,**5, 470–471. Chinese.
437 Yang, F.; Tian, Z.A. Investigation on vector energy of *Anopheles sinensis* in malaria transmission in Yanhe County, Guizhou Province. *Bull Dis Control Prev* **1994,**2, 58–59. Chinese.
438 Yang, D.J.; Fu, S.H.; Zhang, H.L.; Yang, W.H.; Feng, Y.; Wang, J.L.; Zhang, Y.Z.; Wang, P.Y.; Chen, W.X.; Liang, G.D., et al. vestigation of mosquitoes and mosquito-borne viruses in northeast Yunnan province. *Chin J Vector Biol & Control* **2011,**22, 304–308+312. Chinese.
439 Yang, G.; Shao, M.F.; Li, D.B.; Yu, H.T. Threshold of *Anopheles sinensis* in malaria transmission in Shuangliu County. *Parasitoses and Infectious Diseases* **2013,**11, 26–28. Chinese.
440 Yang, J.L.; Li, F.H.; Chen, H.B. A new species of *Culex* from Yunnan (Diptera: Culicidae). *Acta Zootaxonomica Sinica* **1993,**2, 225–228. Chinese.
441 Yang, J.M. Investigation report on mosquitoes in Zhaotong city, Yunnan Province. *J Med Pest Control* **1992,**4, 232–235. Chinese.
442 Yang, J.S.; Liu, Z.Y.; Wu, T.C. Investigation and study on *Anopheles sinensis* species in Zhaotong city. *J Med Pest Control* **1997,**4, 208–210. Chinese.
443 Yang, J.P.; Hu, D.Y.; Li, C.Y.; Xiong, Y.P. Observation on Ecological habits of *Anopheles anthropophagus* and *Anopheles sinensis* in Hengshan district of Yibin County. *Sichuan Journal of Zoology* **1986,**1, 39. Chinese.
444 Yang, K.K. Investigation on malaria protozoa rate and vector *Anopheles* in Gejiu City. *Modern Preventive Medicine* **1995,**1, 34. Chinese.
445 Yang, M.D.; Jiang, J.Y.; Zheng, Y.T.; Zhou, H.N. Distribution survey on *Aedes aegypti* in the border areas of Yunnan province, China. *Chin J Vector Biol & Control* **2015,**26, 406–408. Chinese.
446 Yang, M.L.; Li, Y.M.; Li, K.L.; Chen, H.Y.; Peng, H.; Ma, Y.J. Study on sensitivity and knockdown resistance mutation of five *Anophele*ses to deltamethrin and DDT in Hainan Island. *Acta Parasitol Med Entomol Sin* **2019,**26, 99–104. Chinese.
447 Yang, P.P.; Li, W.J.; Cheng, P.; Guo, X.X.; Wang, H.F.; Wang, H.W.; Zhang, C.X.; Kou, J.X.; Liu, L.J.; Gong, M.Q., et al. Investigation on mosquito density and the natural infection of mosquitoes with Japanese encephalitis virus along the south-north water diversion project in Shandong. *Chin J Vector Biol & Control*

2016,27, 232–234+240. Chinese.
448 Yang, Q.M.; Bian, D.X.; Shi, J.; Yang, J. Distribution and characteristics of distribution area of *Anopheles anthropophagus* in Jingzhou city. *Journal of Pathogen Biology* **1989,**1, 48–49. Chinese.
449 Yang, T.X.; Wang, J.; Wang, X.H. Investigation of mosquito species and mosquito-borne arboviruses in Haikou, China. *Chin J Vector Biol & Control* **2013,**24, 254–256. Chinese.
450 Yang, W.; Kang, W.M.; Lei, X.T.; Tang, S.G.; Feng, S.Z.; He, M.Z.; Li, Z.C. Investigation on mosquito vector density in the suburbs of Chengdu city. *Parasitoses and Infectious Diseases* **1997,**2, 65. Chinese.
451 Yang, W.; Xu, G.J.; Chen, H.L.; Yan, J.C.; Feng, S.Z.; Liu, S.P.; Xu, Z.Z. Investigation of impact of the ecologic environmental and social economic factors on malaria in areas with *Anopheles anthtopophagus* as vector in Sichuan. *China Tropical Medicine* **2003,**1, 86–88. Chinese.
452 Yang, X.Y.; Liu, C.; Chen, X.W.; Cai, F.; Lin, Y.; Zhong, W.B.; Liu, Y. Resistance to 3 kind of pyrethroid insecticides in *Culex pipiens quinquefasciatus* from Haikou. *China Tropical Medicine* **2019,**19, 1092–1094. Chinese.
453 Yang, Y.M.; Zeng, X.G.; Yang, M. Fauna of mosquitoes and blackflies (Diptera: Culicidae and Simuliidae) in the Caohai National Reserve,Weining county, Guizhou province,China. *Journal of Guizhou Medical University* **2018,**43, 12–15. Chinese.
454 Yang, Y.Y.; Li, M.Z. Analysis of surveillance data on adult mosquito population density in Baoshan district of Shanghai, China, 2009-2012. *Chin J Vector Biol & Control* **2014,**25, 584–586. Chinese.
455 Yang, Y.L.; Lin, K.M.; Zheng, C.J.; Lv, X.H. Surveillance results of malaria vectors *Anopheles* in Longlin Gezu Autonomous County of Guangxi, 2014-2018. *China Tropical Medicine* **2020,**20, 356–358. Chinese.
456 Yang, Y.; Zhou, L. Analysis of Mosquito Density in Qingyun Spectrum Area of Nanchang City from 2015 to 2018. *Medical Information* **2019,**32, 129–130. Chinese.
457 Yang, Z.M. Investigation on seasonal growth and decline of *Culex tritaeniorhynchus*. *Chin J Public Health* **1992,**4, 203. Chinese.
458 Yao, C.Q. A new record of *Aedes* in China and a new record of mosquitoes in Hubei Province. *Sichuan Journal of Zoology* **1988,**3, 37. Chinese.
459 Ye, D.G.; Chen, J.Y.; Xu, B.H.; Xie, H.G.; Ye, Z.Y.; Huang, C.C. Study on the effect of *Anopheles anthropophagus* on malaria transmission in Fuzhou. *J Med Pest Control* **2006,**8, 549–550. Chinese.
460 Ye, H.B.; Lan, Q.J.; Su, S.M.; Feng, D.J. Analysis of surveillance results of *Aedes* in Dongxing Port area in 2011. *Applied Prev Med* **2013,**19, 55–57. Chinese.
461 Yi, X.Y.; Yu, M.H.; Tang, D.C. Some biological characteristics of *Anopheles sinensis* in Chenxian County, Hunan Province. *J Cent South Univ* **1959,**4, 25–31. Chinese.
462 Yin, G.H.; Shen, B.Q.; Ye, L.F. Surveillance of adult mosquito density in rural areas in Wuxi city from 1988 to 1992. *Chin J Vector Biol & Control* **1993,**NA, 440. Chinese.
463 Yin, X.P.; Wang, A.D.; Tian, Y.H.; Ba, T.; Zhang, J.G.; Liang, Z. First detection of Wolbachia in *Culex modestus* at Alataw port, China-Kazakhstan border. *Chin J Vector Biol & Control* **2017,**28, 117–119. Chinese.
464 You, X.C.; Su, S.Z. Investigation on *Anopheles anthropophagus* in southern Henan I. Geographical distribution of *Anopheles anthropophagus*. *Henan J Prev Med* **1985,**4, 60–65. Chinese.
465 Yu, F.X.; Wu, Z.Y.; Zhang, Y.P.; Li, L.C.; Ceng, G.J.; Xia, Z.G. Isolation of Japanese Encephalitis virus from mosquitoes in Henan Province for the first time. *Henan J Prev Med* **1994,**3, 137–138. Chinese.

466 Yu, J.; Tang, X.D.; Wang, J.; Huang, Y.; Guo, P.; Zhang, F.Q.; Qiu, W.; Zheng, Y.; Hu, X.B.; Deng, C.Y, et al. Investigation on mosquito diversity in different habitats in Tianzhu section of Qingshui River, Guizhou Province. *Medical Journal of National Defending Forces in Southwest China* **2014,**24, 1039–1042. Chinese.

467 Yu, L.; Zhao, L.J.; Chen, G.; Ma, X.K.; Shang, W. Surveillance on mosquito vector in Wuhua district of Kunming city in Yunnan province, 2016. *Chin J Vector Biol & Control* **2018,**29, 397–399. Chinese.

468 Yu, P.H.; Hu, Y.Q.; Liu, J.Y.; Huang, G.Q.; Zhang, H.X.; Chen, H.; Zhang, C.; Pei, S.J. The field investigation on the resistance of *Anopheles* in Jingshan, Anlu and Xiaochang of Hubei province. *Chin J Vector Biol & Control* **2008,**19, 500–502. Chinese.

469 Yu, Q.Y.; Lv, B.J.; Chen, W.E.; Luo, J. Analysis of mosquito vector surveillance results in Liuzhou City in 2016. *Applied Prev Med* **2017,**23, 337–341. Chinese.

470 Yuan, D.Y.; Tang, T.Y.; Shen, H.K.; Xie, L.B.; Chen, Z.H. Analysis of mosquito surveillance results in Dazhu County from 2016 to 2018. *J Med Pest Control* **2020,**36, 1045–1047. Chinese.

471 Yuan, G.L.; Chen, W.J.; Li, X.Y. Distribution and seasonal growth and decline of mosquito species in the suburbs of Jiaocheng City. *Strait J Prev Med* **2003,**6, 37–38. Chinese.

472 Yuan, M.; Gao, Y.Q.; Wang, D.C.; Zhou, X.J. An analysis of the surveillance results of mosquito vectors surrounding the terminal of Beijing Daxing International Aitport, China, 2014-2017. *Chin J Vector Biol & Control* **2020,**31, 83–87. Chinese.

473 Yuan, M.; Liu, H.B.; Gao, Y.Q.; Gan, Y.D.; Wang, D.C. The result analysis of mosquito-borne monitoring before the second Beijing Capital Airport construction. *Chin J Vector Biol & Control* **2015,**26, 622–624. Chinese.

474 Yuan, Y.C.; Wu, Z.Y.; Li, Q.B.; Wu, F.L.; Kan, S.P. Distribution of *Anopheles anthropophagus* and its transmission of malaria and filariasis in Anhui Province. *Journal of Pathogen Biology* **1991,**1, 58–60. Chinese.

475 Yun, L.; Wang, F.C.; Zhang, Q.F.; Gao, Q.H.; Wang, X.Y.; Li, S.S.; Shang, X.L.; Chao, Y.S.; Liu, Y.Y.; Dong, R.X., et al. Investigation of mosquito population density and insecticide resistance of *Culex pipiens pallens* for International Horticultural Exposition Park and surrounding area in Tangshan city. *Chin J Vector Biol & Control* **2018,**29, 351–354. Chinese.

476 Zhang, F.; Zhang, C.X.; Han, H.M. Monitoring and Application of Mosquito Distribution at the Yellow River Estuary in 2020. *Port Health Control* **2020,**25, 58–60. Chinese.

477 Zhang, H.B.; Ge, B.; Liu, H.X.; Liu, Q.; Yi, K.H.; Zhang, Y.; Huang, T. A trend analysis of the resistance of *Aedes albopictus* to commonly used insecticides in Fengxian district, Shanghai, China, 2015-2019. *Chin J Vector Biol & Control* **2020,**31, 148–151. Chinese.

478 Zhang, H.L.; Shi, H.F.; Mi, Z.Q.; Zi, D.Y.; Gong, Z.D.; Hou, Z.L.; Li, Z.X.; Zhang, Y.Z.; Zhang, J.Y.; Yang, L.P., et al. Study on Japanese Encephalitis virus isolates from four species of *Aedes* in Yunnan Province. *Virologica Sinica* **1999,**1, 33–36. Chinese.

479 Zhang, H.L.; Zi, D.Y.; Mi, Z.Q.; Gong, Z.D.; Shi, H.F.; Zhang, Y.Z.; Hou, Z.L.; Yang, L.P. Characterized Distribution of *Aedes albopictus* and Their Relation with Arbovirus in Yunnan Province. *Chin J Vector Biol & Control* **2001,**2, 103–105. Chinese.

480 Zhang, H. Preliminary investigation report on mosquitoes in Wangmo County. *Acta Entomologica Sinica* **1960,**1, 125–128. Chinese.

481 Zhang, H.J.; Ge, J.Q.; Tang, C.J.; Zhang, Z.; Liu, M.D.; Zhang, Y. Distribution and seasonality of *Culex tritaeniorhynchus* in Chaoyang district, Beijing. *Chin J Vector Biol & Control* **2016,**27, 148–150. Chinese.
482 Zhang, J.S.; Wang, D.M.; Yang, Y.S. *Culex pipiens pallens* density in Miyun District of Beijing from 2011 to 2016. *Chin J Hyg Insect & Equip* **2018,**24, 74–75+80. Chinese.
483 Zhang, J.; Lu, C.H.; Ji, S.H.; Lu, X.Y.; Zong, L.L. Resistance determination of *Aedes albopictus* larvae to four insecticides in Yangpu District of Shanghai City. *Chin J Hyg Insect & Equip* **2019,**25, 289–290. Chinese.
484 Zhang, J.H.; Yang, S.K.; Yang, Z.M.; Li, T.S.; Zheng, G.C. Investigation on the composition of *Anopheles* and the incidence of malaria in different topographic areas of Shangnan County, Shaanxi Province. *Shaanxi Medical Journal* **1990,**10, 13–41. Chinese.
485 Zhang, J.J.; Xing, D.M. Investigation on the distribution of dengue vector *Aedes albopictus* in Pingdingshan City in 2015. *Chin J Hyg Insect & Equip* **2016,**22, 163–165. Chinese.
486 Zhang, J.H.; Wang, J.Y.; Han, S.Z.; Wang, H.Q. Ecological observation of *Anopheles sinensis* on Zhoushan Island. *Chin J Public Health* **1990,**5, 270. Chinese.
487 Zhang, P.X.; Zhang, S.M. A New species of *Culex chungkiangensis* and *Aedes rhungkiangensis* in Guizhou Province. *Acta Entomologica Sinica* **1974,**3, 347–352. Chinese.
488 Zhang, P.X.; Zhang, S.M. A new record of *Culex* from China. *Acta Zootaxonomica Sinica* **1982,**2, 202. Chinese.
489 Zhang, Q.; Liu, Y.; Chang, X.S.; Li, J.B. Investigation of Mosquito Vectors in Chengdu Shuangliu International Airport from 2016 to 2018. *Port Health Control* **2019,**24, 60–62. Chinese.
490 Zhang, S.S.; Zhou, S.S.; Zhou, Z.B.; Wang, X.Z.; Jiang, W.K.; Shi, W.Q.; Yang, Y.H.; Yin, S.Q.; Li, X.S.; Wang, J.Z, et al. Investigation on population density and bionomics of *Anopheles minimus* in China-Myanmar border. *Chin J Vector Biol & Control* **2017,**28, 216–219+254. Chinese.
491 Zhang, S.L.; Li, Y.T.; Shen, Y.; Liu, S.; Hao, X.A. Investigation of species, density, seasonal fluctuations, and prevalence of the epidemic encephalitis B virus in mosquitoes in the Weishan Lake area. *Journal of Pathogen Biology* **2015,**10, 543–545+554. Chinese.
492 Zhang, S.J.; Ma, T.Z.; Li, C.Q.; Tang, C.; Tian, B.; Quan, F.; Zhu, S.J.; Li, X.Y. Surveillance on *Culex tritaeniorhynchus* from 2011 to 2013 in surrounding areas of Beijing Capital International Airport, China. *Chin J Vector Biol & Control* **2014,**25, 323–325+329. Chinese.
493 Zhang, S.J.; Ma, T.Z.; Tang, C.; Tian, B.; Quan, F.; Ji, G.Q.; Zhu, S.J.; Zhang, W.Z.; Song, S.X.; Li, C.Q, et al. Epidemiological analysis based on mosquito surveillance of species, densities and carried pathogen from 2011 to 2014 in surrounding areas of Beijing Capital International Airport, China. *Chin J Vector Biol & Control* **2015,**26, 471–474. Chinese.
494 Zhang, W.; Deng, L.L.; Liu, Z.; He, J.H.; Tian, W.J.; Jiang, Z. Emergency Control for Abnormal Increase on Density of culex tritaeniorhynchus in Chongzhou of Chengdu in 2015. *J Prev Med Inf* **2016,**32, 812–815. Chinese.
495 Zhang, W.Z. Preliminary observation on mosquito species in Pengshui County. *Chin J Vector Biol & Control* **1990,**4, 201. Chinese.
496 Zhang, X.Z.; Fu, X.Z.; Li, F.H.; Peng, X.W.; Wang, P. Observation on mosquito species and overwintering places of adult mosquitoes in Shijiu Port. *Journal of Pathogen Biology* **1989,**S1, 85. Chinese.
497 Zhang, X.Z.; Huang, K.J.; Wang, L.K.; Peng, X.W.; Fu, X.Z.; Liu, H.L. Study on the Ecological habits of

harassing *Armigeres subalbatus* in Mengshan Dawa area of Shandong Province. *Chin J Parasitol Parasit Dis* **1992,**1, 50–53. Chinese.

498 Zhang, X.; Zhao, X.; Li, Y.; Guo, H.L.; A, L. Survey on mosquitoes at Beijing Tianzhu free trade zone in 2015. *Chin J Vector Biol & Control* **2016,**27, 510–512+519. Chinese.

499 Zhang, X.R.; Cheng, J.S.; Ji, P.; Yin, G.C. Study on the seasonal growth and decline of mosquitoes and the incidence and control of mosquito-borne diseases in Tongzhou City. *J Med Pest Control* **1999,**12, 629–631. Chinese.

500 Zhang, Y.M.; Wang, S.; Hu, X.H.; Zhao, Y.; Ge, L.; Bao, L.C. Surveillance and analysis of the resistance of *Culex pipiens pallens* to commonly used insecticides in Tianjin, China, 2013-2019. *Chin J Vector Biol & Control* **2020,**31, 438–441. Chinese.

501 Zhang, Y.; Zheng, F. Background survey of mosquitoes in Fengxian District of Shanghai from 2011-2018. *Occup and Health* **2020,**36, 1251–1254. Chinese.

502 Zhang, Z.X.; Huang, R.; Xiao, Z.R. Investigation and Analytic Methods on the Community Characters of *Anopheles* Adults in the Mountainous Area of Cangyuan County, Yunnan. *Chin J Vector Biol & Control* **1998,**2, 18–21. Chinese.

503 Zhang, Z.D.; Gao, Q.; Cao, H.; Zhou, Y.B.; Leng, P.E. Mosquito population dynamics and distribution of residential areas in downtown Shanghai. *Chin J Vector Biol & Control* **2015,**26, 486–490. Chinese.

504 Zhang, Z.; Lv, W.; Yang, P.R.; Li, X.L.; Deng, F. Study on the resistance of *Culex pipiens pallens* to five insecticides in Baoji City. *Chin J Hyg Insect & Equip* **2017,**23, 522–524. Chinese.

505 Zhang, Z.K.; Chen, J.Y. Study on mosquito fauna in Liaoning Province. *Journal of Liaoning University (Natural Science Edition)* **1980,**2, 26–40. Chinese.

506 Zhang, Z.F. Investigation on mosquito species in Longling county, Yunnan Province. *Chin J Parasitol Parasit Dis* **1988,**4, 20. Chinese.

507 Zhao, D.Y.; Guo, Q.; Zhang, C.; Chen, W.J. Investigation and analysis of mosquitoes in Tianjin Binhai International Airport from 2012 to 2017. *Port Health Control* **2017,**22, 59–62. Chinese.

508 Zhao, F.; Chen, L.; Wang, J.X.; Yang, Y.; Fan, X.J. Comparison of mosquito-trapping effect of carbon dioxide lamp trapping method and perched mosquito capture method. *Chinese Frontier Health Quarantine* **2016,**39, 333–335. Chinese.

509 Zhao, G.H.; Mao, S.H. Observation on the seasonal growth and decline of adult mosquitoes in urban area in Zhuji City from 1996 to 1997. *J Med Pest Control* **1999,**9, 492–493. Chinese.

510 Zhao, J.J.; Zhu, W.G. Analysis of surveillance results of adult mosquito density in Hualong District from 2012 to 2013. *Chinese Journal of Urban and Rural Enterprise Hygiene* **2014,**29, 60–61. Chinese.

511 Zhao, Q.; Gao, L.J.; Guo, X.S.; Zhang, Y.Q.; Tang, Z.Q.; Liu, X.B.; Liu, J.Q. Analysis on surveillance results of *Aedes albopictus* larvae in Henan province in 2016. *Chin J Vector Biol & Control* **2018,**29, 358–360. Chinese.

512 Zhao, S.Q. Investigation on vector energy of *Anopheles* of transmission malaria in Longchuan area of Debao County. *Chinese Youjiang Medical Journal* **1986,**2, 27–31. Chinese.

513 Zhao, S.G.; Song, C.D.; Qiao, S.H. Ecological investigation report on *Aedes seoulensis* and mosquito species in Konglin area. *J Med Pest Control* **1986,**3, 17–20. Chinese.

514 Zheng, G.J.; Han, Z.L.; Jin, R.Z.; Qian, W.J. Investigation report on mosquito population in Tumen Port.

Chinese Frontier Health Quarantine **1994,**S1, 76. Chinese.

515 Zheng, J.Z.; Li, H.M.; Gao, Y.; Liu, C.H. Population dynamics and resistance of *Culex pipiens quinquefasciatus* to three insecticides in Xianning City. *Chin J Hyg Insect & Equip* **2017,**23, 433–435. Chinese.

516 Zheng, K.; Huang, J.C.; Li, X.B.; Hong, Y.; Shi, Y.X.; Xing, L.Q.; Xiang, D.P.; Guo, B.X.; Hu, L.F. Investigation of Arboviruses Carried by Mosquitoes at Ports in Five Provinces of South China. *Chinese Frontier Health Quarantine* **2009,**32, 181–186. Chinese.

517 Zheng, Y.Y.; Fu, S.H.; Tang, X.Y.; Li, X.Y.; Shang, S.Y.; Xu, C.; Liang, G.D. Surveillance on mosquito-borne arboviruses in Xixian county and Xin'an county, Henan province, 2012. *Chin J Vector Biol & Control* **2015,**26, 127–132. Chinese.

518 Zhou, B.; Wang, H.M.; Wang, P.; Yin, H.B.; Che, L.; Wu, X.; Lang, Y.; Liu, C.M. Investigation on resistance of *Anopheles sinensis* to four insecticides in Shenyang city. *Chin J Hyg Insect & Equip* **2015,**21, 481–483. Chinese.

519 Zhou, C.Q.; Chen, H.D.; Zhang, Y.Z. Study on the growth and decline of natural population of *Culex pipiens quinquefasciatus* in Guangzhou city. *Acta Scientiarum Naturalium Universitatis Sunyatseni* **1990,**1, 55–62. Chinese.

520 Zhou, D.C.; Zhao, F.M.; Yang, S.K.; Fang, K.P.; Shen, C.L.; Li, B. Study on the distribution and the major malaria vectors of *Anopheles* from 1989 to 2008 in Ning'er County. *Journal of Pathogen Biology* **2010,**5, 643–644. Chinese.

521 Zhou, H.Y.; Gao, Q.; Jin, X.L.; Li, J.L.; Shen, B.X.; Wang, W.M.; Zhang, X.P.; Gu, Y.P.; Cao, J. Filed investigation on environmental characteristics in areas where *Anopheles anthropophagus* distributed in Jiangsu province. *Chin J Schisto Control* **2002,**6, 442–445. Chinese.

522 Zhou, K.Q.; Ma, Z.F.; Yao, Q.G.; Chen, G.; Li, G.T. Investigation on mosquitoes in Lanzhou Airlines Port. *Port Health Control* **1999,**4, 23–25. Chinese.

523 Zhou, K.M.; Yang, M.D.; Lan, X.M.; Zheng, Y.T.; Tang, Y.R.; Wu, C.; Jiang, J.Y. An investigation of the distribution of dengue vector *Aedes* in the main urban areas of 129 counties of Yunnan province, China. *Chin J Vector Biol & Control* **2021,**32, 150–157. Chinese.

524 Zhou, L.C.; Bao, J.Y.; Wu, X.S.; Chen, X.M.; Zhu, J.S. Investigation on resistance of *Aedes albopictus* to five insecticides in Wuhan city. *Chin J Vector Biol & Control* **2017,**28, 185–187. Chinese.

525 Zhou, M.; Chen, X.L.; Zhou, Y.N.; Chen, Y.N. Mosquito surveillance at Beijing Capital International Airport port, 2016. *Chinese Frontier Health Quarantine* **2017,**40, 406–409. Chinese.

526 Zhou, P.F. Investigation on mosquito species in living rooms and cat ear holes in Laoshan War Zone. *People's Military Surgeon* **1986,**9, 21. Chinese.

527 Zhou, S.S.; Xue, J.M.; Ye, Z.M.; Li, L.Z.; Tan, J.X.; Lu, B.L. Investigation on mosquitoes in Guangxi Zhuang Autonomous region. *Acta Entomologica Sinica* **1960,**3, 307–314. Chinese.

528 Zhu, H.W.; Li, C.H.; Duan, L.S.; Liu, Y.; Wang, G.S.; Fan, K.Q.; Wang, Y.Q. Investigation on species and density of malaria vector anopheles in Chenzhou from 2013 to 2014. *Journal of Community Medicine* **2015,**13, 25–27. Chinese.

529 Zhu, M.H.; Liu, L.J.; Wu, M.; Lv, J.; Zhang, Z.W.; Liu, H.X. Distribution and population density fluctuation of *Aedes albopictus* in Minhang district, Shanghai, China. *Chin J Vector Biol & Control* **2020,**31, 321–324.

Chinese.
530 Zhu, Q.; Fu, Y.D.; Chen, Y.L.; Zhong, X.D. Species, distribution and seasonal fluctuation of *Anopheles* in Xiuying district of Haikou city. *Practical Preventive Medicine* **2019,**26, 1453–1455. Chinese.
531 Zhu, T.H.; Zhang, M.C. A preliminary investigation on the distribution and malaria transmission of *Anopheles anthropophagus* in Guangdong Province. *Chin J Parasitol Parasit Dis* **1990,**3, 64. Chinese.
532 Zhu, W.; Wang, X.; Liu, X.Y.; Zhou, Y.B. Monitoring of *Aedes albopictus* with mosq-ovitraps in Xuhui District of Shanghai. *Chin J Hyg Insect & Equip* **2020,**26, 135–138. Chinese.
533 Zhuang, H.Y.; Tao, L.B.; Ren, B.L.; Wang, X.J. Investigation report on mosquitoes in Rizhao Port. *Chinese Frontier Health Quarantine* **1995,**6, 357–358. Chinese.
534 Zhuo, M.Y.J.; Duo, J.Z.M.; Zha, X.Z.M.; Zhang, Y.M.; Yang, X.D.; Yang, G.R.; Wang, J.; Li, C.F.; Zhou, H.N.; Gong, Z.D., et al. Studies on the classification and flora of mosquitoes in Tibet Autonomous region X. Species composition and distribution of mosquitoes in Tibet. *Acta Parasitol Med Entomol Sin* **2020,**27, 129–142. Chinese.
535 Zou, Q.; Xin, Z.L. Investigation and seasonal observation on mosquito species in rooms in Guangzhou Railway area. *J Med Pest Control* **1993,**2, 79–81. Chinese.
536 Zuo, Y.H.; Zhu, J.M.; Cao, S.C.; Hu, Z.J.; Yang, W.Z.; Song, J.G.; Jiang, B.R. *Anopheles anthropophagus* found for the first time in Hongze County, Jiangsu Province. *Chin J Parasitol Parasit Dis* **1993,**2, 75–76. Chinese.
537 NA Preliminary investigation on malaria transmission of *Anopheles sinensis* in Shangqiu area, Henan Province. *J Med Res* **1975,**3, 21–24. Chinese.
538 NA A preliminary report on the discovery of the *Anopheles gigas baileyi* Edwards in Guantang Commune of Luyi County. *Henan J Prev Med* **1978,**2, 38–39. Chinese.
539 NA Investigation on *Anopheles* vector in Hainan Island in 1975. *South China J Prev Med* **1979,**1, 32–37. Chinese.
540 NA A preliminary observation on the ecological habits of *Aedes albopictus* in Boai bamboo forest area. *Henan J Prev Med* **1980,**3, 38–41. Chinese.
541 NA Monitoring of density and population composition of adult mosquitoes in rooms in Hainan Island. *Chin J Vector Biol & Control* **1994,**2, 81–84. Chinese.
542 Bi, F.Y.; Tan, Y.; Chen, M.M.; Xie, Y.H.; Dong, Y.H.; Mo, J.J.; Yang, J.Y. Study on molecular characteristics of full-length genome of Japanese encephalitis virus strains isolated in Guangxi. *Chin J Dis Control Prev* **2013,**17, 145–149. Chinese.
543 Cao, M.; Tian, Z.G.; He, Y.P.; Zhang, G.N.; Li, P. The distribution investigation and pathogens testing for mosquitoes during 2010 EXPO. *Chin J Hyg Insect & Equip* **2011,**17, 248–250. Chinese.
544 Chen, C.W.; Liu, Q.Y.; Wei, S.C.; Liu, J.Q.; Guo, Y.H.; Ren, D.S.; Zhu, L.; Liu, X.; Luo, Y.D.; Shen, Y, et al. Comparative efficacy of four adult mosquito surveillance tools in Yongcheng city. *Chin J Vector Biol & Control* **2017,**28, 526–529. Chinese.
545 Chen, H.N.; Zhou, M.H.; Ma, Z.H.; Liu, D.P.; Wu, Z.M.; Zhang, A.J.; Yang, W.F.; Liu, H.; Zhang, Y.F.; Tian, Y, et al. Identification of genotype 1 of Japanese encephalitis virus nucleic acid from mosquitoes in Suzhou city for the first time. *Chin J Viral Dis* **2016,**6, 198–201. Chinese.
546 Chen, L.; Zhao, F.; Ni, R.; Tian, L.B.; Fan, X.J.; Liu, Y. Investigation on medical vectors at Chengdu

Shuangliu International Airport, 2012-2014. *Chinese Frontier Health Quarantine* **2016,**39, 260–264+268. Chinese.
547 Chen, L.X.; He, C.Y. Monitoring analysis and control measures of the Four Pests density in Luqiao district. *Strait J Prev Med* **2007,**3, 62–63. Chinese.
548 Chen, M.H.; Lin, Y.; Qiu, E.; Tang, L.Q. Surveillance and Media investigation of Dengue Fever in Fuzhou City. *Practical Preventive Medicine* **2004,**6, 1211–1212. Chinese.
549 Chen, Y.K.; Zhong, L.F.; Lan, J.Q. Epidemiological surveillance and analysis of Malaria in Wuxue City from 2005 to 2011. *Contemporary Medicine* **2012,**18, 20–21. Chinese.
550 Chen, Z.H.; Tang, G.; Zhang, S.Y. Monitoring Analysis and Control measures of the Four Pests density in Panzhihua City. *Chin J Vector Biol & Control* **2007,**1, 64–66. Chinese.
551 Cui, W.J.; Liu, G.S.; Hu, G.C.; Wu, R.H.; He, B.H.; Luo, J.P. Epidemic characteristics of malaria and evaluation of the control measures in Shaoguan city, 1950—2014. *J Trop Med* **2016,**16, 945–947. Chinese.
552 Dai, P.F.; Zhao, J.Y.; Kong, X.S.; Liu, M.D.; Zhang, J.S.; Liu, Z.P.; Hao, X.F.; Zhang, H.Z.; Cheng, J.X. Investigation of Japanese encephalitis vectors in 2009 in Linyi county of Shanxi province, China. *Chin J Vector Biol & Control* **2014,**25, 424–426+431. Chinese.
553 Dai, Q.B.; Gai, J.H. Investigation report on vector insects and rodent at Taiping Bay Port. *Chinese Frontier Health Quarantine* **1989,**6, 335–337+343. Chinese.
554 Deng, B.; Yang, H.; Ma, J.; Fu, Q.; Ji, Y.C.; Li, H.; Li, S.L. The integrated management of vector in the national parade troop concentrated area. *Chin J Hyg Insect ＆ Equip* **2010,**16, 191–193. Chinese.
555 Deng, H.P.; Xu, Y.X.; Wang, S.H. Efficacy comparison of four kinds of mosquito capture methods. *Chin J Hyg Insect ＆ Equip* **2017,**23, 238–240. Chinese.
556 Deng, J.G.; Yu, S.L.; Nong, Z.; Lu, S.G.; Tan, L.F.; Luo, D.; Li, T.Z. Analysis of malaria prevalence in Lingyun County,Guangxi, 1951-2015. *China Tropical Medicine* **2016,**16, 803–806. Chinese.
557 Deng, S.Z.; Zhang, H.L.; Li, J.M. Distribution characteristics of mosquito and their natural infection with Japanese encephalitis virus in Yunnan province. *Chin J Vector Biol & Control* **2009,**20, 344–348. Chinese.
558 Ding, F.; Chen, H.X.; Sun, P.J.; Lu, G.F.; Ling, F.; Wu, F. Analysis of vector monitoring results in 2009-2012 in Haining city. *Chin J Vector Biol & Control* **2015,**26, 188–191. Chinese.
559 Dong, B.J.; Chen, W.Z.; Li, X.W.; Liu, H.B.; Chen, Y.B.; Kan, B.; Kuang, J.C. Isolation of two strains of chikungunya virus from mosquitoes and bats in Hainan Island for the first time. *Chin J Vector Biol & Control* **1993,**3, 47–50. Chinese.
560 Dong, T.F.; Wang, Q.Y.; Wang, S.H.; Wu, Z.R.; Yuan, H.; Liu, H.X. Investigation on mosquito infestation in the schools of Jiading District in the summer of 2019. *Chin J Hyg Insect ＆ Equip* **2021,**27, 35–38. Chinese.
561 Du, B.P.; He, Z.C. Monitoring research on mosquito in Shunde District of Foshan City from 2007 to 2011. *J Med Pest Control* **2012,**28, 1205–1206+1209. Chinese.
562 Du, B.P.; Lin, Q.; Zhou, Z.Y. Vector monitoring in Shunde district Foshan City. *Chin J Hyg Insect ＆ Equip* **2014,**20, 339–342. Chinese.
563 Du, Z.W.; Wang, P.Y.; Wang, X.Z.; Fan, B. Investigation on sensitivity base of *Anopheles minimus* to five pesticides in Yunnan Province. *J Med Pest Control* **1999,**10, 514–516. Chinese.
564 Fan, J.; Cao, H.; Zhou, Y.B.; Jiang, Q.W. Study of mosquito species, density and seasonal changes in Huangpu District of Shanghai from 2008 to 2015. *Shanghai Journal of Preventive Medicine* **2016,**28, 521–524.

Chinese.
565 Feng, J.Q.; Deng, M.C.; Si, W.; Cai, C.L. Surveillance situation of rodent and vector insects at Xuwen Port. *J Med Pest Control* **1997,**2, 29–30. Chinese.
566 Feng, X.G.; Zhang, H.L.; Gong, Z.D.; Yuan, Q.H. Isolation and Identification of the pathogen of Japanese Encephalitis during outbreak in Eryuan County, Yunnan Province. *Bull Dis Control Prev* **1999,**1, 28–29. Chinese.
567 Feng, X.; Shen, T.Y.; Liu, G.T.; Wang, X.J.; Wang, Y. *Aedes albopictus* monitoring with three different methods. *Chin J Hyg Insect & Equip* **2018,**24, 472–474. Chinese.
568 Feng, X.; Shen, T.Y. Investigation of mosquitoes and flies in Xicheng District of Beijing in 2014. *Chin J Hyg Insect & Equip* **2015,**21, 609–611. Chinese.
569 Feng, X.; Shen, T.Y.; Zhao, X.Y.; Du, X.X.; Qin, D.; Wang, X.J. Investigation and control countermeasures of vector density in Xicheng District of Beijing in 2011. *J Med Pest Control* **2012,**28, 945–948. Chinese.
570 Feng, X.Y.; Shi, W.Q.; Li, J.L.; Chen, J.S.; Li, Z.X.; Xia, Z.G. An investigation of malaria vectors in Longhui county, Hunan province. *Journal of Pathogen Biology* **2020,**15, 317–321. Chinese.
571 Feng, Y.; Fu, S.H.; Yang, W.H.; Zhang, Y.Z.; Yang, J.; Liu, Y.H.; Dong, C.L.; Li, S.; Gao, X.Y.; Jiang, W, et al. Molecular characterization of mosquito-borne viruses strains newly isolated in western Yunnan province along the border of China and Myanmar, China. *International Journal of Virology* **2014,**21, 193–198. Chinese.
572 Feng, Y.; Li, S.G.; Zhang, H.L.; Fu, S.H.; Kang, X.H.; Cun, D.Q.; Guo, C.; Zhang, Y.Z.; Yang, W.H.; Wang, H.Y, et al. Molecular characterization of Japanese encephalitis virus and Getah virus strains newly isolated in Tengchong County,Yunnan Province,China. *Chinese Journal of Zoonoses* **2014,**30, 353–357+363. Chinese.
573 Feng, Y.; Zhang, H.L.; Fu, S.H.; Li, M.H.; Wang, H.Y.; Liang, G.D. Full-genome sequencing analysis of two strains of Japanese encephalitis virus isolated from Yunnan, China. *Chin J Viral Dis* **2012,**2, 23–29. Chinese.
574 Fu, G.Q. Mosquito monitoring in Shenxian from 2016 to 2017. *Chin J Hyg Insect & Equip* **2018,**24, 482–483+487. Chinese.
575 Gao, K.; Wen, W.H.; Ma, Z.H. Insecticide resistance of main disease vectors in Changshu City in 2017. *Chin J Hyg Insect & Equip* **2019,**25, 122–124. Chinese.
576 Gao, Q.; Cao, H.; Zhou, Y.B.; Fan, J.; Xiong, C.L.; Jiang, Q.W.; Leng, P.E. Studies on mosquito population dynamics in different microenvironments in downtown Shanghai. *Chin J Vector Biol & Control* **2014,**25, 119–123. Chinese.
577 Gao, Y.M.; Li, X.H.; Wu, S.M.; Wei, M.L.; Liu, Y.M. Baseline survey results about pilot malaria eradication in Cheng’an County of Hebei Province. *Occup and Health* **2012,**28, 1740–1741. Chinese.
578 Gong, D.F.; Guo, X.F.; Lu, Y.L.; Ma, X.Z. Mosquitoes survey and Japanese encephalitis virus poisoned rate and antibodies investigation analysis of Xinping county. *J Med Pest Control* **2015,**31, 198–200+204. Chinese.
579 Gong, D.F.; Guo, X.F.; Zhou, H.N.; Wang, P.Y. Investigation of the prevalence of Japanese encephalitis in Jingdong County, Yunnan. *Journal of Pathogen Biology* **2010,**5, 57–58+68. Chinese.
580 Gong, H.Q.; Zhang, H.L.; Shi, H.F.; Zhang, Y.Z.; Gong, Z.D.; Mi, Z.Q. Investigation on the Mosquito and Arbovirus in the Meng Ding Town, Geng Ma County, Yunnan Province. *Chin J Vector Biol & Control* **2000,**3, 177–179. Chinese.

581 Gong, Z.Y.; Fu, G.M.; Yang, T.C.; Ren, Z.Y.; Hou, J.; He, F. Surveillance of important disease vectors in Zhejiang province. *Chin J Parasitol Parasit Dis* **2010,**25, 294–298. Chinese.

582 Gong, Z.D.; Lu, B.L. An new subgenus and new species, genus *Aedes* (Diptera: Culicidae). *Entomotaxonomia* **1991,**1, 55–59. Chinese.

583 Guan, B.Y.; Liang, B.N.; Xian, Y.H. Study on Malaria Surveillance and key control strategies in Jiangmen City from 2006 to 2015. *China Prac Med* **2016,**11, 195–196. Chinese.

584 Guo, C.K.; Li, J.H.; Tan, Y.X. Investigation on geographical distribution, ecological feature and malaria transmission of *Anopheles* in Guangxi. *Chin J Vector Biol & Control* **2007,**2, 112–115. Chinese.

585 Guo, L.L.; Chen, S.; Wang, K. Mosquitoes and flies surveillance in Danyang from 2009 to 2015. *Chin J Hyg Insect & Equip* **2019,**25, 156–159. Chinese.

586 Guo, T.Q.; Yang, J.; Dong, C.L.; Jiang, W. The analysis of surveillance status on malaria transmission medium in Mangshi from 2011 to 2014. *J Med Pest Control* **2016,**32, 971–973. Chinese.

587 Guo, Y.H.; Zhang, J.; Liu, X.B.; Meng, F.X.; Lu, L.; Wang, J.; Ren, D.S.; Chang, Z.R.; Mao, D.Q.; Liu, Q.Y., et al. Mosquito surveillance in fluctuating zones of the Three Gorges Reservoir region, China, in 2010. *Chin J Vector Biol & Control* **2014,**25, 421–423. Chinese.

588 Han, K.; Wu, Z.L.; Lan, C.J.; Zhang, S.B.; Gao, J.W. Cytochrome oxidase subunit I(COI) gene sequences analysis for different species of mosquitoes collected from Ningxia. *Chin J Hyg Insect & Equip* **2017,**23, 373–375. Chinese.

589 Hao, H.B.; Xia, S.G.; Yao, M.L.; Wu, K.L.; Huang, J.G. Analysis on the results of monitoring dengue vector from 2010 to 2014 in Jingzhou City. *J Med Pest Control* **2016,**32, 1008–1010. Chinese.

590 He, X.F.; Xue, J.L.; Ke, Z.G.; Zhang, S.R.; Ding, J.L.; Chen, J.H.; Chen, M.D.; Ding, S.Q. Vertical distribution of *Aedes albopictus* in bamboo forest area in Fengyang Mountain, Zhejiang Province. *J Med Pest Control* **1987,**1, 10–13. Chinese.

591 He, Y.M.; Tu, T.T.; Shen, T.Z.H.; Xiao, H.S.; Feng, S.Q.; Yang, X.F.; Ye, S.; Yu, Z.; Chen, S.; Wang, Y.Q, et al. An investigation of the distribution of mosquito populations in Chongqing, China, 2018. *Chin J Vector Biol & Control* **2019,**30, 570–573+577. Chinese.

592 He, Z.Z.; Wang, S.Y.; Li, L.; Luo, S.W. Surveillance and Analysis of Vector organisms of Malaria and Dengue Fever Infectious Diseases in Chengdu Shuangliu International Airport from June 2005 to May 2006. *J Med Pest Control* **2006,**9, 645–648. Chinese.

593 He, X.X.; Wang, H.Y.; Fu, S.H.; He, Y.; Yang, F.Z.; Chen, W.X.; Xu, B.H.; Lu, S.N.; Xie, H.G.; Ha, S.S.R, et al. Genotype I Japanese encephalitis virus is in the main genotype in mosquito in Fujian province. *Chinese J Exp Clin Virol* **2012,**2, 81–83. Chinese.

594 Huang, J.B.; Lan, Y.Q.; Pan, X.M.; Li, Y.M.; Yu, Y.; Li, Y.; Chen, R.F. Analysis of mosquito surveillance results in Liandu district. *Prev Med* **2019,**31, 841–843+847. Chinese.

595 Huang, J.Y.; Xu, B.H.; Li, J.H.; Jiang, T.B.; Zhang, L.Y.; Tu, Z.P.; Zhang, S.Y. Analysis on the surveillance of *Anopheles* in the late-stage of malaria eradication in Jiangle County. *Journal of Pathogen Biology* **2007,**2, 170+172. Chinese.

596 Huang, K.; Xu, J.Y.; Lin, H.; Hong, C.Z. The report of Dengue transmission intermedia at Zhaoyin port district in Zhangzhou port. *Science of Travel Medicine* **2009,**15, 38–39. Chinese.

597 Huang, Y.D.; Ma, Z.H. Investigation and Evaluation on the density of mosquitoes, flies and mice in Qizishan

Landfill Farm of Suzhou City. *Shanghai Journal of Preventive Medicine* **1998,**6, 269–270. Chinese.
598 Huang, Z.M.; Fan, Z.H.; Fan, B.; Ma, G.Y.; Xu, C.L.; Wu, B.W. Results of monitoring of malaria transmission vectors after elimination of malaria in three counties in Yuxi City . *China Tropical Medicine* **2007,**12, 2301–2302. Chinese.
599 Ji, H.Q.; Feng, S.Q.; He, Y.M.; Li, X.A.; Liu, N.; Zhu, B.; Yin, T.; Du, J. Studies on Species and Geographical Distribution of Mosquitoes in Chongqing Area, China. *J Med Pest Control* **2009,**25, 568–571. Chinese.
600 Jia, H.L.; Li, M.H.; Fu, S.H.; Wang, J.; Zhang, Y.G.; Hu, W.F.; Liang, G.D. Isolation and identification of genotype I Japanese encephalitis virus from mosquitoes in Anhui province, China in 2010. *Chin J Vector Biol & Control* **2012,**23, 395–397. Chinese.
601 Jia, L.; Wang, Z.B.; Lu, T.L.; Mi, Y.Q. Etiological investigation of Japanese encephalitis in South Shanxi Province. *Shanxi Med J* **1959,**3, 3–9. Chinese.
602 Jiang, H.R.; Wang, W.; Zhang, T.; Ma, D.Q.; Zhao, Y.J.; Jiang, C.L.; Liu, J. An investigation of important vectors at Dongjiakou port. *Chin J Hyg Insect & Equip* **2015,**21, 63–65. Chinese.
603 Jiang, H.J.; Chen, Y.Y.; Zheng, C.H.; Su, C.J.; Tang, Z.Z. Surveillance and analysis of vector organisms of dengue fever at Xiaocuo Port. *Port Health Control* **2010,**15, 45–48. Chinese.
604 Jing, X.; Liu, G.F.; Wang, M.; Lin, X.J.; Shi, Z.K.; Wang, G.Y.; Si, H.X. Monitoring of mosquito vectors of Japanese encephalitis in different habitats of Zibo, Shandong. *Chin J Vector Biol & Control* **2011,**22, 584–586. Chinese.
605 Jing, X.; Wang, H.Y.; Liu, Y.; Ma, J.C.; Wang, S.D.; Wang, H.Y. The survey of mosquito vectors of epidemic encephalitis B in Dongming county, Shandong. *Chin J Vector Biol & Control* **2007,**2, 109–111. Chinese.
606 Ke, C.R.; Duan, B.H.; Lin, C.S.; Li, L.; Liu, Y.; Chen, X.X.; Li, Z.X.; Li, C.G. Dengue Fever Media in 12 Counties (Cities) in Dali Prefecture. *Parasitoses and Infectious Diseases* **2017,**15, 181–185. Chinese.
607 Lai, J.H.; Zheng, Z.W.; Shen, P.L. Monitoring results of vectors in Longgang central urban area of Shenzhen City from 2012-2013. *Occup and Health* **2014,**30, 3260–3262. Chinese.
608 Lei, W.W.; Guo, X.F.; Fu, S.H.; Feng, Y.; Song, J.D.; Zhou, H.N.; Liang, G.D. Isolation and identification of Nam Dinh virus from mosquito specimens from the border between China and Myanmar. *Chinese Journal of Virology* **2016,**32, 782–789. Chinese.
609 Li, Z.Q.; Qu, Z.Q.; Shang, H.Y.; Ling, F.; Li, P.; Luo, M.F.; Shi, J. Field control efficacy of *Aedes* vectors of dengue fever with five public health insecticides. *Chin J Vector Biol & Control* **2018,**29, 54–56. Chinese.
610 Li, C.M.; Tang, J.X.; Cai, Y.S.; Xu, B.; Han, G.J.; Liu, Q.; Zhao, S.; Xu, J. Population structure and seasonal dynamics of mosquitoes in different types of residential areas. *Chin J Schisto Control* **2017,**29, 720–724+787. Chinese.
611 Li, H.; Jiang, J.Y.; Dong, L.M.; Chen, Z.L.; Chen, S.M.; Li, J.M.; Liu, H.X. Emergency monitoring and analysis of the epidemiological characteristics and vectors of a dengue fever outbreak in Mengla County in 2018. *Journal of Pathogen Biology* **2020,**15, 83–85+90. Chinese.
612 Li, H.C.; Pan, H.; Feng, Y.; Deng, W.; Yang, G.R.; Yang, P.; Shi, J.; Zhang, H.L. Epidemiological survey of an outbreak of dengue fever in Lincang, Yunnan, 2015. *Chin J Parasitol Parasit Dis* **2016,**31, 561–565. Chinese.
613 Li, H.C.; Yang, G.R.; Shi, A.J.; Shi, J.; Yang, P.; Li, Y.; Wang, X.Z. Investigation of distribution of dengue vectors in Lincang border area. *China Tropical Medicine* **2015,**15, 186–188. Chinese.

614 Li, J.Q.; Wang, W.J.; An, N.O.; Ye, X.F.; Liu, C.T.; Zhao, S.Y.; You, Y.G. Analysis on monitoring of Japanese Encephalitis, Dejiang county, 2005-2011. *Prev Med Trib* **2013,**19, 289–291. Chinese.

615 Li, J.F.; Ha, Z.; Li, C.H.; Zhao, G.Y.; Li, Z.X.; Zhang, H.; Guo, Y.C.; Lu, H.J.; Zhang, Y.; Jin, N.Y., et al. Detection of PCV2 and genetic and evolutionary analysis of ORF2 gene in mosquitoes in Simao area of Yunnan province in 2018. *Journal of Pathogen Biology* **2019,**14, 746–749+754. Chinese.

616 Li, J.H.; Du, J.F.; Tan, Y.X.; Gong, W.R.; Fang, T.X.; Fu, J.Y.; Li, Z.; Jiang, S.S.; Deng, H.X.; Feng, Y.X., et al. Results of malaria surveillance in monitoring sites of Guangxi for five years. *China Tropical Medicine* **2010,**10, 1046–1047. Chinese.

617 Li, M.Y.; Tang, J.F. Monitoring and Analysis of media for Chikungunya Heat. *China Medical Engineering* **2012,**20, 141+143. Chinese.

618 Li, M.H.; Fu, S.H.; Jiang, H.Y.; Chen, W.X.; Tang, S.; Shi, Y.; Liang, G.D. Isolation and identification of Japanese encephalitis virus in Jiangxi province, China. *Chin J Vector Biol & Control* **2012,**23, 388–390+394. Chinese.

619 Li, W.; Pan, M.; Zhou, X.Y.; Lin, S.H.; Liu, X.C.; Fu, S.H.; Chen, D.L.; Cao, Y.O.; Liang, G.D.; Zhang, J.K., et al. First isolation and identification of Getah virus SC1210 in Sichuan. *Chinese J Exp Clin Virol* **2017,**31, 2–7. Chinese.

620 Li, X.H.; Peng, S.Q.; Wu, X.B. Analysis report on biological data of medical vector in Yantian Port in 2007 and 2012. *Port Health Control* **2014,**19, 46–51. Chinese.

621 Li, X.Y.; Huang, X.Y.; Tang, X.Y.; Xu, B.L. Isolation and identification of arboviruses from mosquito pools of Xinyang city of Henan province in 2009. *Chin J Viral Dis* **2012,**2, 207–210. Chinese.

622 Li, Y.; Han, H.; Jia, R.Z.; Shi, H.; Huang, Q.Z.; Shi, H.Q. Study on insecticide resistance detection and control strategy of mosquitoes and flies in the troop sanatorium in Beidaihe. *Chin J Hyg Insect & Equip* **2017,**23, 410–413+418. Chinese.

623 Li, Y.Y.; Sang, H.; Zhao, W.; Liu, Y.F.; Wang, P.S. Investigation on the Distribution of mosquitoes in port in Mengla county, Yunnan Province, the border between China and Myanmar. *J Med Pest Control* **2010,**26, 729–730. Chinese.

624 Li, Y.D.; Li, Y.T.; Cheng, W.L.; Ma, T.Z.; Ma, Y.X.; Tian, B. Investigation and Analysis of Vector in Water Olympic Stadium and surrounding area of Shunyi District in 2006. *Chin J Vector Biol & Control* **2008,**4, 358–360. Chinese.

625 Li, Y.Y.; Zhu, J.; Li, H.B. Distribution of the dengue fever vector in Xishuangbanna Prefecture of Yunnan. *China Tropical Medicine* **2016,**16, 237–239+265. Chinese.

626 Li, Z.X.; Zhang, H.L.; Gong, Z.D.; Zi, D.Y.; Mi, Z.Q.; Shi, H.F. The characteristic of mosquitoes distribution and relation with the arbovirus in jingHong City Yunnan Province. *Chin J Vector Biol & Control* **1998,**4, 23–25. Chinese.

627 Liang, K.F.; Liu, Q.; Wang, D.Q.; Zhou, J.M.; Jin, Y.J.; Chen, Y.J.; Li, J.M.; Gan, L.P.; Yang, H. Establishment and preliminary Application of Real-time fluorescence PCR for Detection of Nam Dinh virus. *Chin J Vector Biol & Control* **2015,**26, 447–450. Chinese.

628 Liang, Y.H.; Jiao, D.J.; Yao, Z.Q.; Tu, X.; Wang, Q.Z.; Kang, Z. Background investigation report of vector organisms at Qiqihar Air Port. *Chinese Frontier Health Quarantine* **2008,**2, 117–119. Chinese.

629 Lin, C.; Feng, L.; Gu, Y.P.; Liu, J.; Liu, H.Z. Study on mosquito density and population composition in

Pudong New area of Shanghai from 2017 to 2019. *Shanghai Journal of Preventive Medicine* **2020,**32, 974–979. Chinese.

630 Lin, K.M.; Du, J.F.; Li, J.H.; Huang, Y.M.; Fu, J.Y.; Fang, T.X.; Jiang, S.S.; Huang, B.C.; Wang, S.; Deng, J.F, et al. Analysis on epidemic situation of malaria surveillance sites in Guangxi in 2010. *Chin J Vector Biol & Control* **2013,**24, 317–319. Chinese.

631 Lin, K.S.; Xu, B.F. Investigation on population composition of rodent and vector insects in Shuidong Port, Maoming city. *J Med Pest Control* **1996,**3, 39–40. Chinese.

632 Lin, S.F.; Lin, F.X.; Liu, Y.P. Surveillance of dengue fever vector in Taijiang District of Fuzhou City in 2005. *Strait J Prev Med* **2007,**1, 58–59. Chinese.

633 Liu, B.Y.; Xiong, G.H.; Li, Y.C.; Gao, S.W.; Liu, X.T.; Li, H.P.; Lin, X.Z. Investigation and comparative analysis on the distribution of vector organisms in port area and residential area of Mawei Port. *Journal of Fujian Medical University* **2006,**6, 642–647. Chinese.

634 Liu, C.C.; Luo, Y.; Xu, H.B. Investigation on mosquito vectors in urban area in Simao City, Pu'er City, Yunnan Province. *J Diseases Monitor & Control* **2017,**11, 431–434. Chinese.

635 Liu, F.; Zhang, P.; Liu, Y.; Wu, C.Y.; Liu, J.Y. Analysis of surveillance results of malaria vector from 2005 to 2016 in Yuqiao District. *Anhui J Prev Med* **2017,**23, 241–244. Chinese.

636 Liu, G.P.; Xing, A.H.; Ren, Q.M.; Wang, F. Investigation on species Distribution of important Blood-sucking Diptera insects in Heilongjiang Basin. *Chin J Hyg Insect & Equip* **2009,**15, 471–473. Chinese.

637 Liu, H.B.; Yin, J.S.; Zhang, C.M. A preliminary investigation on the distribution of *Anopheles anthropophagus* in Huizhou City. *South China J Prev Med* **1992,**3, 65–66. Chinese.

638 Liu, H.L.; Zhou, Q.F.; Wang, Y.X.; Fan, W.D.; Li, G.S. The establishment and initial application of real-time fluorescent PCR detection of Nam Dinh virus. *J Prev Med Inf* **2008,**8, 583–587. Chinese.

639 Liu, H.; Qu, J.Q.; Wang, X.J.; Jing, X. Evaluation of the application effect of two monitoring methods in dengue media monitoring. *Chin J Hyg Insect & Equip* **2018,**24, 243–246. Chinese.

640 Liu, H.X.; Liu, J.Y.; Lu, X.Y.; Xiao, Y.J.; Li, H.B.; Wu, C.; Ceng, X.C.; Yang, H.L.; Zhou, H.N. Analysis and investigation of outbreaks of dengue fever in Mengla county, Xishuangban prefecture in Yunnan province. *Journal of Pathogen Biology* **2014,**9, 268–270. Chinese.

641 Liu, J.; Che, Z.J.; Liu, Y.Y.; Ma, W.D.; Guo, T.Y.; Li, C.C.; Cao, J.Z.; Wang, D.H.; Tian, J.; Geng, H.S, et al. Analysis on mosquito surveillance in Beijing ports. *Chin J Vector Biol & Control* **2009,**20, 479–480. Chinese.

642 Liu, J.; Jiang, Y.M.; Zhang, J.Y.; Zhao, Z.Y.; Li, X.N.; Liang, X.Y.; Wu, H.Y.; Liu, Y.; Yang, Z.C.; Luo, L., et al. Investigation on species and distribution of mosquitoes in Guangzhou. *Chin J Hyg Insect & Equip* **2019,**25, 244–246. Chinese.

643 Liu, J.; Meng, F.X.; Qian, W.P.; Xu, Y.; Yu, X.L. Investigation of mosquitoes in Neijiang from 2011 to 2013. *Chin J Hyg Insect & Equip* **2015,**21, 175–177. Chinese.

644 Liu, M.G.; Tu, S.; Shu, H.C.; Li, Q.; Min, Z.B. Malaria epidemic characteristics in Fengxin County of Jiangxi: 1950-2015. *Modern Preventive Medicine* **2016,**43, 3539–3542. Chinese.

645 Liu, P.; Zhou, Y.J.; Wang, Z.A.; Yuan, L.Y.; Ma, X.M.; Zhang, Y.Y. The investigation of the seasonal fluctuation of vector in Ankang city. *Chin J Vector Biol & Control* **2010,**21, 596–598. Chinese.

646 Liu, Q.Q.; Wei, J.C.; Zhong, D.K.; Wu, Y.L.; Shi, K.; Zhang, K.L.; Lu, M.L.; Xiao, C.G.; Li, B.B.; Liu, K, et al. Isolation,identification and molecular characteristic of a Japanese encephalitis virus isolate from

Anopheles. *Chinese Veterinary Science* **2017,**47, 16–22. Chinese.
647 Liu, R.; Zhang, G.L.; Sun, X.; Zheng, C.; Liu, X.M.; Zhao, Y.; Liu, S.K.; Dang, R.L.; Zhao, T.Y. Isolation and molecular characterization on Abbey Lake Orthobunyavirus (Bunyaviridae) in Xinjiang, China. *Chin J Epidemiol* **2014,**35, 939–942. Chinese.
648 Liu, X.H.; Zhou, R.J. Investigation on filariasis and mosquito vectors in Boxian County, Anhui Province. *Chin J Parasitol Parasit Dis* **1986,**2, 44. Chinese.
649 Liu, X.B.; Ci, R.D.Z.; Guo, Y.H.; Peng, C.C.R.; Bai, L.; Sang, S.W.; Bai, M.C.W.; Gu, S.H.; Da, Z.; Chen, B, et al. A survey of species composition and population dynamics of mosquitoes in Lhasa, Tibet, China from 2009 to 2013. *Chin J Vector Biol & Control* **2014,**25, 200–204. Chinese.
650 Liu, X.B.; Guo, Y.H.; Li, J.H.; Wang, J.; Zhou, H.N.; Meng, F.X.; Chen, R.; Ren, D.S.; Lai, M.Y.; Liu, Q.Y., et al. Surveillance of adult *Aedes* mosquitoes in response to the outbreak of dengue fever in Xishuangbanna using BG-Sentinel mosquito trap. *Chin J Vector Biol & Control* **2014,**25, 97–100. Chinese.
651 Liu, X.N.; Wu, N.J.; Wu, S.L.; Chen, J.Y.; Xie, M.L.; Wang, D.Q. Analysis of surveillance for dengue vector in Pingshan district of Shenzhen city, 2016. *Chin J Vector Biol & Control* **2017,**28, 283–285. Chinese.
652 Liu, Y.M.; Xia, S.W. Analysis on the monitoring of vectors in Putuo District of Shanghai from 2015 to 2017. *Chin J Hyg Insect & Equip* **2019,**25, 328–333. Chinese.
653 Liu, Y.; Hu, S.B. Analysis of Surveillance results of Dengue Fever in Anfu County from 2008 to 2011. *Chin J of PHM* **2012,**28, 777. Chinese.
654 Liu, Z.R.; Li, Z.; Chen, X.; Li, R.Y.; Zhang, F.N. Epidemiological Situation and Control Effect of Malaria in Meishan, 2006－2011. *J Prev Med Inf* **2013,**29, 229–233. Chinese.
655 Lu, Y.L.; Ceng, X.C.; Xie, H.; Tan, J.; Guo, X.F.; Yang, Z.H.; Ma, X.Z.; Yang, M.D.; Zhou, H.N. A survey of dengue fever vectors in Menglian and Lancang counties, Myanmar border. *Chin J Vector Biol & Control* **2012,**23, 29–31. Chinese.
656 Lu, Y.L.; Gao, F.; Tan, W.H.; Li, Y.P.; Zhang, J.S.; Liu, Z.H.; Luo, Z.X.; Zhu, Y.; Yang, X.B.; Zhou, H.N., et al. Investigation on the Distribution of *Aedes aegypti* at Qingshuihe Highway Port, Lincang, Yunnan Province. *Port Health Control* **2016,**21, 49–51. Chinese.
657 Lu, B.L.; Li, B.S.; Liu, S.Z. Notes of the mosquitoes new to China, 6. *Acta Zootaxonomica Sinica* **1981,**3, 275. Chinese.
658 Lu, B.L.; Li, B.S.; Xu, R.M.; Jiang, Y.Y. Composition of mosquitoes breeding in tree holes in Dangluoshan forest region of Hainan Province. *Mil Med Sci* **1980,**1, 55–59. Chinese.
659 Lu, B.F. Analysis on density Monitoring of the Four Pests in Jiangyin City in 2006. *Jiangsu Health Care* **2007,**6, 5–6. Chinese.
660 Lu, B.F. Analysis on density Monitoring of the Four Pests in Jiangyin City in 2009. *J Med Pest Control* **2011,**27, 47+49. Chinese.
661 Lu, M.; Sun, W.; Wu, H.M.; Pan, D.G.; Bi, Y.J.; Tan, J.; Li, H.W.; Wang, L.H.; Zhou, Y. An Investigation to the population of and the pathogen in the vector at the 2nd phase construction of the terminal building of Baiyun international airport. *Chinese Frontier Health Quarantine* **2015,**38, 394–401+393. Chinese.
662 Lu, X.Q.; Ma, D.Z.; Li, Q.B. Study on Haemophilic habits of Culicini in rooms in Puzhen area of Nanjing City. *Acta Entomologica Sinica* **1959,**2, 178–182. Chinese.
663 Luo, C.; Zheng, D.K.; Chen, C.R.; Wang, J. Analysis of the surveillance results of dengue and mosquito

vectors in Wanzhou district of Chongqing in 2015. *Chin J Vector Biol & Control* **2016,**27, 393–394. Chinese.

664 Luo, H.R.; Lei, L.B.; Wu, S.M.; Liao, Y.H.; Pan, Z.M.; Wang, L.P.; Liang, G.D. Isolation and Identification of four strains of Dengue Fever virus in Guangzhou from 1985 to 1990. *Chin J Parasitol Parasit Dis* **1993,**2, 36–38. Chinese.

665 Luo, Y.N.; Hu, Y. Results of vector monitoring in Cixi city, 2008. *Chin J Vector Biol & Control* **2010,**21, 492–494. Chinese.

666 Luo, Z.X.; Deng, R.; Wang, X. Monitoring on import cases of malaria and vector species at Menglian port. *Chinese Frontier Health Quarantine* **2011,**34, 233–234+237. Chinese.

667 Luo, S.Y.; Zhu, Z.H.; Lou, T. A comparative study on fixed and mobile monitoring for *Aedes albopictus* (Diptera: Culicidae). *Chin J Vector Biol & Control* **2013,**24, 538–540. Chinese.

668 Luo, X.D.; Wen, H.Y.; Wang, D.; Hao, Y.T.; Peng, Y.Q.; Zhou, L.; Zhang, H.R. Molecular characterization of the mosquitoes collected in ports along the Three Gorges Reservoir. *Chinese Frontier Health Quarantine* **2018,**41, 24–27+42. Chinese.

669 Ma, C.Z.; Wang, P. Investigation report on vector insects and rodent at Changbai Port. *Chinese Frontier Health Quarantine* **1992,**5, 301–304. Chinese.

670 Ma, L.; Lu, S.Y. Monitoring results analysis of mosquito density in different regions of Ningxia. *J Med Pest Control* **2015,**31, 1131–1132+1135. Chinese.

671 Ma, M.Z.; Wei, Z.Q.; Wang, X.H.; Gao, M.Q.; Shen, H.E.; Wang, Y. Morphological and molecular identification of mosquitoes collected at Caofeidian area, Hebei province. *Chinese Frontier Health Quarantine* **2019,**42, 403–406. Chinese.

672 Ma, M.; Ma, X.; Yang, S.J.; Wang, G.A.; Sun, B. Analysis of surveillance results on dengue vector of Ningbo city in 2017. *Chin J Vector Biol & Control* **2018,**29, 379–382. Chinese.

673 Ma, X.Q.; Wang, Y.; Gao, L.; Liu, F.; Chen, X.L.; Li, X.L.; Yang, L. Monitoring of medical vectors in Anning District of Lanzhou City in 2014. *Chin J Hyg Insect & Equip* **2016,**22, 166–168. Chinese.

674 Man, Y.Z.; Wang, D.M. Monitoring results of density of the vector organism in Miyun District of Beijing City in 2012. *Occup and Health* **2013,**29, 1375–1376. Chinese.

675 Man, Y.Z.; Wang, D.M. Analysis on surveillance results for vector density in Miyun county in 2013, Beijing. *Capital Journal of Public Health* **2015,**9, 59–62. Chinese.

676 Mao, X.H.; Li, C.M.; Yang, M.D. Monitoring results of mosquito vector of Japanese encephalitis in Yunnan in 2017. *China Tropical Medicine* **2018,**18, 1134–1137. Chinese.

677 Mei, Y.; He, X.M.; Xiao, J.W.; Ding, J.F. Investigation of medical vectors in Rugao port. *Chin J Hyg Insect & Equip* **2015,**21, 384–387. Chinese.

678 Meng, F.X.; Sun, Y.X.; Wu, Z.; Wu, H.X.; Zhao, C.C.; Jia, Y.X.; Li, Z.P.; Mao, W.X.; Lun, X.C.; Zhang, P.J, et al. Integrated mosquito prevention and control package and efficacy evaluation in the endemic area of Japanese encephalitis in Jingning county of Gansu province, China, 2019. *Chin J Vector Biol & Control* **2021,**32, 144–149. Chinese.

679 Meng, Q.H. A new record of *Culex*. *Acta Entomologica Sinica* **1958,**4, 351–354. Chinese.

680 Meng, Q.H. Investigation on mosquitoes in Mangshi City, Yunnan Province II. Preliminary investigation on larval density of *Anopheles*. *Journal of Guizhou Medical University* **1960,**2, 36–37. Chinese.

681 Meng, W.S.; Li, M.H.; Wang, H.Y.; Zhang, J.B.; Gao, Y.; Ding, J.; Fu, S.H.; Zhai, Y.G.; Chen, Z.; Guo, J.Q,

et al. Genotype 1 JEV was isolated again from Liaoning province China 2007. *Chinese J Exp Clin Virol* **2008,**2, 91–94. Chinese.

682 Meng, X.J.; Guo, Y.Q.; Zhao, J. Analysis of mosquito surveillance results in rural areas of Yuhang District. *Prev Med* **2016,**28, 832–834. Chinese.

683 Mi, Z.Q.; Zi, D.Y.; Zhang, H.L.; Gong, Z.D.; Yang, L.P.; Shi, H.F. Investigation of mosquitoes and isolation of Japanese Encephalitis Virus in Luxi city, Yunnan province. *J Med Pest Control* **2002,**7, 367–369. Chinese.

684 Ming, M.; Zhao, A.H.; Zhao, Y.; Wang, C.Y.; Ma, D.Z. Analysis on density and seasonal fluctuation of the important medical vectors in Tai'an City in 2014. *Chin J Hyg Insect & Equip* **2016,**22, 582–585. Chinese.

685 Mo, Z.J.; Lv, X.J.; Tan, Y.; Xie, C.J.; Bi, F.Y.; Li, X.Y.; Pan, X.L.; Sun, X.H.; Wang, H.Y.; Fu, S.H, et al. Isolation of genotype I Japanese encephalitis virus in Beiliu, Guangxi. *Chin J Parasitol Parasit Dis* **2010,**25, 115–119. Chinese.

686 Nan, X.W.; Jie, X.X.; Yu, H.M.; Si, X.Y.; Chen, J.L.; Zhang, C.G.; Zhang, X.H. An investigation of the vector mosquitoes and arboviruses during a Japanese encephalitis epidemic in Baotou of Inner Mongolia autonomous region, China, 2018. *Chin J Vector Biol & Control* **2020,**31, 652–656. Chinese.

687 Pan, H.; Fu, D.M.; Feng, Y.; Yang, W.H.; Li, J.C. Investigation of mosquito-borne viruses in Xiangyun county, Yunnan province. *International Journal of Virology* **2020,**27, 484–487. Chinese.

688 Pan, H.; Gao, Y.; Feng, Y.; Han, Q.; Zhang, J.; Zhu, J.; Li, W.P.; Li, H.B.; Fan, J.H.; Zhang, H.L., et al. Identification of Japanese encephalitis viral infections in mosquitoes in Jinghong city, Yunnan province, China. *Chin J Vector Biol & Control* **2018,**29, 331–335. Chinese.

689 Pan, Y.J.; Pan, J.F.; Xie, L.L.; Gao, J.M. Vectors investigation in the Qingpu district, Shanghai city. *Chin J Vector Biol & Control* **2008,**19, 537–539. Chinese.

690 Pan, Z.M.; Liu, S.G.; Qiu, J.C.; Luo, H.R.; Huang, Y.H.; Liao, Y.H.; Lei, L.B.; Hu, Z.G. Analysis of epidemic characteristics of dengue fever in Guangzhou in 1990. *Chin J Parasitol Parasit Dis* **1993,**4, 92–95. Chinese.

691 Qi, Y. Investigation report on *Aedes albopictus* in Yulin, Luchuan and Bobai counties. *Guangxi Medical Journal* **1982,**3, 145. Chinese.

692 Qian, K.; Han, Y.H.; Xue, S.Q.; Tian, Y.L.; Zhang, Y.; Liu, T.; Fu, X.F.; Ceng, X.P. Analysis of main vectors density from 2006 to 2008 in Beijing Olympic venues. *Chin J Vector Biol & Control* **2009,**20, 31–33. Chinese.

693 Qu, R.B.; Yue, A.P.; Liu, R.C.; Guo, Z.K.; Yue, L.X.; Ren, H. A feasibility exploration of malaria elimination in Rongcheng. *Journal of Pathogen Biology* **2013,**8, 1029–1030+1055. Chinese.

694 Qu, F.Y.; Chen, H.B. Two new rocords of *Uranotaenia* mosquitoes breeding in crab holes from Yunnan province. *Entomotaxonomia* **1983,**4, 343–344. Chinese.

695 Quan, F.; Zhu, S.J.; Zhang, S.J.; Ma, T.Z.; Tian, B.; Tang, C. Investigation of mosquito density in Wenyu Ｒ iver Valley of Shunyi in Beijing. *Chin J Hyg Insect & Equip* **2016,**22, 378–380. Chinese.

696 She, J.J.; Jia, W.H.; Zhou, J.X.; Lv, W. Investigation on population and distribution of mosquitoes and flies in Huanglong County Seat of Shaanxi Province. *Chin J Hyg Insect & Equip* **2018,**24, 475–477. Chinese.

697 Shi, S.Z.; Liu, Z.J. A preliminary record of mosquitoes in Gansu Province. *J Med Pest Control* **1994,**4, 256–258. Chinese.

698 Shu, J.; Shang, Y.H. Analysis on monitoring results of dengue vector from 2015 to 2017 in Wuyi county. *Chin J of PHM* **2019,**35, 286–288. Chinese.

699 Song, F.L.; Wu, G.; Xue, F.; Li, J.X.; Liu, H.W.; Fan, D.H.; Li, C.S.; Jiang, Y.L.; Gao, Y.F.; Qin, F, et al. Study on Pathogens Carried by Vectors at Frontier Ports. *Chinese Frontier Health Quarantine* **2009,**32, 368–374. Chinese.

700 Song, H.; Kong, X.L.; Feng, Y.; Wang, L. Density surveillance of main vectors in urban area of Qufu city. *Chin J Hyg Insect & Equip* **2014,**20, 257–260. Chinese.

701 Sun, G.L. Analysis on mosquito monitoring results, Shouguang city, 2016-2018. *Prev Med Trib* **2019,**25, 121–123. Chinese.

702 Sun, X.H.; Fu, S.H.; Zhang, H.L.; Wang, H.Y.; He, Y.; Liu, W.B.; Yang, W.H.; Feng, Y.; Min, J.G.; Han, R.H, et al. Isolation and identification of arboviruses from mosquito pools in Yunnan province. *Chinese J Exp Clin Virol* **2005,**4, 319–324. Chinese.

703 Sun, Z. Summary of Studies on *Aedes albopictus* in Henan Province. *Henan J Prev Med* **1982,**3, 116–122. Chinese.

704 Tan, Y.; N, I.P.A.B.E.N.J.A.P.H.O.N.G.; U, -.R.U.Y.A.K.O.R.N.C.H.A.N.S.A.N.G.; Zhong, G.M.; Liao, G.H. Sampling investigation on mosquito species and distribution in some areas of Guangxi. *Acta Parasitol Med Entomol Sin* **1999,**4, 34–37. Chinese.

705 Tan, Y.; Bi, F.Y.; Xie, Y.H.; Yang, J.Y. Investigation of infectious status of Japanese encephalitis B virus in mosquitoes in high JE prevalence areas of Guangxi Province. *China Tropical Medicine* **2011,**11, 688–689. Chinese.

706 Tan, Y.; Xia, F.M.; Ning, C.Z.; Tan, L. Investigation report on vector insects and rodent in Qinzhou Port. *J Med Pest Control* **1997,**4, 206–207. Chinese.

707 Tang, A.Q.; Hu, Y.Q. Feasibility analysis of achieving the national goal of eliminating malaria in Yuhang District. *Zhejiang Prev Med* **2014,**26, 211–212. Chinese.

708 Tang, C.J.; Ge, J.Q.; Zhang, H.J.; Fu, S.H.; Li, Y.Y.; Xu, C.; Wang, H.P.; Zhang, Z. First report of Liaoning virus from mosquitoes in Chaoyang district, Beijing. *Chin J Vector Biol & Control* **2017,**28, 113–116. Chinese.

709 Tang, G.P.; Zhou, J.Z.; Jiang, W.J.; Ren, L.J.; Yu, C.; Zhuang, Y.; Tian, X.G.; Wang, D.M. Surveillance of dengue fever vector and investigation of population infection in some counties of Guizhou Province. *J Med Pest Control* **2015,**31, 641–644. Chinese.

710 Tang, Y.; Leng, P.E.; Yuan, J.L.; Xu, K.; Zhou, Y.C.; Huang, Z.Y. Analysis of seasonal fluctuation and infestation of vectors within pre-construction period in the Shanghai World Expo area of Luwan district, Shanghai. *Chin J Vector Biol & Control* **2010,**21, 5–8+11. Chinese.

711 Tang, Z.J.; Zhao, S.C.; Zhang, J.; Li, W.J. The LNV were found in mosquitoes among Delingha area of Qinghai for the first time. *J Med Pest Control* **2017,**33, 771–772. Chinese.

712 Tian, B.; Zhang, S.J.; Ma, T.Z.; Quan, F.; Tang, C. Investigation of density of mosquitoes around Beijing Capital International Airport and its seasonal fluctuation in 2011. *Chin J Vector Biol & Control* **2012,**23, 562–563. Chinese.

713 Tian, S.H.; Huang, R.H.; Yang, X. Analysis of Monitoring results of Medical Vector Biology in Gaobeidian City. *Journal of Clinical Medical* **2014,**1, 1253+1256. Chinese.

714 Tian, Y.; Wu, Z.M.; Zhang, H.D.; Wu, X.Q.; Zhou, M.H.; Chu, H.L. Efficiency of three monitoring methods for the density of adult *Aedes albopictus*: a comparative analysis. *Chin J Vector Biol & Control* **2018,**29, 42–

45. Chinese.

715 Wang, B.; Zhang, B. The monitoring and analysis of seasonal fluctuation of the Main four vectors in Luxian county during 2007. *J Med Pest Control* **2008,**24, 898–900. Chinese.

716 Wang, F.; Jiang, L.; Huang, H.C.; Liu, H.X.; Chen, D.Y. Resistance of main vectors to commonly used insecticides in Hongkou district, Shanghai, China, 2015-2019. *Chin J Vector Biol & Control* **2020,**31, 143–147. Chinese.

717 Wang, F.F.; Zheng, R.G.; Huang, J.X. Surveillance report on the density of adult mosquitoes in Gao'an City. *J of Pub Health and Prev Med* **1998,**3, 38. Chinese.

718 Wang, F.F.; Zheng, R.G.; Liu, G.Q. An investigation on the four pests' ecology circumstances and seasonal growth and decline in Gao'an city. *Modern Preventive Medicine* **2002,**2, 158–159. Chinese.

719 Wang, H.Y.; Gao, X.Y.; Song, L.Z.; Li, Y.; Liu, G.F.; Wang, H.Y.; Fu, S.H.; Tao, Z.X.; Lin, X.J.; Liu, Y, et al. Isolation and Identification of Japanese Encephalitis Virus Firstly in Shandong Province. *Chinese Journal of Vaccines and Immunization* **2009,**15, 337–340. Chinese.

720 Wang, H.Y.; Fu, S.H.; He, Y.; Min, J.G.; Pan, X.L.; Liang, G.D. Co-prevalence of two genotypes of Japanese encephalitis virus in Shanghai, China. *Chin J Vector Biol & Control* **2012,**23, 398–401. Chinese.

721 Wang, H.Y.; Fu, S.H.; Wang, J.W.; He, Y.; Cai, Z.L.; Han, R.H.; Liu, G.P.; Sun, X.H.; Zhang, Q.; Tang, Q, et al. Arbovirus survey in some regions in Heilongjiang province. *Chinese J Exp Clin Virol* **2005,**4, 307–311+417. Chinese.

722 Wang, H.Y.; Hao, Z.Y.; Fu, S.H.; Zhang, A.M.; Cao, Y.X.; Song, F.D.; Li, L.H.; He, Y.; Wang, H.Q.; Tang, Q, et al. Isolation and identification of Japanese encephalitis virus in Tanghe county, Henan province. *Chinese J Exp Clin Virol* **2008,**2, 83–86. Chinese.

723 Wang, J.Z.; Yin, S.Q.; Li, X.S.; Shang, Z.Y.; Yan, H. Malaria focus investigation and disposal in Tengchong City between 2015 and 2016. *Chin J Schisto Control* **2017,**29, 626–628. Chinese.

724 Wang, J.L.; Zhang, H.L.; Sun, X.H.; Fu, S.H.; Mi, Z.Q.; Gong, Z.D.; Zhai, Y.G.; Tang, Q.; Liang, G.D. Identification and sequence analysis of NS1 and NS2a of two strains of dengue type-4 virus from mosquitoes in Yunnan province. *Chinese Journal of Zoonoses* **2008,**7, 636–640. Chinese.

725 Wang, L.; Sun, Y.Q.; Guo, Q.H.; Fan, L.; Fan, Y.Y.; Zhang, S.G. Surveillance of mosquitoes in Jiangbei New District of Nanjing in 2018. *Chin J Hyg Insect & Equip* **2019,**25, 573–575. Chinese.

726 Wang, S.H.; Wu, Z.R.; Zhang, S.Z.; Xu, Y.X.; Zhong, P.S.; Leng, P.E.; Liu, H.X. Investigation of mosquito infestations in kindergartens and surrounding residential areas in Jiading District of Shanghai. *Shanghai Journal of Preventive Medicine* **2016,**28, 532–537. Chinese.

727 Wang, S.H.; Zhou, H.; Xu, Y.X. Analysis of Vector Biological Surveillance in Jiading District of Shanghai from 2009 to 2011. *Applied Prev Med* **2012,**18, 162–165. Chinese.

728 Wang, W.; Gao, R.Q.; Ma, X.F.; Zhou, H.Z.; Jiang, H.R. Survey on the mosquito population density in Qingdao City in 2016. *Chin J Hyg Insect & Equip* **2017,**23, 351–353+358. Chinese.

729 Wang, W.; Wu, T.Y.; Qin, N.; Li, P.Y. Surveillance for mosquitoes in hospitals of Tianjin between 2008 and 2016. *Modern Preventive Medicine* **2018,**45, 1491–1494+1515. Chinese.

730 Wang, W.M.; Cao, J.; Zhou, H.Y.; Li, J.L.; Zhu, G.D.; Gu, Y.P.; Liu, Y.B.; Cao, Y.Y. Seasonal increase and decrease of malaria vector in monitoring sites of Jiangsu Province in 2005-2010. *China Tropical Medicine* **2013,**13, 152–155. Chinese.

731 Wang, W.M.; Zhou, H.Y.; Cao, J.; Li, J.L.; Zhu, G.D.; Gu, Y.P.; Liu, Y.B. Survey on malaria vectors in Jiangsu Province, 2005-2009. *Chin J Schisto Control* **2011,**23, 453–456. Chinese.

732 Wang, W.M.; Zhou, H.Y.; Zhu, G.D.; Tang, J.X.; Cao, Y.Y.; Li, J.L.; Cao, J. Establishment of malaria early warning system in Jiangsu Province VIII Malaria vector monitoring in Jiangsu Province. *Chin J Schisto Control* **2017,**29, 28–32+37. Chinese.

733 Wang, X.D.; Liu, M.D.; Song, F.L.; Yu, H.; Dong, Y.D.; Zhao, T.Y. Study on the relationship of geographical landscape and mosquito distribution in rice growing districts by remote sensing and geography information system. *Chin J Vector Biol & Control* **2008,**5, 396–399. Chinese.

734 Wang, X.L.; Chen, S.J.; Wu, X.J.; Li, S.M. Analysis on biological vector control result in Jurong cityfrom 2008 to 2010. *Chin J Hyg Insect & Equip* **2012,**18, 123–124+127. Chinese.

735 Wang, X.H.; Wei, Z.Q.; Ma, M.Z.; Gao, M.Q.; Lei, T.; Wang, Y.; Shen, H.E. Investigation and Analysis of Mosquito Population in Caofeidian Area of Tangshan City. *Port Health Control* **2019,**24, 56–58+62. Chinese.

736 Wang, Y.; Fu, Q.; Jin, Z.Y.; Liu, Y.J.; Liu, X.H.; Gong, R.; Wang, Q.X. Diversity and Control of Vector mosquitoes at Xianyang International Airport Port. *Port Health Control* **2016,**21, 45–47. Chinese.

737 Wang, Z.S.; Lan, C.J.; Shen, Y. The surveillance results of the mosquitoes in Jiangyin city from 2008 to 2010. *Chin J Hyg Insect & Equip* **2011,**17, 439–440. Chinese.

738 Wang, Z.L.; Ma, W.D.; Li, N.; Huang, J.H.; Zhang, J.J.; Wang, K.L.; Zhao, H.Q.; Wang, D.H. In vestigation and Discussion on the Medical Vectors at Beijing Chaoyang Port. *Chinese Frontier Health Quarantine* **2010,**33, 405–408. Chinese.

739 Wei, Z.B.; Zhu, S.R. Investigation on the population and seasonal growth and decline of the Four Pests in Bishan County. *Chin J Vector Biol & Control* **2005,**5, 56–58. Chinese.

740 Wu, L.Z.; Zhang, Y.J.; Li, X.Q. Analysis of Surveillance results of Japanese Encephalitis virus carried by mosquitoes in Xianju County, Zhejiang Province. *Modern Practical Medicine* **2017,**29, 780–781. Chinese.

741 Wu, N.; Wang, J.; Wang, Y.; Ye, J.; Xu, Y.Z.; Huang, Y.F.; Sun, J. Analysis of surveillance data on main medical vectors in Nanshan district of Shenzhen 2013-2014. *Chin J of Public Health Eng* **2015,**14, 521–523+525. Chinese.

742 Wu, W.X.; Jin, Y.M.; Sun, L.Y.; Ceng, X.J.; Su, X.Y.; Jia, P.B.; Li, Z.; Lao, S.J. Analysis of results of sentinel monitoring of trandmission vector of dengue fever in Hainan Province in 2006. *China Tropical Medicine* **2007,**10, 1863–1864+1920. Chinese.

743 Wu, X.H.; Shi, H.X.; Tang, H.L. Epidemiological characteristics of Japanese encephalitis in Jinhua, China from 2007 to 2012. *Chin J Vector Biol & Control* **2013,**24, 549–551. Chinese.

744 Wu, Y.Q.; Zhang, G.Q. Observation diurnal activity of *Culex modestus* in Nanni Bay. *Negative* **1988,**2, 141–142. Chinese.

745 Wu, Z.M.; Zhu, H.M.; Chang, T.X.; Lv, S.C. Investigation of Mosquito Abundance and Composition around the Rare Birds National Nature Reserve of Yancheng, Jiangsu Province. *Chin J Parasitol Parasit Dis* **2007,**4, 310–313. Chinese.

746 Wu, S.; Huang, F.; Zhang, G.Q.; Pan, J.Y.; Wang, X.Z.; Zhuo, M.Y.J.; Hu, Y.H.; Shang, L.H. Nested PCR identified the malaria vector in Tibet malaria endemic area. *Chinese Journal of Zoonoses* **2010,**26, 648–650+653. Chinese.

747 Wu, Y.N.; Wen, S.; Liang, Q.G.; Yang, Q.; Wu, J.H.; Zhu, R.F.; Cheng, J.Z. The Preliminary Investigation on

Symbiotic Bacteria (Wolbachia) of Common Mosquito in Guiyang. *Journal of Guizhou Medical University* **2017,**42, 1130–1133. Chinese.
748 Wu, Z.R.; Wang, S.H.; Xu, Y.X. Investigation on vectors in Jiading district, Shanghai. *Chin J Vector Biol & Control* **2012,**23, 334–336. Chinese.
749 Xia, S.L.; Wang, T.; Shu, B.; Zhang, R.H.; Wu, H.D.; Luo, Y. Analysis on Results of Monitoring Dengue Fever and Investigation on Dengue Vector during 2005- 2007 in Zhongshan City. *J Trop Med* **2008,**6, 619–620+625. Chinese.
750 Xia, Y.; Liu, H.X.; Shang, H.; Li, Y.; Yin, W.S.; Cai, E.M. Investigation on mosquito infestations in the schools of Changning District in summer. *Chin J Hyg Insect & Equip* **2017,**23, 457–459. Chinese.
751 Xiao, W.; Liang, J.; Song, C.B. A report on the investigation results of rodent and vector insects in Panasonic Port. *Chinese Frontier Health Quarantine* **1997,**1, 30–32+63. Chinese.
752 Xie, R.H.; Zhu, H.P.; Fu, S.H.; Cheng, Y.K.; Xu, F.; Yao, P.P.; Yang, Z.N.; Zhou, X.L.; Zhu, Z.Y. Complete genome sequence analysis of Japanese encephalitis virus newly isolated in China. *Chinese J Exp Clin Virol* **2009,**4, 245–247. Chinese.
753 Xie, Y.H.; Tan, Y.; Bi, F.Y.; Zhou, K.J. Analysis on the surveillance results of dengue fever vectors in Guangxi from 2005 to 2008. *Chin J Vector Biol & Control* **2011,**22, 52–54. Chinese.
754 Xie, Y.H.; Tan, Y.; Yang, J.Y.; Bi, F.Y.; Mo, Z.J.; Zhou, K.J. Analysis of surveillance results of Japanese encephalitis vector mosquitoes in Guangxi from 2006 to 2007. *Applied Prev Med* **2011,**17, 302–304. Chinese.
755 Xu, B.H.; Wen, W.S. Mosquitoes and their Community characteristics in Bamboo Forest area of Meihuashan Nature Reserve. *Wuyi Science Journal* **1987,**7, 69–73. Chinese.
756 Xu, Y.X.; Li, H.P.; Xie, P.M.; Li, Y. Study on the current situation of vectors in Zhongxing town, Sichuan province after Wenchuan earthquake. *Chin J Vector Biol & Control* **2010,**21, 12–15. Chinese.
757 Xu, Z.L.; Yang, C.C.; Yu, Y.H. Monitor and control of biological vectors at Zhangjiagang port. *Chin J Hyg Insect & Equip* **2012,**18, 139–142+147. Chinese.
758 Xu, G.F.; Wang, Z.H. Analysis of investigation results of dengue fever vector in Zhangzhou City. *Strait J Prev Med* **2008,**14, 58–59. Chinese.
759 Xu, H.B.; Yang, H.Z.; Huang, Y.; Peng, Y.H.; Luo, Y.F.; Qin, Y.; Che, W.X.; Wang, C.Q.; Guo, X.X.; Xu, R, et al. Vector surveillance of dengue fever in Lancang County of Pu'er,Yunnan, 2014- 2018. *China Tropical Medicine* **2019,**19, 977–979+987. Chinese.
760 Xu, J.J.; Guan, L.R.; Luo, J.M. A supplementary description of the morphology of *Culex hortensis*, a new record in China. *Bull Dis Control Prev* **1987,**1, 46–47. Chinese.
761 Yan, Q.L.; Pan, D.G.; Zhang, J.M.; Lu, M.; Fu, Y.F.; Xiao, S. Study on the status of Medical Vector Biological population in Guangzhou New Baiyun International Airport. *Chinese Frontier Health Quarantine* **2007,**1, 27–35. Chinese.
762 Yan, G.J.; Zhang, H.Q.; Wang, Z.Z.; Ding, H.J.; Chen, Y.H.; Chen, Y.Z.; Li, F.; Xu, H.; Xue, H.Q.; Zhou, J.D, et al. Vector biological flora growth and decline investigation research in coastal shoal of Dongtai city. *J Med Pest Control* **2015,**31, 355–358+362. Chinese.
763 Yan, J.Y.; Pan, J.R.; Zhang, Y.J.; Zhou, J.Y.; Ni, H.N.; Zhang, Y.J. Study on the genotypic characteristics of Japanese encephalitis virus and serology in the healthy population of Zhejiang province, 2012 -2013. *Chin J Epidemiol* **2014,**35, 1146–1150. Chinese.

764 Yan, J.Y.; Tang, X.W.; Fan, F.N.; Zhang, Y.J.; Luo, Y.N.; Ying, Y.P.; Shen, J.; Zhang, Y.J. Study on the molecular characteristics of Japanese encephalitis virus living in vector mosquitoes in Zhejiang province 2009-2010. *Chin J Epidemiol* **2012,**1, 78–81. Chinese.

765 Yan, J.Y.; Wang, J.Y.; Wang, Z.F.; Yu, Z.W.; Yu, Z.Q.; Zhou, J.Y.; Zhang, Y.J. Identification of genotype III Japanese encephalitis virus isolated in Zhejiang province. *Chinese Journal of Preventive Medicine* **2012,**8, 722–727. Chinese.

766 Yan, S.W.; Sun, R.G.; Zhu, Z.K. Analysis of Vector Biological Surveillance results in Xinghua City in 2013. *Jiangsu J Prev Med* **2015,**26, 93–94. Chinese.

767 Yan, Y.H.; Shao, Y.L.; Kong, D.Y.; Li, X. Mosquito density in Shangrao City. *Chin J Hyg Insect ＆ Equip* **2019,**25, 71–73. Chinese.

768 Yan, Z.Q.; Hu, Z.G.; Jiang, Y.M.; Li, C.L.; Wu, H.Y.; Mai, W.L.; Hu, S.C. Surveillance for the vector of dengue fever in Guangzhou. *Chin J Hyg Insect ＆ Equip* **2010,**16, 42–44. Chinese.

769 Yang, H.X.; Li, J.Q. Japanese encephalitis vectors study of Baoshan city. *J Med Pest Control* **2015,**31, 377–380. Chinese.

770 Yang, H.M.; Guo, T.Y.; Wan, D.Z.; Liang, Z.P.; Huang, L.L. Investigation on medical vectors at Pingxiang railway port. *Chinese Frontier Health Quarantine* **2015,**38, 428–431. Chinese.

771 Yang, M.; Sun, Y.Q.; Gan, C.X.; Zhang, N.; Yang, C.Y.; Jing, Z.M.; Zhang, S.G. Monitoring and analysis of mosquito ecology in Jiangning District of Nanjing City in 2018. *Chin J Hyg Insect ＆ Equip* **2019,**25, 437–440. Chinese.

772 Yang, R.; Zheng, Y.T.; Yang, X.Y.; Dong, L.M.; Lin, Z.R.; Zhou, Y.W.; Ceng, X.C.; Li, H.B.; Jiang, J.Y. Investigation on malaria vectors in Jinghong, a border area in Yunnan Province. *Chin J Parasitol Parasit Dis* **2019,**37, 406–410+416. Chinese.

773 Yang, Y.Y. Analysis of vector monitoring results in Baoshan district, Shanghai from 2006 to 2008. *Chin J Vector Biol & Control* **2010,**21, 601–602. Chinese.

774 Yang, Y.H.; Quan, K.F. Analysis of population and density Monitoring results of the Four Pests in Qingshan Lake District of Nanchang City in 2011. *Journal of Nanchang University (Medical Sciences)* **2012,**52, 83–85. Chinese.

775 Yao, C.Q.; Xu, X.D. Mosquitoes breeding in tree holes in Shennongjia Forest region of Hubei Province. *J Med Pest Control* **1986,**3, 12–16. Chinese.

776 Yao, J.Y.; Chen, J.M.; Qiu, X.Z. Investigation on vectors in Zhangpu County in 2018. *Chin J Hyg Insect ＆ Equip* **2020,**26, 338–341. Chinese.

777 Ye, C.; Dong, B.; Ma, X.F.; Zhang, Y.A.; Sun, Y.J.; Hu, H. Surveillance and Analysis of dengue Fever Media in Suixi County, Anhui Province in 2015. *J Trop Dis Parasitol* **2016,**14, 172–173. Chinese.

778 Ye, H.B.; Feng, D.J.; Lan, Q.J. Investigation on medical vectors at Dongxing Port. *Chinese Frontier Health Quarantine* **2011,**34, 250–253. Chinese.

779 Ye, S.; Wen, H.Y.; Yu, Z.; Feng, Y.; Chen, S.; Ling, H. Sequence analysis of Japanese encephalitis viruses isolated from mosquitoes in the Three Gorges District in Chongqing,2012. *Chinese Journal of Zoonoses* **2014,**30, 622–627. Chinese.

780 Yi, J.P.; Liu, J.N.; Gao, M.L. Analysis of mosquito community structure in Zhoushan City. *Prev Med* **2021,**33, 84–86. Chinese.

781 Ying, K.M.; Zheng, B.F. Analysis of vector density surveillance in Pan'an county, Zhejiang province from 2005 to 2010. *Chin J Vector Biol & Control* **2011,**22, 262–264. Chinese.

782 You, X.C.; Su, S.; Shen, D.Y.; Zhang, X.G.; Huang, Z.S.; Zhuang, J.A.; Li, H.; Li, A.M.; Chen, J.S.; Jiang, Q.G, et al. Epidemiological investigation report on falciparum malaria in Huaibin County, Henan Province. *Journal of Zhengzhou University (Medical Sciences)* **1987,**1, 77–81. Chinese.

783 You, Z.Y.; Wang, Y.M.; Chen, Q.S.; Xu, P.T.; Zhao, D.Y.; Ge, J.Q.; Zhao, Z.J.; Zhang, H.F. Surveillance of two insect-borne viruses in Beijing. *Chinese Journal of Virology* **1987,**4, 326–331. Chinese.

784 You, Z.Y.; Wang, Y.M.; Zhao, Z.J.; Xu, P.T.; Zhao, Z.G.; Chen, Y.B.; Chen, W.Z.; Zhang, H.F. Preliminary identification of two arboviruses isolated from Hainan Island. *Chinese Journal of Virology* **1988,**1, 11–17. Chinese.

785 Yu, H. Notes on blood-sucking Diptera in Qinghai oil field. 1. pestiferous mosquito. *Acta Parasitol Med Entomol Sin* **1999,**3, 59. Chinese.

786 Yu, F.P.; Pan, H.M.; Wei, T.; Wu, J.C. Summary and evaluation on epidemiological situation, preventive and control procedure and elimination of malaria in Yichang City, Hubei. *China Tropical Medicine* **2016,**16, 528–532. Chinese.

787 Yu, J.; Shi, Q.M.; Chen, M.M.; Zhang, F.Q.; Zheng, Y.; Hu, X.B.; Hu, T.S.; Guo, P.; Gu, L.Q.; Li, M, et al. Composition and distribution of mosquitoes at the camp in Chayu county of Tibet, China. *Chin J Vector Biol & Control* **2014,**25, 441–443. Chinese.

788 Yu, J.; Wang, J.; Zhang, F.Q.; Zheng, Y.; Feng, Z.L.; Fan, Q.S. Field test of joint use of repellent and attractant for mosquito control. *Chin J Vector Biol & Control* **2013,**24, 193–195. Chinese.

789 Yu, Z.F.; Wang, W.X.; Xin, B.G.; Lin, S.B.; Yang, Z.R.; Wang, L.L. Study on the Transmission Media of Japanese Encephalitis in Heilongjiang Province. *Journal of Harbin Medical University* **1981,**3, 27–30. Chinese.

790 Yuan, Z.J.; Liu, W.; He, Y.W.; Meng, J.X.; Li, N.; Wang, J.L. Morphological and molecular identification of *Aedes* collected from Tumote and Alashan Left Banners of Inner Mongolia. *Chinese Frontier Health Quarantine* **2019,**42, 93–96. Chinese.

791 Yuan, Z.P. Vector Biology and Environmental quality Monitoring at Changbei International Airport. *Port Health Control* **2013,**18, 15–17. Chinese.

792 Yun, L.; Wang, F.C.; Zhang, Q.F.; Gao, Q.H.; Li, S.S.; Shang, X.L.; Chao, Y.S.; Liu, Y.Y.; Dong, R.X. Vectors monitoring results in International Horticultural Exposition Park and surrounding area in 2016. *Occup and Health* **2018,**34, 1105–1109. Chinese.

793 Zan, Y.; Deng, L.; Zhang, J.Q.; Liang, Y.; Liu, L. Analysis of mosquito density monitoring results in Shuangliu area of Chengdu from 2011 to 2015. *J Prev Med Inf* **2017,**33, 949–951. Chinese.

794 Zhan, Y.G.; Lin, H.F.; Hu, J.X.; Wang, M.; Yu, Y.W. Analysis of main Vector Biological Surveillance results of Yuetan Reservoir in Huangshan City in 2017. *J Trop Dis Parasitol* **2018,**16, 219–220+209. Chinese.

795 Zhang, B.F.; Li, X.L.; Sun, Y.X.; Zhang, Z.; She, J.J.; Yan, J.J. Investigation on mosquito population in Long county of Baoji city. *Chin J Hyg Insect & Equip* **2009,**15, 309–311. Chinese.

796 Zhang, B.; Zhao, J. Investigation on Species Composition and Seasonal Growth and Decline of Flies and Mosquitoes at Shenyang Taoxian International Airport. *Chinese Frontier Health Quarantine* **2010,**33, 402–404. Chinese.

797 Zhang, C.L.; Guo, X.R.; Yang, R.; Yang, Y.M.; Yang, Z.H.; Zheng, Y.T.; Wu, C.; Li, S.G.; Lin, Y.X.; Yu, G.C, et al. Evaluation on Malaria Hotspots in Yingjiang County of the China-Myanmar Border Area in 2015. *Chin J Parasitol Parasit Dis* **2016,**34, 430–434. Chinese.

798 Zhang, C.F.; Dong, Y.K.; Wang, Y.A.; Qiu, B.D.; Zheng, C.B. Investigation on nocturnal activity of adult mosquitoes in Linshu County in summer. *Journal of Shandong Medical College* **1993,**1, 43. Chinese.

799 Zhang, G.N.; Wu, D.C.; Li, X.; Guo, F.J.; Liu, L.; Wei, G.Z.; Xu, L.F. Malaria Surveillance in Later-stage of Control Program in Southwest Guizhou Province. *Parasitoses and Infectious Diseases* **2006,**3, 116–118. Chinese.

800 Zhang, H.L.; Gong, Z.D.; Mi, Z.Q.; Bai, D.Y.; Shi, H.F.; Zhang, Y.Z.; Yang, L.P.; Yang, G.L.; Xie, S.Q. Investigation of mosquitoes and isolation of Japanese Encephalitis virus in Ruili City, Yunnan Province. *J Med Pest Control* **1999,**2, 59–62. Chinese.

801 Zhang, H.L.; Mi, Z.Q.; Shi, H.F.; Gong, Z.D.; Zi, D.Y. Investigation on mosquitoes and arboviruses in Mengla County, Yunnan Province. *J Med Pest Control* **1999,**7, 337–340. Chinese.

802 Zhang, H.L.; Shi, H.F.; Gong, Z.D.; Hou, Z.L.; Mi, Z.Q.; Zi, D.Y. Distribution of mosquitoes and isolation of Japanese encephalitis virus in Shuangjiang county, Yunnan province. *J Med Pest Control* **1998,**6, 37–38. Chinese.

803 Zhang, H.L.; Shi, H.F.; Gong, Z.D.; Hou, Z.L.; Mi, Z.Q.; Zi, D.Y. Investigation of mosquitoes and arboviruses in Cangyuan county, Yunnan province. *J Med Pest Control* **2000,**3, 113–115. Chinese.

804 Zhang, H.L.; Shi, H.F.; Mi, Z.Q.; Zi, D.Y.; Gong, Z.D.; Zhang, Y.Z.; Huang, W.L.; Li, Z.X. Investigation of Arboviruses in Jinghong City, Yunnan Province, China. *Bull Dis Control Prev* **2000,**3, 40–44. Chinese.

805 Zhang, H.L.; Tao, S.J.; Yang, D.R.; Zhang, Y.Z.; Yang, W.H.; Zhang, Y.Z.; Huang, W.L.; Zhou, G.L.; Wang, H.Y.; Fu, S.H, et al. Isolation of Sindbis, Batai and Coltivirus in Yunnan province. *Chinese Journal of Zoonoses* **2005,**7, 548–551+557. Chinese.

806 Zhang, J.S.; Wang, D.M. Investigation and analysis of mosquitoes and flies density in Miyun District of Beijing in 2016. *Chin J Hyg Insect ＆ Equip* **2017,**23, 561–563. Chinese.

807 Zhang, J.K.; Lin, S.H.; Liu, X.C.; Chen, D.L.; Zhou, X.Y.; Cao, Y.O.; Wang, J.L.; Fu, S.H.; Zhang, Y.; Yang, J, et al. Survey on Arbovirus in Sichuan Province. *J Prev Med Inf* **2010,**26, 325–330. Chinese.

808 Zhang, J.; Ji, S.H.; Lu, C.H.; Leng, P.E.; Zhou, Y.B. Investigation on mosquito species in underground garage in Yangpu district of Shanghai. *Chin J Vector Biol & Control* **2017,**28, 38–41. Chinese.

809 Zhang, J.; Li, J.Q.; Feng, Y.; Han, Q.; Pan, H.; Yang, H.X.; Zhang, H.L.; Song, Z.Z. Identification of Japanese encephalitis virus genotype I from mosquitoes in Baoshan, Yunnan. *Chin J Parasitol Parasit Dis* **2018,**33, 150–154. Chinese.

810 Zhang, L.; Guo, F.Q. Surveillance of mosquitoes and flies in Changzhou Port in 2016. *Chin J Hyg Insect ＆ Equip* **2017,**23, 547–549. Chinese.

811 Zhang, M.J.; Lu, Y.C.; Zhang, J.Z. Monitoring report of Medical Vector Biology at Nantong Port. *Chinese Frontier Health Quarantine* **2005,**3, 144–147. Chinese.

812 Zhang, W.; Fang, Y.; Zhang, Y.; Shi, W.Q.; Li, Y.Y.; Yue, Z.Y. Study on kdr allele mutation of *Culex pipiens pallens* in Zichuan District, Zibo City, Shandong Province. *Chin J Schisto Control* **2019,**31, 134–138. Chinese.

813 Zhang, X.L.; Sun, X.H.; Cao, X.M.; Li, Y.P.; He, Z.H.; Fang, J.H.; Wang, J. A survey for mosquito at the

border between China and Burma. *Chin J Hyg Insect & Equip* **2012,**18, 125–127. Chinese.

814 Zhang, X.; Liu, Z.; Tan, Q.Q.; Zhang, H.; Zhou, H.Q.; Li, B.S.; Wu, D.; Cao, Y.X.; Wang, H.Y.; Liang, G.D., et al. Identification and Molecular characteristics of a New Orthomyxovirus (Longchuan virus). *Chinese Journal of Virology* **2019,**35, 599–605. Chinese.

815 Zhang, X.C.; Zhang, H.; Wang, H.P.; Wu, Y. General malaria epidemic situation,prevention and control process, control measures and evaluation of malaria elimination in Pingwu County, Sichuan. *China Tropical Medicine* **2016,**16, 552–555. Chinese.

816 Zhang, Y.P.; Wu, Z.Y.; Hu, W.X.; Wang, J.L.; Wang, W.Z.; Li, G.D.; Wang, L.M.; Shao, L.; Li, G.Z.; Wang, Q.J, et al. Analysis of epidemic factors of Japanese Encephalitis in Xihua County. *Henan J Prev Med* **1990,**4, 267–271+274. Chinese.

817 Zhang, Y.P.; Wu, Z.Y.; Li, L.C.; Guo, W.S.; Yu, F.X.; Wang, W.Z.; Xia, Z.G.; Zhao, Z.J.; Yue, M.J.; Jia, W, et al. Analysis of influencing factors of Japanese Encephalitis in Henan Province. *Henan J Prev Med* **1996,**3, 154–157. Chinese.

818 Zhang, Y.F.; Liu, D.P.; Yang, W.F.; Liu, H.; Chen, H.N.; Wu, Z.M.; Tian, Y.; Zhou, M.H.; Chu, H.L. Distribution characteristics of mosquito population in coastal beach areas of Jiangsu Province. *Jiangsu J Prev Med* **2018,**29, 568–570. Chinese.

819 Zhang, Y.Z.; Zhang, H.L.; Mi, Z.Q.; Gong, Z.D. Investigation on mosquitoes and insect vector viruses in Hekou City, Yunnan Province. *J Med Pest Control* **1998,**5, 37–38. Chinese.

820 Zhang, Z.X.; Zhou, H.N.; Zhao, X.T.; Chang, F.X.; Wang, H.J.; Li, X.J.; Zhuo, M.Y.J.; Ci, R.Q.Z.; Bian, M.Z.M.; Sang, D.L.M, et al. Epidemiological Survey on Malaria Situation in Motuo County of Tibet, China. *Chin J Parasitol Parasit Dis* **2008,**5, 343–348. Chinese.

821 Zhao, F.; Chen, H.; Liu, Y.; Zhong, W.; Shang, Z.P.; Chang, X.S. Investigation on vectors at Shuangliu International Airport in 2009~2011. *Chinese Frontier Health Quarantine* **2013,**36, 32–36. Chinese.

822 Zhao, Z.G.; Li, Z.L.; Wang, N.; Xu, J.H.; Wang, C.Z.; Chen, Y.B.; Su, S.Q.; Yang, J.; Fu, Z.C.; Chen, J.Y, et al. Study on Environmental and Community Dengue Fever Control in Hainan Island. *Chin J Vector Biol & Control* **1993,**5, 377–382. Chinese.

823 Zheng, K.; Huang, J.C.; Li, X.B.; Hong, Y.; Shi, Y.X.; Xing, L.Q.; Xiang, D.P.; Guo, B.X.; Hu, L.F. Rapid detection of Japanese encephalitis virus from mosquitoes by real-time fluorescence RT-PCR and is covery of genotype I Japanese encephalitis virus in Fujian Province. *Chin J Health Lab Tec* **2008,**11, 2212–2215. Chinese.

824 Zheng, Q.L.; Wang, Z.W.; Ding, F.; Chen, H.X. Malaria epidemiological characteristics and control strategies in Haining city from 1950 to 2018. *Modern Preventive Medicine* **2019,**46, 3795–3798. Chinese.

825 Zheng, Y.T.; Yang, M.D.; Zhou, K.M. Analysis on the surveillance results of dengue fever vectors in border areas of Yunnan province, 2016. *Chin J Vector Biol & Control* **2018,**29, 157–160. Chinese.

826 Zhou, A.Q.; Pan, M.; Zhu, H.B.; Li, P.P.; Zhang, S.L.; Ma, Y.F. Mosquito population density monitoring in urban and rural areas in Yandu District of Yancheng City in 2016 and 2017. *Chin J Hyg Insect & Equip* **2018,**24, 369–372. Chinese.

827 Zhou, H.N.; Zhang, Z.X.; C, .C.U.R.T.I.S.; N, .H.I.L.L.; Li, C.F.; Wu, C.; Wang, P.Y. Evaluation of the enzyme-linked immunosorbant assay in detecting circumsporozoite protein of *Anopheles* vectors in Yunnan. *Chin J Parasitol Parasit Dis* **2004,**4, 35–38. Chinese.

828 Zhou, J.M.; Wang, D.Q.; Lin, L.; Liu, Q.; Zhang, Q.W. Surveillance on mosquito vectors of dengue fever and dengue virus in Longgang district of Shenzhen city, 2009-2011. *Chin J Public Health* **2012,**28, 1628–1630. Chinese.

829 Zhou, J.J.; Huai, G.D.; Hua, J.X. Analysis of malaria prevalence in Jiangyin City of Jiangsu Province from 2010 to 2015. *J Med Pest Control* **2017,**33, 112–113+116. Chinese.

830 Zhou, T.; Zhang, H.L.; Li, M.H.; Wang, J.L.; Fu, S.H.; Feng, Y.; Liang, G.D. Investigation on arboviruses at Sino-Vietnam border areas in Wenshan of Yunnan province. *Chinese Journal of Preventive Medicine* **2009,**12, 1086–1090. Chinese.

831 Zhou, T.Y.; Gu, M.H.; Zhang, J.J.; Lu, B.F.; Lv, D.B. Surveillance of mosquitoes from 2008 to 2017 in Jiangyin City. *Chin J Hyg Insect & Equip* **2019,**25, 432–436. Chinese.

832 Zhou, W.; Xie, C.Y.; Li, Y.Q. Analysis of malaria epidemic situation in Nanjing city from 1995 to 2015. *J Trop Dis Parasitol* **2016,**14, 228–230+210. Chinese.

833 Zhou, Y.; Wang, J.; Tan, W.C.; Chen, M.; Zhu, Z.H.; Ma, J.Y.; Zheng, L.Q. An investigation of mosquito species and breeding habit in the residential area around Erhai Lake in Dali, Yunnan province, China. *Chin J Vector Biol & Control* **2019,**30, 56–59+68. Chinese.

834 Zhu, C.Y.; Zhao, C.C.; Lun, X.C.; Zhu, J.; Li, H.B.; Jiang, J.Y.; Yan, D.M.; Song, X.P.; Wang, J.; Meng, F.X., et al. Distribution of knockdown resistance genotypes in *Aedes albopictus* in Jinghong, Yunnan province, China, 2018-2019. *Chin J Vector Biol & Control* **2020,**31, 7–11. Chinese.

835 Zhu, H.B. Surveillance of vector biological density in Yandu District of Yancheng City in 2013. *Jiangsu Health Care* **2014,**16, 31–32. Chinese.

836 Zhu, H.M.; Zhuo, M.Y.; Zhang, H.; Wen, H. The epidemiological status and sustainability for malaria elimination in Nanping city. *Strait J Prev Med* **2014,**20, 9–10+29. Chinese.

837 Zhu, H.Q.; Zhang, L.X.; Yang, S.B.; Jin, D.L.; Feng, Y.L. Surveillance and Analysis of Malaria vectors in Yongsheng County, Yunnan Province. *Soft Science of Health* **2012,**26, 456–458. Chinese.

838 Zhu, Q.; Lin, Z.W.; Fu, J.C.; Fu, X.S. Study on the distribution and control of dengue fever vector in Haikou city. *Chin J Vector Biol & Control* **2008,**19, 553–556. Chinese.

839 Zou, C.Y.; Qu, D.J.; Wei, H.Y.; Lin, K.M.; Zheng, B.; Sun, Y.; Yan, G.Y.; Huang, Y.M. Study on the geographical distribution of population density of *Anopheles minimus* and molecular identification of the species in Guangxi. *Chin J Vector Biol & Control* **2012,**23, 101–104. Chinese.

840 Zou, Z.H.; Zhou, J.M.; Lin, L.; Liu, Q.; Zhang, Q.W.; Wang, D.Q. Survey on natural infection of Japanese encephalitis virus in mosquitoes in Longgang District of Shenzhen City. *Modern Preventive Medicine* **2012,**39, 5083–5085. Chinese.

841 Liu, K.L.; Liu, Y.; Wei, X.C. Resistance of *Aedes albopictus* to commonly used insecticides in Shenzhen, 2017. *Journal of the Graduates Sun Yat-Sen University* **2018,**4, 1–5. Chinese.

842 Chen, H.B. Descrtptions of the female and larva of *Culex* (Eumelanomyia) richei klken. *Acta Zootaxonomica Sinica* **1980,**3, 320–321. Chinese.

843 Hu, L.Z.; Kuang, X.L.; Huang, H.S.; Zhang, L.F. Process of malaria prevention and control and evaluation on malaria elimination measures in Gao'an City, Jiangxi Province. *China Tropical Medicine* **2016,**16, 680–684. Chinese.

844 Lin, M.H.; Wen, L.; Weng, S.W.; Li, C.Y.; Tao, Z.; Zhu, D.C.; Ceng, W.; Huang, S.L.; Zhang, L.X.; Chen,

X.5., et al. Effect in implementation of Global Fund Malaria Project in previously high malaria-endemic area of Wanning City. *China Tropical Medicine* **2013,**13, 674–676+683. Chinese.

845 Lv, S.Y.; Wang, J.L.; Li, N.; Hu, Q.; He, Y.W.; Zhao, D.H.; Chen, H.Y.; Li, H.C. Nucleotide detection of mosquito Japanese encephalitis virus and sequence analysis of PrM and E genes in upper Pearl River region. *China Animal Husbandry & Veterinary Medicine* **2017,**44, 336–343. Chinese.

846 Lv, X.J.; Fu, S.H.; Yang, Y.L.; He, H.H.; Zhang, G.J.; Chen, X.W.; Liang, G.D. Identification of XJ-90260 virus isolated in China as Western equine Encephalitis virus. *Chinese Journal of Virology* **2001,**4, 307–312. Chinese.

847 Ou, Y.Q.F.; Zheng, C.H.; Ke, Y.Z.; Liu, J.Z.; Wu, R.Q.; Liu, B.Y. Study on the Distribution characteristics and influencing factors of Medical Vector organisms in Quanzhou Petrochemical Port. *Port Health Control* **2013,**18, 51–55. Chinese.

848 Wang, J.; Xie, L.; Xie, X.L.; Dong, X.S.; Jiang, J.Y.; Yang, B.; Li, C.M.; Yang, Z.H.; Sun, X.D.; Lin, Z.R, et al. Dengue vectors and the natural infection in border with Laos, Jiangcheng county, China. *Chinese Journal of Zoonoses* **2016,**32, 843–849. Chinese.

849 Wang, W.; Yang, D.J.; Guo, J.; Wu, T.Y.; Qin, N.; Zhang, J.; Hou, H.G. Investigation on West Nile virus in mosquitoes in Tianjin. *Chin J Hyg Insect & Equip* **2013,**19, 432–433+435. Chinese.

850 Wu, T.Y.; Zhang, Y.M.; Zhang, J.; Qin, N.; Wang, W.; Li, P.Y.; Li, J.Y.; Hao, L.Y. Surveillance of the resistance of *Culex pipiens pallens* and Musca domestica to insecticides in Tianjin in 2010. *Chin J Vector Biol & Control* **2012,**23, 122–124. Chinese.

851 Xie, R.H.; Fu, S.H.; Cheng, Y.K.; Xu, F.; Yao, P.P.; Weng, J.Q.; Zhu, H.P.; Zhang, Y.J.; Zhu, Z.Y. Isolation and identification o f Japanese encephalitis virus from mosquitoes in Zhejiang province. *Chin J Epidemiol* **2008,**7, 712–715. Chinese.

852 Zhang, J.; Zhao, Z.Q. Investigation report on the species and seasonal growth and decline of Vector organisms at Tianjin Airport Port. *Port Health Control* **2009,**14, 34–38. Chinese.

853 Fu, S.H.; Song, S.; Liu, H.; Li, Y.Y.; Li, X.L.; Gao, X.Y.; Xu, Z.Q.; Liu, G.P.; Wang, D.M.; Tian, Z.Z, et al. ZIKA virus isolated from mosquitoes: a field and laboratory investigation in China, 2016. *Science China. Life sciences* **2017,**60, 1–8. .

854 Cao, Y.X.; Fu, S.H.; Tian, Z.F.; Lu, Z.; He, Y.; Wang, H.Y.; Wang, J.L.; Guo, W.D.; Tao, B.; Liang, G.D., et al. Distribution of mosquitoes and mosquito-borne arboviruses in Inner Mongolia, China. *Vector Borne Zoonotic Dis* **2011,**11, 1577–1581. .

855 Wu, D.; Tan, Q.Q.; Zhang, H.; Huang, P.; Zhou, H.Q.; Zhang, X.; Sun, J.F.; Huang, L.M.; Liang, G.D. Genomic and biological features of a novel orbivirus isolated from mosquitoes, in China. *Virus Research* **2020,**285, 197990. .

856 Guo, X.X.; Zhang, Y.M.; Li, C.X.; Zhang, G.L.; Zheng, C.; Dong, Y.D.; Xue, R.D.; Xing, D.; Zhao, T.Y. Host-Seeking Behavior and Arbovirus Detection in Mosquitoes of Habahe County, Xinjiang Uygur Autonomous Region, China. *J Am Mosq Control Assoc* **2015,**31, 329–335. .

857 Li, L.L.; Guo, X.F.; Zhao, Q.M.; Tong, Y.G.; Fan, H.; Sun, Q.; Xing, S.Z.; Zhou, H.N.; Zhang, J.S. Investigation on Mosquito-Borne Viruses at Lancang River and Nu River Watersheds in Southwestern China. *Vector Borne Zoonotic Dis* **2017,**17, 804–812. .

858 Li, Y.H.; Li, M.H.; Fu, S.H.; Chen, W.X.; Liu, Q.Y.; Zhang, H.L.; Da, W.; Hu, S.L.; Sang, D.L.M.; Bai, J, et

al. Japanese Encephalitis, Tibet, China. *Emerging Infectious Diseases* **2011,**17, 934–936. .
859 Liu, H.; Li, M.H.; Zhai, Y.G.; Meng, W.S.; Sun, X.H.; Cao, Y.X.; Fu, S.H.; Wang, H.Y.; Xu, L.H.; Tang, Q, et al. Banna virus, China, 1987-2007. *Emerging Infectious Diseases* **2010,**16, 514–517. .
860 Liu, H.; Zhang, X.; Li, L.X.; Shi, N.; Sun, X.T.; Liu, Q.; Jin, N.Y.; Si, X.K. First isolation and characterization of Getah virus from cattle in northeastern China. *BMC Veterinary Research* **2019,**15, 1. .
861 Liu, W.J.; Fu, S.H.; Ma, X.M.; Chen, X.J.; Wu, D.; Zhou, L.W.; Yin, Q.K.; Li, F.; He, Y.; Lei, W.W, et al. An outbreak of Japanese encephalitis caused by genotype Ib Japanese encephalitis virus in China, 2018: A laboratory and field investigation. *PLoS neglected tropical diseases* **2020,**14, e0008312. .
862 Shu, L.P.; Zuo, L.; Hao, M.; Zhao, X.; Wei, L.H. Investigation on dengue virus from *Aedes albopictus* field in Guizhou. *China Public Health* **2004,**5, 24–26. .
863 Hu, Q.; Chen, B.H.; Zhu, Z.R.; Tian, J.H.; Zhou, Y.; Zhang, X.Y.; Zheng, X. Recurrence of Japanese Encephalitis Epidemic in Wuhan, China, 2009-2010. *Plos One* **2013,**8, e52687. .
864 Sun, X.H.; Zhang, X.L.; Liu, Y.F.; Wang, J.; Wei, L.; Gao, X.; Zhao, W.; Zhang, X.; Yang, X.B.; Zhang, Y., et al. Isolation and identification of a Banna virus strain from mosquitoes in Yunnan province. *Chin J Epidemiol* **2010,**31, 685. Chinese.
865 Tao, Z.X.; Liu, G.F.; Wang, M.; Wang, H.Y.; Lin, X.J.; Song, L.Z.; Wang, H.Y.; Liu, X.D.; Cui, N.; Song, Y.Y, et al. Molecular Epidemiology of Japanese Encephalitis Virus in Mosquitoes during an Outbreak in China, 2013. *Scientific Reports* **2013,**4, 4908. .
866 Wang, H.Q.; Liu, W.B.; Yang, D.R.; Liang, Y.; Wang, J.W.; Zhang, L.S.; Liu, J.W.; Tao, S.J.; Lv, X.J.; Liang, G.D., et al. Isolation and identification of arboviruses in Hebei Province. *Chinese Journal of Experimental & Clinical Virology* **2006,**20, 52–55. Chinese.
867 Wang, J.L.; Li, H.C.; He, Y.W.; Zhou, Y.; Meng, J.X.; Zhu, W.Y.; Chen, H.Y.; Liao, D.F.; Man, Y.P. Isolation and Genetic Characterization of Mangshi Virus: A Newly Discovered Seadornavirus of the Reoviridae Family Found in Yunnan Province, China. *Plos One* **2015,**10, e0143601. .
868 Deng, X.; Yan, J.Y.; He, H.Q.; Yan, R.; Sun, Y.; Tang, X.W.; Zhou, Y.; Pan, J.H.; Mao, H.Y.; Zhang, Y.J, et al. Serological and molecular epidemiology of Japanese Encephalitis in Zhejiang, China, 2015-2018. *PLoS neglected tropical diseases* **2020,**14, e0008574. .
869 Yu, H.; Kong, Q.X.; Wang, J.; Qiu, X.F.; Wen, Y.Y.; Yu, X.F.; Liu, M.W.; Wang, H.Q.; Pan, J.C.; Sun, Z., et al. Multiple Lineages of Dengue Virus Serotype 2 Cosmopolitan Genotype Caused a Local Dengue Outbreak in Hangzhou, Zhejiang Province, China, in 2017. *Scientific Reports* **2019,**9, 7345. .
870 Fang, Y.; Zhang, Y.; Zhou, Z.B.; Xia, S.; Shi, W.Q.; Xue, J.B.; Li, Y.Y.; Wu, J.T. New strains of Japanese encephalitis virus circulating in Shanghai, China after a ten-year hiatus in local mosquito surveillance. *Parasites & Vectors* **2019,**12, 22. .
871 Feng, Y.; Fu, S.H.; Wang, W.H.; Zhang, Y.Z.; He, B.; Tu, C.C.; Liang, G.D.; Zhang, H.L. Isolation and full-length genome analysis of mosquito-borne Manzanilla virus from Yunnan Province, China. *Bmc Research Notes* **2015,**8, 255. .
872 Zhang, H.L.; Zhang, Y.Z.; Yang, W.H.; Feng, Y.; Nasci, R.S.; Yang, J.; Liu, Y.H.; Dong, C.L.; Li, S.; Zhang, B.S, et al. Mosquitoes of Western Yunnan Province, China: Seasonal Abundance, Diversity, and Arbovirus Associations. *Plos One* **2013,**8, e77017. .
873 Lu, B.L.; Chen, H.B.; Li, B.S.; Yu, H. Supplement to "a checklist of Chinese mosquitoes (Diptera:Culiciade)".

Acta Parasitol Med Entomol Sin **1997,**2, 25–34. .
874 Cao, H. The study of mosquito population dynamics and the effect of different monitoring methods in Huangpu District of Shanghai Expoarea. Master thesis, Fudan University, 2010. Chinese.
875 Cao, Y.X. Study on arboviruses isolated from mosquitoes collected in Hunan and Tibet, China. Dissertation, Chinese Center for Disease Control and Prevention, 2013. Chinese.
876 Cheng, R. Arbovirus isolation and identification of mosquitoes and midges collected in Inner Mongolia province in China. Master thesis, Qingdao University, 2019. Chinese.
877 Chu, H.L. Study on *Culex tritaeniorhynchus* population characters and infection with Japanese encephalitis virus. Dissertation, Academy of Military Medical Sciences, 2017. Chinese.
878 Fan, F.N.; Chen, G.H.; Ye, J.J. Molecular Epidemiological study on Japanese Encephalovirus in mosquito Vector in Cixi City, Zhejiang Province. In The 20th academic Conference on Health Reform and Development at the Grass-roots level in Zhejiang Province, 2012; Zhejiang, Chinese.
879 Fu, F.Y. Bioassasy of deltamethrin resistance and preliminary study on its mechanism of *Anopheles sinensis* populations. Master thesis, Chongqing Normal University, 2013. Chinese.
880 Gong, D.F. Study of the communistic characteristics of vector mosquito and the prevalent situation of Japanese Encephalitis in Yuanjiang-Red River, Yunnan province. Master thesis, Dali University, 2010. Chinese.
881 Guo, X.F. Investigation on Mosquitoes and Mosquito-borne viruses at Lancang River Watershed in Yunnan province. Master thesis, Academy of Military Medical Sciences, 2014. Chinese.
882 He, Y.M. Study on the correlation between mosquito ecology, meteorological factors, and mosquito-borne diseases in the Wanzhou section of the Three Gorges Reservoir Region. Master thesis, Third Military Medical University, 2018. Chinese.
883 He, X.X. Screening and research on common pathogens of viral encephalitis in some regions of Fujian province, 2010-2011. Master thesis, Inner MongoliaAgricultural University, 2012. Chinese.
884 Jia, H.L. Epidemiological analysis and etilogical investigation of Japanese encephalitis in Anhui province. Master thesis, Chinese Center for Disease Control and Prevention, 2012. Chinese.
885 Jiang, H.Y. Investigation of Japanese encephalitis virus infection status in the Three Gorges Reservoir Area, China. Master thesis, Shandong University, 2010. Chinese.
886 Li, D. Investigation on mosquito fauna and Micronecta formosana Matsumura, a Natural Enemy of mosquitoes in east Guangdong province. Master thesis, Shantou University, 2010. Chinese.
887 Li, L.L. Molecular biology characteristics of newly discovered mosquito-borne viruses and investigation of pathogens on the wild rodents in Yunnan province. Master thesis, Anhui Medical University, 2018. Chinese.
888 Li, M.H. Isolation and identification for arboviruses isolated in mosquitoes collected in Shanxi Gansu and Jiangxi provinces. Master thesis, Chinese Center for Disease Control and Prevention, 2009. Chinese.
889 Li, W.B. Epidemiologic and etiologic study of Japanese encephalitis in Henan province. Master thesis, Shandong University, 2005. Chinese.
890 Li, W.J. Mosquitoes and mosquito-borne arboviruses and the relation with local Human disease in Qinghai province. Dissertation, Shandong University, 2011. Chinese.
891 Liang, L.L. Study on density monitoring and prevention and control measures of dengue vector *Aedes albopictus* in Guangdzhou. Master thesis, Guangdong Pharmaceutical University, 2019. Chinese.

892 Liu, H. Research on molecular epidemiology,diagnosis and recombinant vaccine of Japanese encephalitis virus. Dissertation, Jilin University, 2013. Chinese.
893 Liu, P.B. Population Genetics of *Aedes aegypti* invading in Southwest Yunnan. Master thesis, Chinese Center for Disease Control and Prevention, 2018. Chinese.
894 Lu, Y.L. Investigation of the mosquito vector of Japanese Encephalitis in Mohan Port and Guanlei Port in the Border Area of Yunnan Province. Master thesis, Dali University, 2012. Chinese.
895 Lu, M.L. Isolation and Identification of Arboviruses (mosquito and midge)of Shanghai. Master thesis, Changchun University of Science and Technology, 2017. Chinese.
896 Luo, L.F. Surveillance Distribution of Vectors and Chemical Resistance of *Aedes* AlboPictus in Pingshan District of. Master thesis, Guangdong Pharmaceutical University, 2015. Chinese.
897 Lv, Z. Arboviruses investigation in Xinjiang--Tahana virus and human infection. Dissertation, Chinese Center for Disease Control and Prevention, 2009. Chinese.
898 Ma, S.Y. Evaluation of Killing effect of Bacillus thuringiensis israelensis on *Culex tritaeniorhynchus* larvae in China- Myanmar border area of Yunnan Province. Master thesis, Third Military Medical University, 2013. Chinese.
899 Ren, X.J. Distribution of Mosquitoes and Mosquito-Borne Arboviruses in South of Shanxi, China. Master thesis, Shanxi Medical University, 2017. Chinese.
900 Shi, Y.Y.; Liu, Z.J.; Wang, X.M.; Di, W.J.X. Effect of killing mosquitoes and flies in Wudu earthquack-hit areas in Longnan county. In The 25th National academic Exchange and Product Exhibition on Insecticidal and Insecticidal Pharmacy, 2008; Hunan, Chinese.
901 Tang, C.J. Liao Ning virus investigation in Liaoning, Xinjiang and Yunnan provinces and molecular characterization of Liao Ning virus isolates. Master thesis, Chinese Center for Disease Control and Prevention, 2013. Chinese.
902 Wang, A.D. Identification of vetor species and investigation of diseases in Alashankou Port. Master thesis, Shihezi University, 2016. Chinese.
903 Wang, J.Y. The Survey on Density Monitoring and Virus Infection for Mosquitoes and Rodents in Dapeng New District of Shenzhen. Master thesis, Guangdong Pharmaceutical University, 2018. Chinese.
904 Wang, J.L. Distribution and molecular characteristics of arboviruses in Yunnan province near the China-Myanmar-Laos border. Dissertation, Chinese Center for Disease Control and Prevention, 2010. Chinese.
905 Wang, J.W. Investigation of Arboviruses in Liaoning and Heilongjiang Provinces. Master thesis, Shanxi Medical University, 2005. Chinese.
906 Wu, R.Q. Study on the Distribution of Medical Vectors Population on the south of Meizhou Bay Port and Flies Population Molecular identification. Master thesis, Fujian Medical University, 2013. Chinese.
907 Wu, B. Investigation of mosquito and its relationship with diseases in Yuanjiang county valley of Yunnan province. Master thesis, Dali University, 2013. Chinese.
908 Xiao, Y. The distribution of *Aedes albopictus* in Guangzhou and its association with meteorological factors and the occurrence of dengue fever. Master thesis, Guangdong Pharmaceutical University, 2017. Chinese.
909 Xie, Y.; Weng, T.Y.; Liao, H.Y. Molecular epidemiological study of the first local dengue fever case in Foshan in 2015. In 2015 Hot Symposium on the Prevention and Control of New Infectious Diseases, 2015; Guangdong, Chinese.

910 Yang, D.Z. Effects of seasonal climatic variation on the biological characteristics of *Aedes albopictus* in Guangzhou. Master thesis, Southern Medical University, 2019. Chinese.

911 Yang, X.Y. Investigations of mosquito species and arbovirus in the part areas of Chuxiong prefecture, Yunnan. Master thesis, Dali University, 2015. Chinese.

912 Zhang, H.J. Analysis of surveillance results from 2009 to 2015 and study on the potential distribution and resistance of *Aedes albopictus* in Beijing Chaoyang district. Master thesis, Academy of Military Medical Sciences, 2016. Chinese.

913 Zhang, W.J. Mosquitoes and mosquito-borne arboviruses in Shandong province. Master thesis, Shandong University, 2018. Chinese.

914 Zheng, Y.Y. Arboviruses investigation in Shanxi and Henan provinces and molecular evolutionary genetics analysus of Tibet novel Orbiviruses. Dissertation, Shandong University, 2014. Chinese.

915 Zhou, J.M. Molecular Epidemiological Study of Some Mosquito-borne Arbovirus in Longgang District, Shenzhen city. Master thesis, Guangdong Pharmaceutical University, 2013. Chinese.

916 Zhu, L.P. Surveillance of the Medical Vectors and Research on the Insecticide Resistance of *Aedes* Albopictus and Blattella Germanica in Dapeng District of Shenzhen City. Master thesis, Guangdong Pharmaceutical University, 2018. Chinese.

917 Li, M.H.; Fu, S.H.; Wang, H.Y.; Cao, Y.X.; Liang, G.D. Isolation of Genotype V JEV in China. In The fourth National Symposium on zoonosis, 2014; Jilin, Chinese.

918 Liu, X.N. Study on density monitoring, virus carrying and insecticide resistance of *Aedes* albopitus in Pingshan district of Shenzhen city. Master thesis, Guangdong Pharmaceutical University, 2018. Chinese.

919 Song, S. Arbovirus isolation and identification of mosquitoes and midges collected in Guizhou and Sichuan province in China. Master thesis, Qingdao University, 2018. Chinese.

920 Tan, J. The survey of potential risk factors of dengue fever in Guanlei port of Xishuangbanna, the frontiers of Yunnan Province. Master thesis, Dali University, 2012. Chinese.

921 Wang, W.M.; Zhou, H.Y.; Liu, Y.B.; Li, J.L.; Cao, Y.Y.; Cao, J. Comparative observation on seasonal fluctuation and nocturnal activity of *Anopheles sinensis* in different areas of Jiangsu Province. In The 13th Congress and Symposium of Jiangsu insect Society, Jiangsu, Chinese.

922 Chen, C. Study on the Transmission cycle of JEV in Huaihua City, Hunan Province. Master thesis, Academy of Military Medical Sciences, 2015. Chinese.
